# Supplementary material for: Analysis of Cohort Stepped Wedge Cluster‐Randomized Trials With Nonignorable Dropout via Joint Modeling
Source: Stat Med. 2025 Feb 18;44(5):e10347. doi: 10.1002/sim.10347 (PMC11833761; doi:10.1002/sim.10347)
Supplement: Supplementary file 1 — Data S1.Supporting Information. [file SIM-44-0-s001.pdf]

Supplementary material: *Analysis of cohort stepped wedge cluster-randomized trials with non-ignorable dropout via joint modeling*

Authors: Alessandro Gasparini[1,2], Michael J. Crowther[1], Emiel O. Hoogendijk[3], Fan Li[†,4,5], Michael O. Harhay[†,6,7]

1. Red Door Analytics AB, Stockholm, Sweden
2. Department of Medical Epidemiology and Biostatistics, Karolinska Institutet, Stockholm, Sweden
3. Department of Epidemiology and Data Science, Amsterdam Public Health Research Institute, Amsterdam UMC – Location VU University Medical Center, Amsterdam, Netherlands
4. Department of Biostatistics, Yale School of Public Health, Connecticut, USA
5. Center for Methods in Implementation and Prevention Science, Yale School of Public Health, Connecticut, USA
6. Department of Biostatistics, Epidemiology, and Informatics, Perelman School of Medicine at the University of Pennsylvania, Pennsylvania, USA
7. MRC Clinical Trials Unit, University College London, London, UK

Correspondence to Alessandro Gasparini, e-mail: alessandro.gasparini@reddooranalytics.se. Note that the † symbol in the authors list denotes shared co-senior authorship.

Contents

A Expected Monte Carlo standard error for bias **2**

B Additional results for the simulation studies **3**

B.1 Convergence rates . . . 3
B.2 Constant intervention model . . . 5
B.3 General time on treatment model . . . 34
B.4 Neutral simulation scenario . . . 81
B.4.1 Constant intervention model . . . 81
B.4.2 General time on treatment model . . . 81
B.5 Monte Carlo standard errors . . . 90

C Improving convergence of the joint model estimation procedure **94**

D Reanalysis of the 'Frail Older Adults: Care in Transition' trial, additional results **94**

Appendix A Expected Monte Carlo standard error for bias

We start by simulating a small, preliminary batch of repetitions to inform our choice regarding the total number of simulations. Specifically, we run 50 repetitions for both the constant intervention and general time on treatment scenarios and summarize these results. We calculate the maximum, for each model and across scenarios, of either empirical or model-based variances for the treatment effect parameters $(\delta, \delta_0, \ldots, \delta_3)$, denoted with $\widehat{\text{var}}$, and plug that into the formula (based on the definition of Monte Carlo standard error for bias) $n_{\text{sim}} = \widehat{\text{var}}/\text{MCSE}^2$. $\widehat{\text{var}}$ was 0.344 and 2.724 for the constant intervention effect and general time on treatment models, respectively.

We use the definition of Monte Carlo standard error for bias to calculate the expected Monte Carlo error with a fixed number of 1000 iterations, which would be approximately 0.052 for the largest $\widehat{\text{var}}$. Given the magnitude of the treatment effects assumed for the different data-generating mechanisms, the expected Monte Carlo error would range between 2.09% and 0.17% of the different treatment effects parameters, which we deem acceptable. Thus, we run 1000 repetitions of each simulation study.

Note that we study Monte Carlo standard errors in more detail later on in this document, in Appendix B.5.

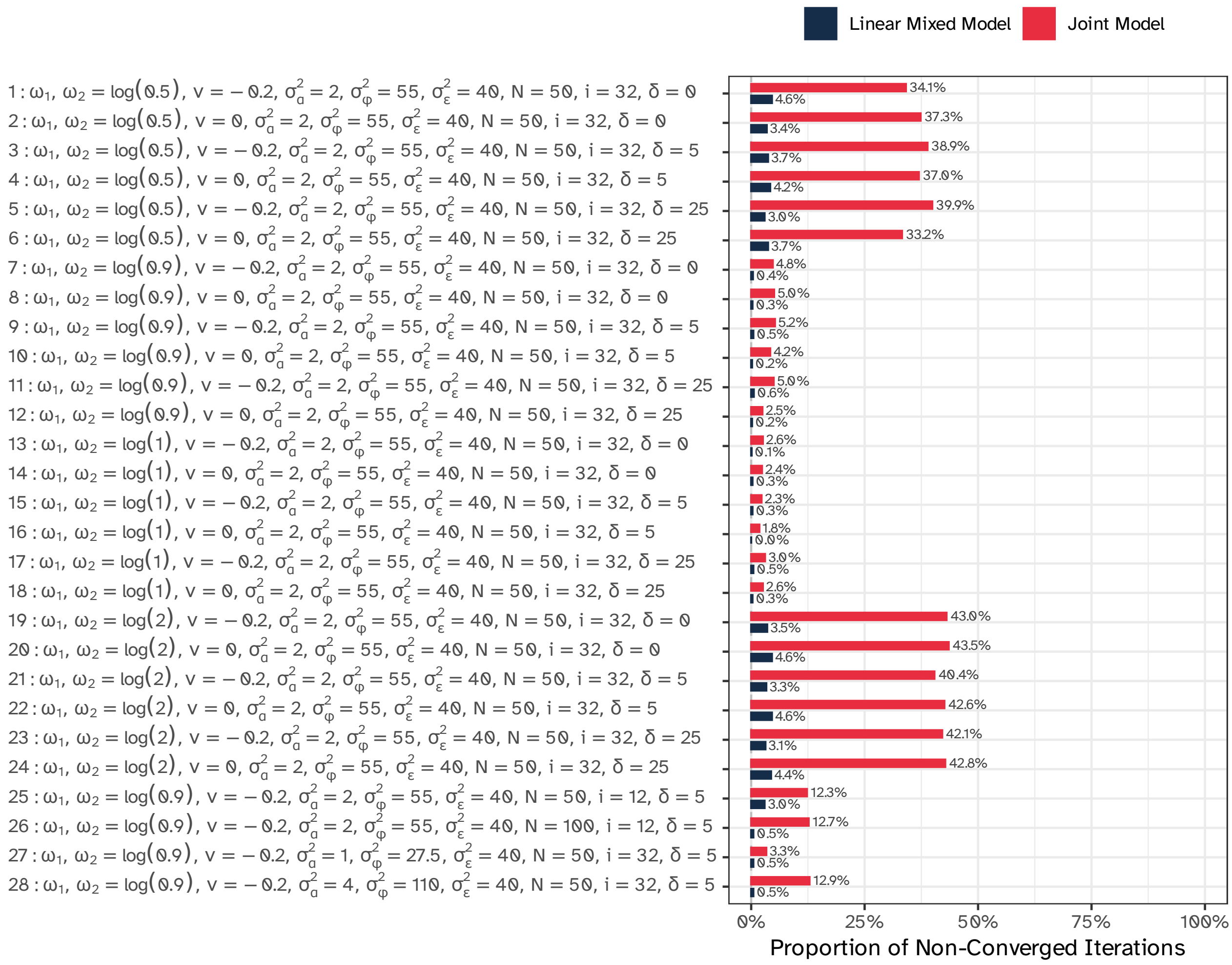

Figure B1: Proportion of non-converged repetitions for the joint and linear mixed models, by simulation scenario, for the constant intervention model. Note that N denotes the number of participants per cluster, and i the total number of clusters (product of number of cluster per intervention sequence and number of intervention sequences).

Appendix B Additional results for the simulation studies

B.1 Convergence rates

Convergence rates across all simulation scenarios based on the joint model, for both the constant intervention and general time on treatment models, are depicted in Figures B1 and B2, respectively.

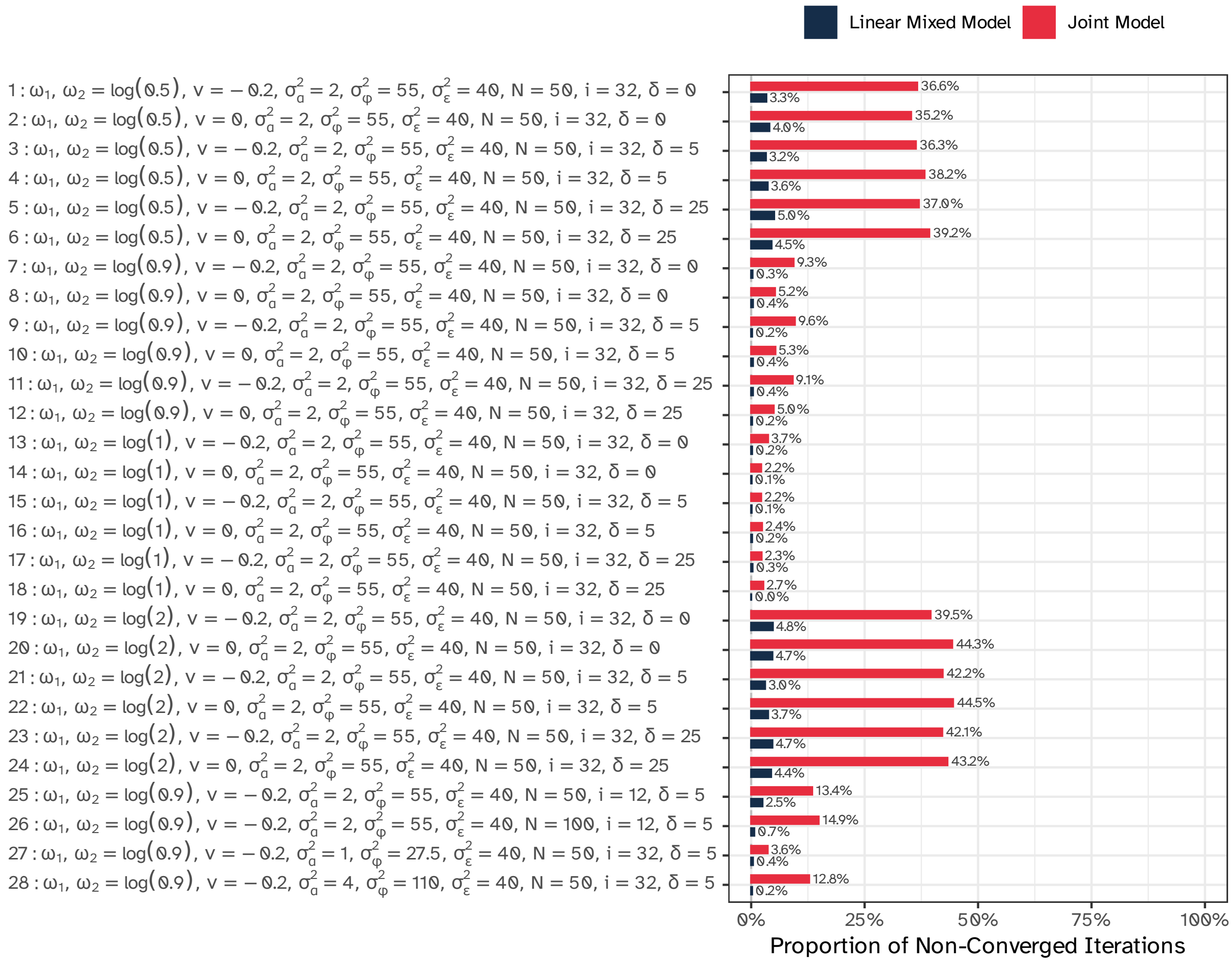

Figure B2: Proportion of non-converged repetitions for the joint and the linear mixed model, by simulation scenario, for the general time on treatment model. Note that N denotes the number of participants per cluster, and i the total number of clusters (product of number of cluster per intervention sequence and number of intervention sequences).

B.2 Constant intervention model

Bias (with 95% C.I. based on Monte Carlo standard errors) for the treatment effect on the longitudinal outcome are tabulated in Table B1, with relative bias in Table B2. Coverage probabilities are tabulated in Table B3.

Bias, relative bias, and coverage probabilities for the period effects are tabulated in Tables B4, B5 and B6, respectively.

Bias, relative bias, and coverage probabilities for the intra-class correlation coefficients (ICCs) are tabulated in Tables B7, B8 and B9, respectively.

Finally, bias, relative bias, and coverage probabilities for the variance components are tabulated in Tables B10, B11 and B12, respectively.

Table B1: Bias of treatment effect on the longitudinal outcome for the constant treatment effect parametrisation, with 95% confidence intervals based on Monte Carlo errors. LMM denotes the linear mixed model, while JM denotes the joint model. Statistically significant biases are highlighted in bold.

Scenario	LMM	JM
**Main scenarios:**		
$\omega_1 = \omega_2 = \log(0.5)$, $\delta = 0.0$, $\nu = -0.2$	**-0.026 (-0.047, -0.005)**	0.003 (-0.021, 0.028)
$\omega_1 = \omega_2 = \log(0.5)$, $\delta = 0.0$, $\nu = 0.0$	-0.005 (-0.026, 0.017)	0.001 (-0.025, 0.027)
$\omega_1 = \omega_2 = \log(0.5)$, $\delta = 5.0$, $\nu = -0.2$	**-0.027 (-0.049, -0.005)**	0.014 (-0.012, 0.040)
$\omega_1 = \omega_2 = \log(0.5)$, $\delta = 5.0$, $\nu = 0.0$	-0.010 (-0.031, 0.012)	-0.023 (-0.048, 0.002)
$\omega_1 = \omega_2 = \log(0.5)$, $\delta = 25$, $\nu = -0.2$	**-0.034 (-0.055, -0.013)**	0.009 (-0.018, 0.036)
$\omega_1 = \omega_2 = \log(0.5)$, $\delta = 25$, $\nu = 0.0$	-0.019 (-0.041, 0.003)	-0.012 (-0.038, 0.013)
$\omega_1 = \omega_2 = \log(0.9)$, $\delta = 0.0$, $\nu = -0.2$	**-0.042 (-0.063, -0.020)**	0.002 (-0.019, 0.024)
$\omega_1 = \omega_2 = \log(0.9)$, $\delta = 0.0$, $\nu = 0.0$	-0.003 (-0.025, 0.019)	-0.002 (-0.023, 0.020)
$\omega_1 = \omega_2 = \log(0.9)$, $\delta = 5.0$, $\nu = -0.2$	**-0.052 (-0.074, -0.031)**	-0.010 (-0.032, 0.011)
$\omega_1 = \omega_2 = \log(0.9)$, $\delta = 5.0$, $\nu = 0.0$	0.016 (-0.005, 0.038)	0.016 (-0.005, 0.038)
$\omega_1 = \omega_2 = \log(0.9)$, $\delta = 25$, $\nu = -0.2$	**-0.040 (-0.061, -0.019)**	0.004 (-0.017, 0.026)
$\omega_1 = \omega_2 = \log(0.9)$, $\delta = 25$, $\nu = 0.0$	**0.024 (0.002, 0.045)**	0.018 (-0.003, 0.040)
$\omega_1 = \omega_2 = \log(1.0)$, $\delta = 0.0$, $\nu = -0.2$	-0.007 (-0.028, 0.014)	-0.007 (-0.028, 0.014)
$\omega_1 = \omega_2 = \log(1.0)$, $\delta = 0.0$, $\nu = 0.0$	0.010 (-0.011, 0.032)	0.010 (-0.012, 0.031)
$\omega_1 = \omega_2 = \log(1.0)$, $\delta = 5.0$, $\nu = -0.2$	-0.001 (-0.023, 0.021)	-0.003 (-0.025, 0.019)
$\omega_1 = \omega_2 = \log(1.0)$, $\delta = 5.0$, $\nu = 0.0$	-0.006 (-0.028, 0.016)	-0.007 (-0.029, 0.016)
$\omega_1 = \omega_2 = \log(1.0)$, $\delta = 25$, $\nu = -0.2$	0.016 (-0.006, 0.037)	0.017 (-0.005, 0.039)
$\omega_1 = \omega_2 = \log(1.0)$, $\delta = 25$, $\nu = 0.0$	0.019 (-0.002, 0.041)	0.018 (-0.004, 0.040)
$\omega_1 = \omega_2 = \log(2.0)$, $\delta = 0.0$, $\nu = -0.2$	**0.036 (0.015, 0.058)**	0.008 (-0.019, 0.034)
$\omega_1 = \omega_2 = \log(2.0)$, $\delta = 0.0$, $\nu = 0.0$	-0.001 (-0.022, 0.020)	-0.004 (-0.031, 0.023)
$\omega_1 = \omega_2 = \log(2.0)$, $\delta = 5.0$, $\nu = -0.2$	0.014 (-0.008, 0.035)	-0.005 (-0.031, 0.022)
$\omega_1 = \omega_2 = \log(2.0)$, $\delta = 5.0$, $\nu = 0.0$	0.001 (-0.020, 0.022)	**0.029 (0.002, 0.056)**
$\omega_1 = \omega_2 = \log(2.0)$, $\delta = 25$, $\nu = -0.2$	**0.032 (0.010, 0.053)**	0.007 (-0.020, 0.034)
$\omega_1 = \omega_2 = \log(2.0)$, $\delta = 25$, $\nu = 0.0$	0.003 (-0.018, 0.024)	0.016 (-0.011, 0.043)
**Additional scenarios:**		

Table B1: Bias of treatment effect on the longitudinal outcome for the constant treatment effect parametrisation, with 95% confidence intervals based on Monte Carlo errors. LMM denotes the linear mixed model, while JM denotes the joint model. Statistically significant biases are highlighted in bold. *(continued)*

Scenario	LMM	JM
$i = 3 \times 4$	**-0.069 (-0.106, -0.033)**	-0.026 (-0.063, 0.011)
$i = 3 \times 4, N = 100$	**-0.043 (-0.067, -0.018)**	-0.006 (-0.031, 0.019)
$\sigma_\alpha^2 = 1, \sigma_\phi^2 = 27.5$	**-0.036 (-0.056, -0.017)**	0.005 (-0.015, 0.024)
$\sigma_\alpha^2 = 4, \sigma_\phi^2 = 110$	**-0.027 (-0.048, -0.005)**	0.015 (-0.007, 0.037)

Table B2: Relative bias of treatment effect on the longitudinal outcome for the constant treatment effect parametrisation, with 95% confidence intervals based on Monte Carlo errors. LMM denotes the linear mixed model, while JM denotes the joint model. Statistically significant biases are highlighted in bold.

Scenario	LMM	JM
**Main scenarios:**		
$\omega_1 = \omega_2 = \log(0.5), \delta = 0.0, \nu = -0.2$	—	—
$\omega_1 = \omega_2 = \log(0.5), \delta = 0.0, \nu = 0.0$	—	—
$\omega_1 = \omega_2 = \log(0.5), \delta = 5.0, \nu = -0.2$	**-0.005 (-0.010, -0.001)**	0.003 (-0.002, 0.008)
$\omega_1 = \omega_2 = \log(0.5), \delta = 5.0, \nu = 0.0$	-0.002 (-0.006, 0.002)	-0.005 (-0.010, 0.000)
$\omega_1 = \omega_2 = \log(0.5), \delta = 25, \nu = -0.2$	**-0.001 (-0.002, -0.001)**	0.000 (-0.001, 0.001)
$\omega_1 = \omega_2 = \log(0.5), \delta = 25, \nu = 0.0$	-0.001 (-0.002, 0.000)	-0.000 (-0.002, 0.001)
$\omega_1 = \omega_2 = \log(0.9), \delta = 0.0, \nu = -0.2$	—	—
$\omega_1 = \omega_2 = \log(0.9), \delta = 0.0, \nu = 0.0$	—	—
$\omega_1 = \omega_2 = \log(0.9), \delta = 5.0, \nu = -0.2$	**-0.010 (-0.015, -0.006)**	-0.002 (-0.006, 0.002)
$\omega_1 = \omega_2 = \log(0.9), \delta = 5.0, \nu = 0.0$	0.003 (-0.001, 0.008)	0.003 (-0.001, 0.008)
$\omega_1 = \omega_2 = \log(0.9), \delta = 25, \nu = -0.2$	**-0.002 (-0.002, -0.001)**	0.000 (-0.001, 0.001)
$\omega_1 = \omega_2 = \log(0.9), \delta = 25, \nu = 0.0$	**0.001 (0.000, 0.002)**	0.001 (-0.000, 0.002)
$\omega_1 = \omega_2 = \log(1.0), \delta = 0.0, \nu = -0.2$	—	—
$\omega_1 = \omega_2 = \log(1.0), \delta = 0.0, \nu = 0.0$	—	—
$\omega_1 = \omega_2 = \log(1.0), \delta = 5.0, \nu = -0.2$	-0.000 (-0.005, 0.004)	-0.001 (-0.005, 0.004)
$\omega_1 = \omega_2 = \log(1.0), \delta = 5.0, \nu = 0.0$	-0.001 (-0.006, 0.003)	-0.001 (-0.006, 0.003)
$\omega_1 = \omega_2 = \log(1.0), \delta = 25, \nu = -0.2$	0.001 (-0.000, 0.001)	0.001 (-0.000, 0.002)
$\omega_1 = \omega_2 = \log(1.0), \delta = 25, \nu = 0.0$	0.001 (-0.000, 0.002)	0.001 (-0.000, 0.002)
$\omega_1 = \omega_2 = \log(2.0), \delta = 0.0, \nu = -0.2$	—	—
$\omega_1 = \omega_2 = \log(2.0), \delta = 0.0, \nu = 0.0$	—	—
$\omega_1 = \omega_2 = \log(2.0), \delta = 5.0, \nu = -0.2$	0.003 (-0.002, 0.007)	-0.001 (-0.006, 0.004)
$\omega_1 = \omega_2 = \log(2.0), \delta = 5.0, \nu = 0.0$	0.000 (-0.004, 0.004)	**0.006 (0.000, 0.011)**
$\omega_1 = \omega_2 = \log(2.0), \delta = 25, \nu = -0.2$	**0.001 (0.000, 0.002)**	0.000 (-0.001, 0.001)

Table B2: Relative bias of treatment effect on the longitudinal outcome for the constant treatment effect parametrisation, with 95% confidence intervals based on Monte Carlo errors. LMM denotes the linear mixed model, while JM denotes the joint model. Statistically significant biases are highlighted in bold. *(continued)*

Scenario	LMM	JM
$\omega_1 = \omega_2 = \log(2.0)$, $\delta = 25$, $\nu = 0.0$	0.000 (-0.001, 0.001)	0.001 (-0.000, 0.002)
**Additional scenarios:**		
$i = 3 \times 4$	**-0.014 (-0.021, -0.007)**	-0.005 (-0.013, 0.002)
$i = 3 \times 4, N = 100$	**-0.009 (-0.013, -0.004)**	-0.001 (-0.006, 0.004)
$\sigma^2_\alpha = 1, \sigma^2_\phi = 27.5$	**-0.007 (-0.011, -0.003)**	0.001 (-0.003, 0.005)
$\sigma^2_\alpha = 4, \sigma^2_\phi = 110$	**-0.005 (-0.010, -0.001)**	0.003 (-0.001, 0.007)

Table B3: Coverage probability of treatment effect on the longitudinal outcome for the constant treatment effect parametrisation, with 95% confidence intervals based on Monte Carlo errors. LMM denotes the linear mixed model, while JM denotes the joint model.

Scenario	LMM	JM
**Main scenarios:**		
$\omega_1 = \omega_2 = \log(0.5)$, $\delta = 0.0$, $\nu = -0.2$	0.960 (0.948, 0.972)	0.964 (0.950, 0.978)
$\omega_1 = \omega_2 = \log(0.5)$, $\delta = 0.0$, $\nu = 0.0$	0.941 (0.926, 0.955)	0.950 (0.933, 0.967)
$\omega_1 = \omega_2 = \log(0.5)$, $\delta = 5.0$, $\nu = -0.2$	0.938 (0.923, 0.953)	0.950 (0.933, 0.967)
$\omega_1 = \omega_2 = \log(0.5)$, $\delta = 5.0$, $\nu = 0.0$	0.943 (0.929, 0.958)	0.946 (0.928, 0.963)
$\omega_1 = \omega_2 = \log(0.5)$, $\delta = 25$, $\nu = -0.2$	0.959 (0.947, 0.971)	0.957 (0.941, 0.973)
$\omega_1 = \omega_2 = \log(0.5)$, $\delta = 25$, $\nu = 0.0$	0.952 (0.938, 0.965)	0.954 (0.939, 0.970)
$\omega_1 = \omega_2 = \log(0.9)$, $\delta = 0.0$, $\nu = -0.2$	0.949 (0.936, 0.963)	0.958 (0.946, 0.970)
$\omega_1 = \omega_2 = \log(0.9)$, $\delta = 0.0$, $\nu = 0.0$	0.954 (0.941, 0.967)	0.958 (0.945, 0.970)
$\omega_1 = \omega_2 = \log(0.9)$, $\delta = 5.0$, $\nu = -0.2$	0.949 (0.936, 0.963)	0.957 (0.944, 0.969)
$\omega_1 = \omega_2 = \log(0.9)$, $\delta = 5.0$, $\nu = 0.0$	0.957 (0.945, 0.969)	0.958 (0.946, 0.971)
$\omega_1 = \omega_2 = \log(0.9)$, $\delta = 25$, $\nu = -0.2$	0.951 (0.938, 0.964)	0.954 (0.941, 0.967)
$\omega_1 = \omega_2 = \log(0.9)$, $\delta = 25$, $\nu = 0.0$	0.957 (0.945, 0.969)	0.956 (0.944, 0.969)
$\omega_1 = \omega_2 = \log(1.0)$, $\delta = 0.0$, $\nu = -0.2$	0.953 (0.941, 0.966)	0.952 (0.939, 0.965)
$\omega_1 = \omega_2 = \log(1.0)$, $\delta = 0.0$, $\nu = 0.0$	0.954 (0.941, 0.967)	0.955 (0.942, 0.968)
$\omega_1 = \omega_2 = \log(1.0)$, $\delta = 5.0$, $\nu = -0.2$	0.955 (0.943, 0.968)	0.954 (0.941, 0.967)
$\omega_1 = \omega_2 = \log(1.0)$, $\delta = 5.0$, $\nu = 0.0$	0.949 (0.935, 0.962)	0.947 (0.933, 0.960)
$\omega_1 = \omega_2 = \log(1.0)$, $\delta = 25$, $\nu = -0.2$	0.946 (0.933, 0.960)	0.944 (0.930, 0.958)
$\omega_1 = \omega_2 = \log(1.0)$, $\delta = 25$, $\nu = 0.0$	0.947 (0.934, 0.961)	0.946 (0.932, 0.960)
$\omega_1 = \omega_2 = \log(2.0)$, $\delta = 0.0$, $\nu = -0.2$	0.961 (0.949, 0.973)	0.963 (0.948, 0.978)
$\omega_1 = \omega_2 = \log(2.0)$, $\delta = 0.0$, $\nu = 0.0$	0.956 (0.943, 0.969)	0.953 (0.936, 0.970)
$\omega_1 = \omega_2 = \log(2.0)$, $\delta = 5.0$, $\nu = -0.2$	0.945 (0.931, 0.959)	0.949 (0.932, 0.966)

Table B3: Coverage probability of treatment effect on the longitudinal outcome for the constant treatment effect parametrisation, with 95% confidence intervals based on Monte Carlo errors. LMM denotes the linear mixed model, while JM denotes the joint model. *(continued)*

Scenario	LMM	JM
$\omega_1 = \omega_2 = \log(2.0)$, $\delta = 5.0$, $\nu = 0.0$	0.949 (0.935, 0.963)	0.935 (0.916, 0.955)
$\omega_1 = \omega_2 = \log(2.0)$, $\delta = 25$, $\nu = -0.2$	0.950 (0.936, 0.963)	0.949 (0.932, 0.966)
$\omega_1 = \omega_2 = \log(2.0)$, $\delta = 25$, $\nu = 0.0$	0.962 (0.950, 0.974)	0.960 (0.944, 0.976)
**Additional scenarios:**		
$i = 3 \times 4$	0.944 (0.930, 0.958)	0.949 (0.935, 0.963)
$i = 3 \times 4, N = 100$	0.954 (0.941, 0.967)	0.961 (0.948, 0.973)
$\sigma^2_\alpha = 1, \sigma^2_\phi = 27.5$	0.955 (0.942, 0.968)	0.960 (0.947, 0.972)
$\sigma^2_\alpha = 4, \sigma^2_\phi = 110$	0.960 (0.948, 0.972)	0.956 (0.943, 0.970)

Table B4: Bias of period effects on the longitudinal outcome for the constant treatment effect parametrisation, with 95% confidence intervals based on Monte Carlo errors. LMM denotes the linear mixed model, while JM denotes the joint model. Statistically significant biases are highlighted in bold.

Scenario	Parameter	LMM	JM
**Main scenarios:**			
$\omega_1 = \omega_2 = \log(0.5)$, $\delta = 0.0$, $\nu = -0.2$	β_1	**5.060 (5.042, 5.078)**	**0.950 (0.919, 0.981)**
$\omega_1 = \omega_2 = \log(0.5)$, $\delta = 0.0$, $\nu = -0.2$	β_2	**5.426 (5.407, 5.446)**	**0.988 (0.955, 1.020)**
$\omega_1 = \omega_2 = \log(0.5)$, $\delta = 0.0$, $\nu = -0.2$	β_3	**5.575 (5.554, 5.596)**	**0.990 (0.956, 1.024)**
$\omega_1 = \omega_2 = \log(0.5)$, $\delta = 0.0$, $\nu = -0.2$	β_4	**5.672 (5.648, 5.696)**	**0.994 (0.957, 1.030)**
$\omega_1 = \omega_2 = \log(0.5)$, $\delta = 0.0$, $\nu = -0.2$	β_5	**5.733 (5.704, 5.761)**	**1.001 (0.960, 1.042)**
$\omega_1 = \omega_2 = \log(0.5)$, $\delta = 0.0$, $\nu = 0.0$	β_1	**5.055 (5.037, 5.072)**	**0.964 (0.933, 0.995)**
$\omega_1 = \omega_2 = \log(0.5)$, $\delta = 0.0$, $\nu = 0.0$	β_2	**5.427 (5.409, 5.446)**	**0.989 (0.957, 1.021)**
$\omega_1 = \omega_2 = \log(0.5)$, $\delta = 0.0$, $\nu = 0.0$	β_3	**5.576 (5.554, 5.597)**	**0.989 (0.954, 1.024)**
$\omega_1 = \omega_2 = \log(0.5)$, $\delta = 0.0$, $\nu = 0.0$	β_4	**5.699 (5.675, 5.722)**	**1.015 (0.978, 1.052)**
$\omega_1 = \omega_2 = \log(0.5)$, $\delta = 0.0$, $\nu = 0.0$	β_5	**5.756 (5.728, 5.784)**	**1.016 (0.973, 1.058)**
$\omega_1 = \omega_2 = \log(0.5)$, $\delta = 5.0$, $\nu = -0.2$	β_1	**5.051 (5.033, 5.069)**	**0.969 (0.938, 1.000)**
$\omega_1 = \omega_2 = \log(0.5)$, $\delta = 5.0$, $\nu = -0.2$	β_2	**5.412 (5.394, 5.431)**	**0.968 (0.936, 1.001)**
$\omega_1 = \omega_2 = \log(0.5)$, $\delta = 5.0$, $\nu = -0.2$	β_3	**5.571 (5.551, 5.592)**	**0.989 (0.954, 1.025)**
$\omega_1 = \omega_2 = \log(0.5)$, $\delta = 5.0$, $\nu = -0.2$	β_4	**5.662 (5.637, 5.686)**	**0.984 (0.945, 1.023)**
$\omega_1 = \omega_2 = \log(0.5)$, $\delta = 5.0$, $\nu = -0.2$	β_5	**5.734 (5.705, 5.763)**	**1.005 (0.962, 1.048)**
$\omega_1 = \omega_2 = \log(0.5)$, $\delta = 5.0$, $\nu = 0.0$	β_1	**5.035 (5.018, 5.053)**	**0.946 (0.917, 0.975)**
$\omega_1 = \omega_2 = \log(0.5)$, $\delta = 5.0$, $\nu = 0.0$	β_2	**5.411 (5.392, 5.429)**	**0.980 (0.949, 1.012)**
$\omega_1 = \omega_2 = \log(0.5)$, $\delta = 5.0$, $\nu = 0.0$	β_3	**5.574 (5.554, 5.595)**	**1.008 (0.975, 1.041)**
$\omega_1 = \omega_2 = \log(0.5)$, $\delta = 5.0$, $\nu = 0.0$	β_4	**5.685 (5.661, 5.709)**	**1.022 (0.985, 1.058)**
$\omega_1 = \omega_2 = \log(0.5)$, $\delta = 5.0$, $\nu = 0.0$	β_5	**5.741 (5.713, 5.769)**	**1.039 (0.999, 1.078)**

Table B4: Bias of period effects on the longitudinal outcome for the constant treatment effect parametrisation, with 95% confidence intervals based on Monte Carlo errors. LMM denotes the linear mixed model, while JM denotes the joint model. Statistically significant biases are highlighted in bold. *(continued)*

Scenario	Parameter	LMM	JM
$\omega_1 = \omega_2 = \log(0.5), \delta = 25, \nu = -0.2$	β_1	**5.058 (5.040, 5.076)**	**0.965 (0.932, 0.999)**
$\omega_1 = \omega_2 = \log(0.5), \delta = 25, \nu = -0.2$	β_2	**5.430 (5.411, 5.449)**	**0.980 (0.945, 1.015)**
$\omega_1 = \omega_2 = \log(0.5), \delta = 25, \nu = -0.2$	β_3	**5.587 (5.566, 5.608)**	**0.995 (0.958, 1.032)**
$\omega_1 = \omega_2 = \log(0.5), \delta = 25, \nu = -0.2$	β_4	**5.679 (5.654, 5.703)**	**1.001 (0.961, 1.041)**
$\omega_1 = \omega_2 = \log(0.5), \delta = 25, \nu = -0.2$	β_5	**5.745 (5.717, 5.772)**	**1.001 (0.958, 1.044)**
$\omega_1 = \omega_2 = \log(0.5), \delta = 25, \nu = 0.0$	β_1	**5.057 (5.040, 5.074)**	**0.982 (0.954, 1.011)**
$\omega_1 = \omega_2 = \log(0.5), \delta = 25, \nu = 0.0$	β_2	**5.421 (5.403, 5.438)**	**0.988 (0.959, 1.018)**
$\omega_1 = \omega_2 = \log(0.5), \delta = 25, \nu = 0.0$	β_3	**5.600 (5.579, 5.621)**	**1.018 (0.986, 1.050)**
$\omega_1 = \omega_2 = \log(0.5), \delta = 25, \nu = 0.0$	β_4	**5.692 (5.668, 5.715)**	**1.035 (1.001, 1.068)**
$\omega_1 = \omega_2 = \log(0.5), \delta = 25, \nu = 0.0$	β_5	**5.762 (5.734, 5.789)**	**1.034 (0.995, 1.073)**
$\omega_1 = \omega_2 = \log(0.9), \delta = 0.0, \nu = -0.2$	β_1	**1.457 (1.436, 1.477)**	-0.022 (-0.046, 0.002)
$\omega_1 = \omega_2 = \log(0.9), \delta = 0.0, \nu = -0.2$	β_2	**1.954 (1.932, 1.976)**	-0.026 (-0.053, 0.000)
$\omega_1 = \omega_2 = \log(0.9), \delta = 0.0, \nu = -0.2$	β_3	**2.215 (2.191, 2.239)**	-0.021 (-0.050, 0.009)
$\omega_1 = \omega_2 = \log(0.9), \delta = 0.0, \nu = -0.2$	β_4	**2.372 (2.344, 2.400)**	-0.027 (-0.060, 0.005)
$\omega_1 = \omega_2 = \log(0.9), \delta = 0.0, \nu = -0.2$	β_5	**2.489 (2.456, 2.522)**	-0.018 (-0.055, 0.020)
$\omega_1 = \omega_2 = \log(0.9), \delta = 0.0, \nu = 0.0$	β_1	**1.465 (1.445, 1.486)**	-0.017 (-0.042, 0.007)
$\omega_1 = \omega_2 = \log(0.9), \delta = 0.0, \nu = 0.0$	β_2	**1.969 (1.946, 1.991)**	-0.025 (-0.052, 0.003)
$\omega_1 = \omega_2 = \log(0.9), \delta = 0.0, \nu = 0.0$	β_3	**2.243 (2.218, 2.267)**	-0.019 (-0.049, 0.010)
$\omega_1 = \omega_2 = \log(0.9), \delta = 0.0, \nu = 0.0$	β_4	**2.423 (2.395, 2.452)**	-0.007 (-0.040, 0.026)
$\omega_1 = \omega_2 = \log(0.9), \delta = 0.0, \nu = 0.0$	β_5	**2.546 (2.514, 2.578)**	-0.003 (-0.040, 0.033)
$\omega_1 = \omega_2 = \log(0.9), \delta = 5.0, \nu = -0.2$	β_1	**1.468 (1.447, 1.489)**	-0.008 (-0.033, 0.016)
$\omega_1 = \omega_2 = \log(0.9), \delta = 5.0, \nu = -0.2$	β_2	**1.960 (1.937, 1.984)**	-0.015 (-0.043, 0.013)
$\omega_1 = \omega_2 = \log(0.9), \delta = 5.0, \nu = -0.2$	β_3	**2.224 (2.198, 2.249)**	-0.007 (-0.037, 0.023)
$\omega_1 = \omega_2 = \log(0.9), \delta = 5.0, \nu = -0.2$	β_4	**2.381 (2.352, 2.409)**	-0.012 (-0.045, 0.021)
$\omega_1 = \omega_2 = \log(0.9), \delta = 5.0, \nu = -0.2$	β_5	**2.494 (2.460, 2.527)**	-0.010 (-0.047, 0.028)
$\omega_1 = \omega_2 = \log(0.9), \delta = 5.0, \nu = 0.0$	β_1	**1.460 (1.439, 1.481)**	-0.020 (-0.045, 0.005)
$\omega_1 = \omega_2 = \log(0.9), \delta = 5.0, \nu = 0.0$	β_2	**1.961 (1.939, 1.983)**	**-0.030 (-0.057, -0.003)**
$\omega_1 = \omega_2 = \log(0.9), \delta = 5.0, \nu = 0.0$	β_3	**2.240 (2.215, 2.265)**	-0.017 (-0.047, 0.013)
$\omega_1 = \omega_2 = \log(0.9), \delta = 5.0, \nu = 0.0$	β_4	**2.405 (2.376, 2.433)**	-0.021 (-0.054, 0.012)
$\omega_1 = \omega_2 = \log(0.9), \delta = 5.0, \nu = 0.0$	β_5	**2.521 (2.489, 2.553)**	-0.021 (-0.057, 0.016)
$\omega_1 = \omega_2 = \log(0.9), \delta = 25, \nu = -0.2$	β_1	**1.481 (1.460, 1.502)**	-0.002 (-0.027, 0.022)
$\omega_1 = \omega_2 = \log(0.9), \delta = 25, \nu = -0.2$	β_2	**1.968 (1.946, 1.990)**	-0.015 (-0.042, 0.012)
$\omega_1 = \omega_2 = \log(0.9), \delta = 25, \nu = -0.2$	β_3	**2.228 (2.203, 2.254)**	-0.012 (-0.042, 0.019)
$\omega_1 = \omega_2 = \log(0.9), \delta = 25, \nu = -0.2$	β_4	**2.378 (2.350, 2.407)**	-0.023 (-0.056, 0.010)
$\omega_1 = \omega_2 = \log(0.9), \delta = 25, \nu = -0.2$	β_5	**2.507 (2.475, 2.538)**	-0.007 (-0.043, 0.029)
$\omega_1 = \omega_2 = \log(0.9), \delta = 25, \nu = 0.0$	β_1	**1.479 (1.458, 1.500)**	-0.003 (-0.028, 0.023)
$\omega_1 = \omega_2 = \log(0.9), \delta = 25, \nu = 0.0$	β_2	**1.973 (1.950, 1.995)**	-0.019 (-0.047, 0.008)

Table B4: Bias of period effects on the longitudinal outcome for the constant treatment effect parametrisation, with 95% confidence intervals based on Monte Carlo errors. LMM denotes the linear mixed model, while JM denotes the joint model. Statistically significant biases are highlighted in bold. *(continued)*

Scenario	Parameter	LMM	JM
$\omega_1 = \omega_2 = \log(0.9)$, $\delta = 25$, $\nu = 0.0$	β_3	**2.248 (2.223, 2.273)**	-0.011 (-0.041, 0.019)
$\omega_1 = \omega_2 = \log(0.9)$, $\delta = 25$, $\nu = 0.0$	β_4	**2.398 (2.369, 2.427)**	-0.027 (-0.061, 0.006)
$\omega_1 = \omega_2 = \log(0.9)$, $\delta = 25$, $\nu = 0.0$	β_5	**2.525 (2.492, 2.558)**	-0.017 (-0.054, 0.020)
$\omega_1 = \omega_2 = \log(1.0)$, $\delta = 0.0$, $\nu = -0.2$	β_1	**-0.022 (-0.044, -0.000)**	**-0.026 (-0.049, -0.003)**
$\omega_1 = \omega_2 = \log(1.0)$, $\delta = 0.0$, $\nu = -0.2$	β_2	-0.016 (-0.039, 0.007)	-0.019 (-0.044, 0.005)
$\omega_1 = \omega_2 = \log(1.0)$, $\delta = 0.0$, $\nu = -0.2$	β_3	-0.015 (-0.040, 0.010)	-0.018 (-0.044, 0.008)
$\omega_1 = \omega_2 = \log(1.0)$, $\delta = 0.0$, $\nu = -0.2$	β_4	-0.007 (-0.036, 0.022)	-0.011 (-0.042, 0.019)
$\omega_1 = \omega_2 = \log(1.0)$, $\delta = 0.0$, $\nu = -0.2$	β_5	-0.008 (-0.040, 0.025)	-0.010 (-0.044, 0.024)
$\omega_1 = \omega_2 = \log(1.0)$, $\delta = 0.0$, $\nu = 0.0$	β_1	0.003 (-0.020, 0.026)	0.007 (-0.016, 0.031)
$\omega_1 = \omega_2 = \log(1.0)$, $\delta = 0.0$, $\nu = 0.0$	β_2	0.010 (-0.013, 0.034)	0.018 (-0.007, 0.043)
$\omega_1 = \omega_2 = \log(1.0)$, $\delta = 0.0$, $\nu = 0.0$	β_3	0.006 (-0.020, 0.033)	0.013 (-0.014, 0.041)
$\omega_1 = \omega_2 = \log(1.0)$, $\delta = 0.0$, $\nu = 0.0$	β_4	0.007 (-0.023, 0.036)	0.015 (-0.016, 0.046)
$\omega_1 = \omega_2 = \log(1.0)$, $\delta = 0.0$, $\nu = 0.0$	β_5	0.008 (-0.025, 0.041)	0.016 (-0.019, 0.051)
$\omega_1 = \omega_2 = \log(1.0)$, $\delta = 5.0$, $\nu = -0.2$	β_1	-0.007 (-0.029, 0.015)	-0.014 (-0.036, 0.009)
$\omega_1 = \omega_2 = \log(1.0)$, $\delta = 5.0$, $\nu = -0.2$	β_2	-0.015 (-0.038, 0.009)	-0.019 (-0.043, 0.006)
$\omega_1 = \omega_2 = \log(1.0)$, $\delta = 5.0$, $\nu = -0.2$	β_3	-0.020 (-0.047, 0.006)	-0.025 (-0.053, 0.002)
$\omega_1 = \omega_2 = \log(1.0)$, $\delta = 5.0$, $\nu = -0.2$	β_4	0.002 (-0.027, 0.032)	-0.004 (-0.035, 0.028)
$\omega_1 = \omega_2 = \log(1.0)$, $\delta = 5.0$, $\nu = -0.2$	β_5	0.001 (-0.032, 0.034)	-0.002 (-0.037, 0.033)
$\omega_1 = \omega_2 = \log(1.0)$, $\delta = 5.0$, $\nu = 0.0$	β_1	-0.008 (-0.031, 0.015)	-0.011 (-0.034, 0.013)
$\omega_1 = \omega_2 = \log(1.0)$, $\delta = 5.0$, $\nu = 0.0$	β_2	-0.007 (-0.031, 0.018)	-0.009 (-0.034, 0.017)
$\omega_1 = \omega_2 = \log(1.0)$, $\delta = 5.0$, $\nu = 0.0$	β_3	-0.001 (-0.028, 0.026)	-0.005 (-0.033, 0.023)
$\omega_1 = \omega_2 = \log(1.0)$, $\delta = 5.0$, $\nu = 0.0$	β_4	-0.007 (-0.037, 0.024)	-0.009 (-0.041, 0.023)
$\omega_1 = \omega_2 = \log(1.0)$, $\delta = 5.0$, $\nu = 0.0$	β_5	-0.001 (-0.036, 0.035)	-0.004 (-0.040, 0.033)
$\omega_1 = \omega_2 = \log(1.0)$, $\delta = 25$, $\nu = -0.2$	β_1	-0.001 (-0.023, 0.021)	-0.001 (-0.024, 0.022)
$\omega_1 = \omega_2 = \log(1.0)$, $\delta = 25$, $\nu = -0.2$	β_2	-0.007 (-0.030, 0.017)	-0.005 (-0.029, 0.020)
$\omega_1 = \omega_2 = \log(1.0)$, $\delta = 25$, $\nu = -0.2$	β_3	-0.002 (-0.028, 0.025)	-0.002 (-0.030, 0.026)
$\omega_1 = \omega_2 = \log(1.0)$, $\delta = 25$, $\nu = -0.2$	β_4	0.001 (-0.030, 0.031)	0.001 (-0.031, 0.034)
$\omega_1 = \omega_2 = \log(1.0)$, $\delta = 25$, $\nu = -0.2$	β_5	-0.018 (-0.051, 0.015)	-0.019 (-0.054, 0.016)
$\omega_1 = \omega_2 = \log(1.0)$, $\delta = 25$, $\nu = 0.0$	β_1	-0.005 (-0.027, 0.017)	-0.001 (-0.024, 0.021)
$\omega_1 = \omega_2 = \log(1.0)$, $\delta = 25$, $\nu = 0.0$	β_2	0.003 (-0.021, 0.027)	0.011 (-0.014, 0.037)
$\omega_1 = \omega_2 = \log(1.0)$, $\delta = 25$, $\nu = 0.0$	β_3	-0.020 (-0.046, 0.007)	-0.011 (-0.039, 0.017)
$\omega_1 = \omega_2 = \log(1.0)$, $\delta = 25$, $\nu = 0.0$	β_4	-0.014 (-0.043, 0.016)	-0.002 (-0.033, 0.029)
$\omega_1 = \omega_2 = \log(1.0)$, $\delta = 25$, $\nu = 0.0$	β_5	-0.023 (-0.057, 0.011)	-0.011 (-0.047, 0.025)
$\omega_1 = \omega_2 = \log(2.0)$, $\delta = 0.0$, $\nu = -0.2$	β_1	**-5.047 (-5.065, -5.029)**	**-0.943 (-0.975, -0.910)**
$\omega_1 = \omega_2 = \log(2.0)$, $\delta = 0.0$, $\nu = -0.2$	β_2	**-5.418 (-5.437, -5.399)**	**-0.984 (-1.017, -0.950)**
$\omega_1 = \omega_2 = \log(2.0)$, $\delta = 0.0$, $\nu = -0.2$	β_3	**-5.581 (-5.602, -5.560)**	**-0.993 (-1.028, -0.957)**
$\omega_1 = \omega_2 = \log(2.0)$, $\delta = 0.0$, $\nu = -0.2$	β_4	**-5.686 (-5.710, -5.661)**	**-1.018 (-1.057, -0.979)**

Table B4: Bias of period effects on the longitudinal outcome for the constant treatment effect parametrisation, with 95% confidence intervals based on Monte Carlo errors. LMM denotes the linear mixed model, while JM denotes the joint model. Statistically significant biases are highlighted in bold. *(continued)*

Scenario	Parameter	LMM	JM
$\omega_1 = \omega_2 = \log(2.0)$, $\delta = 0.0$, $\nu = -0.2$	β_5	**-5.752 (-5.780, -5.723)**	**-1.019 (-1.062, -0.977)**
$\omega_1 = \omega_2 = \log(2.0)$, $\delta = 0.0$, $\nu = 0.0$	β_1	**-5.042 (-5.060, -5.024)**	**-0.911 (-0.942, -0.879)**
$\omega_1 = \omega_2 = \log(2.0)$, $\delta = 0.0$, $\nu = 0.0$	β_2	**-5.415 (-5.434, -5.397)**	**-0.943 (-0.977, -0.909)**
$\omega_1 = \omega_2 = \log(2.0)$, $\delta = 0.0$, $\nu = 0.0$	β_3	**-5.586 (-5.607, -5.565)**	**-0.963 (-0.999, -0.927)**
$\omega_1 = \omega_2 = \log(2.0)$, $\delta = 0.0$, $\nu = 0.0$	β_4	**-5.669 (-5.694, -5.644)**	**-0.961 (-1.002, -0.920)**
$\omega_1 = \omega_2 = \log(2.0)$, $\delta = 0.0$, $\nu = 0.0$	β_5	**-5.739 (-5.767, -5.712)**	**-0.972 (-1.015, -0.928)**
$\omega_1 = \omega_2 = \log(2.0)$, $\delta = 5.0$, $\nu = -0.2$	β_1	**-5.042 (-5.060, -5.023)**	**-0.945 (-0.977, -0.913)**
$\omega_1 = \omega_2 = \log(2.0)$, $\delta = 5.0$, $\nu = -0.2$	β_2	**-5.415 (-5.434, -5.396)**	**-0.982 (-1.014, -0.949)**
$\omega_1 = \omega_2 = \log(2.0)$, $\delta = 5.0$, $\nu = -0.2$	β_3	**-5.562 (-5.582, -5.541)**	**-0.982 (-1.018, -0.946)**
$\omega_1 = \omega_2 = \log(2.0)$, $\delta = 5.0$, $\nu = -0.2$	β_4	**-5.657 (-5.681, -5.633)**	**-0.993 (-1.031, -0.955)**
$\omega_1 = \omega_2 = \log(2.0)$, $\delta = 5.0$, $\nu = -0.2$	β_5	**-5.711 (-5.738, -5.684)**	**-0.996 (-1.038, -0.954)**
$\omega_1 = \omega_2 = \log(2.0)$, $\delta = 5.0$, $\nu = 0.0$	β_1	**-5.045 (-5.062, -5.028)**	**-0.939 (-0.971, -0.907)**
$\omega_1 = \omega_2 = \log(2.0)$, $\delta = 5.0$, $\nu = 0.0$	β_2	**-5.416 (-5.434, -5.398)**	**-0.975 (-1.007, -0.942)**
$\omega_1 = \omega_2 = \log(2.0)$, $\delta = 5.0$, $\nu = 0.0$	β_3	**-5.602 (-5.623, -5.581)**	**-1.016 (-1.052, -0.981)**
$\omega_1 = \omega_2 = \log(2.0)$, $\delta = 5.0$, $\nu = 0.0$	β_4	**-5.684 (-5.709, -5.660)**	**-1.020 (-1.059, -0.981)**
$\omega_1 = \omega_2 = \log(2.0)$, $\delta = 5.0$, $\nu = 0.0$	β_5	**-5.750 (-5.778, -5.721)**	**-1.042 (-1.085, -0.999)**
$\omega_1 = \omega_2 = \log(2.0)$, $\delta = 25$, $\nu = -0.2$	β_1	**-5.036 (-5.053, -5.018)**	**-0.931 (-0.962, -0.899)**
$\omega_1 = \omega_2 = \log(2.0)$, $\delta = 25$, $\nu = -0.2$	β_2	**-5.405 (-5.423, -5.387)**	**-0.955 (-0.987, -0.922)**
$\omega_1 = \omega_2 = \log(2.0)$, $\delta = 25$, $\nu = -0.2$	β_3	**-5.571 (-5.591, -5.550)**	**-0.976 (-1.012, -0.940)**
$\omega_1 = \omega_2 = \log(2.0)$, $\delta = 25$, $\nu = -0.2$	β_4	**-5.651 (-5.674, -5.627)**	**-0.965 (-1.004, -0.926)**
$\omega_1 = \omega_2 = \log(2.0)$, $\delta = 25$, $\nu = -0.2$	β_5	**-5.726 (-5.754, -5.698)**	**-0.996 (-1.040, -0.951)**
$\omega_1 = \omega_2 = \log(2.0)$, $\delta = 25$, $\nu = 0.0$	β_1	**-5.044 (-5.061, -5.026)**	**-0.937 (-0.967, -0.906)**
$\omega_1 = \omega_2 = \log(2.0)$, $\delta = 25$, $\nu = 0.0$	β_2	**-5.417 (-5.436, -5.398)**	**-0.962 (-0.994, -0.930)**
$\omega_1 = \omega_2 = \log(2.0)$, $\delta = 25$, $\nu = 0.0$	β_3	**-5.576 (-5.597, -5.555)**	**-0.981 (-1.015, -0.946)**
$\omega_1 = \omega_2 = \log(2.0)$, $\delta = 25$, $\nu = 0.0$	β_4	**-5.686 (-5.711, -5.661)**	**-1.002 (-1.040, -0.965)**
$\omega_1 = \omega_2 = \log(2.0)$, $\delta = 25$, $\nu = 0.0$	β_5	**-5.738 (-5.767, -5.709)**	**-1.013 (-1.055, -0.970)**
**Additional scenarios:**			
$i = 3 \times 4$	β_1	**1.479 (1.445, 1.514)**	-0.004 (-0.046, 0.038)
$i = 3 \times 4$	β_2	**1.973 (1.936, 2.011)**	-0.007 (-0.054, 0.040)
$i = 3 \times 4$	β_3	**2.269 (2.228, 2.310)**	0.043 (-0.007, 0.093)
$i = 3 \times 4$	β_4	**2.410 (2.362, 2.458)**	0.006 (-0.050, 0.062)
$i = 3 \times 4$	β_5	**2.536 (2.481, 2.590)**	0.027 (-0.035, 0.090)
$i = 3 \times 4, N = 100$	β_1	**1.479 (1.451, 1.507)**	0.008 (-0.028, 0.044)
$i = 3 \times 4, N = 100$	β_2	**1.970 (1.940, 2.000)**	-0.004 (-0.045, 0.036)
$i = 3 \times 4, N = 100$	β_3	**2.241 (2.209, 2.273)**	0.015 (-0.028, 0.058)
$i = 3 \times 4, N = 100$	β_4	**2.388 (2.352, 2.423)**	-0.001 (-0.046, 0.045)
$i = 3 \times 4, N = 100$	β_5	**2.512 (2.474, 2.551)**	0.019 (-0.030, 0.068)

Table B4: Bias of period effects on the longitudinal outcome for the constant treatment effect parametrisation, with 95% confidence intervals based on Monte Carlo errors. LMM denotes the linear mixed model, while JM denotes the joint model. Statistically significant biases are highlighted in bold. *(continued)*

Scenario	Parameter	LMM	JM
$\sigma^2_\alpha = 1, \sigma^2_\phi = 27.5$	β_1	**0.700 (0.683, 0.716)**	-0.007 (-0.025, 0.012)
$\sigma^2_\alpha = 1, \sigma^2_\phi = 27.5$	β_2	**1.067 (1.048, 1.086)**	-0.007 (-0.029, 0.015)
$\sigma^2_\alpha = 1, \sigma^2_\phi = 27.5$	β_3	**1.293 (1.271, 1.314)**	-0.013 (-0.038, 0.013)
$\sigma^2_\alpha = 1, \sigma^2_\phi = 27.5$	β_4	**1.459 (1.434, 1.485)**	0.001 (-0.028, 0.029)
$\sigma^2_\alpha = 1, \sigma^2_\phi = 27.5$	β_5	**1.572 (1.543, 1.601)**	-0.008 (-0.040, 0.025)
$\sigma^2_\alpha = 4, \sigma^2_\phi = 110$	β_1	**3.032 (3.005, 3.059)**	0.009 (-0.026, 0.045)
$\sigma^2_\alpha = 4, \sigma^2_\phi = 110$	β_2	**3.585 (3.558, 3.612)**	0.007 (-0.030, 0.044)
$\sigma^2_\alpha = 4, \sigma^2_\phi = 110$	β_3	**3.821 (3.792, 3.850)**	-0.004 (-0.043, 0.036)
$\sigma^2_\alpha = 4, \sigma^2_\phi = 110$	β_4	**3.944 (3.911, 3.976)**	-0.023 (-0.065, 0.019)
$\sigma^2_\alpha = 4, \sigma^2_\phi = 110$	β_5	**4.053 (4.018, 4.087)**	-0.011 (-0.055, 0.033)

Table B5: Relative bias of period effects on the longitudinal outcome for the constant treatment effect parametrisation, with 95% confidence intervals based on Monte Carlo errors. LMM denotes the linear mixed model, while JM denotes the joint model. Statistically significant biases are highlighted in bold.

Scenario	Parameter	LMM	JM
**Main scenarios:**			
$\omega_1 = \omega_2 = \log(0.5)$, $\delta = 0.0$, $\nu = -0.2$	β_1	**0.169 (0.168, 0.169)**	**0.032 (0.031, 0.033)**
$\omega_1 = \omega_2 = \log(0.5)$, $\delta = 0.0$, $\nu = -0.2$	β_2	**0.181 (0.180, 0.182)**	**0.033 (0.032, 0.034)**
$\omega_1 = \omega_2 = \log(0.5)$, $\delta = 0.0$, $\nu = -0.2$	β_3	**0.186 (0.185, 0.187)**	**0.033 (0.032, 0.034)**
$\omega_1 = \omega_2 = \log(0.5)$, $\delta = 0.0$, $\nu = -0.2$	β_4	**0.189 (0.188, 0.190)**	**0.033 (0.032, 0.034)**
$\omega_1 = \omega_2 = \log(0.5)$, $\delta = 0.0$, $\nu = -0.2$	β_5	**0.191 (0.190, 0.192)**	**0.033 (0.032, 0.035)**
$\omega_1 = \omega_2 = \log(0.5)$, $\delta = 0.0$, $\nu = 0.0$	β_1	**0.168 (0.168, 0.169)**	**0.032 (0.031, 0.033)**
$\omega_1 = \omega_2 = \log(0.5)$, $\delta = 0.0$, $\nu = 0.0$	β_2	**0.181 (0.180, 0.182)**	**0.033 (0.032, 0.034)**
$\omega_1 = \omega_2 = \log(0.5)$, $\delta = 0.0$, $\nu = 0.0$	β_3	**0.186 (0.185, 0.187)**	**0.033 (0.032, 0.034)**
$\omega_1 = \omega_2 = \log(0.5)$, $\delta = 0.0$, $\nu = 0.0$	β_4	**0.190 (0.189, 0.191)**	**0.034 (0.033, 0.035)**
$\omega_1 = \omega_2 = \log(0.5)$, $\delta = 0.0$, $\nu = 0.0$	β_5	**0.192 (0.191, 0.193)**	**0.034 (0.032, 0.035)**
$\omega_1 = \omega_2 = \log(0.5)$, $\delta = 5.0$, $\nu = -0.2$	β_1	**0.168 (0.168, 0.169)**	**0.032 (0.031, 0.033)**
$\omega_1 = \omega_2 = \log(0.5)$, $\delta = 5.0$, $\nu = -0.2$	β_2	**0.180 (0.180, 0.181)**	**0.032 (0.031, 0.033)**
$\omega_1 = \omega_2 = \log(0.5)$, $\delta = 5.0$, $\nu = -0.2$	β_3	**0.186 (0.185, 0.186)**	**0.033 (0.032, 0.034)**
$\omega_1 = \omega_2 = \log(0.5)$, $\delta = 5.0$, $\nu = -0.2$	β_4	**0.189 (0.188, 0.190)**	**0.033 (0.031, 0.034)**
$\omega_1 = \omega_2 = \log(0.5)$, $\delta = 5.0$, $\nu = -0.2$	β_5	**0.191 (0.190, 0.192)**	**0.033 (0.032, 0.035)**
$\omega_1 = \omega_2 = \log(0.5)$, $\delta = 5.0$, $\nu = 0.0$	β_1	**0.168 (0.167, 0.168)**	**0.032 (0.031, 0.033)**
$\omega_1 = \omega_2 = \log(0.5)$, $\delta = 5.0$, $\nu = 0.0$	β_2	**0.180 (0.180, 0.181)**	**0.033 (0.032, 0.034)**

Table B5: Relative bias of period effects on the longitudinal outcome for the constant treatment effect parametrisation, with 95% confidence intervals based on Monte Carlo errors. LMM denotes the linear mixed model, while JM denotes the joint model. Statistically significant biases are highlighted in bold. *(continued)*

Scenario	Parameter	LMM	JM
$\omega_1 = \omega_2 = \log(0.5), \delta = 5.0, \nu = 0.0$	β_3	**0.186 (0.185, 0.186)**	**0.034 (0.032, 0.035)**
$\omega_1 = \omega_2 = \log(0.5), \delta = 5.0, \nu = 0.0$	β_4	**0.190 (0.189, 0.190)**	**0.034 (0.033, 0.035)**
$\omega_1 = \omega_2 = \log(0.5), \delta = 5.0, \nu = 0.0$	β_5	**0.191 (0.190, 0.192)**	**0.035 (0.033, 0.036)**
$\omega_1 = \omega_2 = \log(0.5), \delta = 25, \nu = -0.2$	β_1	**0.169 (0.168, 0.169)**	**0.032 (0.031, 0.033)**
$\omega_1 = \omega_2 = \log(0.5), \delta = 25, \nu = -0.2$	β_2	**0.181 (0.180, 0.182)**	**0.033 (0.032, 0.034)**
$\omega_1 = \omega_2 = \log(0.5), \delta = 25, \nu = -0.2$	β_3	**0.186 (0.186, 0.187)**	**0.033 (0.032, 0.034)**
$\omega_1 = \omega_2 = \log(0.5), \delta = 25, \nu = -0.2$	β_4	**0.189 (0.188, 0.190)**	**0.033 (0.032, 0.035)**
$\omega_1 = \omega_2 = \log(0.5), \delta = 25, \nu = -0.2$	β_5	**0.191 (0.191, 0.192)**	**0.033 (0.032, 0.035)**
$\omega_1 = \omega_2 = \log(0.5), \delta = 25, \nu = 0.0$	β_1	**0.169 (0.168, 0.169)**	**0.033 (0.032, 0.034)**
$\omega_1 = \omega_2 = \log(0.5), \delta = 25, \nu = 0.0$	β_2	**0.181 (0.180, 0.181)**	**0.033 (0.032, 0.034)**
$\omega_1 = \omega_2 = \log(0.5), \delta = 25, \nu = 0.0$	β_3	**0.187 (0.186, 0.187)**	**0.034 (0.033, 0.035)**
$\omega_1 = \omega_2 = \log(0.5), \delta = 25, \nu = 0.0$	β_4	**0.190 (0.189, 0.191)**	**0.034 (0.033, 0.036)**
$\omega_1 = \omega_2 = \log(0.5), \delta = 25, \nu = 0.0$	β_5	**0.192 (0.191, 0.193)**	**0.034 (0.033, 0.036)**
$\omega_1 = \omega_2 = \log(0.9), \delta = 0.0, \nu = -0.2$	β_1	**0.049 (0.048, 0.049)**	-0.001 (-0.002, 0.000)
$\omega_1 = \omega_2 = \log(0.9), \delta = 0.0, \nu = -0.2$	β_2	**0.065 (0.064, 0.066)**	-0.001 (-0.002, 0.000)
$\omega_1 = \omega_2 = \log(0.9), \delta = 0.0, \nu = -0.2$	β_3	**0.074 (0.073, 0.075)**	-0.001 (-0.002, 0.000)
$\omega_1 = \omega_2 = \log(0.9), \delta = 0.0, \nu = -0.2$	β_4	**0.079 (0.078, 0.080)**	-0.001 (-0.002, 0.000)
$\omega_1 = \omega_2 = \log(0.9), \delta = 0.0, \nu = -0.2$	β_5	**0.083 (0.082, 0.084)**	-0.001 (-0.002, 0.001)
$\omega_1 = \omega_2 = \log(0.9), \delta = 0.0, \nu = 0.0$	β_1	**0.049 (0.048, 0.050)**	-0.001 (-0.001, 0.000)
$\omega_1 = \omega_2 = \log(0.9), \delta = 0.0, \nu = 0.0$	β_2	**0.066 (0.065, 0.066)**	-0.001 (-0.002, 0.000)
$\omega_1 = \omega_2 = \log(0.9), \delta = 0.0, \nu = 0.0$	β_3	**0.075 (0.074, 0.076)**	-0.001 (-0.002, 0.000)
$\omega_1 = \omega_2 = \log(0.9), \delta = 0.0, \nu = 0.0$	β_4	**0.081 (0.080, 0.082)**	-0.000 (-0.001, 0.001)
$\omega_1 = \omega_2 = \log(0.9), \delta = 0.0, \nu = 0.0$	β_5	**0.085 (0.084, 0.086)**	-0.000 (-0.001, 0.001)
$\omega_1 = \omega_2 = \log(0.9), \delta = 5.0, \nu = -0.2$	β_1	**0.049 (0.048, 0.050)**	-0.000 (-0.001, 0.001)
$\omega_1 = \omega_2 = \log(0.9), \delta = 5.0, \nu = -0.2$	β_2	**0.065 (0.065, 0.066)**	-0.000 (-0.001, 0.000)
$\omega_1 = \omega_2 = \log(0.9), \delta = 5.0, \nu = -0.2$	β_3	**0.074 (0.073, 0.075)**	-0.000 (-0.001, 0.001)
$\omega_1 = \omega_2 = \log(0.9), \delta = 5.0, \nu = -0.2$	β_4	**0.079 (0.078, 0.080)**	-0.000 (-0.002, 0.001)
$\omega_1 = \omega_2 = \log(0.9), \delta = 5.0, \nu = -0.2$	β_5	**0.083 (0.082, 0.084)**	-0.000 (-0.002, 0.001)
$\omega_1 = \omega_2 = \log(0.9), \delta = 5.0, \nu = 0.0$	β_1	**0.049 (0.048, 0.049)**	-0.001 (-0.002, 0.000)
$\omega_1 = \omega_2 = \log(0.9), \delta = 5.0, \nu = 0.0$	β_2	**0.065 (0.065, 0.066)**	**-0.001 (-0.002, -0.000)**
$\omega_1 = \omega_2 = \log(0.9), \delta = 5.0, \nu = 0.0$	β_3	**0.075 (0.074, 0.075)**	-0.001 (-0.002, 0.000)
$\omega_1 = \omega_2 = \log(0.9), \delta = 5.0, \nu = 0.0$	β_4	**0.080 (0.079, 0.081)**	-0.001 (-0.002, 0.000)
$\omega_1 = \omega_2 = \log(0.9), \delta = 5.0, \nu = 0.0$	β_5	**0.084 (0.083, 0.085)**	-0.001 (-0.002, 0.001)
$\omega_1 = \omega_2 = \log(0.9), \delta = 25, \nu = -0.2$	β_1	**0.049 (0.049, 0.050)**	-0.000 (-0.001, 0.001)
$\omega_1 = \omega_2 = \log(0.9), \delta = 25, \nu = -0.2$	β_2	**0.066 (0.065, 0.066)**	-0.000 (-0.001, 0.000)
$\omega_1 = \omega_2 = \log(0.9), \delta = 25, \nu = -0.2$	β_3	**0.074 (0.073, 0.075)**	-0.000 (-0.001, 0.001)
$\omega_1 = \omega_2 = \log(0.9), \delta = 25, \nu = -0.2$	β_4	**0.079 (0.078, 0.080)**	-0.001 (-0.002, 0.000)

Table B5: Relative bias of period effects on the longitudinal outcome for the constant treatment effect parametrisation, with 95% confidence intervals based on Monte Carlo errors. LMM denotes the linear mixed model, while JM denotes the joint model. Statistically significant biases are highlighted in bold. *(continued)*

Scenario	Parameter	LMM	JM
$\omega_1 = \omega_2 = \log(0.9)$, $\delta = 25$, $\nu = -0.2$	β_5	**0.084 (0.083, 0.085)**	-0.000 (-0.001, 0.001)
$\omega_1 = \omega_2 = \log(0.9)$, $\delta = 25$, $\nu = 0.0$	β_1	**0.049 (0.049, 0.050)**	-0.000 (-0.001, 0.001)
$\omega_1 = \omega_2 = \log(0.9)$, $\delta = 25$, $\nu = 0.0$	β_2	**0.066 (0.065, 0.067)**	-0.001 (-0.002, 0.000)
$\omega_1 = \omega_2 = \log(0.9)$, $\delta = 25$, $\nu = 0.0$	β_3	**0.075 (0.074, 0.076)**	-0.000 (-0.001, 0.001)
$\omega_1 = \omega_2 = \log(0.9)$, $\delta = 25$, $\nu = 0.0$	β_4	**0.080 (0.079, 0.081)**	-0.001 (-0.002, 0.000)
$\omega_1 = \omega_2 = \log(0.9)$, $\delta = 25$, $\nu = 0.0$	β_5	**0.084 (0.083, 0.085)**	-0.001 (-0.002, 0.001)
$\omega_1 = \omega_2 = \log(1.0)$, $\delta = 0.0$, $\nu = -0.2$	β_1	**-0.001 (-0.001, -0.000)**	**-0.001 (-0.002, -0.000)**
$\omega_1 = \omega_2 = \log(1.0)$, $\delta = 0.0$, $\nu = -0.2$	β_2	-0.001 (-0.001, 0.000)	-0.001 (-0.001, 0.000)
$\omega_1 = \omega_2 = \log(1.0)$, $\delta = 0.0$, $\nu = -0.2$	β_3	-0.000 (-0.001, 0.000)	-0.001 (-0.001, 0.000)
$\omega_1 = \omega_2 = \log(1.0)$, $\delta = 0.0$, $\nu = -0.2$	β_4	-0.000 (-0.001, 0.001)	-0.000 (-0.001, 0.001)
$\omega_1 = \omega_2 = \log(1.0)$, $\delta = 0.0$, $\nu = -0.2$	β_5	-0.000 (-0.001, 0.001)	-0.000 (-0.001, 0.001)
$\omega_1 = \omega_2 = \log(1.0)$, $\delta = 0.0$, $\nu = 0.0$	β_1	0.000 (-0.001, 0.001)	0.000 (-0.001, 0.001)
$\omega_1 = \omega_2 = \log(1.0)$, $\delta = 0.0$, $\nu = 0.0$	β_2	0.000 (-0.000, 0.001)	0.001 (-0.000, 0.001)
$\omega_1 = \omega_2 = \log(1.0)$, $\delta = 0.0$, $\nu = 0.0$	β_3	0.000 (-0.001, 0.001)	0.000 (-0.000, 0.001)
$\omega_1 = \omega_2 = \log(1.0)$, $\delta = 0.0$, $\nu = 0.0$	β_4	0.000 (-0.001, 0.001)	0.000 (-0.001, 0.002)
$\omega_1 = \omega_2 = \log(1.0)$, $\delta = 0.0$, $\nu = 0.0$	β_5	0.000 (-0.001, 0.001)	0.001 (-0.001, 0.002)
$\omega_1 = \omega_2 = \log(1.0)$, $\delta = 5.0$, $\nu = -0.2$	β_1	-0.000 (-0.001, 0.001)	-0.000 (-0.001, 0.000)
$\omega_1 = \omega_2 = \log(1.0)$, $\delta = 5.0$, $\nu = -0.2$	β_2	-0.000 (-0.001, 0.000)	-0.001 (-0.001, 0.000)
$\omega_1 = \omega_2 = \log(1.0)$, $\delta = 5.0$, $\nu = -0.2$	β_3	-0.001 (-0.002, 0.000)	-0.001 (-0.002, 0.000)
$\omega_1 = \omega_2 = \log(1.0)$, $\delta = 5.0$, $\nu = -0.2$	β_4	0.000 (-0.001, 0.001)	-0.000 (-0.001, 0.001)
$\omega_1 = \omega_2 = \log(1.0)$, $\delta = 5.0$, $\nu = -0.2$	β_5	0.000 (-0.001, 0.001)	-0.000 (-0.001, 0.001)
$\omega_1 = \omega_2 = \log(1.0)$, $\delta = 5.0$, $\nu = 0.0$	β_1	-0.000 (-0.001, 0.001)	-0.000 (-0.001, 0.000)
$\omega_1 = \omega_2 = \log(1.0)$, $\delta = 5.0$, $\nu = 0.0$	β_2	-0.000 (-0.001, 0.001)	-0.000 (-0.001, 0.001)
$\omega_1 = \omega_2 = \log(1.0)$, $\delta = 5.0$, $\nu = 0.0$	β_3	-0.000 (-0.001, 0.001)	-0.000 (-0.001, 0.001)
$\omega_1 = \omega_2 = \log(1.0)$, $\delta = 5.0$, $\nu = 0.0$	β_4	-0.000 (-0.001, 0.001)	-0.000 (-0.001, 0.001)
$\omega_1 = \omega_2 = \log(1.0)$, $\delta = 5.0$, $\nu = 0.0$	β_5	-0.000 (-0.001, 0.001)	-0.000 (-0.001, 0.001)
$\omega_1 = \omega_2 = \log(1.0)$, $\delta = 25$, $\nu = -0.2$	β_1	-0.000 (-0.001, 0.001)	-0.000 (-0.001, 0.001)
$\omega_1 = \omega_2 = \log(1.0)$, $\delta = 25$, $\nu = -0.2$	β_2	-0.000 (-0.001, 0.001)	-0.000 (-0.001, 0.001)
$\omega_1 = \omega_2 = \log(1.0)$, $\delta = 25$, $\nu = -0.2$	β_3	-0.000 (-0.001, 0.001)	-0.000 (-0.001, 0.001)
$\omega_1 = \omega_2 = \log(1.0)$, $\delta = 25$, $\nu = -0.2$	β_4	0.000 (-0.001, 0.001)	0.000 (-0.001, 0.001)
$\omega_1 = \omega_2 = \log(1.0)$, $\delta = 25$, $\nu = -0.2$	β_5	-0.001 (-0.002, 0.001)	-0.001 (-0.002, 0.001)
$\omega_1 = \omega_2 = \log(1.0)$, $\delta = 25$, $\nu = 0.0$	β_1	-0.000 (-0.001, 0.001)	-0.000 (-0.001, 0.001)
$\omega_1 = \omega_2 = \log(1.0)$, $\delta = 25$, $\nu = 0.0$	β_2	0.000 (-0.001, 0.001)	0.000 (-0.000, 0.001)
$\omega_1 = \omega_2 = \log(1.0)$, $\delta = 25$, $\nu = 0.0$	β_3	-0.001 (-0.002, 0.000)	-0.000 (-0.001, 0.001)
$\omega_1 = \omega_2 = \log(1.0)$, $\delta = 25$, $\nu = 0.0$	β_4	-0.000 (-0.001, 0.001)	-0.000 (-0.001, 0.001)
$\omega_1 = \omega_2 = \log(1.0)$, $\delta = 25$, $\nu = 0.0$	β_5	-0.001 (-0.002, 0.000)	-0.000 (-0.002, 0.001)
$\omega_1 = \omega_2 = \log(2.0)$, $\delta = 0.0$, $\nu = -0.2$	β_1	**-0.168 (-0.169, -0.168)**	**-0.031 (-0.033, -0.030)**

Table B5: Relative bias of period effects on the longitudinal outcome for the constant treatment effect parametrisation, with 95% confidence intervals based on Monte Carlo errors. LMM denotes the linear mixed model, while JM denotes the joint model. Statistically significant biases are highlighted in bold. *(continued)*

Scenario	Parameter	LMM	JM
$\omega_1 = \omega_2 = \log(2.0)$, $\delta = 0.0$, $\nu = -0.2$	β_2	**-0.181 (-0.181, -0.180)**	**-0.033 (-0.034, -0.032)**
$\omega_1 = \omega_2 = \log(2.0)$, $\delta = 0.0$, $\nu = -0.2$	β_3	**-0.186 (-0.187, -0.185)**	**-0.033 (-0.034, -0.032)**
$\omega_1 = \omega_2 = \log(2.0)$, $\delta = 0.0$, $\nu = -0.2$	β_4	**-0.190 (-0.190, -0.189)**	**-0.034 (-0.035, -0.033)**
$\omega_1 = \omega_2 = \log(2.0)$, $\delta = 0.0$, $\nu = -0.2$	β_5	**-0.192 (-0.193, -0.191)**	**-0.034 (-0.035, -0.033)**
$\omega_1 = \omega_2 = \log(2.0)$, $\delta = 0.0$, $\nu = 0.0$	β_1	**-0.168 (-0.169, -0.167)**	**-0.030 (-0.031, -0.029)**
$\omega_1 = \omega_2 = \log(2.0)$, $\delta = 0.0$, $\nu = 0.0$	β_2	**-0.181 (-0.181, -0.180)**	**-0.031 (-0.033, -0.030)**
$\omega_1 = \omega_2 = \log(2.0)$, $\delta = 0.0$, $\nu = 0.0$	β_3	**-0.186 (-0.187, -0.185)**	**-0.032 (-0.033, -0.031)**
$\omega_1 = \omega_2 = \log(2.0)$, $\delta = 0.0$, $\nu = 0.0$	β_4	**-0.189 (-0.190, -0.188)**	**-0.032 (-0.033, -0.031)**
$\omega_1 = \omega_2 = \log(2.0)$, $\delta = 0.0$, $\nu = 0.0$	β_5	**-0.191 (-0.192, -0.190)**	**-0.032 (-0.034, -0.031)**
$\omega_1 = \omega_2 = \log(2.0)$, $\delta = 5.0$, $\nu = -0.2$	β_1	**-0.168 (-0.169, -0.167)**	**-0.032 (-0.033, -0.030)**
$\omega_1 = \omega_2 = \log(2.0)$, $\delta = 5.0$, $\nu = -0.2$	β_2	**-0.180 (-0.181, -0.180)**	**-0.033 (-0.034, -0.032)**
$\omega_1 = \omega_2 = \log(2.0)$, $\delta = 5.0$, $\nu = -0.2$	β_3	**-0.185 (-0.186, -0.185)**	**-0.033 (-0.034, -0.032)**
$\omega_1 = \omega_2 = \log(2.0)$, $\delta = 5.0$, $\nu = -0.2$	β_4	**-0.189 (-0.189, -0.188)**	**-0.033 (-0.034, -0.032)**
$\omega_1 = \omega_2 = \log(2.0)$, $\delta = 5.0$, $\nu = -0.2$	β_5	**-0.190 (-0.191, -0.189)**	**-0.033 (-0.035, -0.032)**
$\omega_1 = \omega_2 = \log(2.0)$, $\delta = 5.0$, $\nu = 0.0$	β_1	**-0.168 (-0.169, -0.168)**	**-0.031 (-0.032, -0.030)**
$\omega_1 = \omega_2 = \log(2.0)$, $\delta = 5.0$, $\nu = 0.0$	β_2	**-0.181 (-0.181, -0.180)**	**-0.032 (-0.034, -0.031)**
$\omega_1 = \omega_2 = \log(2.0)$, $\delta = 5.0$, $\nu = 0.0$	β_3	**-0.187 (-0.187, -0.186)**	**-0.034 (-0.035, -0.033)**
$\omega_1 = \omega_2 = \log(2.0)$, $\delta = 5.0$, $\nu = 0.0$	β_4	**-0.189 (-0.190, -0.189)**	**-0.034 (-0.035, -0.033)**
$\omega_1 = \omega_2 = \log(2.0)$, $\delta = 5.0$, $\nu = 0.0$	β_5	**-0.192 (-0.193, -0.191)**	**-0.035 (-0.036, -0.033)**
$\omega_1 = \omega_2 = \log(2.0)$, $\delta = 25$, $\nu = -0.2$	β_1	**-0.168 (-0.168, -0.167)**	**-0.031 (-0.032, -0.030)**
$\omega_1 = \omega_2 = \log(2.0)$, $\delta = 25$, $\nu = -0.2$	β_2	**-0.180 (-0.181, -0.180)**	**-0.032 (-0.033, -0.031)**
$\omega_1 = \omega_2 = \log(2.0)$, $\delta = 25$, $\nu = -0.2$	β_3	**-0.186 (-0.186, -0.185)**	**-0.033 (-0.034, -0.031)**
$\omega_1 = \omega_2 = \log(2.0)$, $\delta = 25$, $\nu = -0.2$	β_4	**-0.188 (-0.189, -0.188)**	**-0.032 (-0.033, -0.031)**
$\omega_1 = \omega_2 = \log(2.0)$, $\delta = 25$, $\nu = -0.2$	β_5	**-0.191 (-0.192, -0.190)**	**-0.033 (-0.035, -0.032)**
$\omega_1 = \omega_2 = \log(2.0)$, $\delta = 25$, $\nu = 0.0$	β_1	**-0.168 (-0.169, -0.168)**	**-0.031 (-0.032, -0.030)**
$\omega_1 = \omega_2 = \log(2.0)$, $\delta = 25$, $\nu = 0.0$	β_2	**-0.181 (-0.181, -0.180)**	**-0.032 (-0.033, -0.031)**
$\omega_1 = \omega_2 = \log(2.0)$, $\delta = 25$, $\nu = 0.0$	β_3	**-0.186 (-0.187, -0.185)**	**-0.033 (-0.034, -0.032)**
$\omega_1 = \omega_2 = \log(2.0)$, $\delta = 25$, $\nu = 0.0$	β_4	**-0.190 (-0.190, -0.189)**	**-0.033 (-0.035, -0.032)**
$\omega_1 = \omega_2 = \log(2.0)$, $\delta = 25$, $\nu = 0.0$	β_5	**-0.191 (-0.192, -0.190)**	**-0.034 (-0.035, -0.032)**
**Additional scenarios:**			
$i = 3 \times 4$	β_1	**0.049 (0.048, 0.050)**	-0.000 (-0.002, 0.001)
$i = 3 \times 4$	β_2	**0.066 (0.065, 0.067)**	-0.000 (-0.002, 0.001)
$i = 3 \times 4$	β_3	**0.076 (0.074, 0.077)**	0.001 (-0.000, 0.003)
$i = 3 \times 4$	β_4	**0.080 (0.079, 0.082)**	0.000 (-0.002, 0.002)
$i = 3 \times 4$	β_5	**0.085 (0.083, 0.086)**	0.001 (-0.001, 0.003)
$i = 3 \times 4, N = 100$	β_1	**0.049 (0.048, 0.050)**	0.000 (-0.001, 0.001)
$i = 3 \times 4, N = 100$	β_2	**0.066 (0.065, 0.067)**	-0.000 (-0.001, 0.001)

Table B5: Relative bias of period effects on the longitudinal outcome for the constant treatment effect parametrisation, with 95% confidence intervals based on Monte Carlo errors. LMM denotes the linear mixed model, while JM denotes the joint model. Statistically significant biases are highlighted in bold. *(continued)*

Scenario	Parameter	LMM	JM
$i = 3 \times 4, N = 100$	β_3	**0.075 (0.074, 0.076)**	0.001 (-0.001, 0.002)
$i = 3 \times 4, N = 100$	β_4	**0.080 (0.078, 0.081)**	-0.000 (-0.002, 0.002)
$i = 3 \times 4, N = 100$	β_5	**0.084 (0.082, 0.085)**	0.001 (-0.001, 0.002)
$\sigma^2_\alpha = 1, \sigma^2_\phi = 27.5$	β_1	**0.023 (0.023, 0.024)**	-0.000 (-0.001, 0.000)
$\sigma^2_\alpha = 1, \sigma^2_\phi = 27.5$	β_2	**0.036 (0.035, 0.036)**	-0.000 (-0.001, 0.000)
$\sigma^2_\alpha = 1, \sigma^2_\phi = 27.5$	β_3	**0.043 (0.042, 0.044)**	-0.000 (-0.001, 0.000)
$\sigma^2_\alpha = 1, \sigma^2_\phi = 27.5$	β_4	**0.049 (0.048, 0.049)**	0.000 (-0.001, 0.001)
$\sigma^2_\alpha = 1, \sigma^2_\phi = 27.5$	β_5	**0.052 (0.051, 0.053)**	-0.000 (-0.001, 0.001)
$\sigma^2_\alpha = 4, \sigma^2_\phi = 110$	β_1	**0.101 (0.100, 0.102)**	0.000 (-0.001, 0.002)
$\sigma^2_\alpha = 4, \sigma^2_\phi = 110$	β_2	**0.119 (0.119, 0.120)**	0.000 (-0.001, 0.001)
$\sigma^2_\alpha = 4, \sigma^2_\phi = 110$	β_3	**0.127 (0.126, 0.128)**	-0.000 (-0.001, 0.001)
$\sigma^2_\alpha = 4, \sigma^2_\phi = 110$	β_4	**0.131 (0.130, 0.133)**	-0.001 (-0.002, 0.001)
$\sigma^2_\alpha = 4, \sigma^2_\phi = 110$	β_5	**0.135 (0.134, 0.136)**	-0.000 (-0.002, 0.001)

Table B6: Coverage probability of period effects on the longitudinal outcome for the constant treatment effect parametrisation, with 95% confidence intervals based on Monte Carlo errors. LMM denotes the linear mixed model, while JM denotes the joint model.

Scenario	Parameter	LMM	JM
**Main scenarios:**			
$\omega_1 = \omega_2 = \log(0.5), \delta = 0.0, \nu = -0.2$	β_1	0.000 (0.000, 0.000)	0.538 (0.500, 0.575)
$\omega_1 = \omega_2 = \log(0.5), \delta = 0.0, \nu = -0.2$	β_2	0.000 (0.000, 0.000)	0.527 (0.490, 0.565)
$\omega_1 = \omega_2 = \log(0.5), \delta = 0.0, \nu = -0.2$	β_3	0.000 (0.000, 0.000)	0.542 (0.505, 0.579)
$\omega_1 = \omega_2 = \log(0.5), \delta = 0.0, \nu = -0.2$	β_4	0.000 (0.000, 0.000)	0.594 (0.557, 0.631)
$\omega_1 = \omega_2 = \log(0.5), \delta = 0.0, \nu = -0.2$	β_5	0.000 (0.000, 0.000)	0.659 (0.624, 0.694)
$\omega_1 = \omega_2 = \log(0.5), \delta = 0.0, \nu = 0.0$	β_1	0.000 (0.000, 0.000)	0.533 (0.495, 0.572)
$\omega_1 = \omega_2 = \log(0.5), \delta = 0.0, \nu = 0.0$	β_2	0.000 (0.000, 0.000)	0.523 (0.485, 0.561)
$\omega_1 = \omega_2 = \log(0.5), \delta = 0.0, \nu = 0.0$	β_3	0.000 (0.000, 0.000)	0.567 (0.529, 0.605)
$\omega_1 = \omega_2 = \log(0.5), \delta = 0.0, \nu = 0.0$	β_4	0.000 (0.000, 0.000)	0.600 (0.563, 0.638)
$\omega_1 = \omega_2 = \log(0.5), \delta = 0.0, \nu = 0.0$	β_5	0.000 (0.000, 0.000)	0.628 (0.591, 0.665)
$\omega_1 = \omega_2 = \log(0.5), \delta = 5.0, \nu = -0.2$	β_1	0.000 (0.000, 0.000)	0.506 (0.468, 0.545)
$\omega_1 = \omega_2 = \log(0.5), \delta = 5.0, \nu = -0.2$	β_2	0.000 (0.000, 0.000)	0.530 (0.491, 0.568)
$\omega_1 = \omega_2 = \log(0.5), \delta = 5.0, \nu = -0.2$	β_3	0.000 (0.000, 0.000)	0.562 (0.524, 0.601)
$\omega_1 = \omega_2 = \log(0.5), \delta = 5.0, \nu = -0.2$	β_4	0.000 (0.000, 0.000)	0.589 (0.551, 0.627)
$\omega_1 = \omega_2 = \log(0.5), \delta = 5.0, \nu = -0.2$	β_5	0.000 (0.000, 0.000)	0.628 (0.590, 0.665)

Table B6: Coverage probability of period effects on the longitudinal outcome for the constant treatment effect parametrisation, with 95% confidence intervals based on Monte Carlo errors. LMM denotes the linear mixed model, while JM denotes the joint model. *(continued)*

Scenario	Parameter	LMM	JM
$\omega_1 = \omega_2 = \log(0.5)$, $\delta = 5.0$, $\nu = 0.0$	β_1	0.000 (0.000, 0.000)	0.591 (0.553, 0.628)
$\omega_1 = \omega_2 = \log(0.5)$, $\delta = 5.0$, $\nu = 0.0$	β_2	0.000 (0.000, 0.000)	0.563 (0.526, 0.601)
$\omega_1 = \omega_2 = \log(0.5)$, $\delta = 5.0$, $\nu = 0.0$	β_3	0.000 (0.000, 0.000)	0.579 (0.541, 0.616)
$\omega_1 = \omega_2 = \log(0.5)$, $\delta = 5.0$, $\nu = 0.0$	β_4	0.000 (0.000, 0.000)	0.603 (0.565, 0.640)
$\omega_1 = \omega_2 = \log(0.5)$, $\delta = 5.0$, $\nu = 0.0$	β_5	0.000 (0.000, 0.000)	0.656 (0.619, 0.692)
$\omega_1 = \omega_2 = \log(0.5)$, $\delta = 25$, $\nu = -0.2$	β_1	0.000 (0.000, 0.000)	0.550 (0.511, 0.589)
$\omega_1 = \omega_2 = \log(0.5)$, $\delta = 25$, $\nu = -0.2$	β_2	0.000 (0.000, 0.000)	0.566 (0.527, 0.604)
$\omega_1 = \omega_2 = \log(0.5)$, $\delta = 25$, $\nu = -0.2$	β_3	0.000 (0.000, 0.000)	0.599 (0.561, 0.637)
$\omega_1 = \omega_2 = \log(0.5)$, $\delta = 25$, $\nu = -0.2$	β_4	0.000 (0.000, 0.000)	0.616 (0.579, 0.654)
$\omega_1 = \omega_2 = \log(0.5)$, $\delta = 25$, $\nu = -0.2$	β_5	0.000 (0.000, 0.000)	0.693 (0.657, 0.729)
$\omega_1 = \omega_2 = \log(0.5)$, $\delta = 25$, $\nu = 0.0$	β_1	0.000 (0.000, 0.000)	0.515 (0.478, 0.552)
$\omega_1 = \omega_2 = \log(0.5)$, $\delta = 25$, $\nu = 0.0$	β_2	0.000 (0.000, 0.000)	0.536 (0.499, 0.573)
$\omega_1 = \omega_2 = \log(0.5)$, $\delta = 25$, $\nu = 0.0$	β_3	0.000 (0.000, 0.000)	0.545 (0.508, 0.582)
$\omega_1 = \omega_2 = \log(0.5)$, $\delta = 25$, $\nu = 0.0$	β_4	0.000 (0.000, 0.000)	0.618 (0.582, 0.654)
$\omega_1 = \omega_2 = \log(0.5)$, $\delta = 25$, $\nu = 0.0$	β_5	0.000 (0.000, 0.000)	0.638 (0.602, 0.673)
$\omega_1 = \omega_2 = \log(0.9)$, $\delta = 0.0$, $\nu = -0.2$	β_1	0.010 (0.004, 0.015)	0.953 (0.940, 0.966)
$\omega_1 = \omega_2 = \log(0.9)$, $\delta = 0.0$, $\nu = -0.2$	β_2	0.001 (-0.001, 0.003)	0.943 (0.929, 0.957)
$\omega_1 = \omega_2 = \log(0.9)$, $\delta = 0.0$, $\nu = -0.2$	β_3	0.001 (-0.001, 0.003)	0.950 (0.936, 0.964)
$\omega_1 = \omega_2 = \log(0.9)$, $\delta = 0.0$, $\nu = -0.2$	β_4	0.003 (-0.000, 0.006)	0.954 (0.941, 0.967)
$\omega_1 = \omega_2 = \log(0.9)$, $\delta = 0.0$, $\nu = -0.2$	β_5	0.003 (-0.000, 0.006)	0.943 (0.929, 0.957)
$\omega_1 = \omega_2 = \log(0.9)$, $\delta = 0.0$, $\nu = 0.0$	β_1	0.007 (0.002, 0.012)	0.944 (0.930, 0.958)
$\omega_1 = \omega_2 = \log(0.9)$, $\delta = 0.0$, $\nu = 0.0$	β_2	0.000 (0.000, 0.000)	0.938 (0.923, 0.953)
$\omega_1 = \omega_2 = \log(0.9)$, $\delta = 0.0$, $\nu = 0.0$	β_3	0.000 (0.000, 0.000)	0.954 (0.941, 0.967)
$\omega_1 = \omega_2 = \log(0.9)$, $\delta = 0.0$, $\nu = 0.0$	β_4	0.002 (-0.001, 0.005)	0.949 (0.935, 0.963)
$\omega_1 = \omega_2 = \log(0.9)$, $\delta = 0.0$, $\nu = 0.0$	β_5	0.003 (-0.000, 0.006)	0.960 (0.948, 0.972)
$\omega_1 = \omega_2 = \log(0.9)$, $\delta = 5.0$, $\nu = -0.2$	β_1	0.015 (0.008, 0.023)	0.936 (0.920, 0.951)
$\omega_1 = \omega_2 = \log(0.9)$, $\delta = 5.0$, $\nu = -0.2$	β_2	0.001 (-0.001, 0.003)	0.943 (0.928, 0.957)
$\omega_1 = \omega_2 = \log(0.9)$, $\delta = 5.0$, $\nu = -0.2$	β_3	0.000 (0.000, 0.000)	0.943 (0.928, 0.957)
$\omega_1 = \omega_2 = \log(0.9)$, $\delta = 5.0$, $\nu = -0.2$	β_4	0.000 (0.000, 0.000)	0.953 (0.940, 0.966)
$\omega_1 = \omega_2 = \log(0.9)$, $\delta = 5.0$, $\nu = -0.2$	β_5	0.004 (0.000, 0.008)	0.952 (0.938, 0.965)
$\omega_1 = \omega_2 = \log(0.9)$, $\delta = 5.0$, $\nu = 0.0$	β_1	0.014 (0.007, 0.022)	0.939 (0.925, 0.954)
$\omega_1 = \omega_2 = \log(0.9)$, $\delta = 5.0$, $\nu = 0.0$	β_2	0.002 (-0.001, 0.005)	0.940 (0.926, 0.955)
$\omega_1 = \omega_2 = \log(0.9)$, $\delta = 5.0$, $\nu = 0.0$	β_3	0.000 (0.000, 0.000)	0.948 (0.935, 0.962)
$\omega_1 = \omega_2 = \log(0.9)$, $\delta = 5.0$, $\nu = 0.0$	β_4	0.000 (0.000, 0.000)	0.961 (0.949, 0.973)
$\omega_1 = \omega_2 = \log(0.9)$, $\delta = 5.0$, $\nu = 0.0$	β_5	0.004 (0.000, 0.008)	0.943 (0.929, 0.958)
$\omega_1 = \omega_2 = \log(0.9)$, $\delta = 25$, $\nu = -0.2$	β_1	0.012 (0.006, 0.019)	0.941 (0.926, 0.956)
$\omega_1 = \omega_2 = \log(0.9)$, $\delta = 25$, $\nu = -0.2$	β_2	0.003 (-0.000, 0.006)	0.947 (0.933, 0.961)
$\omega_1 = \omega_2 = \log(0.9)$, $\delta = 25$, $\nu = -0.2$	β_3	0.001 (-0.001, 0.003)	0.945 (0.931, 0.959)

Table B6: Coverage probability of period effects on the longitudinal outcome for the constant treatment effect parametrisation, with 95% confidence intervals based on Monte Carlo errors. LMM denotes the linear mixed model, while JM denotes the joint model. *(continued)*

Scenario	Parameter	LMM	JM
$\omega_1 = \omega_2 = \log(0.9)$, $\delta = 25$, $\nu = -0.2$	β_4	0.002 (-0.001, 0.005)	0.950 (0.936, 0.963)
$\omega_1 = \omega_2 = \log(0.9)$, $\delta = 25$, $\nu = -0.2$	β_5	0.003 (-0.000, 0.006)	0.960 (0.948, 0.972)
$\omega_1 = \omega_2 = \log(0.9)$, $\delta = 25$, $\nu = 0.0$	β_1	0.010 (0.004, 0.015)	0.930 (0.914, 0.945)
$\omega_1 = \omega_2 = \log(0.9)$, $\delta = 25$, $\nu = 0.0$	β_2	0.000 (0.000, 0.000)	0.931 (0.915, 0.946)
$\omega_1 = \omega_2 = \log(0.9)$, $\delta = 25$, $\nu = 0.0$	β_3	0.000 (0.000, 0.000)	0.941 (0.927, 0.956)
$\omega_1 = \omega_2 = \log(0.9)$, $\delta = 25$, $\nu = 0.0$	β_4	0.000 (0.000, 0.000)	0.950 (0.937, 0.964)
$\omega_1 = \omega_2 = \log(0.9)$, $\delta = 25$, $\nu = 0.0$	β_5	0.006 (0.001, 0.010)	0.947 (0.934, 0.961)
$\omega_1 = \omega_2 = \log(1.0)$, $\delta = 0.0$, $\nu = -0.2$	β_1	0.938 (0.923, 0.953)	0.945 (0.931, 0.959)
$\omega_1 = \omega_2 = \log(1.0)$, $\delta = 0.0$, $\nu = -0.2$	β_2	0.951 (0.938, 0.964)	0.957 (0.945, 0.969)
$\omega_1 = \omega_2 = \log(1.0)$, $\delta = 0.0$, $\nu = -0.2$	β_3	0.963 (0.951, 0.974)	0.958 (0.946, 0.970)
$\omega_1 = \omega_2 = \log(1.0)$, $\delta = 0.0$, $\nu = -0.2$	β_4	0.959 (0.947, 0.971)	0.955 (0.942, 0.968)
$\omega_1 = \omega_2 = \log(1.0)$, $\delta = 0.0$, $\nu = -0.2$	β_5	0.955 (0.943, 0.968)	0.964 (0.952, 0.975)
$\omega_1 = \omega_2 = \log(1.0)$, $\delta = 0.0$, $\nu = 0.0$	β_1	0.939 (0.924, 0.953)	0.942 (0.928, 0.957)
$\omega_1 = \omega_2 = \log(1.0)$, $\delta = 0.0$, $\nu = 0.0$	β_2	0.942 (0.928, 0.956)	0.942 (0.928, 0.957)
$\omega_1 = \omega_2 = \log(1.0)$, $\delta = 0.0$, $\nu = 0.0$	β_3	0.946 (0.932, 0.959)	0.947 (0.934, 0.961)
$\omega_1 = \omega_2 = \log(1.0)$, $\delta = 0.0$, $\nu = 0.0$	β_4	0.950 (0.937, 0.963)	0.953 (0.940, 0.966)
$\omega_1 = \omega_2 = \log(1.0)$, $\delta = 0.0$, $\nu = 0.0$	β_5	0.956 (0.944, 0.968)	0.954 (0.941, 0.967)
$\omega_1 = \omega_2 = \log(1.0)$, $\delta = 5.0$, $\nu = -0.2$	β_1	0.946 (0.932, 0.959)	0.945 (0.932, 0.959)
$\omega_1 = \omega_2 = \log(1.0)$, $\delta = 5.0$, $\nu = -0.2$	β_2	0.954 (0.941, 0.967)	0.940 (0.925, 0.954)
$\omega_1 = \omega_2 = \log(1.0)$, $\delta = 5.0$, $\nu = -0.2$	β_3	0.943 (0.929, 0.957)	0.945 (0.932, 0.959)
$\omega_1 = \omega_2 = \log(1.0)$, $\delta = 5.0$, $\nu = -0.2$	β_4	0.952 (0.939, 0.965)	0.949 (0.936, 0.963)
$\omega_1 = \omega_2 = \log(1.0)$, $\delta = 5.0$, $\nu = -0.2$	β_5	0.962 (0.950, 0.973)	0.944 (0.930, 0.958)
$\omega_1 = \omega_2 = \log(1.0)$, $\delta = 5.0$, $\nu = 0.0$	β_1	0.930 (0.914, 0.945)	0.941 (0.926, 0.955)
$\omega_1 = \omega_2 = \log(1.0)$, $\delta = 5.0$, $\nu = 0.0$	β_2	0.945 (0.931, 0.959)	0.937 (0.922, 0.952)
$\omega_1 = \omega_2 = \log(1.0)$, $\delta = 5.0$, $\nu = 0.0$	β_3	0.950 (0.937, 0.964)	0.962 (0.951, 0.974)
$\omega_1 = \omega_2 = \log(1.0)$, $\delta = 5.0$, $\nu = 0.0$	β_4	0.953 (0.941, 0.966)	0.946 (0.932, 0.960)
$\omega_1 = \omega_2 = \log(1.0)$, $\delta = 5.0$, $\nu = 0.0$	β_5	0.944 (0.930, 0.958)	0.944 (0.930, 0.958)
$\omega_1 = \omega_2 = \log(1.0)$, $\delta = 25$, $\nu = -0.2$	β_1	0.942 (0.927, 0.956)	0.946 (0.932, 0.960)
$\omega_1 = \omega_2 = \log(1.0)$, $\delta = 25$, $\nu = -0.2$	β_2	0.945 (0.932, 0.959)	0.951 (0.938, 0.964)
$\omega_1 = \omega_2 = \log(1.0)$, $\delta = 25$, $\nu = -0.2$	β_3	0.944 (0.931, 0.958)	0.939 (0.924, 0.954)
$\omega_1 = \omega_2 = \log(1.0)$, $\delta = 25$, $\nu = -0.2$	β_4	0.946 (0.933, 0.960)	0.947 (0.933, 0.961)
$\omega_1 = \omega_2 = \log(1.0)$, $\delta = 25$, $\nu = -0.2$	β_5	0.948 (0.935, 0.962)	0.958 (0.945, 0.970)
$\omega_1 = \omega_2 = \log(1.0)$, $\delta = 25$, $\nu = 0.0$	β_1	0.942 (0.928, 0.956)	0.946 (0.932, 0.960)
$\omega_1 = \omega_2 = \log(1.0)$, $\delta = 25$, $\nu = 0.0$	β_2	0.946 (0.932, 0.959)	0.938 (0.924, 0.953)
$\omega_1 = \omega_2 = \log(1.0)$, $\delta = 25$, $\nu = 0.0$	β_3	0.955 (0.943, 0.968)	0.947 (0.934, 0.961)
$\omega_1 = \omega_2 = \log(1.0)$, $\delta = 25$, $\nu = 0.0$	β_4	0.962 (0.950, 0.973)	0.951 (0.938, 0.964)
$\omega_1 = \omega_2 = \log(1.0)$, $\delta = 25$, $\nu = 0.0$	β_5	0.955 (0.943, 0.968)	0.949 (0.936, 0.963)
$\omega_1 = \omega_2 = \log(2.0)$, $\delta = 0.0$, $\nu = -0.2$	β_1	0.000 (0.000, 0.000)	0.569 (0.529, 0.608)

Table B6: Coverage probability of period effects on the longitudinal outcome for the constant treatment effect parametrisation, with 95% confidence intervals based on Monte Carlo errors. LMM denotes the linear mixed model, while JM denotes the joint model. *(continued)*

Scenario	Parameter	LMM	JM
$\omega_1 = \omega_2 = \log(2.0)$, $\delta = 0.0$, $\nu = -0.2$	β_2	0.000 (0.000, 0.000)	0.517 (0.477, 0.557)
$\omega_1 = \omega_2 = \log(2.0)$, $\delta = 0.0$, $\nu = -0.2$	β_3	0.000 (0.000, 0.000)	0.567 (0.527, 0.607)
$\omega_1 = \omega_2 = \log(2.0)$, $\delta = 0.0$, $\nu = -0.2$	β_4	0.000 (0.000, 0.000)	0.590 (0.551, 0.630)
$\omega_1 = \omega_2 = \log(2.0)$, $\delta = 0.0$, $\nu = -0.2$	β_5	0.000 (0.000, 0.000)	0.630 (0.592, 0.669)
$\omega_1 = \omega_2 = \log(2.0)$, $\delta = 0.0$, $\nu = 0.0$	β_1	0.000 (0.000, 0.000)	0.548 (0.508, 0.588)
$\omega_1 = \omega_2 = \log(2.0)$, $\delta = 0.0$, $\nu = 0.0$	β_2	0.000 (0.000, 0.000)	0.550 (0.510, 0.590)
$\omega_1 = \omega_2 = \log(2.0)$, $\delta = 0.0$, $\nu = 0.0$	β_3	0.000 (0.000, 0.000)	0.577 (0.537, 0.616)
$\omega_1 = \omega_2 = \log(2.0)$, $\delta = 0.0$, $\nu = 0.0$	β_4	0.000 (0.000, 0.000)	0.595 (0.556, 0.635)
$\omega_1 = \omega_2 = \log(2.0)$, $\delta = 0.0$, $\nu = 0.0$	β_5	0.000 (0.000, 0.000)	0.669 (0.632, 0.707)
$\omega_1 = \omega_2 = \log(2.0)$, $\delta = 5.0$, $\nu = -0.2$	β_1	0.000 (0.000, 0.000)	0.546 (0.507, 0.585)
$\omega_1 = \omega_2 = \log(2.0)$, $\delta = 5.0$, $\nu = -0.2$	β_2	0.000 (0.000, 0.000)	0.516 (0.477, 0.555)
$\omega_1 = \omega_2 = \log(2.0)$, $\delta = 5.0$, $\nu = -0.2$	β_3	0.000 (0.000, 0.000)	0.553 (0.514, 0.592)
$\omega_1 = \omega_2 = \log(2.0)$, $\delta = 5.0$, $\nu = -0.2$	β_4	0.000 (0.000, 0.000)	0.604 (0.566, 0.642)
$\omega_1 = \omega_2 = \log(2.0)$, $\delta = 5.0$, $\nu = -0.2$	β_5	0.000 (0.000, 0.000)	0.660 (0.623, 0.697)
$\omega_1 = \omega_2 = \log(2.0)$, $\delta = 5.0$, $\nu = 0.0$	β_1	0.000 (0.000, 0.000)	0.522 (0.483, 0.562)
$\omega_1 = \omega_2 = \log(2.0)$, $\delta = 5.0$, $\nu = 0.0$	β_2	0.000 (0.000, 0.000)	0.501 (0.461, 0.541)
$\omega_1 = \omega_2 = \log(2.0)$, $\delta = 5.0$, $\nu = 0.0$	β_3	0.000 (0.000, 0.000)	0.521 (0.481, 0.561)
$\omega_1 = \omega_2 = \log(2.0)$, $\delta = 5.0$, $\nu = 0.0$	β_4	0.000 (0.000, 0.000)	0.572 (0.533, 0.612)
$\omega_1 = \omega_2 = \log(2.0)$, $\delta = 5.0$, $\nu = 0.0$	β_5	0.000 (0.000, 0.000)	0.625 (0.587, 0.664)
$\omega_1 = \omega_2 = \log(2.0)$, $\delta = 25$, $\nu = -0.2$	β_1	0.000 (0.000, 0.000)	0.569 (0.530, 0.608)
$\omega_1 = \omega_2 = \log(2.0)$, $\delta = 25$, $\nu = -0.2$	β_2	0.000 (0.000, 0.000)	0.551 (0.511, 0.591)
$\omega_1 = \omega_2 = \log(2.0)$, $\delta = 25$, $\nu = -0.2$	β_3	0.000 (0.000, 0.000)	0.564 (0.525, 0.604)
$\omega_1 = \omega_2 = \log(2.0)$, $\delta = 25$, $\nu = -0.2$	β_4	0.000 (0.000, 0.000)	0.630 (0.592, 0.668)
$\omega_1 = \omega_2 = \log(2.0)$, $\delta = 25$, $\nu = -0.2$	β_5	0.000 (0.000, 0.000)	0.658 (0.620, 0.696)
$\omega_1 = \omega_2 = \log(2.0)$, $\delta = 25$, $\nu = 0.0$	β_1	0.000 (0.000, 0.000)	0.531 (0.491, 0.571)
$\omega_1 = \omega_2 = \log(2.0)$, $\delta = 25$, $\nu = 0.0$	β_2	0.000 (0.000, 0.000)	0.551 (0.511, 0.591)
$\omega_1 = \omega_2 = \log(2.0)$, $\delta = 25$, $\nu = 0.0$	β_3	0.000 (0.000, 0.000)	0.581 (0.541, 0.620)
$\omega_1 = \omega_2 = \log(2.0)$, $\delta = 25$, $\nu = 0.0$	β_4	0.000 (0.000, 0.000)	0.609 (0.570, 0.648)
$\omega_1 = \omega_2 = \log(2.0)$, $\delta = 25$, $\nu = 0.0$	β_5	0.000 (0.000, 0.000)	0.662 (0.624, 0.700)
**Additional scenarios:**			
$i = 3 \times 4$	β_1	0.216 (0.191, 0.241)	0.942 (0.927, 0.957)
$i = 3 \times 4$	β_2	0.091 (0.074, 0.109)	0.941 (0.926, 0.957)
$i = 3 \times 4$	β_3	0.077 (0.060, 0.093)	0.937 (0.921, 0.953)
$i = 3 \times 4$	β_4	0.114 (0.094, 0.133)	0.941 (0.926, 0.957)
$i = 3 \times 4$	β_5	0.181 (0.157, 0.204)	0.937 (0.921, 0.953)
$i = 3 \times 4, N = 100$	β_1	0.100 (0.082, 0.119)	0.920 (0.903, 0.938)
$i = 3 \times 4, N = 100$	β_2	0.024 (0.015, 0.033)	0.917 (0.899, 0.935)
$i = 3 \times 4, N = 100$	β_3	0.018 (0.010, 0.026)	0.936 (0.920, 0.952)

Table B6: Coverage probability of period effects on the longitudinal outcome for the constant treatment effect parametrisation, with 95% confidence intervals based on Monte Carlo errors. LMM denotes the linear mixed model, while JM denotes the joint model. *(continued)*

Scenario	Parameter	LMM	JM
$i = 3 \times 4, N = 100$	β_4	0.018 (0.010, 0.026)	0.931 (0.915, 0.948)
$i = 3 \times 4, N = 100$	β_5	0.030 (0.019, 0.040)	0.940 (0.925, 0.955)
$\sigma_\alpha^2 = 1, \sigma_\phi^2 = 27.5$	β_1	0.281 (0.254, 0.309)	0.943 (0.929, 0.957)
$\sigma_\alpha^2 = 1, \sigma_\phi^2 = 27.5$	β_2	0.068 (0.053, 0.083)	0.940 (0.925, 0.955)
$\sigma_\alpha^2 = 1, \sigma_\phi^2 = 27.5$	β_3	0.048 (0.035, 0.061)	0.945 (0.931, 0.959)
$\sigma_\alpha^2 = 1, \sigma_\phi^2 = 27.5$	β_4	0.059 (0.045, 0.074)	0.948 (0.934, 0.961)
$\sigma_\alpha^2 = 1, \sigma_\phi^2 = 27.5$	β_5	0.100 (0.082, 0.119)	0.946 (0.932, 0.960)
$\sigma_\alpha^2 = 4, \sigma_\phi^2 = 110$	β_1	0.000 (0.000, 0.000)	0.942 (0.927, 0.957)
$\sigma_\alpha^2 = 4, \sigma_\phi^2 = 110$	β_2	0.000 (0.000, 0.000)	0.946 (0.932, 0.961)
$\sigma_\alpha^2 = 4, \sigma_\phi^2 = 110$	β_3	0.000 (0.000, 0.000)	0.945 (0.931, 0.960)
$\sigma_\alpha^2 = 4, \sigma_\phi^2 = 110$	β_4	0.000 (0.000, 0.000)	0.950 (0.936, 0.964)
$\sigma_\alpha^2 = 4, \sigma_\phi^2 = 110$	β_5	0.000 (0.000, 0.000)	0.962 (0.949, 0.974)

Table B7: Bias of ICCs for the constant treatment effect parametrisation, with 95% confidence intervals based on Monte Carlo errors. LMM denotes the linear mixed model, while JM denotes the joint model. Statistically significant biases are highlighted in bold.

Scenario	Parameter	LMM	JM
**Main scenarios:**			
$\omega_1 = \omega_2 = \log(0.5), \delta = 0.0, \nu = -0.2$	ρ_a	**-0.225 (-0.226, -0.224)**	**-0.026 (-0.028, -0.025)**
$\omega_1 = \omega_2 = \log(0.5), \delta = 0.0, \nu = -0.2$	ρ_d	**-0.015 (-0.016, -0.015)**	**-0.008 (-0.008, -0.007)**
$\omega_1 = \omega_2 = \log(0.5), \delta = 0.0, \nu = 0.0$	ρ_a	**-0.226 (-0.227, -0.225)**	**-0.026 (-0.028, -0.025)**
$\omega_1 = \omega_2 = \log(0.5), \delta = 0.0, \nu = 0.0$	ρ_d	**-0.015 (-0.016, -0.015)**	**-0.008 (-0.008, -0.007)**
$\omega_1 = \omega_2 = \log(0.5), \delta = 5.0, \nu = -0.2$	ρ_a	**-0.225 (-0.226, -0.224)**	**-0.026 (-0.028, -0.025)**
$\omega_1 = \omega_2 = \log(0.5), \delta = 5.0, \nu = -0.2$	ρ_d	**-0.015 (-0.015, -0.015)**	**-0.007 (-0.008, -0.007)**
$\omega_1 = \omega_2 = \log(0.5), \delta = 5.0, \nu = 0.0$	ρ_a	**-0.226 (-0.227, -0.225)**	**-0.026 (-0.028, -0.025)**
$\omega_1 = \omega_2 = \log(0.5), \delta = 5.0, \nu = 0.0$	ρ_d	**-0.015 (-0.015, -0.015)**	**-0.007 (-0.008, -0.007)**
$\omega_1 = \omega_2 = \log(0.5), \delta = 25, \nu = -0.2$	ρ_a	**-0.225 (-0.227, -0.224)**	**-0.026 (-0.028, -0.025)**
$\omega_1 = \omega_2 = \log(0.5), \delta = 25, \nu = -0.2$	ρ_d	**-0.015 (-0.015, -0.015)**	**-0.007 (-0.008, -0.007)**
$\omega_1 = \omega_2 = \log(0.5), \delta = 25, \nu = 0.0$	ρ_a	**-0.227 (-0.228, -0.226)**	**-0.027 (-0.029, -0.026)**
$\omega_1 = \omega_2 = \log(0.5), \delta = 25, \nu = 0.0$	ρ_d	**-0.015 (-0.016, -0.015)**	**-0.007 (-0.008, -0.007)**
$\omega_1 = \omega_2 = \log(0.9), \delta = 0.0, \nu = -0.2$	ρ_a	**-0.053 (-0.054, -0.052)**	-0.000 (-0.001, 0.001)
$\omega_1 = \omega_2 = \log(0.9), \delta = 0.0, \nu = -0.2$	ρ_d	**-0.005 (-0.006, -0.005)**	**-0.001 (-0.001, -0.000)**
$\omega_1 = \omega_2 = \log(0.9), \delta = 0.0, \nu = 0.0$	ρ_a	**-0.055 (-0.056, -0.054)**	-0.000 (-0.001, 0.001)
$\omega_1 = \omega_2 = \log(0.9), \delta = 0.0, \nu = 0.0$	ρ_d	**-0.006 (-0.006, -0.005)**	**-0.001 (-0.002, -0.001)**
$\omega_1 = \omega_2 = \log(0.9), \delta = 5.0, \nu = -0.2$	ρ_a	**-0.053 (-0.054, -0.053)**	-0.000 (-0.001, 0.001)

Table B7: Bias of ICCs for the constant treatment effect parametrisation, with 95% confidence intervals based on Monte Carlo errors. LMM denotes the linear mixed model, while JM denotes the joint model. Statistically significant biases are highlighted in bold. *(continued)*

Scenario	Parameter	LMM	JM
$\omega_1 = \omega_2 = \log(0.9), \delta = 5.0, \nu = -0.2$	ρ_d	**-0.005 (-0.006, -0.005)**	**-0.001 (-0.001, -0.000)**
$\omega_1 = \omega_2 = \log(0.9), \delta = 5.0, \nu = 0.0$	ρ_a	**-0.054 (-0.055, -0.053)**	-0.000 (-0.002, 0.001)
$\omega_1 = \omega_2 = \log(0.9), \delta = 5.0, \nu = 0.0$	ρ_d	**-0.005 (-0.006, -0.005)**	-0.000 (-0.001, 0.000)
$\omega_1 = \omega_2 = \log(0.9), \delta = 25, \nu = -0.2$	ρ_a	**-0.053 (-0.054, -0.052)**	0.000 (-0.001, 0.001)
$\omega_1 = \omega_2 = \log(0.9), \delta = 25, \nu = -0.2$	ρ_d	**-0.005 (-0.006, -0.005)**	-0.000 (-0.001, 0.000)
$\omega_1 = \omega_2 = \log(0.9), \delta = 25, \nu = 0.0$	ρ_a	**-0.054 (-0.055, -0.053)**	-0.000 (-0.001, 0.001)
$\omega_1 = \omega_2 = \log(0.9), \delta = 25, \nu = 0.0$	ρ_d	**-0.005 (-0.006, -0.005)**	**-0.001 (-0.001, -0.000)**
$\omega_1 = \omega_2 = \log(1.0), \delta = 0.0, \nu = -0.2$	ρ_a	0.001 (-0.000, 0.002)	**0.001 (0.000, 0.002)**
$\omega_1 = \omega_2 = \log(1.0), \delta = 0.0, \nu = -0.2$	ρ_d	**-0.001 (-0.001, -0.000)**	-0.000 (-0.001, 0.000)
$\omega_1 = \omega_2 = \log(1.0), \delta = 0.0, \nu = 0.0$	ρ_a	0.000 (-0.000, 0.001)	0.001 (-0.000, 0.002)
$\omega_1 = \omega_2 = \log(1.0), \delta = 0.0, \nu = 0.0$	ρ_d	**-0.002 (-0.002, -0.001)**	**-0.001 (-0.002, -0.001)**
$\omega_1 = \omega_2 = \log(1.0), \delta = 5.0, \nu = -0.2$	ρ_a	0.000 (-0.001, 0.001)	0.000 (-0.000, 0.001)
$\omega_1 = \omega_2 = \log(1.0), \delta = 5.0, \nu = -0.2$	ρ_d	**-0.001 (-0.002, -0.000)**	**-0.001 (-0.001, -0.000)**
$\omega_1 = \omega_2 = \log(1.0), \delta = 5.0, \nu = 0.0$	ρ_a	0.000 (-0.001, 0.001)	0.000 (-0.001, 0.001)
$\omega_1 = \omega_2 = \log(1.0), \delta = 5.0, \nu = 0.0$	ρ_d	**-0.001 (-0.001, -0.000)**	**-0.001 (-0.001, -0.000)**
$\omega_1 = \omega_2 = \log(1.0), \delta = 25, \nu = -0.2$	ρ_a	-0.000 (-0.001, 0.001)	0.000 (-0.001, 0.001)
$\omega_1 = \omega_2 = \log(1.0), \delta = 25, \nu = -0.2$	ρ_d	**-0.001 (-0.002, -0.001)**	**-0.001 (-0.002, -0.000)**
$\omega_1 = \omega_2 = \log(1.0), \delta = 25, \nu = 0.0$	ρ_a	-0.000 (-0.001, 0.000)	-0.000 (-0.001, 0.001)
$\omega_1 = \omega_2 = \log(1.0), \delta = 25, \nu = 0.0$	ρ_d	**-0.001 (-0.002, -0.001)**	**-0.001 (-0.002, -0.000)**
$\omega_1 = \omega_2 = \log(2.0), \delta = 0.0, \nu = -0.2$	ρ_a	**-0.225 (-0.226, -0.224)**	**-0.027 (-0.028, -0.025)**
$\omega_1 = \omega_2 = \log(2.0), \delta = 0.0, \nu = -0.2$	ρ_d	**-0.015 (-0.015, -0.015)**	**-0.008 (-0.008, -0.007)**
$\omega_1 = \omega_2 = \log(2.0), \delta = 0.0, \nu = 0.0$	ρ_a	**-0.226 (-0.227, -0.225)**	**-0.025 (-0.026, -0.023)**
$\omega_1 = \omega_2 = \log(2.0), \delta = 0.0, \nu = 0.0$	ρ_d	**-0.015 (-0.015, -0.015)**	**-0.008 (-0.008, -0.007)**
$\omega_1 = \omega_2 = \log(2.0), \delta = 5.0, \nu = -0.2$	ρ_a	**-0.225 (-0.226, -0.224)**	**-0.027 (-0.028, -0.025)**
$\omega_1 = \omega_2 = \log(2.0), \delta = 5.0, \nu = -0.2$	ρ_d	**-0.015 (-0.016, -0.015)**	**-0.008 (-0.008, -0.007)**
$\omega_1 = \omega_2 = \log(2.0), \delta = 5.0, \nu = 0.0$	ρ_a	**-0.226 (-0.227, -0.225)**	**-0.026 (-0.027, -0.024)**
$\omega_1 = \omega_2 = \log(2.0), \delta = 5.0, \nu = 0.0$	ρ_d	**-0.015 (-0.015, -0.015)**	**-0.008 (-0.008, -0.007)**
$\omega_1 = \omega_2 = \log(2.0), \delta = 25, \nu = -0.2$	ρ_a	**-0.225 (-0.226, -0.224)**	**-0.026 (-0.028, -0.025)**
$\omega_1 = \omega_2 = \log(2.0), \delta = 25, \nu = -0.2$	ρ_d	**-0.015 (-0.015, -0.015)**	**-0.007 (-0.008, -0.007)**
$\omega_1 = \omega_2 = \log(2.0), \delta = 25, \nu = 0.0$	ρ_a	**-0.226 (-0.228, -0.225)**	**-0.027 (-0.028, -0.026)**
$\omega_1 = \omega_2 = \log(2.0), \delta = 25, \nu = 0.0$	ρ_d	**-0.015 (-0.015, -0.015)**	**-0.007 (-0.008, -0.007)**
**Additional scenarios:**			
$i = 3 \times 4$	ρ_a	**-0.054 (-0.056, -0.052)**	-0.001 (-0.002, 0.001)
$i = 3 \times 4$	ρ_d	**-0.006 (-0.007, -0.005)**	-0.001 (-0.002, 0.000)
$i = 3 \times 4, N = 100$	ρ_a	**-0.054 (-0.055, -0.053)**	**-0.001 (-0.003, -0.000)**
$i = 3 \times 4, N = 100$	ρ_d	**-0.006 (-0.007, -0.006)**	**-0.001 (-0.002, -0.001)**
$\sigma^2_\alpha = 1, \sigma^2_\phi = 27.5$	ρ_a	**-0.035 (-0.036, -0.034)**	**-0.002 (-0.003, -0.001)**
$\sigma^2_\alpha = 1, \sigma^2_\phi = 27.5$	ρ_d	**-0.003 (-0.004, -0.003)**	**-0.001 (-0.001, -0.000)**

Table B7: Bias of ICCs for the constant treatment effect parametrisation, with 95% confidence intervals based on Monte Carlo errors. LMM denotes the linear mixed model, while JM denotes the joint model. Statistically significant biases are highlighted in bold. *(continued)*

Scenario	Parameter	LMM	JM
$\sigma^2_\alpha = 4, \sigma^2_\phi = 110$	ρ_a	**-0.066 (-0.067, -0.066)**	-0.001 (-0.001, 0.000)
$\sigma^2_\alpha = 4, \sigma^2_\phi = 110$	ρ_d	**-0.008 (-0.009, -0.008)**	**-0.001 (-0.002, -0.000)**

Table B8: Relative bias of ICCs for the constant treatment effect parametrisation, with 95% confidence intervals based on Monte Carlo errors. LMM denotes the linear mixed model, while JM denotes the joint model. Statistically significant biases are highlighted in bold.

Scenario	Parameter	LMM	JM
**Main scenarios:**			
$\omega_1 = \omega_2 = \log(0.5), \delta = 0.0, \nu = -0.2$	ρ_a	**-0.383 (-0.385, -0.380)**	**-0.044 (-0.047, -0.042)**
$\omega_1 = \omega_2 = \log(0.5), \delta = 0.0, \nu = -0.2$	ρ_d	**-0.737 (-0.753, -0.721)**	**-0.367 (-0.389, -0.345)**
$\omega_1 = \omega_2 = \log(0.5), \delta = 0.0, \nu = 0.0$	ρ_a	**-0.384 (-0.386, -0.382)**	**-0.044 (-0.047, -0.042)**
$\omega_1 = \omega_2 = \log(0.5), \delta = 0.0, \nu = 0.0$	ρ_d	**-0.737 (-0.752, -0.722)**	**-0.384 (-0.406, -0.362)**
$\omega_1 = \omega_2 = \log(0.5), \delta = 5.0, \nu = -0.2$	ρ_a	**-0.383 (-0.385, -0.381)**	**-0.044 (-0.047, -0.042)**
$\omega_1 = \omega_2 = \log(0.5), \delta = 5.0, \nu = -0.2$	ρ_d	**-0.719 (-0.735, -0.704)**	**-0.361 (-0.385, -0.338)**
$\omega_1 = \omega_2 = \log(0.5), \delta = 5.0, \nu = 0.0$	ρ_a	**-0.385 (-0.387, -0.383)**	**-0.045 (-0.048, -0.042)**
$\omega_1 = \omega_2 = \log(0.5), \delta = 5.0, \nu = 0.0$	ρ_d	**-0.731 (-0.746, -0.716)**	**-0.348 (-0.371, -0.325)**
$\omega_1 = \omega_2 = \log(0.5), \delta = 25, \nu = -0.2$	ρ_a	**-0.384 (-0.386, -0.382)**	**-0.045 (-0.048, -0.042)**
$\omega_1 = \omega_2 = \log(0.5), \delta = 25, \nu = -0.2$	ρ_d	**-0.734 (-0.750, -0.718)**	**-0.349 (-0.374, -0.323)**
$\omega_1 = \omega_2 = \log(0.5), \delta = 25, \nu = 0.0$	ρ_a	**-0.386 (-0.388, -0.384)**	**-0.046 (-0.049, -0.044)**
$\omega_1 = \omega_2 = \log(0.5), \delta = 25, \nu = 0.0$	ρ_d	**-0.742 (-0.756, -0.727)**	**-0.363 (-0.384, -0.342)**
$\omega_1 = \omega_2 = \log(0.9), \delta = 0.0, \nu = -0.2$	ρ_a	**-0.091 (-0.092, -0.089)**	-0.000 (-0.002, 0.002)
$\omega_1 = \omega_2 = \log(0.9), \delta = 0.0, \nu = -0.2$	ρ_d	**-0.248 (-0.273, -0.224)**	**-0.031 (-0.059, -0.002)**
$\omega_1 = \omega_2 = \log(0.9), \delta = 0.0, \nu = 0.0$	ρ_a	**-0.093 (-0.095, -0.091)**	-0.001 (-0.002, 0.001)
$\omega_1 = \omega_2 = \log(0.9), \delta = 0.0, \nu = 0.0$	ρ_d	**-0.288 (-0.313, -0.264)**	**-0.067 (-0.095, -0.039)**
$\omega_1 = \omega_2 = \log(0.9), \delta = 5.0, \nu = -0.2$	ρ_a	**-0.091 (-0.093, -0.089)**	-0.000 (-0.002, 0.002)
$\omega_1 = \omega_2 = \log(0.9), \delta = 5.0, \nu = -0.2$	ρ_d	**-0.247 (-0.272, -0.222)**	**-0.029 (-0.057, -0.001)**
$\omega_1 = \omega_2 = \log(0.9), \delta = 5.0, \nu = 0.0$	ρ_a	**-0.092 (-0.094, -0.091)**	-0.001 (-0.003, 0.001)
$\omega_1 = \omega_2 = \log(0.9), \delta = 5.0, \nu = 0.0$	ρ_d	**-0.260 (-0.285, -0.236)**	-0.024 (-0.052, 0.004)
$\omega_1 = \omega_2 = \log(0.9), \delta = 25, \nu = -0.2$	ρ_a	**-0.090 (-0.092, -0.089)**	0.001 (-0.001, 0.002)
$\omega_1 = \omega_2 = \log(0.9), \delta = 25, \nu = -0.2$	ρ_d	**-0.250 (-0.276, -0.225)**	-0.019 (-0.047, 0.010)
$\omega_1 = \omega_2 = \log(0.9), \delta = 25, \nu = 0.0$	ρ_a	**-0.092 (-0.094, -0.091)**	-0.000 (-0.002, 0.001)
$\omega_1 = \omega_2 = \log(0.9), \delta = 25, \nu = 0.0$	ρ_d	**-0.257 (-0.283, -0.231)**	**-0.033 (-0.062, -0.005)**
$\omega_1 = \omega_2 = \log(1.0), \delta = 0.0, \nu = -0.2$	ρ_a	0.001 (-0.000, 0.003)	**0.002 (0.000, 0.003)**
$\omega_1 = \omega_2 = \log(1.0), \delta = 0.0, \nu = -0.2$	ρ_d	**-0.032 (-0.061, -0.004)**	-0.017 (-0.046, 0.011)
$\omega_1 = \omega_2 = \log(1.0), \delta = 0.0, \nu = 0.0$	ρ_a	0.001 (-0.001, 0.002)	0.001 (-0.000, 0.003)

Table B8: Relative bias of ICCs for the constant treatment effect parametrisation, with 95% confidence intervals based on Monte Carlo errors. LMM denotes the linear mixed model, while JM denotes the joint model. Statistically significant biases are highlighted in bold. *(continued)*

Scenario	Parameter	LMM	JM
$\omega_1 = \omega_2 = \log(1.0)$, $\delta = 0.0$, $\nu = 0.0$	ρ_d	**-0.075 (-0.102, -0.048)**	**-0.062 (-0.089, -0.035)**
$\omega_1 = \omega_2 = \log(1.0)$, $\delta = 5.0$, $\nu = -0.2$	ρ_a	0.000 (-0.001, 0.002)	0.001 (-0.001, 0.002)
$\omega_1 = \omega_2 = \log(1.0)$, $\delta = 5.0$, $\nu = -0.2$	ρ_d	**-0.049 (-0.076, -0.021)**	**-0.037 (-0.064, -0.010)**
$\omega_1 = \omega_2 = \log(1.0)$, $\delta = 5.0$, $\nu = 0.0$	ρ_a	0.000 (-0.001, 0.002)	0.000 (-0.001, 0.002)
$\omega_1 = \omega_2 = \log(1.0)$, $\delta = 5.0$, $\nu = 0.0$	ρ_d	**-0.040 (-0.068, -0.011)**	**-0.031 (-0.059, -0.003)**
$\omega_1 = \omega_2 = \log(1.0)$, $\delta = 25$, $\nu = -0.2$	ρ_a	-0.000 (-0.002, 0.001)	0.000 (-0.001, 0.002)
$\omega_1 = \omega_2 = \log(1.0)$, $\delta = 25$, $\nu = -0.2$	ρ_d	**-0.067 (-0.094, -0.039)**	**-0.047 (-0.074, -0.020)**
$\omega_1 = \omega_2 = \log(1.0)$, $\delta = 25$, $\nu = 0.0$	ρ_a	-0.001 (-0.002, 0.001)	-0.000 (-0.002, 0.001)
$\omega_1 = \omega_2 = \log(1.0)$, $\delta = 25$, $\nu = 0.0$	ρ_d	**-0.058 (-0.086, -0.030)**	**-0.045 (-0.073, -0.017)**
$\omega_1 = \omega_2 = \log(2.0)$, $\delta = 0.0$, $\nu = -0.2$	ρ_a	**-0.383 (-0.385, -0.381)**	**-0.045 (-0.048, -0.043)**
$\omega_1 = \omega_2 = \log(2.0)$, $\delta = 0.0$, $\nu = -0.2$	ρ_d	**-0.732 (-0.747, -0.717)**	**-0.371 (-0.394, -0.349)**
$\omega_1 = \omega_2 = \log(2.0)$, $\delta = 0.0$, $\nu = 0.0$	ρ_a	**-0.385 (-0.387, -0.383)**	**-0.042 (-0.045, -0.040)**
$\omega_1 = \omega_2 = \log(2.0)$, $\delta = 0.0$, $\nu = 0.0$	ρ_d	**-0.736 (-0.750, -0.721)**	**-0.367 (-0.390, -0.344)**
$\omega_1 = \omega_2 = \log(2.0)$, $\delta = 5.0$, $\nu = -0.2$	ρ_a	**-0.383 (-0.385, -0.381)**	**-0.045 (-0.048, -0.043)**
$\omega_1 = \omega_2 = \log(2.0)$, $\delta = 5.0$, $\nu = -0.2$	ρ_d	**-0.744 (-0.759, -0.728)**	**-0.370 (-0.392, -0.347)**
$\omega_1 = \omega_2 = \log(2.0)$, $\delta = 5.0$, $\nu = 0.0$	ρ_a	**-0.385 (-0.387, -0.383)**	**-0.044 (-0.046, -0.041)**
$\omega_1 = \omega_2 = \log(2.0)$, $\delta = 5.0$, $\nu = 0.0$	ρ_d	**-0.732 (-0.747, -0.717)**	**-0.374 (-0.397, -0.351)**
$\omega_1 = \omega_2 = \log(2.0)$, $\delta = 25$, $\nu = -0.2$	ρ_a	**-0.383 (-0.385, -0.381)**	**-0.045 (-0.047, -0.042)**
$\omega_1 = \omega_2 = \log(2.0)$, $\delta = 25$, $\nu = -0.2$	ρ_d	**-0.735 (-0.751, -0.720)**	**-0.342 (-0.365, -0.319)**
$\omega_1 = \omega_2 = \log(2.0)$, $\delta = 25$, $\nu = 0.0$	ρ_a	**-0.385 (-0.387, -0.383)**	**-0.046 (-0.048, -0.043)**
$\omega_1 = \omega_2 = \log(2.0)$, $\delta = 25$, $\nu = 0.0$	ρ_d	**-0.730 (-0.746, -0.715)**	**-0.357 (-0.380, -0.333)**
**Additional scenarios:**			
$i = 3 \times 4$	ρ_a	**-0.092 (-0.095, -0.089)**	-0.001 (-0.004, 0.002)
$i = 3 \times 4$	ρ_d	**-0.299 (-0.336, -0.262)**	-0.042 (-0.087, 0.002)
$i = 3 \times 4, N = 100$	ρ_a	**-0.092 (-0.094, -0.090)**	**-0.002 (-0.004, -0.000)**
$i = 3 \times 4, N = 100$	ρ_d	**-0.305 (-0.334, -0.276)**	**-0.069 (-0.106, -0.033)**
$\sigma^2_\alpha = 1, \sigma^2_\phi = 27.5$	ρ_a	**-0.085 (-0.087, -0.082)**	**-0.005 (-0.007, -0.002)**
$\sigma^2_\alpha = 1, \sigma^2_\phi = 27.5$	ρ_d	**-0.215 (-0.243, -0.186)**	**-0.044 (-0.075, -0.014)**
$\sigma^2_\alpha = 4, \sigma^2_\phi = 110$	ρ_a	**-0.090 (-0.091, -0.089)**	-0.001 (-0.002, 0.000)
$\sigma^2_\alpha = 4, \sigma^2_\phi = 110$	ρ_d	**-0.317 (-0.341, -0.294)**	**-0.033 (-0.062, -0.005)**

Table B9: Coverage probability of ICCs for the constant treatment effect parametrisation, with 95% confidence intervals based on Monte Carlo errors. LMM denotes the linear mixed model, while JM denotes the joint model.

Scenario	Parameter	LMM	JM
**Main scenarios:**			
$\omega_1 = \omega_2 = \log(0.5)$, $\delta = 0.0$, $\nu = -0.2$	ρ_a	0.000 (0.000, 0.000)	0.733 (0.700, 0.766)
$\omega_1 = \omega_2 = \log(0.5)$, $\delta = 0.0$, $\nu = -0.2$	ρ_d	0.265 (0.238, 0.293)	0.672 (0.637, 0.707)
$\omega_1 = \omega_2 = \log(0.5)$, $\delta = 0.0$, $\nu = 0.0$	ρ_a	0.000 (0.000, 0.000)	0.749 (0.716, 0.782)
$\omega_1 = \omega_2 = \log(0.5)$, $\delta = 0.0$, $\nu = 0.0$	ρ_d	0.279 (0.251, 0.307)	0.631 (0.594, 0.668)
$\omega_1 = \omega_2 = \log(0.5)$, $\delta = 5.0$, $\nu = -0.2$	ρ_a	0.000 (0.000, 0.000)	0.783 (0.752, 0.815)
$\omega_1 = \omega_2 = \log(0.5)$, $\delta = 5.0$, $\nu = -0.2$	ρ_d	0.313 (0.284, 0.341)	0.668 (0.632, 0.705)
$\omega_1 = \omega_2 = \log(0.5)$, $\delta = 5.0$, $\nu = 0.0$	ρ_a	0.000 (0.000, 0.000)	0.733 (0.699, 0.766)
$\omega_1 = \omega_2 = \log(0.5)$, $\delta = 5.0$, $\nu = 0.0$	ρ_d	0.297 (0.269, 0.325)	0.699 (0.664, 0.734)
$\omega_1 = \omega_2 = \log(0.5)$, $\delta = 25$, $\nu = -0.2$	ρ_a	0.000 (0.000, 0.000)	0.742 (0.708, 0.776)
$\omega_1 = \omega_2 = \log(0.5)$, $\delta = 25$, $\nu = -0.2$	ρ_d	0.284 (0.256, 0.311)	0.667 (0.630, 0.704)
$\omega_1 = \omega_2 = \log(0.5)$, $\delta = 25$, $\nu = 0.0$	ρ_a	0.000 (0.000, 0.000)	0.733 (0.700, 0.766)
$\omega_1 = \omega_2 = \log(0.5)$, $\delta = 25$, $\nu = 0.0$	ρ_d	0.299 (0.271, 0.327)	0.679 (0.644, 0.714)
$\omega_1 = \omega_2 = \log(0.9)$, $\delta = 0.0$, $\nu = -0.2$	ρ_a	0.074 (0.058, 0.089)	0.950 (0.936, 0.964)
$\omega_1 = \omega_2 = \log(0.9)$, $\delta = 0.0$, $\nu = -0.2$	ρ_d	0.822 (0.799, 0.845)	0.915 (0.898, 0.932)
$\omega_1 = \omega_2 = \log(0.9)$, $\delta = 0.0$, $\nu = 0.0$	ρ_a	0.075 (0.059, 0.091)	0.956 (0.943, 0.969)
$\omega_1 = \omega_2 = \log(0.9)$, $\delta = 0.0$, $\nu = 0.0$	ρ_d	0.789 (0.764, 0.814)	0.903 (0.884, 0.921)
$\omega_1 = \omega_2 = \log(0.9)$, $\delta = 5.0$, $\nu = -0.2$	ρ_a	0.068 (0.053, 0.083)	0.951 (0.937, 0.964)
$\omega_1 = \omega_2 = \log(0.9)$, $\delta = 5.0$, $\nu = -0.2$	ρ_d	0.825 (0.802, 0.848)	0.922 (0.905, 0.938)
$\omega_1 = \omega_2 = \log(0.9)$, $\delta = 5.0$, $\nu = 0.0$	ρ_a	0.069 (0.053, 0.084)	0.944 (0.930, 0.959)
$\omega_1 = \omega_2 = \log(0.9)$, $\delta = 5.0$, $\nu = 0.0$	ρ_d	0.815 (0.791, 0.838)	0.927 (0.911, 0.943)
$\omega_1 = \omega_2 = \log(0.9)$, $\delta = 25$, $\nu = -0.2$	ρ_a	0.071 (0.055, 0.086)	0.969 (0.958, 0.980)
$\omega_1 = \omega_2 = \log(0.9)$, $\delta = 25$, $\nu = -0.2$	ρ_d	0.802 (0.778, 0.826)	0.924 (0.907, 0.940)
$\omega_1 = \omega_2 = \log(0.9)$, $\delta = 25$, $\nu = 0.0$	ρ_a	0.069 (0.053, 0.084)	0.957 (0.945, 0.969)
$\omega_1 = \omega_2 = \log(0.9)$, $\delta = 25$, $\nu = 0.0$	ρ_d	0.810 (0.786, 0.834)	0.921 (0.904, 0.937)
$\omega_1 = \omega_2 = \log(1.0)$, $\delta = 0.0$, $\nu = -0.2$	ρ_a	0.945 (0.931, 0.959)	0.944 (0.930, 0.958)
$\omega_1 = \omega_2 = \log(1.0)$, $\delta = 0.0$, $\nu = -0.2$	ρ_d	0.907 (0.889, 0.924)	0.916 (0.899, 0.933)
$\omega_1 = \omega_2 = \log(1.0)$, $\delta = 0.0$, $\nu = 0.0$	ρ_a	0.963 (0.951, 0.974)	0.962 (0.950, 0.974)
$\omega_1 = \omega_2 = \log(1.0)$, $\delta = 0.0$, $\nu = 0.0$	ρ_d	0.901 (0.883, 0.919)	0.907 (0.890, 0.925)
$\omega_1 = \omega_2 = \log(1.0)$, $\delta = 5.0$, $\nu = -0.2$	ρ_a	0.959 (0.947, 0.971)	0.961 (0.949, 0.973)
$\omega_1 = \omega_2 = \log(1.0)$, $\delta = 5.0$, $\nu = -0.2$	ρ_d	0.919 (0.902, 0.935)	0.925 (0.909, 0.941)
$\omega_1 = \omega_2 = \log(1.0)$, $\delta = 5.0$, $\nu = 0.0$	ρ_a	0.950 (0.936, 0.963)	0.950 (0.936, 0.963)
$\omega_1 = \omega_2 = \log(1.0)$, $\delta = 5.0$, $\nu = 0.0$	ρ_d	0.908 (0.890, 0.925)	0.910 (0.892, 0.927)
$\omega_1 = \omega_2 = \log(1.0)$, $\delta = 25$, $\nu = -0.2$	ρ_a	0.944 (0.931, 0.958)	0.943 (0.929, 0.957)
$\omega_1 = \omega_2 = \log(1.0)$, $\delta = 25$, $\nu = -0.2$	ρ_d	0.900 (0.881, 0.918)	0.917 (0.901, 0.934)
$\omega_1 = \omega_2 = \log(1.0)$, $\delta = 25$, $\nu = 0.0$	ρ_a	0.960 (0.948, 0.972)	0.959 (0.947, 0.971)
$\omega_1 = \omega_2 = \log(1.0)$, $\delta = 25$, $\nu = 0.0$	ρ_d	0.900 (0.882, 0.918)	0.906 (0.888, 0.924)
$\omega_1 = \omega_2 = \log(2.0)$, $\delta = 0.0$, $\nu = -0.2$	ρ_a	0.000 (0.000, 0.000)	0.739 (0.704, 0.774)

Table B9: Coverage probability of ICCs for the constant treatment effect parametrisation, with 95% confidence intervals based on Monte Carlo errors. LMM denotes the linear mixed model, while JM denotes the joint model. *(continued)*

Scenario	Parameter	LMM	JM
$\omega_1 = \omega_2 = \log(2.0)$, $\delta = 0.0$, $\nu = -0.2$	ρ_d	0.294 (0.266, 0.322)	0.686 (0.648, 0.723)
$\omega_1 = \omega_2 = \log(2.0)$, $\delta = 0.0$, $\nu = 0.0$	ρ_a	0.000 (0.000, 0.000)	0.776 (0.742, 0.809)
$\omega_1 = \omega_2 = \log(2.0)$, $\delta = 0.0$, $\nu = 0.0$	ρ_d	0.277 (0.250, 0.305)	0.675 (0.637, 0.712)
$\omega_1 = \omega_2 = \log(2.0)$, $\delta = 5.0$, $\nu = -0.2$	ρ_a	0.000 (0.000, 0.000)	0.744 (0.710, 0.779)
$\omega_1 = \omega_2 = \log(2.0)$, $\delta = 5.0$, $\nu = -0.2$	ρ_d	0.273 (0.246, 0.300)	0.663 (0.626, 0.700)
$\omega_1 = \omega_2 = \log(2.0)$, $\delta = 5.0$, $\nu = 0.0$	ρ_a	0.000 (0.000, 0.000)	0.765 (0.731, 0.798)
$\omega_1 = \omega_2 = \log(2.0)$, $\delta = 5.0$, $\nu = 0.0$	ρ_d	0.298 (0.270, 0.327)	0.658 (0.621, 0.696)
$\omega_1 = \omega_2 = \log(2.0)$, $\delta = 25$, $\nu = -0.2$	ρ_a	0.000 (0.000, 0.000)	0.757 (0.722, 0.791)
$\omega_1 = \omega_2 = \log(2.0)$, $\delta = 25$, $\nu = -0.2$	ρ_d	0.280 (0.253, 0.308)	0.722 (0.686, 0.758)
$\omega_1 = \omega_2 = \log(2.0)$, $\delta = 25$, $\nu = 0.0$	ρ_a	0.000 (0.000, 0.000)	0.755 (0.721, 0.790)
$\omega_1 = \omega_2 = \log(2.0)$, $\delta = 25$, $\nu = 0.0$	ρ_d	0.291 (0.263, 0.319)	0.686 (0.648, 0.723)
**Additional scenarios:**			
$i = 3 \times 4$	ρ_a	0.464 (0.434, 0.495)	0.952 (0.938, 0.966)
$i = 3 \times 4$	ρ_d	0.804 (0.779, 0.828)	0.912 (0.894, 0.930)
$i = 3 \times 4, N = 100$	ρ_a	0.158 (0.136, 0.180)	0.952 (0.938, 0.966)
$i = 3 \times 4, N = 100$	ρ_d	0.730 (0.703, 0.757)	0.860 (0.838, 0.883)
$\sigma^2_\alpha = 1, \sigma^2_\phi = 27.5$	ρ_a	0.470 (0.440, 0.500)	0.940 (0.925, 0.955)
$\sigma^2_\alpha = 1, \sigma^2_\phi = 27.5$	ρ_d	0.825 (0.802, 0.848)	0.901 (0.883, 0.920)
$\sigma^2_\alpha = 4, \sigma^2_\phi = 110$	ρ_a	0.000 (0.000, 0.000)	0.952 (0.938, 0.966)
$\sigma^2_\alpha = 4, \sigma^2_\phi = 110$	ρ_d	0.743 (0.716, 0.769)	0.903 (0.884, 0.922)

Table B10: Bias of variance components for the constant treatment effect parametrisation, with 95% confidence intervals based on Monte Carlo errors. LMM denotes the linear mixed model, while JM denotes the joint model. Statistically significant biases are highlighted in bold.

Scenario	Parameter	LMM	JM
**Main scenarios:**			
$\omega_1 = \omega_2 = \log(0.5)$, $\delta = 0.0$, $\nu = -0.2$	σ^2_α	**-1.657 (-1.678, -1.636)**	**-0.817 (-0.858, -0.775)**
$\omega_1 = \omega_2 = \log(0.5)$, $\delta = 0.0$, $\nu = -0.2$	σ^2_ϕ	**-32.444 (-32.555, -32.332)**	**-5.265 (-5.547, -4.983)**
$\omega_1 = \omega_2 = \log(0.5)$, $\delta = 0.0$, $\nu = -0.2$	σ^2_ϵ	**0.145 (0.082, 0.207)**	**-0.363 (-0.435, -0.291)**
$\omega_1 = \omega_2 = \log(0.5)$, $\delta = 0.0$, $\nu = 0.0$	σ^2_α	**-1.657 (-1.677, -1.637)**	**-0.848 (-0.890, -0.806)**
$\omega_1 = \omega_2 = \log(0.5)$, $\delta = 0.0$, $\nu = 0.0$	σ^2_ϕ	**-32.533 (-32.643, -32.423)**	**-5.293 (-5.568, -5.018)**
$\omega_1 = \omega_2 = \log(0.5)$, $\delta = 0.0$, $\nu = 0.0$	σ^2_ϵ	**0.174 (0.113, 0.236)**	**-0.395 (-0.469, -0.321)**
$\omega_1 = \omega_2 = \log(0.5)$, $\delta = 5.0$, $\nu = -0.2$	σ^2_α	**-1.634 (-1.654, -1.613)**	**-0.804 (-0.850, -0.758)**
$\omega_1 = \omega_2 = \log(0.5)$, $\delta = 5.0$, $\nu = -0.2$	σ^2_ϕ	**-32.488 (-32.594, -32.381)**	**-5.341 (-5.609, -5.072)**

Table B10: Bias of variance components for the constant treatment effect parametrisation, with 95% confidence intervals based on Monte Carlo errors. LMM denotes the linear mixed model, while JM denotes the joint model. Statistically significant biases are highlighted in bold. *(continued)*

Scenario	Parameter	LMM	JM
$\omega_1 = \omega_2 = \log(0.5)$, $\delta = 5.0$, $\nu = -0.2$	σ^2_ϵ	**0.197 (0.134, 0.259)**	**-0.399 (-0.475, -0.323)**
$\omega_1 = \omega_2 = \log(0.5)$, $\delta = 5.0$, $\nu = 0.0$	σ^2_α	**-1.649 (-1.669, -1.629)**	**-0.778 (-0.822, -0.734)**
$\omega_1 = \omega_2 = \log(0.5)$, $\delta = 5.0$, $\nu = 0.0$	σ^2_ϕ	**-32.581 (-32.692, -32.471)**	**-5.370 (-5.642, -5.099)**
$\omega_1 = \omega_2 = \log(0.5)$, $\delta = 5.0$, $\nu = 0.0$	σ^2_ϵ	**0.150 (0.087, 0.212)**	**-0.350 (-0.426, -0.274)**
$\omega_1 = \omega_2 = \log(0.5)$, $\delta = 25$, $\nu = -0.2$	σ^2_α	**-1.653 (-1.673, -1.632)**	**-0.781 (-0.830, -0.733)**
$\omega_1 = \omega_2 = \log(0.5)$, $\delta = 25$, $\nu = -0.2$	σ^2_ϕ	**-32.489 (-32.602, -32.376)**	**-5.366 (-5.666, -5.066)**
$\omega_1 = \omega_2 = \log(0.5)$, $\delta = 25$, $\nu = -0.2$	σ^2_ϵ	**0.185 (0.123, 0.248)**	**-0.371 (-0.445, -0.297)**
$\omega_1 = \omega_2 = \log(0.5)$, $\delta = 25$, $\nu = 0.0$	σ^2_α	**-1.665 (-1.684, -1.646)**	**-0.814 (-0.853, -0.775)**
$\omega_1 = \omega_2 = \log(0.5)$, $\delta = 25$, $\nu = 0.0$	σ^2_ϕ	**-32.629 (-32.736, -32.521)**	**-5.598 (-5.858, -5.339)**
$\omega_1 = \omega_2 = \log(0.5)$, $\delta = 25$, $\nu = 0.0$	σ^2_ϵ	**0.154 (0.092, 0.216)**	**-0.402 (-0.475, -0.329)**
$\omega_1 = \omega_2 = \log(0.9)$, $\delta = 0.0$, $\nu = -0.2$	σ^2_α	**-0.656 (-0.701, -0.612)**	**-0.058 (-0.116, -0.000)**
$\omega_1 = \omega_2 = \log(0.9)$, $\delta = 0.0$, $\nu = -0.2$	σ^2_ϕ	**-10.083 (-10.233, -9.933)**	0.023 (-0.184, 0.230)
$\omega_1 = \omega_2 = \log(0.9)$, $\delta = 0.0$, $\nu = -0.2$	σ^2_ϵ	**0.268 (0.205, 0.332)**	**-0.082 (-0.146, -0.018)**
$\omega_1 = \omega_2 = \log(0.9)$, $\delta = 0.0$, $\nu = 0.0$	σ^2_α	**-0.729 (-0.774, -0.685)**	**-0.132 (-0.190, -0.075)**
$\omega_1 = \omega_2 = \log(0.9)$, $\delta = 0.0$, $\nu = 0.0$	σ^2_ϕ	**-10.208 (-10.361, -10.054)**	0.066 (-0.151, 0.282)
$\omega_1 = \omega_2 = \log(0.9)$, $\delta = 0.0$, $\nu = 0.0$	σ^2_ϵ	**0.314 (0.248, 0.381)**	**-0.080 (-0.147, -0.013)**
$\omega_1 = \omega_2 = \log(0.9)$, $\delta = 5.0$, $\nu = -0.2$	σ^2_α	**-0.654 (-0.699, -0.610)**	-0.056 (-0.113, 0.001)
$\omega_1 = \omega_2 = \log(0.9)$, $\delta = 5.0$, $\nu = -0.2$	σ^2_ϕ	**-10.110 (-10.261, -9.959)**	0.022 (-0.187, 0.232)
$\omega_1 = \omega_2 = \log(0.9)$, $\delta = 5.0$, $\nu = -0.2$	σ^2_ϵ	**0.269 (0.206, 0.331)**	**-0.098 (-0.160, -0.035)**
$\omega_1 = \omega_2 = \log(0.9)$, $\delta = 5.0$, $\nu = 0.0$	σ^2_α	**-0.678 (-0.722, -0.634)**	-0.046 (-0.102, 0.010)
$\omega_1 = \omega_2 = \log(0.9)$, $\delta = 5.0$, $\nu = 0.0$	σ^2_ϕ	**-10.165 (-10.322, -10.009)**	-0.010 (-0.224, 0.204)
$\omega_1 = \omega_2 = \log(0.9)$, $\delta = 5.0$, $\nu = 0.0$	σ^2_ϵ	**0.336 (0.272, 0.401)**	-0.042 (-0.107, 0.022)
$\omega_1 = \omega_2 = \log(0.9)$, $\delta = 25$, $\nu = -0.2$	σ^2_α	**-0.660 (-0.705, -0.615)**	-0.034 (-0.092, 0.024)
$\omega_1 = \omega_2 = \log(0.9)$, $\delta = 25$, $\nu = -0.2$	σ^2_ϕ	**-10.046 (-10.196, -9.896)**	0.098 (-0.111, 0.307)
$\omega_1 = \omega_2 = \log(0.9)$, $\delta = 25$, $\nu = -0.2$	σ^2_ϵ	**0.267 (0.206, 0.328)**	**-0.087 (-0.149, -0.025)**
$\omega_1 = \omega_2 = \log(0.9)$, $\delta = 25$, $\nu = 0.0$	σ^2_α	**-0.673 (-0.719, -0.627)**	**-0.066 (-0.124, -0.008)**
$\omega_1 = \omega_2 = \log(0.9)$, $\delta = 25$, $\nu = 0.0$	σ^2_ϕ	**-10.187 (-10.341, -10.033)**	0.038 (-0.176, 0.252)
$\omega_1 = \omega_2 = \log(0.9)$, $\delta = 25$, $\nu = 0.0$	σ^2_ϵ	**0.314 (0.252, 0.375)**	-0.057 (-0.117, 0.004)
$\omega_1 = \omega_2 = \log(1.0)$, $\delta = 0.0$, $\nu = -0.2$	σ^2_α	-0.057 (-0.115, 0.001)	-0.026 (-0.084, 0.032)
$\omega_1 = \omega_2 = \log(1.0)$, $\delta = 0.0$, $\nu = -0.2$	σ^2_ϕ	**0.207 (0.035, 0.378)**	**0.240 (0.067, 0.413)**
$\omega_1 = \omega_2 = \log(1.0)$, $\delta = 0.0$, $\nu = -0.2$	σ^2_ϵ	**-0.060 (-0.118, -0.002)**	**-0.072 (-0.131, -0.013)**
$\omega_1 = \omega_2 = \log(1.0)$, $\delta = 0.0$, $\nu = 0.0$	σ^2_α	**-0.147 (-0.203, -0.091)**	**-0.119 (-0.175, -0.064)**
$\omega_1 = \omega_2 = \log(1.0)$, $\delta = 0.0$, $\nu = 0.0$	σ^2_ϕ	**0.194 (0.028, 0.360)**	**0.229 (0.060, 0.397)**
$\omega_1 = \omega_2 = \log(1.0)$, $\delta = 0.0$, $\nu = 0.0$	σ^2_ϵ	**-0.095 (-0.156, -0.033)**	**-0.101 (-0.163, -0.039)**
$\omega_1 = \omega_2 = \log(1.0)$, $\delta = 5.0$, $\nu = -0.2$	σ^2_α	**-0.096 (-0.151, -0.041)**	**-0.073 (-0.128, -0.017)**
$\omega_1 = \omega_2 = \log(1.0)$, $\delta = 5.0$, $\nu = -0.2$	σ^2_ϕ	0.008 (-0.160, 0.176)	0.028 (-0.141, 0.198)
$\omega_1 = \omega_2 = \log(1.0)$, $\delta = 5.0$, $\nu = -0.2$	σ^2_ϵ	**-0.146 (-0.203, -0.088)**	**-0.159 (-0.217, -0.102)**
$\omega_1 = \omega_2 = \log(1.0)$, $\delta = 5.0$, $\nu = 0.0$	σ^2_α	**-0.076 (-0.133, -0.019)**	-0.058 (-0.115, 0.000)

Table B10: Bias of variance components for the constant treatment effect parametrisation, with 95% confidence intervals based on Monte Carlo errors. LMM denotes the linear mixed model, while JM denotes the joint model. Statistically significant biases are highlighted in bold. *(continued)*

Scenario	Parameter	LMM	JM
$\omega_1 = \omega_2 = \log(1.0), \delta = 5.0, \nu = 0.0$	σ^2_ϕ	0.062 (-0.106, 0.229)	0.089 (-0.080, 0.257)
$\omega_1 = \omega_2 = \log(1.0), \delta = 5.0, \nu = 0.0$	σ^2_ϵ	**-0.066 (-0.128, -0.005)**	**-0.068 (-0.130, -0.005)**
$\omega_1 = \omega_2 = \log(1.0), \delta = 25, \nu = -0.2$	σ^2_α	**-0.131 (-0.186, -0.075)**	**-0.090 (-0.146, -0.035)**
$\omega_1 = \omega_2 = \log(1.0), \delta = 25, \nu = -0.2$	σ^2_ϕ	0.070 (-0.101, 0.241)	0.108 (-0.066, 0.281)
$\omega_1 = \omega_2 = \log(1.0), \delta = 25, \nu = -0.2$	σ^2_ϵ	**-0.087 (-0.149, -0.024)**	**-0.087 (-0.150, -0.024)**
$\omega_1 = \omega_2 = \log(1.0), \delta = 25, \nu = 0.0$	σ^2_α	**-0.112 (-0.169, -0.056)**	**-0.086 (-0.143, -0.029)**
$\omega_1 = \omega_2 = \log(1.0), \delta = 25, \nu = 0.0$	σ^2_ϕ	0.033 (-0.130, 0.196)	0.058 (-0.106, 0.222)
$\omega_1 = \omega_2 = \log(1.0), \delta = 25, \nu = 0.0$	σ^2_ϵ	-0.032 (-0.095, 0.030)	-0.038 (-0.101, 0.025)
$\omega_1 = \omega_2 = \log(2.0), \delta = 0.0, \nu = -0.2$	σ^2_α	**-1.650 (-1.670, -1.630)**	**-0.825 (-0.868, -0.783)**
$\omega_1 = \omega_2 = \log(2.0), \delta = 0.0, \nu = -0.2$	σ^2_ϕ	**-32.435 (-32.541, -32.328)**	**-5.419 (-5.701, -5.136)**
$\omega_1 = \omega_2 = \log(2.0), \delta = 0.0, \nu = -0.2$	σ^2_ϵ	**0.184 (0.123, 0.245)**	**-0.374 (-0.452, -0.296)**
$\omega_1 = \omega_2 = \log(2.0), \delta = 0.0, \nu = 0.0$	σ^2_α	**-1.657 (-1.676, -1.638)**	**-0.815 (-0.858, -0.771)**
$\omega_1 = \omega_2 = \log(2.0), \delta = 0.0, \nu = 0.0$	σ^2_ϕ	**-32.555 (-32.664, -32.447)**	**-5.111 (-5.397, -4.826)**
$\omega_1 = \omega_2 = \log(2.0), \delta = 0.0, \nu = 0.0$	σ^2_ϵ	**0.166 (0.103, 0.229)**	**-0.410 (-0.489, -0.332)**
$\omega_1 = \omega_2 = \log(2.0), \delta = 5.0, \nu = -0.2$	σ^2_α	**-1.666 (-1.686, -1.646)**	**-0.822 (-0.864, -0.779)**
$\omega_1 = \omega_2 = \log(2.0), \delta = 5.0, \nu = -0.2$	σ^2_ϕ	**-32.434 (-32.543, -32.325)**	**-5.353 (-5.635, -5.070)**
$\omega_1 = \omega_2 = \log(2.0), \delta = 5.0, \nu = -0.2$	σ^2_ϵ	**0.167 (0.105, 0.228)**	**-0.342 (-0.418, -0.266)**
$\omega_1 = \omega_2 = \log(2.0), \delta = 5.0, \nu = 0.0$	σ^2_α	**-1.652 (-1.671, -1.632)**	**-0.830 (-0.873, -0.786)**
$\omega_1 = \omega_2 = \log(2.0), \delta = 5.0, \nu = 0.0$	σ^2_ϕ	**-32.566 (-32.675, -32.458)**	**-5.275 (-5.560, -4.990)**
$\omega_1 = \omega_2 = \log(2.0), \delta = 5.0, \nu = 0.0$	σ^2_ϵ	**0.168 (0.105, 0.230)**	**-0.420 (-0.497, -0.344)**
$\omega_1 = \omega_2 = \log(2.0), \delta = 25, \nu = -0.2$	σ^2_α	**-1.655 (-1.675, -1.634)**	**-0.767 (-0.811, -0.723)**
$\omega_1 = \omega_2 = \log(2.0), \delta = 25, \nu = -0.2$	σ^2_ϕ	**-32.452 (-32.560, -32.345)**	**-5.275 (-5.553, -4.997)**
$\omega_1 = \omega_2 = \log(2.0), \delta = 25, \nu = -0.2$	σ^2_ϵ	**0.222 (0.160, 0.284)**	**-0.290 (-0.368, -0.213)**
$\omega_1 = \omega_2 = \log(2.0), \delta = 25, \nu = 0.0$	σ^2_α	**-1.649 (-1.669, -1.629)**	**-0.799 (-0.843, -0.755)**
$\omega_1 = \omega_2 = \log(2.0), \delta = 25, \nu = 0.0$	σ^2_ϕ	**-32.564 (-32.674, -32.455)**	**-5.443 (-5.717, -5.169)**
$\omega_1 = \omega_2 = \log(2.0), \delta = 25, \nu = 0.0$	σ^2_ϵ	**0.215 (0.153, 0.276)**	**-0.315 (-0.392, -0.238)**
**Additional scenarios:**			
$i = 3 \times 4$	σ^2_α	**-0.743 (-0.811, -0.676)**	-0.072 (-0.164, 0.019)
$i = 3 \times 4$	σ^2_ϕ	**-10.107 (-10.367, -9.847)**	0.089 (-0.281, 0.459)
$i = 3 \times 4$	σ^2_ϵ	**0.196 (0.090, 0.303)**	**-0.122 (-0.233, -0.011)**
$i = 3 \times 4, N = 100$	σ^2_α	**-0.755 (-0.807, -0.703)**	**-0.132 (-0.206, -0.059)**
$i = 3 \times 4, N = 100$	σ^2_ϕ	**-10.088 (-10.266, -9.911)**	-0.052 (-0.308, 0.204)
$i = 3 \times 4, N = 100$	σ^2_ϵ	**0.285 (0.213, 0.357)**	-0.032 (-0.107, 0.043)
$\sigma^2_\alpha = 1, \sigma^2_\phi = 27.5$	σ^2_α	**-0.253 (-0.281, -0.226)**	**-0.045 (-0.077, -0.014)**
$\sigma^2_\alpha = 1, \sigma^2_\phi = 27.5$	σ^2_ϕ	**-3.494 (-3.590, -3.399)**	**-0.157 (-0.276, -0.039)**
$\sigma^2_\alpha = 1, \sigma^2_\phi = 27.5$	σ^2_ϵ	**0.216 (0.154, 0.278)**	-0.041 (-0.102, 0.021)
$\sigma^2_\alpha = 4, \sigma^2_\phi = 110$	σ^2_α	**-1.802 (-1.879, -1.725)**	**-0.130 (-0.246, -0.013)**
$\sigma^2_\alpha = 4, \sigma^2_\phi = 110$	σ^2_ϕ	**-28.851 (-29.108, -28.594)**	-0.083 (-0.526, 0.359)

Table B10: Bias of variance components for the constant treatment effect parametrisation, with 95% confidence intervals based on Monte Carlo errors. LMM denotes the linear mixed model, while JM denotes the joint model. Statistically significant biases are highlighted in bold. *(continued)*

Scenario	Parameter	LMM	JM
$\sigma^2_\alpha = 4, \sigma^2_\phi = 110$	σ^2_ϵ	**0.262 (0.200, 0.323)**	-0.064 (-0.129, 0.001)

Table B11: Relative bias of variance components for the constant treatment effect parametrisation, with 95% confidence intervals based on Monte Carlo errors. LMM denotes the linear mixed model, while JM denotes the joint model. Statistically significant biases are highlighted in bold.

Scenario	Parameter	LMM	JM
**Main scenarios:**			
$\omega_1 = \omega_2 = \log(0.5), \delta = 0.0, \nu = -0.2$	σ^2_α	**-0.829 (-0.839, -0.818)**	**-0.408 (-0.429, -0.388)**
$\omega_1 = \omega_2 = \log(0.5), \delta = 0.0, \nu = -0.2$	σ^2_ϕ	**-0.590 (-0.592, -0.588)**	**-0.096 (-0.101, -0.091)**
$\omega_1 = \omega_2 = \log(0.5), \delta = 0.0, \nu = -0.2$	σ^2_ϵ	**0.004 (0.002, 0.005)**	**-0.009 (-0.011, -0.007)**
$\omega_1 = \omega_2 = \log(0.5), \delta = 0.0, \nu = 0.0$	σ^2_α	**-0.829 (-0.839, -0.819)**	**-0.424 (-0.445, -0.403)**
$\omega_1 = \omega_2 = \log(0.5), \delta = 0.0, \nu = 0.0$	σ^2_ϕ	**-0.592 (-0.594, -0.590)**	**-0.096 (-0.101, -0.091)**
$\omega_1 = \omega_2 = \log(0.5), \delta = 0.0, \nu = 0.0$	σ^2_ϵ	**0.004 (0.003, 0.006)**	**-0.010 (-0.012, -0.008)**
$\omega_1 = \omega_2 = \log(0.5), \delta = 5.0, \nu = -0.2$	σ^2_α	**-0.817 (-0.827, -0.807)**	**-0.402 (-0.425, -0.379)**
$\omega_1 = \omega_2 = \log(0.5), \delta = 5.0, \nu = -0.2$	σ^2_ϕ	**-0.591 (-0.593, -0.589)**	**-0.097 (-0.102, -0.092)**
$\omega_1 = \omega_2 = \log(0.5), \delta = 5.0, \nu = -0.2$	σ^2_ϵ	**0.005 (0.003, 0.006)**	**-0.010 (-0.012, -0.008)**
$\omega_1 = \omega_2 = \log(0.5), \delta = 5.0, \nu = 0.0$	σ^2_α	**-0.824 (-0.834, -0.815)**	**-0.389 (-0.411, -0.367)**
$\omega_1 = \omega_2 = \log(0.5), \delta = 5.0, \nu = 0.0$	σ^2_ϕ	**-0.592 (-0.594, -0.590)**	**-0.098 (-0.103, -0.093)**
$\omega_1 = \omega_2 = \log(0.5), \delta = 5.0, \nu = 0.0$	σ^2_ϵ	**0.004 (0.002, 0.005)**	**-0.009 (-0.011, -0.007)**
$\omega_1 = \omega_2 = \log(0.5), \delta = 25, \nu = -0.2$	σ^2_α	**-0.826 (-0.837, -0.816)**	**-0.391 (-0.415, -0.366)**
$\omega_1 = \omega_2 = \log(0.5), \delta = 25, \nu = -0.2$	σ^2_ϕ	**-0.591 (-0.593, -0.589)**	**-0.098 (-0.103, -0.092)**
$\omega_1 = \omega_2 = \log(0.5), \delta = 25, \nu = -0.2$	σ^2_ϵ	**0.005 (0.003, 0.006)**	**-0.009 (-0.011, -0.007)**
$\omega_1 = \omega_2 = \log(0.5), \delta = 25, \nu = 0.0$	σ^2_α	**-0.833 (-0.842, -0.823)**	**-0.407 (-0.427, -0.387)**
$\omega_1 = \omega_2 = \log(0.5), \delta = 25, \nu = 0.0$	σ^2_ϕ	**-0.593 (-0.595, -0.591)**	**-0.102 (-0.107, -0.097)**
$\omega_1 = \omega_2 = \log(0.5), \delta = 25, \nu = 0.0$	σ^2_ϵ	**0.004 (0.002, 0.005)**	**-0.010 (-0.012, -0.008)**
$\omega_1 = \omega_2 = \log(0.9), \delta = 0.0, \nu = -0.2$	σ^2_α	**-0.328 (-0.350, -0.306)**	**-0.029 (-0.058, -0.000)**
$\omega_1 = \omega_2 = \log(0.9), \delta = 0.0, \nu = -0.2$	σ^2_ϕ	**-0.183 (-0.186, -0.181)**	0.000 (-0.003, 0.004)
$\omega_1 = \omega_2 = \log(0.9), \delta = 0.0, \nu = -0.2$	σ^2_ϵ	**0.007 (0.005, 0.008)**	**-0.002 (-0.004, -0.000)**
$\omega_1 = \omega_2 = \log(0.9), \delta = 0.0, \nu = 0.0$	σ^2_α	**-0.365 (-0.387, -0.342)**	**-0.066 (-0.095, -0.038)**
$\omega_1 = \omega_2 = \log(0.9), \delta = 0.0, \nu = 0.0$	σ^2_ϕ	**-0.186 (-0.188, -0.183)**	0.001 (-0.003, 0.005)
$\omega_1 = \omega_2 = \log(0.9), \delta = 0.0, \nu = 0.0$	σ^2_ϵ	**0.008 (0.006, 0.010)**	**-0.002 (-0.004, -0.000)**
$\omega_1 = \omega_2 = \log(0.9), \delta = 5.0, \nu = -0.2$	σ^2_α	**-0.327 (-0.349, -0.305)**	-0.028 (-0.056, 0.001)
$\omega_1 = \omega_2 = \log(0.9), \delta = 5.0, \nu = -0.2$	σ^2_ϕ	**-0.184 (-0.187, -0.181)**	0.000 (-0.003, 0.004)
$\omega_1 = \omega_2 = \log(0.9), \delta = 5.0, \nu = -0.2$	σ^2_ϵ	**0.007 (0.005, 0.008)**	**-0.002 (-0.004, -0.001)**

Table B11: Relative bias of variance components for the constant treatment effect parametrisation, with 95% confidence intervals based on Monte Carlo errors. LMM denotes the linear mixed model, while JM denotes the joint model. Statistically significant biases are highlighted in bold. *(continued)*

Scenario	Parameter	LMM	JM
$\omega_1 = \omega_2 = \log(0.9), \delta = 5.0, \nu = 0.0$	σ^2_α	**-0.339 (-0.361, -0.317)**	-0.023 (-0.051, 0.005)
$\omega_1 = \omega_2 = \log(0.9), \delta = 5.0, \nu = 0.0$	σ^2_ϕ	**-0.185 (-0.188, -0.182)**	-0.000 (-0.004, 0.004)
$\omega_1 = \omega_2 = \log(0.9), \delta = 5.0, \nu = 0.0$	σ^2_ϵ	**0.008 (0.007, 0.010)**	-0.001 (-0.003, 0.001)
$\omega_1 = \omega_2 = \log(0.9), \delta = 25, \nu = -0.2$	σ^2_α	**-0.330 (-0.353, -0.307)**	-0.017 (-0.046, 0.012)
$\omega_1 = \omega_2 = \log(0.9), \delta = 25, \nu = -0.2$	σ^2_ϕ	**-0.183 (-0.185, -0.180)**	0.002 (-0.002, 0.006)
$\omega_1 = \omega_2 = \log(0.9), \delta = 25, \nu = -0.2$	σ^2_ϵ	**0.007 (0.005, 0.008)**	**-0.002 (-0.004, -0.001)**
$\omega_1 = \omega_2 = \log(0.9), \delta = 25, \nu = 0.0$	σ^2_α	**-0.337 (-0.360, -0.313)**	**-0.033 (-0.062, -0.004)**
$\omega_1 = \omega_2 = \log(0.9), \delta = 25, \nu = 0.0$	σ^2_ϕ	**-0.185 (-0.188, -0.182)**	0.001 (-0.003, 0.005)
$\omega_1 = \omega_2 = \log(0.9), \delta = 25, \nu = 0.0$	σ^2_ϵ	**0.008 (0.006, 0.009)**	-0.001 (-0.003, 0.000)
$\omega_1 = \omega_2 = \log(1.0), \delta = 0.0, \nu = -0.2$	σ^2_α	-0.029 (-0.057, 0.000)	-0.013 (-0.042, 0.016)
$\omega_1 = \omega_2 = \log(1.0), \delta = 0.0, \nu = -0.2$	σ^2_ϕ	**0.004 (0.001, 0.007)**	**0.004 (0.001, 0.008)**
$\omega_1 = \omega_2 = \log(1.0), \delta = 0.0, \nu = -0.2$	σ^2_ϵ	**-0.001 (-0.003, -0.000)**	**-0.002 (-0.003, -0.000)**
$\omega_1 = \omega_2 = \log(1.0), \delta = 0.0, \nu = 0.0$	σ^2_α	**-0.073 (-0.101, -0.046)**	**-0.060 (-0.087, -0.032)**
$\omega_1 = \omega_2 = \log(1.0), \delta = 0.0, \nu = 0.0$	σ^2_ϕ	**0.004 (0.001, 0.007)**	**0.004 (0.001, 0.007)**
$\omega_1 = \omega_2 = \log(1.0), \delta = 0.0, \nu = 0.0$	σ^2_ϵ	**-0.002 (-0.004, -0.001)**	**-0.003 (-0.004, -0.001)**
$\omega_1 = \omega_2 = \log(1.0), \delta = 5.0, \nu = -0.2$	σ^2_α	**-0.048 (-0.076, -0.020)**	**-0.036 (-0.064, -0.009)**
$\omega_1 = \omega_2 = \log(1.0), \delta = 5.0, \nu = -0.2$	σ^2_ϕ	0.000 (-0.003, 0.003)	0.001 (-0.003, 0.004)
$\omega_1 = \omega_2 = \log(1.0), \delta = 5.0, \nu = -0.2$	σ^2_ϵ	**-0.004 (-0.005, -0.002)**	**-0.004 (-0.005, -0.003)**
$\omega_1 = \omega_2 = \log(1.0), \delta = 5.0, \nu = 0.0$	σ^2_α	**-0.038 (-0.067, -0.009)**	-0.029 (-0.058, 0.000)
$\omega_1 = \omega_2 = \log(1.0), \delta = 5.0, \nu = 0.0$	σ^2_ϕ	0.001 (-0.002, 0.004)	0.002 (-0.001, 0.005)
$\omega_1 = \omega_2 = \log(1.0), \delta = 5.0, \nu = 0.0$	σ^2_ϵ	**-0.002 (-0.003, -0.000)**	**-0.002 (-0.003, -0.000)**
$\omega_1 = \omega_2 = \log(1.0), \delta = 25, \nu = -0.2$	σ^2_α	**-0.065 (-0.093, -0.037)**	**-0.045 (-0.073, -0.017)**
$\omega_1 = \omega_2 = \log(1.0), \delta = 25, \nu = -0.2$	σ^2_ϕ	0.001 (-0.002, 0.004)	0.002 (-0.001, 0.005)
$\omega_1 = \omega_2 = \log(1.0), \delta = 25, \nu = -0.2$	σ^2_ϵ	**-0.002 (-0.004, -0.001)**	**-0.002 (-0.004, -0.001)**
$\omega_1 = \omega_2 = \log(1.0), \delta = 25, \nu = 0.0$	σ^2_α	**-0.056 (-0.085, -0.028)**	**-0.043 (-0.072, -0.015)**
$\omega_1 = \omega_2 = \log(1.0), \delta = 25, \nu = 0.0$	σ^2_ϕ	0.001 (-0.002, 0.004)	0.001 (-0.002, 0.004)
$\omega_1 = \omega_2 = \log(1.0), \delta = 25, \nu = 0.0$	σ^2_ϵ	-0.001 (-0.002, 0.001)	-0.001 (-0.003, 0.001)
$\omega_1 = \omega_2 = \log(2.0), \delta = 0.0, \nu = -0.2$	σ^2_α	**-0.825 (-0.835, -0.815)**	**-0.413 (-0.434, -0.391)**
$\omega_1 = \omega_2 = \log(2.0), \delta = 0.0, \nu = -0.2$	σ^2_ϕ	**-0.590 (-0.592, -0.588)**	**-0.099 (-0.104, -0.093)**
$\omega_1 = \omega_2 = \log(2.0), \delta = 0.0, \nu = -0.2$	σ^2_ϵ	**0.005 (0.003, 0.006)**	**-0.009 (-0.011, -0.007)**
$\omega_1 = \omega_2 = \log(2.0), \delta = 0.0, \nu = 0.0$	σ^2_α	**-0.828 (-0.838, -0.819)**	**-0.407 (-0.429, -0.386)**
$\omega_1 = \omega_2 = \log(2.0), \delta = 0.0, \nu = 0.0$	σ^2_ϕ	**-0.592 (-0.594, -0.590)**	**-0.093 (-0.098, -0.088)**
$\omega_1 = \omega_2 = \log(2.0), \delta = 0.0, \nu = 0.0$	σ^2_ϵ	**0.004 (0.003, 0.006)**	**-0.010 (-0.012, -0.008)**
$\omega_1 = \omega_2 = \log(2.0), \delta = 5.0, \nu = -0.2$	σ^2_α	**-0.833 (-0.843, -0.823)**	**-0.411 (-0.432, -0.389)**
$\omega_1 = \omega_2 = \log(2.0), \delta = 5.0, \nu = -0.2$	σ^2_ϕ	**-0.590 (-0.592, -0.588)**	**-0.097 (-0.102, -0.092)**
$\omega_1 = \omega_2 = \log(2.0), \delta = 5.0, \nu = -0.2$	σ^2_ϵ	**0.004 (0.003, 0.006)**	**-0.009 (-0.010, -0.007)**
$\omega_1 = \omega_2 = \log(2.0), \delta = 5.0, \nu = 0.0$	σ^2_α	**-0.826 (-0.836, -0.816)**	**-0.415 (-0.436, -0.393)**

Table B11: Relative bias of variance components for the constant treatment effect parametrisation, with 95% confidence intervals based on Monte Carlo errors. LMM denotes the linear mixed model, while JM denotes the joint model. Statistically significant biases are highlighted in bold. *(continued)*

Scenario	Parameter	LMM	JM
$\omega_1 = \omega_2 = \log(2.0)$, $\delta = 5.0$, $\nu = 0.0$	σ^2_ϕ	**-0.592 (-0.594, -0.590)**	**-0.096 (-0.101, -0.091)**
$\omega_1 = \omega_2 = \log(2.0)$, $\delta = 5.0$, $\nu = 0.0$	σ^2_ϵ	**0.004 (0.003, 0.006)**	**-0.011 (-0.012, -0.009)**
$\omega_1 = \omega_2 = \log(2.0)$, $\delta = 25$, $\nu = -0.2$	σ^2_α	**-0.827 (-0.838, -0.817)**	**-0.384 (-0.406, -0.362)**
$\omega_1 = \omega_2 = \log(2.0)$, $\delta = 25$, $\nu = -0.2$	σ^2_ϕ	**-0.590 (-0.592, -0.588)**	**-0.096 (-0.101, -0.091)**
$\omega_1 = \omega_2 = \log(2.0)$, $\delta = 25$, $\nu = -0.2$	σ^2_ϵ	**0.006 (0.004, 0.007)**	**-0.007 (-0.009, -0.005)**
$\omega_1 = \omega_2 = \log(2.0)$, $\delta = 25$, $\nu = 0.0$	σ^2_α	**-0.825 (-0.835, -0.815)**	**-0.400 (-0.422, -0.378)**
$\omega_1 = \omega_2 = \log(2.0)$, $\delta = 25$, $\nu = 0.0$	σ^2_ϕ	**-0.592 (-0.594, -0.590)**	**-0.099 (-0.104, -0.094)**
$\omega_1 = \omega_2 = \log(2.0)$, $\delta = 25$, $\nu = 0.0$	σ^2_ϵ	**0.005 (0.004, 0.007)**	**-0.008 (-0.010, -0.006)**
**Additional scenarios:**			
$i = 3 \times 4$	σ^2_α	**-0.372 (-0.405, -0.338)**	-0.036 (-0.082, 0.010)
$i = 3 \times 4$	σ^2_ϕ	**-0.184 (-0.188, -0.179)**	0.002 (-0.005, 0.008)
$i = 3 \times 4$	σ^2_ϵ	**0.005 (0.002, 0.008)**	**-0.003 (-0.006, -0.000)**
$i = 3 \times 4, N = 100$	σ^2_α	**-0.377 (-0.403, -0.351)**	**-0.066 (-0.103, -0.029)**
$i = 3 \times 4, N = 100$	σ^2_ϕ	**-0.183 (-0.187, -0.180)**	-0.001 (-0.006, 0.004)
$i = 3 \times 4, N = 100$	σ^2_ϵ	**0.007 (0.005, 0.009)**	-0.001 (-0.003, 0.001)
$\sigma^2_\alpha = 1, \sigma^2_\phi = 27.5$	σ^2_α	**-0.253 (-0.281, -0.226)**	**-0.045 (-0.077, -0.014)**
$\sigma^2_\alpha = 1, \sigma^2_\phi = 27.5$	σ^2_ϕ	**-0.127 (-0.131, -0.124)**	**-0.006 (-0.010, -0.001)**
$\sigma^2_\alpha = 1, \sigma^2_\phi = 27.5$	σ^2_ϵ	**0.005 (0.004, 0.007)**	-0.001 (-0.003, 0.001)
$\sigma^2_\alpha = 4, \sigma^2_\phi = 110$	σ^2_α	**-0.450 (-0.470, -0.431)**	**-0.032 (-0.062, -0.003)**
$\sigma^2_\alpha = 4, \sigma^2_\phi = 110$	σ^2_ϕ	**-0.262 (-0.265, -0.260)**	-0.001 (-0.005, 0.003)
$\sigma^2_\alpha = 4, \sigma^2_\phi = 110$	σ^2_ϵ	**0.007 (0.005, 0.008)**	-0.002 (-0.003, 0.000)

Table B12: Coverage probability of variance components for the constant treatment effect parametrisation, with 95% confidence intervals based on Monte Carlo errors. LMM denotes the linear mixed model, while JM denotes the joint model.

Scenario	Parameter	LMM	JM
**Main scenarios:**			
$\omega_1 = \omega_2 = \log(0.5)$, $\delta = 0.0$, $\nu = -0.2$	σ^2_α	0.052 (0.038, 0.066)	0.603 (0.566, 0.639)
$\omega_1 = \omega_2 = \log(0.5)$, $\delta = 0.0$, $\nu = -0.2$	σ^2_ϕ	0.000 (0.000, 0.000)	0.672 (0.637, 0.707)
$\omega_1 = \omega_2 = \log(0.5)$, $\delta = 0.0$, $\nu = -0.2$	σ^2_ϵ	0.960 (0.948, 0.972)	0.931 (0.912, 0.950)
$\omega_1 = \omega_2 = \log(0.5)$, $\delta = 0.0$, $\nu = 0.0$	σ^2_α	0.050 (0.037, 0.064)	0.578 (0.540, 0.615)
$\omega_1 = \omega_2 = \log(0.5)$, $\delta = 0.0$, $\nu = 0.0$	σ^2_ϕ	0.000 (0.000, 0.000)	0.687 (0.651, 0.722)
$\omega_1 = \omega_2 = \log(0.5)$, $\delta = 0.0$, $\nu = 0.0$	σ^2_ϵ	0.953 (0.940, 0.966)	0.936 (0.917, 0.955)

Table B12: Coverage probability of variance components for the constant treatment effect parametrisation, with 95% confidence intervals based on Monte Carlo errors. LMM denotes the linear mixed model, while JM denotes the joint model. *(continued)*

Scenario	Parameter	LMM	JM
$\omega_1 = \omega_2 = \log(0.5)$, $\delta = 5.0$, $\nu = -0.2$	σ^2_α	0.058 (0.044, 0.073)	0.606 (0.568, 0.644)
$\omega_1 = \omega_2 = \log(0.5)$, $\delta = 5.0$, $\nu = -0.2$	σ^2_ϕ	0.000 (0.000, 0.000)	0.698 (0.662, 0.733)
$\omega_1 = \omega_2 = \log(0.5)$, $\delta = 5.0$, $\nu = -0.2$	σ^2_ϵ	0.947 (0.933, 0.960)	0.928 (0.908, 0.948)
$\omega_1 = \omega_2 = \log(0.5)$, $\delta = 5.0$, $\nu = 0.0$	σ^2_α	0.053 (0.039, 0.066)	0.640 (0.604, 0.677)
$\omega_1 = \omega_2 = \log(0.5)$, $\delta = 5.0$, $\nu = 0.0$	σ^2_ϕ	0.000 (0.000, 0.000)	0.680 (0.644, 0.715)
$\omega_1 = \omega_2 = \log(0.5)$, $\delta = 5.0$, $\nu = 0.0$	σ^2_ϵ	0.956 (0.944, 0.969)	0.932 (0.913, 0.951)
$\omega_1 = \omega_2 = \log(0.5)$, $\delta = 25$, $\nu = -0.2$	σ^2_α	0.046 (0.033, 0.059)	0.607 (0.569, 0.645)
$\omega_1 = \omega_2 = \log(0.5)$, $\delta = 25$, $\nu = -0.2$	σ^2_ϕ	0.000 (0.000, 0.000)	0.667 (0.630, 0.704)
$\omega_1 = \omega_2 = \log(0.5)$, $\delta = 25$, $\nu = -0.2$	σ^2_ϵ	0.958 (0.945, 0.970)	0.940 (0.921, 0.958)
$\omega_1 = \omega_2 = \log(0.5)$, $\delta = 25$, $\nu = 0.0$	σ^2_α	0.035 (0.023, 0.046)	0.609 (0.573, 0.645)
$\omega_1 = \omega_2 = \log(0.5)$, $\delta = 25$, $\nu = 0.0$	σ^2_ϕ	0.000 (0.000, 0.000)	0.665 (0.630, 0.700)
$\omega_1 = \omega_2 = \log(0.5)$, $\delta = 25$, $\nu = 0.0$	σ^2_ϵ	0.951 (0.937, 0.964)	0.929 (0.910, 0.948)
$\omega_1 = \omega_2 = \log(0.9)$, $\delta = 0.0$, $\nu = -0.2$	σ^2_α	0.739 (0.712, 0.766)	0.914 (0.897, 0.931)
$\omega_1 = \omega_2 = \log(0.9)$, $\delta = 0.0$, $\nu = -0.2$	σ^2_ϕ	0.024 (0.015, 0.033)	0.953 (0.940, 0.966)
$\omega_1 = \omega_2 = \log(0.9)$, $\delta = 0.0$, $\nu = -0.2$	σ^2_ϵ	0.944 (0.930, 0.958)	0.948 (0.934, 0.962)
$\omega_1 = \omega_2 = \log(0.9)$, $\delta = 0.0$, $\nu = 0.0$	σ^2_α	0.707 (0.679, 0.734)	0.902 (0.883, 0.920)
$\omega_1 = \omega_2 = \log(0.9)$, $\delta = 0.0$, $\nu = 0.0$	σ^2_ϕ	0.034 (0.023, 0.045)	0.957 (0.944, 0.969)
$\omega_1 = \omega_2 = \log(0.9)$, $\delta = 0.0$, $\nu = 0.0$	σ^2_ϵ	0.936 (0.921, 0.951)	0.932 (0.916, 0.947)
$\omega_1 = \omega_2 = \log(0.9)$, $\delta = 5.0$, $\nu = -0.2$	σ^2_α	0.747 (0.721, 0.774)	0.916 (0.898, 0.933)
$\omega_1 = \omega_2 = \log(0.9)$, $\delta = 5.0$, $\nu = -0.2$	σ^2_ϕ	0.029 (0.019, 0.039)	0.950 (0.936, 0.963)
$\omega_1 = \omega_2 = \log(0.9)$, $\delta = 5.0$, $\nu = -0.2$	σ^2_ϵ	0.946 (0.933, 0.960)	0.952 (0.938, 0.965)
$\omega_1 = \omega_2 = \log(0.9)$, $\delta = 5.0$, $\nu = 0.0$	σ^2_α	0.745 (0.719, 0.772)	0.923 (0.907, 0.940)
$\omega_1 = \omega_2 = \log(0.9)$, $\delta = 5.0$, $\nu = 0.0$	σ^2_ϕ	0.027 (0.017, 0.036)	0.945 (0.931, 0.959)
$\omega_1 = \omega_2 = \log(0.9)$, $\delta = 5.0$, $\nu = 0.0$	σ^2_ϵ	0.933 (0.918, 0.948)	0.949 (0.936, 0.963)
$\omega_1 = \omega_2 = \log(0.9)$, $\delta = 25$, $\nu = -0.2$	σ^2_α	0.728 (0.701, 0.755)	0.925 (0.908, 0.941)
$\omega_1 = \omega_2 = \log(0.9)$, $\delta = 25$, $\nu = -0.2$	σ^2_ϕ	0.029 (0.019, 0.039)	0.962 (0.950, 0.974)
$\omega_1 = \omega_2 = \log(0.9)$, $\delta = 25$, $\nu = -0.2$	σ^2_ϵ	0.947 (0.934, 0.961)	0.945 (0.931, 0.959)
$\omega_1 = \omega_2 = \log(0.9)$, $\delta = 25$, $\nu = 0.0$	σ^2_α	0.736 (0.709, 0.762)	0.920 (0.903, 0.937)
$\omega_1 = \omega_2 = \log(0.9)$, $\delta = 25$, $\nu = 0.0$	σ^2_ϕ	0.032 (0.022, 0.043)	0.950 (0.937, 0.964)
$\omega_1 = \omega_2 = \log(0.9)$, $\delta = 25$, $\nu = 0.0$	σ^2_ϵ	0.949 (0.936, 0.963)	0.965 (0.954, 0.976)
$\omega_1 = \omega_2 = \log(1.0)$, $\delta = 0.0$, $\nu = -0.2$	σ^2_α	0.903 (0.885, 0.921)	0.914 (0.897, 0.931)
$\omega_1 = \omega_2 = \log(1.0)$, $\delta = 0.0$, $\nu = -0.2$	σ^2_ϕ	0.949 (0.935, 0.962)	0.950 (0.937, 0.963)
$\omega_1 = \omega_2 = \log(1.0)$, $\delta = 0.0$, $\nu = -0.2$	σ^2_ϵ	0.965 (0.954, 0.976)	0.966 (0.955, 0.977)
$\omega_1 = \omega_2 = \log(1.0)$, $\delta = 0.0$, $\nu = 0.0$	σ^2_α	0.899 (0.880, 0.917)	0.907 (0.890, 0.925)
$\omega_1 = \omega_2 = \log(1.0)$, $\delta = 0.0$, $\nu = 0.0$	σ^2_ϕ	0.963 (0.951, 0.974)	0.965 (0.954, 0.976)
$\omega_1 = \omega_2 = \log(1.0)$, $\delta = 0.0$, $\nu = 0.0$	σ^2_ϵ	0.950 (0.937, 0.963)	0.949 (0.936, 0.963)
$\omega_1 = \omega_2 = \log(1.0)$, $\delta = 5.0$, $\nu = -0.2$	σ^2_α	0.914 (0.897, 0.931)	0.923 (0.907, 0.939)
$\omega_1 = \omega_2 = \log(1.0)$, $\delta = 5.0$, $\nu = -0.2$	σ^2_ϕ	0.949 (0.936, 0.963)	0.950 (0.937, 0.964)

Table B12: Coverage probability of variance components for the constant treatment effect parametrisation, with 95% confidence intervals based on Monte Carlo errors. LMM denotes the linear mixed model, while JM denotes the joint model. *(continued)*

Scenario	Parameter	LMM	JM
$\omega_1 = \omega_2 = \log(1.0), \delta = 5.0, \nu = -0.2$	σ^2_ϵ	0.955 (0.943, 0.968)	0.957 (0.945, 0.970)
$\omega_1 = \omega_2 = \log(1.0), \delta = 5.0, \nu = 0.0$	σ^2_α	0.906 (0.888, 0.923)	0.909 (0.891, 0.926)
$\omega_1 = \omega_2 = \log(1.0), \delta = 5.0, \nu = 0.0$	σ^2_ϕ	0.955 (0.943, 0.968)	0.957 (0.945, 0.970)
$\omega_1 = \omega_2 = \log(1.0), \delta = 5.0, \nu = 0.0$	σ^2_ϵ	0.955 (0.943, 0.968)	0.954 (0.942, 0.967)
$\omega_1 = \omega_2 = \log(1.0), \delta = 25, \nu = -0.2$	σ^2_α	0.901 (0.883, 0.920)	0.918 (0.902, 0.935)
$\omega_1 = \omega_2 = \log(1.0), \delta = 25, \nu = -0.2$	σ^2_ϕ	0.945 (0.932, 0.959)	0.946 (0.932, 0.960)
$\omega_1 = \omega_2 = \log(1.0), \delta = 25, \nu = -0.2$	σ^2_ϵ	0.946 (0.933, 0.960)	0.948 (0.934, 0.962)
$\omega_1 = \omega_2 = \log(1.0), \delta = 25, \nu = 0.0$	σ^2_α	0.891 (0.872, 0.910)	0.899 (0.881, 0.918)
$\omega_1 = \omega_2 = \log(1.0), \delta = 25, \nu = 0.0$	σ^2_ϕ	0.964 (0.952, 0.975)	0.966 (0.955, 0.977)
$\omega_1 = \omega_2 = \log(1.0), \delta = 25, \nu = 0.0$	σ^2_ϵ	0.957 (0.945, 0.969)	0.956 (0.943, 0.969)
$\omega_1 = \omega_2 = \log(2.0), \delta = 0.0, \nu = -0.2$	σ^2_α	0.050 (0.037, 0.064)	0.595 (0.556, 0.635)
$\omega_1 = \omega_2 = \log(2.0), \delta = 0.0, \nu = -0.2$	σ^2_ϕ	0.000 (0.000, 0.000)	0.677 (0.640, 0.715)
$\omega_1 = \omega_2 = \log(2.0), \delta = 0.0, \nu = -0.2$	σ^2_ϵ	0.953 (0.940, 0.966)	0.936 (0.917, 0.956)
$\omega_1 = \omega_2 = \log(2.0), \delta = 0.0, \nu = 0.0$	σ^2_α	0.039 (0.027, 0.051)	0.612 (0.573, 0.651)
$\omega_1 = \omega_2 = \log(2.0), \delta = 0.0, \nu = 0.0$	σ^2_ϕ	0.000 (0.000, 0.000)	0.710 (0.673, 0.746)
$\omega_1 = \omega_2 = \log(2.0), \delta = 0.0, \nu = 0.0$	σ^2_ϵ	0.945 (0.931, 0.959)	0.919 (0.897, 0.941)
$\omega_1 = \omega_2 = \log(2.0), \delta = 5.0, \nu = -0.2$	σ^2_α	0.046 (0.033, 0.059)	0.605 (0.567, 0.644)
$\omega_1 = \omega_2 = \log(2.0), \delta = 5.0, \nu = -0.2$	σ^2_ϕ	0.000 (0.000, 0.000)	0.706 (0.670, 0.742)
$\omega_1 = \omega_2 = \log(2.0), \delta = 5.0, \nu = -0.2$	σ^2_ϵ	0.955 (0.942, 0.967)	0.925 (0.904, 0.946)
$\omega_1 = \omega_2 = \log(2.0), \delta = 5.0, \nu = 0.0$	σ^2_α	0.046 (0.033, 0.059)	0.595 (0.556, 0.635)
$\omega_1 = \omega_2 = \log(2.0), \delta = 5.0, \nu = 0.0$	σ^2_ϕ	0.000 (0.000, 0.000)	0.667 (0.629, 0.704)
$\omega_1 = \omega_2 = \log(2.0), \delta = 5.0, \nu = 0.0$	σ^2_ϵ	0.961 (0.949, 0.973)	0.925 (0.904, 0.946)
$\omega_1 = \omega_2 = \log(2.0), \delta = 25, \nu = -0.2$	σ^2_α	0.060 (0.045, 0.075)	0.650 (0.612, 0.688)
$\omega_1 = \omega_2 = \log(2.0), \delta = 25, \nu = -0.2$	σ^2_ϕ	0.000 (0.000, 0.000)	0.694 (0.657, 0.731)
$\omega_1 = \omega_2 = \log(2.0), \delta = 25, \nu = -0.2$	σ^2_ϵ	0.943 (0.929, 0.957)	0.947 (0.930, 0.965)
$\omega_1 = \omega_2 = \log(2.0), \delta = 25, \nu = 0.0$	σ^2_α	0.060 (0.045, 0.074)	0.629 (0.590, 0.668)
$\omega_1 = \omega_2 = \log(2.0), \delta = 25, \nu = 0.0$	σ^2_ϕ	0.000 (0.000, 0.000)	0.676 (0.638, 0.713)
$\omega_1 = \omega_2 = \log(2.0), \delta = 25, \nu = 0.0$	σ^2_ϵ	0.949 (0.936, 0.963)	0.938 (0.919, 0.958)
**Additional scenarios:**			
$i = 3 \times 4$	σ^2_α	0.757 (0.730, 0.783)	0.903 (0.884, 0.922)
$i = 3 \times 4$	σ^2_ϕ	0.317 (0.288, 0.346)	0.959 (0.946, 0.972)
$i = 3 \times 4$	σ^2_ϵ	0.951 (0.938, 0.964)	0.940 (0.925, 0.956)
$i = 3 \times 4, N = 100$	σ^2_α	0.677 (0.648, 0.705)	0.853 (0.830, 0.876)
$i = 3 \times 4, N = 100$	σ^2_ϕ	0.087 (0.070, 0.104)	0.944 (0.930, 0.959)
$i = 3 \times 4, N = 100$	σ^2_ϵ	0.948 (0.935, 0.962)	0.942 (0.927, 0.957)
$\sigma^2_\alpha = 1, \sigma^2_\phi = 27.5$	σ^2_α	0.797 (0.773, 0.822)	0.902 (0.884, 0.921)
$\sigma^2_\alpha = 1, \sigma^2_\phi = 27.5$	σ^2_ϕ	0.407 (0.377, 0.436)	0.943 (0.929, 0.957)
$\sigma^2_\alpha = 1, \sigma^2_\phi = 27.5$	σ^2_ϵ	0.949 (0.936, 0.963)	0.956 (0.943, 0.968)

Table B12: Coverage probability of variance components for the constant treatment effect parametrisation, with 95% confidence intervals based on Monte Carlo errors. LMM denotes the linear mixed model, while JM denotes the joint model. *(continued)*

Scenario	Parameter	LMM	JM
$\sigma^2_\alpha = 4, \sigma^2_\phi = 110$	σ^2_α	0.589 (0.559, 0.618)	0.899 (0.880, 0.919)
$\sigma^2_\alpha = 4, \sigma^2_\phi = 110$	σ^2_ϕ	0.000 (0.000, 0.000)	0.941 (0.926, 0.956)
$\sigma^2_\alpha = 4, \sigma^2_\phi = 110$	σ^2_ϵ	0.950 (0.937, 0.963)	0.956 (0.943, 0.970)

B.3 General time on treatment model

Bias (with 95% C.I. based on Monte Carlo standard errors) for the treatment effect on the longitudinal outcome are tabulated in Table B13, with relative bias in Table B14. Coverage probabilities are tabulated in Table B15.

Bias, relative bias, and coverage probabilities for the period effects are tabulated in Tables B16, B17 and B18, respectively.

Bias, relative bias, and coverage probabilities for the ICCs are tabulated in Tables B19, B20 and B21.

Finally, bias, relative bias, and coverage probabilities for the variance components are tabulated in Tables B22, B23 and B24.

Table B13: Bias of treatment effect on the longitudinal outcome for the general time on treatment effect parametrisation, with 95% confidence intervals based on Monte Carlo errors. LMM denotes the linear mixed model, while JM denotes the joint model. Statistically significant biases are highlighted in bold.

Scenario	Parameter	LMM	JM
**Main scenarios:**			
$\omega_1 = \omega_2 = \log(0.5)$, $\delta_0 = \delta_1 = \delta_2 = \delta_3 = 0.00$, $\nu = -0.2$	δ_0	**-0.030 (-0.051, -0.008)**	0.008 (-0.018, 0.034)
$\omega_1 = \omega_2 = \log(0.5)$, $\delta_0 = \delta_1 = \delta_2 = \delta_3 = 0.00$, $\nu = -0.2$	δ_1	**-0.077 (-0.106, -0.049)**	-0.034 (-0.068, 0.001)
$\omega_1 = \omega_2 = \log(0.5)$, $\delta_0 = \delta_1 = \delta_2 = \delta_3 = 0.00$, $\nu = -0.2$	δ_2	**-0.093 (-0.129, -0.057)**	-0.012 (-0.057, 0.032)
$\omega_1 = \omega_2 = \log(0.5)$, $\delta_0 = \delta_1 = \delta_2 = \delta_3 = 0.00$, $\nu = -0.2$	δ_3	**-0.111 (-0.160, -0.061)**	-0.024 (-0.084, 0.036)
$\omega_1 = \omega_2 = \log(0.5)$, $\delta_0 = \delta_1 = \delta_2 = \delta_3 = 0.00$, $\nu = 0.0$	δ_0	-0.010 (-0.031, 0.012)	-0.011 (-0.035, 0.014)
$\omega_1 = \omega_2 = \log(0.5)$, $\delta_0 = \delta_1 = \delta_2 = \delta_3 = 0.00$, $\nu = 0.0$	δ_1	-0.008 (-0.036, 0.020)	-0.027 (-0.060, 0.005)
$\omega_1 = \omega_2 = \log(0.5)$, $\delta_0 = \delta_1 = \delta_2 = \delta_3 = 0.00$, $\nu = 0.0$	δ_2	-0.011 (-0.048, 0.027)	**-0.050 (-0.095, -0.004)**
$\omega_1 = \omega_2 = \log(0.5)$, $\delta_0 = \delta_1 = \delta_2 = \delta_3 = 0.00$, $\nu = 0.0$	δ_3	0.025 (-0.026, 0.076)	-0.016 (-0.075, 0.043)
$\omega_1 = \omega_2 = \log(0.5)$, $\delta_0 = 0.00, \delta_1 = 2.50, \delta_2 = 5.00, \delta_3 = 6.25$, $\nu = -0.2$	δ_0	-0.014 (-0.036, 0.008)	0.015 (-0.011, 0.041)
$\omega_1 = \omega_2 = \log(0.5)$, $\delta_0 = 0.00, \delta_1 = 2.50, \delta_2 = 5.00, \delta_3 = 6.25$, $\nu = -0.2$	δ_1	**-0.035 (-0.064, -0.006)**	0.015 (-0.020, 0.049)
$\omega_1 = \omega_2 = \log(0.5)$, $\delta_0 = 0.00, \delta_1 = 2.50, \delta_2 = 5.00, \delta_3 = 6.25$, $\nu = -0.2$	δ_2	**-0.064 (-0.102, -0.026)**	-0.003 (-0.047, 0.041)
$\omega_1 = \omega_2 = \log(0.5)$, $\delta_0 = 0.00, \delta_1 = 2.50, \delta_2 = 5.00, \delta_3 = 6.25$, $\nu = -0.2$	δ_3	**-0.069 (-0.120, -0.018)**	0.014 (-0.047, 0.075)
$\omega_1 = \omega_2 = \log(0.5)$, $\delta_0 = 0.00, \delta_1 = 2.50, \delta_2 = 5.00, \delta_3 = 6.25$, $\nu = 0.0$	δ_0	0.002 (-0.022, 0.025)	-0.004 (-0.032, 0.025)
$\omega_1 = \omega_2 = \log(0.5)$, $\delta_0 = 0.00, \delta_1 = 2.50, \delta_2 = 5.00, \delta_3 = 6.25$, $\nu = 0.0$	δ_1	-0.003 (-0.033, 0.027)	-0.014 (-0.049, 0.020)
$\omega_1 = \omega_2 = \log(0.5)$, $\delta_0 = 0.00, \delta_1 = 2.50, \delta_2 = 5.00, \delta_3 = 6.25$, $\nu = 0.0$	δ_2	-0.001 (-0.039, 0.038)	-0.006 (-0.051, 0.039)
$\omega_1 = \omega_2 = \log(0.5)$, $\delta_0 = 0.00, \delta_1 = 2.50, \delta_2 = 5.00, \delta_3 = 6.25$, $\nu = 0.0$	δ_3	-0.013 (-0.066, 0.040)	-0.009 (-0.070, 0.053)
$\omega_1 = \omega_2 = \log(0.5)$, $\delta_0 = 0.00, \delta_1 = 12.50, \delta_2 = 25.00, \delta_3 = 31.25$, $\nu = -0.2$	δ_0	**-0.040 (-0.063, -0.017)**	-0.006 (-0.034, 0.022)
$\omega_1 = \omega_2 = \log(0.5)$, $\delta_0 = 0.00, \delta_1 = 12.50, \delta_2 = 25.00, \delta_3 = 31.25$, $\nu = -0.2$	δ_1	**-0.054 (-0.083, -0.024)**	-0.001 (-0.037, 0.035)
$\omega_1 = \omega_2 = \log(0.5)$, $\delta_0 = 0.00, \delta_1 = 12.50, \delta_2 = 25.00, \delta_3 = 31.25$, $\nu = -0.2$	δ_2	**-0.085 (-0.123, -0.046)**	-0.002 (-0.048, 0.044)
$\omega_1 = \omega_2 = \log(0.5)$, $\delta_0 = 0.00, \delta_1 = 12.50, \delta_2 = 25.00, \delta_3 = 31.25$, $\nu = -0.2$	δ_3	**-0.083 (-0.135, -0.032)**	0.023 (-0.039, 0.084)
$\omega_1 = \omega_2 = \log(0.5)$, $\delta_0 = 0.00, \delta_1 = 12.50, \delta_2 = 25.00, \delta_3 = 31.25$, $\nu = 0.0$	δ_0	0.009 (-0.015, 0.033)	0.013 (-0.014, 0.041)
$\omega_1 = \omega_2 = \log(0.5)$, $\delta_0 = 0.00, \delta_1 = 12.50, \delta_2 = 25.00, \delta_3 = 31.25$, $\nu = 0.0$	δ_1	0.002 (-0.028, 0.032)	0.005 (-0.030, 0.040)
$\omega_1 = \omega_2 = \log(0.5)$, $\delta_0 = 0.00, \delta_1 = 12.50, \delta_2 = 25.00, \delta_3 = 31.25$, $\nu = 0.0$	δ_2	-0.001 (-0.040, 0.038)	0.008 (-0.038, 0.053)
$\omega_1 = \omega_2 = \log(0.5)$, $\delta_0 = 0.00, \delta_1 = 12.50, \delta_2 = 25.00, \delta_3 = 31.25$, $\nu = 0.0$	δ_3	0.006 (-0.047, 0.060)	-0.019 (-0.083, 0.044)

Table B13: Bias of treatment effect on the longitudinal outcome for the general time on treatment effect parametrisation, with 95% confidence intervals based on Monte Carlo errors. LMM denotes the linear mixed model, while JM denotes the joint model. Statistically significant biases are highlighted in bold. *(continued)*

Scenario	Parameter	LMM	JM
$\omega_1 = \omega_2 = \log(0.9), \delta_0 = \delta_1 = \delta_2 = \delta_3 = 0.00, \nu = -0.2$	δ_0	**-0.076 (-0.100, -0.053)**	-0.014 (-0.039, 0.010)
$\omega_1 = \omega_2 = \log(0.9), \delta_0 = \delta_1 = \delta_2 = \delta_3 = 0.00, \nu = -0.2$	δ_1	**-0.118 (-0.149, -0.087)**	-0.019 (-0.051, 0.014)
$\omega_1 = \omega_2 = \log(0.9), \delta_0 = \delta_1 = \delta_2 = \delta_3 = 0.00, \nu = -0.2$	δ_2	**-0.176 (-0.217, -0.135)**	-0.037 (-0.079, 0.005)
$\omega_1 = \omega_2 = \log(0.9), \delta_0 = \delta_1 = \delta_2 = \delta_3 = 0.00, \nu = -0.2$	δ_3	**-0.210 (-0.268, -0.152)**	-0.020 (-0.080, 0.040)
$\omega_1 = \omega_2 = \log(0.9), \delta_0 = \delta_1 = \delta_2 = \delta_3 = 0.00, \nu = 0.0$	δ_0	**0.024 (0.001, 0.046)**	0.019 (-0.004, 0.042)
$\omega_1 = \omega_2 = \log(0.9), \delta_0 = \delta_1 = \delta_2 = \delta_3 = 0.00, \nu = 0.0$	δ_1	0.023 (-0.009, 0.055)	0.020 (-0.013, 0.052)
$\omega_1 = \omega_2 = \log(0.9), \delta_0 = \delta_1 = \delta_2 = \delta_3 = 0.00, \nu = 0.0$	δ_2	**0.046 (0.004, 0.088)**	**0.044 (0.002, 0.087)**
$\omega_1 = \omega_2 = \log(0.9), \delta_0 = \delta_1 = \delta_2 = \delta_3 = 0.00, \nu = 0.0$	δ_3	0.045 (-0.015, 0.106)	0.035 (-0.026, 0.097)
$\omega_1 = \omega_2 = \log(0.9), \delta_0 = 0.00, \delta_1 = 2.50, \delta_2 = 5.00, \delta_3 = 6.25, \nu = -0.2$	δ_0	**-0.059 (-0.082, -0.036)**	0.002 (-0.022, 0.025)
$\omega_1 = \omega_2 = \log(0.9), \delta_0 = 0.00, \delta_1 = 2.50, \delta_2 = 5.00, \delta_3 = 6.25, \nu = -0.2$	δ_1	**-0.116 (-0.147, -0.085)**	-0.018 (-0.051, 0.014)
$\omega_1 = \omega_2 = \log(0.9), \delta_0 = 0.00, \delta_1 = 2.50, \delta_2 = 5.00, \delta_3 = 6.25, \nu = -0.2$	δ_2	**-0.154 (-0.195, -0.113)**	-0.017 (-0.060, 0.026)
$\omega_1 = \omega_2 = \log(0.9), \delta_0 = 0.00, \delta_1 = 2.50, \delta_2 = 5.00, \delta_3 = 6.25, \nu = -0.2$	δ_3	**-0.210 (-0.269, -0.151)**	-0.026 (-0.089, 0.036)
$\omega_1 = \omega_2 = \log(0.9), \delta_0 = 0.00, \delta_1 = 2.50, \delta_2 = 5.00, \delta_3 = 6.25, \nu = 0.0$	δ_0	0.009 (-0.014, 0.031)	0.012 (-0.011, 0.035)
$\omega_1 = \omega_2 = \log(0.9), \delta_0 = 0.00, \delta_1 = 2.50, \delta_2 = 5.00, \delta_3 = 6.25, \nu = 0.0$	δ_1	-0.001 (-0.032, 0.029)	-0.001 (-0.032, 0.031)
$\omega_1 = \omega_2 = \log(0.9), \delta_0 = 0.00, \delta_1 = 2.50, \delta_2 = 5.00, \delta_3 = 6.25, \nu = 0.0$	δ_2	-0.025 (-0.067, 0.017)	-0.020 (-0.062, 0.022)
$\omega_1 = \omega_2 = \log(0.9), \delta_0 = 0.00, \delta_1 = 2.50, \delta_2 = 5.00, \delta_3 = 6.25, \nu = 0.0$	δ_3	-0.052 (-0.112, 0.007)	-0.039 (-0.100, 0.022)
$\omega_1 = \omega_2 = \log(0.9), \delta_0 = 0.00, \delta_1 = 12.50, \delta_2 = 25.00, \delta_3 = 31.25, \nu = -0.2$	δ_0	**-0.067 (-0.089, -0.045)**	-0.013 (-0.037, 0.010)
$\omega_1 = \omega_2 = \log(0.9), \delta_0 = 0.00, \delta_1 = 12.50, \delta_2 = 25.00, \delta_3 = 31.25, \nu = -0.2$	δ_1	**-0.118 (-0.150, -0.087)**	-0.018 (-0.051, 0.015)
$\omega_1 = \omega_2 = \log(0.9), \delta_0 = 0.00, \delta_1 = 12.50, \delta_2 = 25.00, \delta_3 = 31.25, \nu = -0.2$	δ_2	**-0.176 (-0.217, -0.135)**	**-0.043 (-0.086, -0.000)**
$\omega_1 = \omega_2 = \log(0.9), \delta_0 = 0.00, \delta_1 = 12.50, \delta_2 = 25.00, \delta_3 = 31.25, \nu = -0.2$	δ_3	**-0.228 (-0.287, -0.168)**	-0.046 (-0.108, 0.016)
$\omega_1 = \omega_2 = \log(0.9), \delta_0 = 0.00, \delta_1 = 12.50, \delta_2 = 25.00, \delta_3 = 31.25, \nu = 0.0$	δ_0	-0.021 (-0.042, 0.001)	**-0.023 (-0.045, -0.000)**
$\omega_1 = \omega_2 = \log(0.9), \delta_0 = 0.00, \delta_1 = 12.50, \delta_2 = 25.00, \delta_3 = 31.25, \nu = 0.0$	δ_1	-0.018 (-0.048, 0.012)	-0.021 (-0.052, 0.009)
$\omega_1 = \omega_2 = \log(0.9), \delta_0 = 0.00, \delta_1 = 12.50, \delta_2 = 25.00, \delta_3 = 31.25, \nu = 0.0$	δ_2	-0.020 (-0.061, 0.021)	-0.024 (-0.065, 0.018)
$\omega_1 = \omega_2 = \log(0.9), \delta_0 = 0.00, \delta_1 = 12.50, \delta_2 = 25.00, \delta_3 = 31.25, \nu = 0.0$	δ_3	0.001 (-0.056, 0.059)	-0.006 (-0.064, 0.053)
$\omega_1 = \omega_2 = \log(1.0), \delta_0 = \delta_1 = \delta_2 = \delta_3 = 0.00, \nu = -0.2$	δ_0	0.011 (-0.012, 0.034)	0.009 (-0.014, 0.032)

Table B13: Bias of treatment effect on the longitudinal outcome for the general time on treatment effect parametrisation, with 95% confidence intervals based on Monte Carlo errors. LMM denotes the linear mixed model, while JM denotes the joint model. Statistically significant biases are highlighted in bold. *(continued)*

Scenario	Parameter	LMM	JM
$\omega_1 = \omega_2 = \log(1.0)$, $\delta_0 = \delta_1 = \delta_2 = \delta_3 = 0.00$, $\nu = -0.2$	δ_1	-0.002 (-0.034, 0.030)	-0.008 (-0.040, 0.025)
$\omega_1 = \omega_2 = \log(1.0)$, $\delta_0 = \delta_1 = \delta_2 = \delta_3 = 0.00$, $\nu = -0.2$	δ_2	-0.004 (-0.047, 0.038)	-0.012 (-0.055, 0.032)
$\omega_1 = \omega_2 = \log(1.0)$, $\delta_0 = \delta_1 = \delta_2 = \delta_3 = 0.00$, $\nu = -0.2$	δ_3	-0.020 (-0.080, 0.040)	-0.027 (-0.088, 0.035)
$\omega_1 = \omega_2 = \log(1.0)$, $\delta_0 = \delta_1 = \delta_2 = \delta_3 = 0.00$, $\nu = 0.0$	δ_0	0.010 (-0.013, 0.032)	0.011 (-0.012, 0.034)
$\omega_1 = \omega_2 = \log(1.0)$, $\delta_0 = \delta_1 = \delta_2 = \delta_3 = 0.00$, $\nu = 0.0$	δ_1	0.000 (-0.031, 0.032)	0.001 (-0.031, 0.032)
$\omega_1 = \omega_2 = \log(1.0)$, $\delta_0 = \delta_1 = \delta_2 = \delta_3 = 0.00$, $\nu = 0.0$	δ_2	0.013 (-0.029, 0.054)	0.012 (-0.030, 0.054)
$\omega_1 = \omega_2 = \log(1.0)$, $\delta_0 = \delta_1 = \delta_2 = \delta_3 = 0.00$, $\nu = 0.0$	δ_3	-0.000 (-0.061, 0.060)	-0.000 (-0.062, 0.062)
$\omega_1 = \omega_2 = \log(1.0)$, $\delta_0 = 0.00, \delta_1 = 2.50, \delta_2 = 5.00, \delta_3 = 6.25$, $\nu = -0.2$	δ_0	0.001 (-0.022, 0.024)	0.002 (-0.021, 0.025)
$\omega_1 = \omega_2 = \log(1.0)$, $\delta_0 = 0.00, \delta_1 = 2.50, \delta_2 = 5.00, \delta_3 = 6.25$, $\nu = -0.2$	δ_1	0.015 (-0.016, 0.047)	0.017 (-0.015, 0.048)
$\omega_1 = \omega_2 = \log(1.0)$, $\delta_0 = 0.00, \delta_1 = 2.50, \delta_2 = 5.00, \delta_3 = 6.25$, $\nu = -0.2$	δ_2	0.017 (-0.025, 0.058)	0.019 (-0.023, 0.060)
$\omega_1 = \omega_2 = \log(1.0)$, $\delta_0 = 0.00, \delta_1 = 2.50, \delta_2 = 5.00, \delta_3 = 6.25$, $\nu = -0.2$	δ_3	0.048 (-0.010, 0.106)	0.050 (-0.009, 0.108)
$\omega_1 = \omega_2 = \log(1.0)$, $\delta_0 = 0.00, \delta_1 = 2.50, \delta_2 = 5.00, \delta_3 = 6.25$, $\nu = 0.0$	δ_0	0.014 (-0.009, 0.036)	0.014 (-0.009, 0.037)
$\omega_1 = \omega_2 = \log(1.0)$, $\delta_0 = 0.00, \delta_1 = 2.50, \delta_2 = 5.00, \delta_3 = 6.25$, $\nu = 0.0$	δ_1	0.014 (-0.018, 0.045)	0.012 (-0.020, 0.044)
$\omega_1 = \omega_2 = \log(1.0)$, $\delta_0 = 0.00, \delta_1 = 2.50, \delta_2 = 5.00, \delta_3 = 6.25$, $\nu = 0.0$	δ_2	0.011 (-0.032, 0.055)	0.010 (-0.034, 0.054)
$\omega_1 = \omega_2 = \log(1.0)$, $\delta_0 = 0.00, \delta_1 = 2.50, \delta_2 = 5.00, \delta_3 = 6.25$, $\nu = 0.0$	δ_3	0.021 (-0.039, 0.080)	0.015 (-0.046, 0.075)
$\omega_1 = \omega_2 = \log(1.0)$, $\delta_0 = 0.00, \delta_1 = 12.50, \delta_2 = 25.00, \delta_3 = 31.25$, $\nu = -0.2$	δ_0	0.017 (-0.006, 0.040)	0.014 (-0.009, 0.038)
$\omega_1 = \omega_2 = \log(1.0)$, $\delta_0 = 0.00, \delta_1 = 12.50, \delta_2 = 25.00, \delta_3 = 31.25$, $\nu = -0.2$	δ_1	0.016 (-0.014, 0.047)	0.013 (-0.018, 0.043)
$\omega_1 = \omega_2 = \log(1.0)$, $\delta_0 = 0.00, \delta_1 = 12.50, \delta_2 = 25.00, \delta_3 = 31.25$, $\nu = -0.2$	δ_2	0.038 (-0.004, 0.079)	0.033 (-0.010, 0.075)
$\omega_1 = \omega_2 = \log(1.0)$, $\delta_0 = 0.00, \delta_1 = 12.50, \delta_2 = 25.00, \delta_3 = 31.25$, $\nu = -0.2$	δ_3	0.051 (-0.006, 0.109)	0.045 (-0.013, 0.103)
$\omega_1 = \omega_2 = \log(1.0)$, $\delta_0 = 0.00, \delta_1 = 12.50, \delta_2 = 25.00, \delta_3 = 31.25$, $\nu = 0.0$	δ_0	-0.006 (-0.030, 0.017)	-0.007 (-0.030, 0.017)
$\omega_1 = \omega_2 = \log(1.0)$, $\delta_0 = 0.00, \delta_1 = 12.50, \delta_2 = 25.00, \delta_3 = 31.25$, $\nu = 0.0$	δ_1	-0.005 (-0.037, 0.026)	-0.002 (-0.034, 0.030)
$\omega_1 = \omega_2 = \log(1.0)$, $\delta_0 = 0.00, \delta_1 = 12.50, \delta_2 = 25.00, \delta_3 = 31.25$, $\nu = 0.0$	δ_2	-0.018 (-0.062, 0.026)	-0.016 (-0.061, 0.028)
$\omega_1 = \omega_2 = \log(1.0)$, $\delta_0 = 0.00, \delta_1 = 12.50, \delta_2 = 25.00, \delta_3 = 31.25$, $\nu = 0.0$	δ_3	-0.036 (-0.095, 0.023)	-0.032 (-0.092, 0.029)
$\omega_1 = \omega_2 = \log(2.0)$, $\delta_0 = \delta_1 = \delta_2 = \delta_3 = 0.00$, $\nu = -0.2$	δ_0	**0.032 (0.010, 0.054)**	0.003 (-0.023, 0.029)
$\omega_1 = \omega_2 = \log(2.0)$, $\delta_0 = \delta_1 = \delta_2 = \delta_3 = 0.00$, $\nu = -0.2$	δ_1	**0.055 (0.027, 0.084)**	0.014 (-0.021, 0.048)

Table B13: Bias of treatment effect on the longitudinal outcome for the general time on treatment effect parametrisation, with 95% confidence intervals based on Monte Carlo errors. LMM denotes the linear mixed model, while JM denotes the joint model. Statistically significant biases are highlighted in bold. *(continued)*

Scenario	Parameter	LMM	JM
$\omega_1 = \omega_2 = \log(2.0)$, $\delta_0 = \delta_1 = \delta_2 = \delta_3 = 0.00$, $\nu = -0.2$	δ_2	**0.053 (0.015, 0.091)**	-0.006 (-0.052, 0.040)
$\omega_1 = \omega_2 = \log(2.0)$, $\delta_0 = \delta_1 = \delta_2 = \delta_3 = 0.00$, $\nu = -0.2$	δ_3	**0.123 (0.072, 0.175)**	0.051 (-0.011, 0.114)
$\omega_1 = \omega_2 = \log(2.0)$, $\delta_0 = \delta_1 = \delta_2 = \delta_3 = 0.00$, $\nu = 0.0$	δ_0	0.005 (-0.018, 0.027)	0.011 (-0.017, 0.038)
$\omega_1 = \omega_2 = \log(2.0)$, $\delta_0 = \delta_1 = \delta_2 = \delta_3 = 0.00$, $\nu = 0.0$	δ_1	0.008 (-0.021, 0.036)	0.031 (-0.005, 0.067)
$\omega_1 = \omega_2 = \log(2.0)$, $\delta_0 = \delta_1 = \delta_2 = \delta_3 = 0.00$, $\nu = 0.0$	δ_2	0.016 (-0.022, 0.054)	**0.054 (0.008, 0.100)**
$\omega_1 = \omega_2 = \log(2.0)$, $\delta_0 = \delta_1 = \delta_2 = \delta_3 = 0.00$, $\nu = 0.0$	δ_3	0.003 (-0.049, 0.054)	-0.011 (-0.074, 0.053)
$\omega_1 = \omega_2 = \log(2.0)$, $\delta_0 = 0.00, \delta_1 = 2.50, \delta_2 = 5.00, \delta_3 = 6.25$, $\nu = -0.2$	δ_0	**0.046 (0.024, 0.068)**	0.019 (-0.008, 0.046)
$\omega_1 = \omega_2 = \log(2.0)$, $\delta_0 = 0.00, \delta_1 = 2.50, \delta_2 = 5.00, \delta_3 = 6.25$, $\nu = -0.2$	δ_1	**0.048 (0.018, 0.078)**	-0.009 (-0.046, 0.028)
$\omega_1 = \omega_2 = \log(2.0)$, $\delta_0 = 0.00, \delta_1 = 2.50, \delta_2 = 5.00, \delta_3 = 6.25$, $\nu = -0.2$	δ_2	**0.071 (0.033, 0.110)**	0.000 (-0.047, 0.048)
$\omega_1 = \omega_2 = \log(2.0)$, $\delta_0 = 0.00, \delta_1 = 2.50, \delta_2 = 5.00, \delta_3 = 6.25$, $\nu = -0.2$	δ_3	**0.150 (0.099, 0.200)**	**0.069 (0.005, 0.134)**
$\omega_1 = \omega_2 = \log(2.0)$, $\delta_0 = 0.00, \delta_1 = 2.50, \delta_2 = 5.00, \delta_3 = 6.25$, $\nu = 0.0$	δ_0	0.013 (-0.009, 0.035)	0.013 (-0.016, 0.042)
$\omega_1 = \omega_2 = \log(2.0)$, $\delta_0 = 0.00, \delta_1 = 2.50, \delta_2 = 5.00, \delta_3 = 6.25$, $\nu = 0.0$	δ_1	0.025 (-0.006, 0.055)	0.027 (-0.010, 0.063)
$\omega_1 = \omega_2 = \log(2.0)$, $\delta_0 = 0.00, \delta_1 = 2.50, \delta_2 = 5.00, \delta_3 = 6.25$, $\nu = 0.0$	δ_2	0.014 (-0.024, 0.053)	0.027 (-0.021, 0.076)
$\omega_1 = \omega_2 = \log(2.0)$, $\delta_0 = 0.00, \delta_1 = 2.50, \delta_2 = 5.00, \delta_3 = 6.25$, $\nu = 0.0$	δ_3	0.040 (-0.015, 0.094)	0.040 (-0.030, 0.110)
$\omega_1 = \omega_2 = \log(2.0)$, $\delta_0 = 0.00, \delta_1 = 12.50, \delta_2 = 25.00, \delta_3 = 31.25$, $\nu = -0.2$	δ_0	**0.045 (0.022, 0.068)**	-0.001 (-0.029, 0.028)
$\omega_1 = \omega_2 = \log(2.0)$, $\delta_0 = 0.00, \delta_1 = 12.50, \delta_2 = 25.00, \delta_3 = 31.25$, $\nu = -0.2$	δ_1	**0.059 (0.030, 0.088)**	-0.024 (-0.060, 0.013)
$\omega_1 = \omega_2 = \log(2.0)$, $\delta_0 = 0.00, \delta_1 = 12.50, \delta_2 = 25.00, \delta_3 = 31.25$, $\nu = -0.2$	δ_2	**0.094 (0.056, 0.132)**	-0.017 (-0.062, 0.028)
$\omega_1 = \omega_2 = \log(2.0)$, $\delta_0 = 0.00, \delta_1 = 12.50, \delta_2 = 25.00, \delta_3 = 31.25$, $\nu = -0.2$	δ_3	**0.112 (0.060, 0.164)**	0.002 (-0.061, 0.066)
$\omega_1 = \omega_2 = \log(2.0)$, $\delta_0 = 0.00, \delta_1 = 12.50, \delta_2 = 25.00, \delta_3 = 31.25$, $\nu = 0.0$	δ_0	0.001 (-0.022, 0.024)	-0.003 (-0.032, 0.027)
$\omega_1 = \omega_2 = \log(2.0)$, $\delta_0 = 0.00, \delta_1 = 12.50, \delta_2 = 25.00, \delta_3 = 31.25$, $\nu = 0.0$	δ_1	-0.014 (-0.045, 0.016)	-0.030 (-0.068, 0.008)
$\omega_1 = \omega_2 = \log(2.0)$, $\delta_0 = 0.00, \delta_1 = 12.50, \delta_2 = 25.00, \delta_3 = 31.25$, $\nu = 0.0$	δ_2	0.004 (-0.035, 0.044)	0.005 (-0.044, 0.053)
$\omega_1 = \omega_2 = \log(2.0)$, $\delta_0 = 0.00, \delta_1 = 12.50, \delta_2 = 25.00, \delta_3 = 31.25$, $\nu = 0.0$	δ_3	0.007 (-0.047, 0.060)	0.019 (-0.050, 0.088)
**Additional scenarios:**			
$i = 3 \times 4$	δ_0	**-0.075 (-0.112, -0.038)**	-0.012 (-0.051, 0.027)
$i = 3 \times 4$	δ_1	**-0.130 (-0.182, -0.078)**	-0.018 (-0.073, 0.037)

Table B13: Bias of treatment effect on the longitudinal outcome for the general time on treatment effect parametrisation, with 95% confidence intervals based on Monte Carlo errors. LMM denotes the linear mixed model, while JM denotes the joint model. Statistically significant biases are highlighted in bold. *(continued)*

Scenario	Parameter	LMM	JM
$i = 3 \times 4$	δ_2	**-0.184 (-0.250, -0.118)**	-0.034 (-0.105, 0.037)
$i = 3 \times 4$	δ_3	**-0.291 (-0.382, -0.200)**	-0.088 (-0.185, 0.009)
$i = 3 \times 4, N = 100$	δ_0	**-0.061 (-0.087, -0.035)**	-0.012 (-0.040, 0.016)
$i = 3 \times 4, N = 100$	δ_1	**-0.085 (-0.121, -0.049)**	0.010 (-0.029, 0.049)
$i = 3 \times 4, N = 100$	δ_2	**-0.125 (-0.172, -0.078)**	0.010 (-0.041, 0.062)
$i = 3 \times 4, N = 100$	δ_3	**-0.179 (-0.244, -0.113)**	0.002 (-0.069, 0.072)
$\sigma^2_\alpha = 1, \sigma^2_\phi = 27.5$	δ_0	**-0.054 (-0.076, -0.032)**	-0.003 (-0.026, 0.020)
$\sigma^2_\alpha = 1, \sigma^2_\phi = 27.5$	δ_1	**-0.087 (-0.117, -0.057)**	0.002 (-0.028, 0.033)
$\sigma^2_\alpha = 1, \sigma^2_\phi = 27.5$	δ_2	**-0.148 (-0.189, -0.108)**	-0.016 (-0.057, 0.025)
$\sigma^2_\alpha = 1, \sigma^2_\phi = 27.5$	δ_3	**-0.209 (-0.265, -0.154)**	-0.037 (-0.092, 0.019)
$\sigma^2_\alpha = 4, \sigma^2_\phi = 110$	δ_0	**-0.058 (-0.080, -0.035)**	0.002 (-0.023, 0.026)
$\sigma^2_\alpha = 4, \sigma^2_\phi = 110$	δ_1	**-0.093 (-0.125, -0.061)**	0.002 (-0.032, 0.036)
$\sigma^2_\alpha = 4, \sigma^2_\phi = 110$	δ_2	**-0.137 (-0.179, -0.095)**	0.001 (-0.043, 0.046)
$\sigma^2_\alpha = 4, \sigma^2_\phi = 110$	δ_3	**-0.176 (-0.236, -0.116)**	-0.000 (-0.063, 0.062)

Table B14: Relative bias of treatment effect on the longitudinal outcome for the general time on treatment effect parametrisation, with 95% confidence intervals based on Monte Carlo errors. LMM denotes the linear mixed model, while JM denotes the joint model. Statistically significant biases are highlighted in bold.

Scenario	Parameter	LMM	JM
**Main scenarios:**			
$\omega_1 = \omega_2 = \log(0.5)$, $\delta_0 = \delta_1 = \delta_2 = \delta_3 = 0.00$, $\nu = -0.2$	δ_0	—	—
$\omega_1 = \omega_2 = \log(0.5)$, $\delta_0 = \delta_1 = \delta_2 = \delta_3 = 0.00$, $\nu = -0.2$	δ_1	—	—
$\omega_1 = \omega_2 = \log(0.5)$, $\delta_0 = \delta_1 = \delta_2 = \delta_3 = 0.00$, $\nu = -0.2$	δ_2	—	—
$\omega_1 = \omega_2 = \log(0.5)$, $\delta_0 = \delta_1 = \delta_2 = \delta_3 = 0.00$, $\nu = -0.2$	δ_3	—	—
$\omega_1 = \omega_2 = \log(0.5)$, $\delta_0 = \delta_1 = \delta_2 = \delta_3 = 0.00$, $\nu = 0.0$	δ_0	—	—
$\omega_1 = \omega_2 = \log(0.5)$, $\delta_0 = \delta_1 = \delta_2 = \delta_3 = 0.00$, $\nu = 0.0$	δ_1	—	—
$\omega_1 = \omega_2 = \log(0.5)$, $\delta_0 = \delta_1 = \delta_2 = \delta_3 = 0.00$, $\nu = 0.0$	δ_2	—	—
$\omega_1 = \omega_2 = \log(0.5)$, $\delta_0 = \delta_1 = \delta_2 = \delta_3 = 0.00$, $\nu = 0.0$	δ_3	—	—
$\omega_1 = \omega_2 = \log(0.5)$, $\delta_0 = 0.00, \delta_1 = 2.50, \delta_2 = 5.00, \delta_3 = 6.25$, $\nu = -0.2$	δ_0	—	—
$\omega_1 = \omega_2 = \log(0.5)$, $\delta_0 = 0.00, \delta_1 = 2.50, \delta_2 = 5.00, \delta_3 = 6.25$, $\nu = -0.2$	δ_1	**-0.014 (-0.026, -0.003)**	0.006 (-0.008, 0.020)
$\omega_1 = \omega_2 = \log(0.5)$, $\delta_0 = 0.00, \delta_1 = 2.50, \delta_2 = 5.00, \delta_3 = 6.25$, $\nu = -0.2$	δ_2	**-0.013 (-0.020, -0.005)**	-0.001 (-0.009, 0.008)
$\omega_1 = \omega_2 = \log(0.5)$, $\delta_0 = 0.00, \delta_1 = 2.50, \delta_2 = 5.00, \delta_3 = 6.25$, $\nu = -0.2$	δ_3	**-0.011 (-0.019, -0.003)**	0.002 (-0.008, 0.012)
$\omega_1 = \omega_2 = \log(0.5)$, $\delta_0 = 0.00, \delta_1 = 2.50, \delta_2 = 5.00, \delta_3 = 6.25$, $\nu = 0.0$	δ_0	—	—
$\omega_1 = \omega_2 = \log(0.5)$, $\delta_0 = 0.00, \delta_1 = 2.50, \delta_2 = 5.00, \delta_3 = 6.25$, $\nu = 0.0$	δ_1	-0.001 (-0.013, 0.011)	-0.006 (-0.019, 0.008)
$\omega_1 = \omega_2 = \log(0.5)$, $\delta_0 = 0.00, \delta_1 = 2.50, \delta_2 = 5.00, \delta_3 = 6.25$, $\nu = 0.0$	δ_2	-0.000 (-0.008, 0.008)	-0.001 (-0.010, 0.008)
$\omega_1 = \omega_2 = \log(0.5)$, $\delta_0 = 0.00, \delta_1 = 2.50, \delta_2 = 5.00, \delta_3 = 6.25$, $\nu = 0.0$	δ_3	-0.002 (-0.011, 0.006)	-0.001 (-0.011, 0.008)
$\omega_1 = \omega_2 = \log(0.5)$, $\delta_0 = 0.00, \delta_1 = 12.50, \delta_2 = 25.00, \delta_3 = 31.25$, $\nu = -0.2$	δ_0	—	—
$\omega_1 = \omega_2 = \log(0.5)$, $\delta_0 = 0.00, \delta_1 = 12.50, \delta_2 = 25.00, \delta_3 = 31.25$, $\nu = -0.2$	δ_1	**-0.004 (-0.007, -0.002)**	-0.000 (-0.003, 0.003)
$\omega_1 = \omega_2 = \log(0.5)$, $\delta_0 = 0.00, \delta_1 = 12.50, \delta_2 = 25.00, \delta_3 = 31.25$, $\nu = -0.2$	δ_2	**-0.003 (-0.005, -0.002)**	-0.000 (-0.002, 0.002)
$\omega_1 = \omega_2 = \log(0.5)$, $\delta_0 = 0.00, \delta_1 = 12.50, \delta_2 = 25.00, \delta_3 = 31.25$, $\nu = -0.2$	δ_3	**-0.003 (-0.004, -0.001)**	0.001 (-0.001, 0.003)
$\omega_1 = \omega_2 = \log(0.5)$, $\delta_0 = 0.00, \delta_1 = 12.50, \delta_2 = 25.00, \delta_3 = 31.25$, $\nu = 0.0$	δ_0	—	—
$\omega_1 = \omega_2 = \log(0.5)$, $\delta_0 = 0.00, \delta_1 = 12.50, \delta_2 = 25.00, \delta_3 = 31.25$, $\nu = 0.0$	δ_1	0.000 (-0.002, 0.003)	0.000 (-0.002, 0.003)
$\omega_1 = \omega_2 = \log(0.5)$, $\delta_0 = 0.00, \delta_1 = 12.50, \delta_2 = 25.00, \delta_3 = 31.25$, $\nu = 0.0$	δ_2	-0.000 (-0.002, 0.002)	0.000 (-0.002, 0.002)
$\omega_1 = \omega_2 = \log(0.5)$, $\delta_0 = 0.00, \delta_1 = 12.50, \delta_2 = 25.00, \delta_3 = 31.25$, $\nu = 0.0$	δ_3	0.000 (-0.002, 0.002)	-0.001 (-0.003, 0.001)

Table B14: Relative bias of treatment effect on the longitudinal outcome for the general time on treatment effect parametrisation, with 95% confidence intervals based on Monte Carlo errors. LMM denotes the linear mixed model, while JM denotes the joint model. Statistically significant biases are highlighted in bold. *(continued)*

Scenario	Parameter	LMM	JM
$\omega_1 = \omega_2 = \log(0.9)$, $\delta_0 = \delta_1 = \delta_2 = \delta_3 = 0.00$, $\nu = -0.2$	δ_0	—	—
$\omega_1 = \omega_2 = \log(0.9)$, $\delta_0 = \delta_1 = \delta_2 = \delta_3 = 0.00$, $\nu = -0.2$	δ_1	—	—
$\omega_1 = \omega_2 = \log(0.9)$, $\delta_0 = \delta_1 = \delta_2 = \delta_3 = 0.00$, $\nu = -0.2$	δ_2	—	—
$\omega_1 = \omega_2 = \log(0.9)$, $\delta_0 = \delta_1 = \delta_2 = \delta_3 = 0.00$, $\nu = -0.2$	δ_3	—	—
$\omega_1 = \omega_2 = \log(0.9)$, $\delta_0 = \delta_1 = \delta_2 = \delta_3 = 0.00$, $\nu = 0.0$	δ_0	—	—
$\omega_1 = \omega_2 = \log(0.9)$, $\delta_0 = \delta_1 = \delta_2 = \delta_3 = 0.00$, $\nu = 0.0$	δ_1	—	—
$\omega_1 = \omega_2 = \log(0.9)$, $\delta_0 = \delta_1 = \delta_2 = \delta_3 = 0.00$, $\nu = 0.0$	δ_2	—	—
$\omega_1 = \omega_2 = \log(0.9)$, $\delta_0 = \delta_1 = \delta_2 = \delta_3 = 0.00$, $\nu = 0.0$	δ_3	—	—
$\omega_1 = \omega_2 = \log(0.9)$, $\delta_0 = 0.00, \delta_1 = 2.50, \delta_2 = 5.00, \delta_3 = 6.25$, $\nu = -0.2$	δ_0	—	—
$\omega_1 = \omega_2 = \log(0.9)$, $\delta_0 = 0.00, \delta_1 = 2.50, \delta_2 = 5.00, \delta_3 = 6.25$, $\nu = -0.2$	δ_1	**-0.046 (-0.059, -0.034)**	-0.007 (-0.020, 0.006)
$\omega_1 = \omega_2 = \log(0.9)$, $\delta_0 = 0.00, \delta_1 = 2.50, \delta_2 = 5.00, \delta_3 = 6.25$, $\nu = -0.2$	δ_2	**-0.031 (-0.039, -0.023)**	-0.003 (-0.012, 0.005)
$\omega_1 = \omega_2 = \log(0.9)$, $\delta_0 = 0.00, \delta_1 = 2.50, \delta_2 = 5.00, \delta_3 = 6.25$, $\nu = -0.2$	δ_3	**-0.034 (-0.043, -0.024)**	-0.004 (-0.014, 0.006)
$\omega_1 = \omega_2 = \log(0.9)$, $\delta_0 = 0.00, \delta_1 = 2.50, \delta_2 = 5.00, \delta_3 = 6.25$, $\nu = 0.0$	δ_0	—	—
$\omega_1 = \omega_2 = \log(0.9)$, $\delta_0 = 0.00, \delta_1 = 2.50, \delta_2 = 5.00, \delta_3 = 6.25$, $\nu = 0.0$	δ_1	-0.001 (-0.013, 0.012)	-0.000 (-0.013, 0.012)
$\omega_1 = \omega_2 = \log(0.9)$, $\delta_0 = 0.00, \delta_1 = 2.50, \delta_2 = 5.00, \delta_3 = 6.25$, $\nu = 0.0$	δ_2	-0.005 (-0.013, 0.003)	-0.004 (-0.012, 0.004)
$\omega_1 = \omega_2 = \log(0.9)$, $\delta_0 = 0.00, \delta_1 = 2.50, \delta_2 = 5.00, \delta_3 = 6.25$, $\nu = 0.0$	δ_3	-0.008 (-0.018, 0.001)	-0.006 (-0.016, 0.003)
$\omega_1 = \omega_2 = \log(0.9)$, $\delta_0 = 0.00, \delta_1 = 12.50, \delta_2 = 25.00, \delta_3 = 31.25$, $\nu = -0.2$	δ_0	—	—
$\omega_1 = \omega_2 = \log(0.9)$, $\delta_0 = 0.00, \delta_1 = 12.50, \delta_2 = 25.00, \delta_3 = 31.25$, $\nu = -0.2$	δ_1	**-0.009 (-0.012, -0.007)**	-0.001 (-0.004, 0.001)
$\omega_1 = \omega_2 = \log(0.9)$, $\delta_0 = 0.00, \delta_1 = 12.50, \delta_2 = 25.00, \delta_3 = 31.25$, $\nu = -0.2$	δ_2	**-0.007 (-0.009, -0.005)**	**-0.002 (-0.003, -0.000)**
$\omega_1 = \omega_2 = \log(0.9)$, $\delta_0 = 0.00, \delta_1 = 12.50, \delta_2 = 25.00, \delta_3 = 31.25$, $\nu = -0.2$	δ_3	**-0.007 (-0.009, -0.005)**	-0.001 (-0.003, 0.001)
$\omega_1 = \omega_2 = \log(0.9)$, $\delta_0 = 0.00, \delta_1 = 12.50, \delta_2 = 25.00, \delta_3 = 31.25$, $\nu = 0.0$	δ_0	—	—
$\omega_1 = \omega_2 = \log(0.9)$, $\delta_0 = 0.00, \delta_1 = 12.50, \delta_2 = 25.00, \delta_3 = 31.25$, $\nu = 0.0$	δ_1	-0.001 (-0.004, 0.001)	-0.002 (-0.004, 0.001)
$\omega_1 = \omega_2 = \log(0.9)$, $\delta_0 = 0.00, \delta_1 = 12.50, \delta_2 = 25.00, \delta_3 = 31.25$, $\nu = 0.0$	δ_2	-0.001 (-0.002, 0.001)	-0.001 (-0.003, 0.001)
$\omega_1 = \omega_2 = \log(0.9)$, $\delta_0 = 0.00, \delta_1 = 12.50, \delta_2 = 25.00, \delta_3 = 31.25$, $\nu = 0.0$	δ_3	0.000 (-0.002, 0.002)	-0.000 (-0.002, 0.002)
$\omega_1 = \omega_2 = \log(1.0)$, $\delta_0 = \delta_1 = \delta_2 = \delta_3 = 0.00$, $\nu = -0.2$	δ_0	—	—

Table B14: Relative bias of treatment effect on the longitudinal outcome for the general time on treatment effect parametrisation, with 95% confidence intervals based on Monte Carlo errors. LMM denotes the linear mixed model, while JM denotes the joint model. Statistically significant biases are highlighted in bold. *(continued)*

Scenario	Parameter	LMM	JM
$\omega_1 = \omega_2 = \log(1.0)$, $\delta_0 = \delta_1 = \delta_2 = \delta_3 = 0.00$, $\nu = -0.2$	δ_1	—	—
$\omega_1 = \omega_2 = \log(1.0)$, $\delta_0 = \delta_1 = \delta_2 = \delta_3 = 0.00$, $\nu = -0.2$	δ_2	—	—
$\omega_1 = \omega_2 = \log(1.0)$, $\delta_0 = \delta_1 = \delta_2 = \delta_3 = 0.00$, $\nu = -0.2$	δ_3	—	—
$\omega_1 = \omega_2 = \log(1.0)$, $\delta_0 = \delta_1 = \delta_2 = \delta_3 = 0.00$, $\nu = 0.0$	δ_0	—	—
$\omega_1 = \omega_2 = \log(1.0)$, $\delta_0 = \delta_1 = \delta_2 = \delta_3 = 0.00$, $\nu = 0.0$	δ_1	—	—
$\omega_1 = \omega_2 = \log(1.0)$, $\delta_0 = \delta_1 = \delta_2 = \delta_3 = 0.00$, $\nu = 0.0$	δ_2	—	—
$\omega_1 = \omega_2 = \log(1.0)$, $\delta_0 = \delta_1 = \delta_2 = \delta_3 = 0.00$, $\nu = 0.0$	δ_3	—	—
$\omega_1 = \omega_2 = \log(1.0)$, $\delta_0 = 0.00, \delta_1 = 2.50, \delta_2 = 5.00, \delta_3 = 6.25$, $\nu = -0.2$	δ_0	—	—
$\omega_1 = \omega_2 = \log(1.0)$, $\delta_0 = 0.00, \delta_1 = 2.50, \delta_2 = 5.00, \delta_3 = 6.25$, $\nu = -0.2$	δ_1	0.006 (-0.006, 0.019)	0.007 (-0.006, 0.019)
$\omega_1 = \omega_2 = \log(1.0)$, $\delta_0 = 0.00, \delta_1 = 2.50, \delta_2 = 5.00, \delta_3 = 6.25$, $\nu = -0.2$	δ_2	0.003 (-0.005, 0.012)	0.004 (-0.005, 0.012)
$\omega_1 = \omega_2 = \log(1.0)$, $\delta_0 = 0.00, \delta_1 = 2.50, \delta_2 = 5.00, \delta_3 = 6.25$, $\nu = -0.2$	δ_3	0.008 (-0.002, 0.017)	0.008 (-0.001, 0.017)
$\omega_1 = \omega_2 = \log(1.0)$, $\delta_0 = 0.00, \delta_1 = 2.50, \delta_2 = 5.00, \delta_3 = 6.25$, $\nu = 0.0$	δ_0	—	—
$\omega_1 = \omega_2 = \log(1.0)$, $\delta_0 = 0.00, \delta_1 = 2.50, \delta_2 = 5.00, \delta_3 = 6.25$, $\nu = 0.0$	δ_1	0.005 (-0.007, 0.018)	0.005 (-0.008, 0.018)
$\omega_1 = \omega_2 = \log(1.0)$, $\delta_0 = 0.00, \delta_1 = 2.50, \delta_2 = 5.00, \delta_3 = 6.25$, $\nu = 0.0$	δ_2	0.002 (-0.006, 0.011)	0.002 (-0.007, 0.011)
$\omega_1 = \omega_2 = \log(1.0)$, $\delta_0 = 0.00, \delta_1 = 2.50, \delta_2 = 5.00, \delta_3 = 6.25$, $\nu = 0.0$	δ_3	0.003 (-0.006, 0.013)	0.002 (-0.007, 0.012)
$\omega_1 = \omega_2 = \log(1.0)$, $\delta_0 = 0.00, \delta_1 = 12.50, \delta_2 = 25.00, \delta_3 = 31.25$, $\nu = -0.2$	δ_0	—	—
$\omega_1 = \omega_2 = \log(1.0)$, $\delta_0 = 0.00, \delta_1 = 12.50, \delta_2 = 25.00, \delta_3 = 31.25$, $\nu = -0.2$	δ_1	0.001 (-0.001, 0.004)	0.001 (-0.001, 0.003)
$\omega_1 = \omega_2 = \log(1.0)$, $\delta_0 = 0.00, \delta_1 = 12.50, \delta_2 = 25.00, \delta_3 = 31.25$, $\nu = -0.2$	δ_2	0.002 (-0.000, 0.003)	0.001 (-0.000, 0.003)
$\omega_1 = \omega_2 = \log(1.0)$, $\delta_0 = 0.00, \delta_1 = 12.50, \delta_2 = 25.00, \delta_3 = 31.25$, $\nu = -0.2$	δ_3	0.002 (-0.000, 0.003)	0.001 (-0.000, 0.003)
$\omega_1 = \omega_2 = \log(1.0)$, $\delta_0 = 0.00, \delta_1 = 12.50, \delta_2 = 25.00, \delta_3 = 31.25$, $\nu = 0.0$	δ_0	—	—
$\omega_1 = \omega_2 = \log(1.0)$, $\delta_0 = 0.00, \delta_1 = 12.50, \delta_2 = 25.00, \delta_3 = 31.25$, $\nu = 0.0$	δ_1	-0.000 (-0.003, 0.002)	-0.000 (-0.003, 0.002)
$\omega_1 = \omega_2 = \log(1.0)$, $\delta_0 = 0.00, \delta_1 = 12.50, \delta_2 = 25.00, \delta_3 = 31.25$, $\nu = 0.0$	δ_2	-0.001 (-0.002, 0.001)	-0.001 (-0.002, 0.001)
$\omega_1 = \omega_2 = \log(1.0)$, $\delta_0 = 0.00, \delta_1 = 12.50, \delta_2 = 25.00, \delta_3 = 31.25$, $\nu = 0.0$	δ_3	-0.001 (-0.003, 0.001)	-0.001 (-0.003, 0.001)
$\omega_1 = \omega_2 = \log(2.0)$, $\delta_0 = \delta_1 = \delta_2 = \delta_3 = 0.00$, $\nu = -0.2$	δ_0	—	—
$\omega_1 = \omega_2 = \log(2.0)$, $\delta_0 = \delta_1 = \delta_2 = \delta_3 = 0.00$, $\nu = -0.2$	δ_1	—	—

Table B14: Relative bias of treatment effect on the longitudinal outcome for the general time on treatment effect parametrisation, with 95% confidence intervals based on Monte Carlo errors. LMM denotes the linear mixed model, while JM denotes the joint model. Statistically significant biases are highlighted in bold. *(continued)*

Scenario	Parameter	LMM	JM
$\omega_1 = \omega_2 = \log(2.0)$, $\delta_0 = \delta_1 = \delta_2 = \delta_3 = 0.00$, $\nu = -0.2$	δ_2	—	—
$\omega_1 = \omega_2 = \log(2.0)$, $\delta_0 = \delta_1 = \delta_2 = \delta_3 = 0.00$, $\nu = -0.2$	δ_3	—	—
$\omega_1 = \omega_2 = \log(2.0)$, $\delta_0 = \delta_1 = \delta_2 = \delta_3 = 0.00$, $\nu = 0.0$	δ_0	—	—
$\omega_1 = \omega_2 = \log(2.0)$, $\delta_0 = \delta_1 = \delta_2 = \delta_3 = 0.00$, $\nu = 0.0$	δ_1	—	—
$\omega_1 = \omega_2 = \log(2.0)$, $\delta_0 = \delta_1 = \delta_2 = \delta_3 = 0.00$, $\nu = 0.0$	δ_2	—	—
$\omega_1 = \omega_2 = \log(2.0)$, $\delta_0 = \delta_1 = \delta_2 = \delta_3 = 0.00$, $\nu = 0.0$	δ_3	—	—
$\omega_1 = \omega_2 = \log(2.0)$, $\delta_0 = 0.00$, $\delta_1 = 2.50$, $\delta_2 = 5.00$, $\delta_3 = 6.25$, $\nu = -0.2$	δ_0	—	—
$\omega_1 = \omega_2 = \log(2.0)$, $\delta_0 = 0.00$, $\delta_1 = 2.50$, $\delta_2 = 5.00$, $\delta_3 = 6.25$, $\nu = -0.2$	δ_1	**0.019 (0.007, 0.031)**	-0.004 (-0.018, 0.011)
$\omega_1 = \omega_2 = \log(2.0)$, $\delta_0 = 0.00$, $\delta_1 = 2.50$, $\delta_2 = 5.00$, $\delta_3 = 6.25$, $\nu = -0.2$	δ_2	**0.014 (0.007, 0.022)**	0.000 (-0.009, 0.010)
$\omega_1 = \omega_2 = \log(2.0)$, $\delta_0 = 0.00$, $\delta_1 = 2.50$, $\delta_2 = 5.00$, $\delta_3 = 6.25$, $\nu = -0.2$	δ_3	**0.024 (0.016, 0.032)**	**0.011 (0.001, 0.021)**
$\omega_1 = \omega_2 = \log(2.0)$, $\delta_0 = 0.00$, $\delta_1 = 2.50$, $\delta_2 = 5.00$, $\delta_3 = 6.25$, $\nu = 0.0$	δ_0	—	—
$\omega_1 = \omega_2 = \log(2.0)$, $\delta_0 = 0.00$, $\delta_1 = 2.50$, $\delta_2 = 5.00$, $\delta_3 = 6.25$, $\nu = 0.0$	δ_1	0.010 (-0.002, 0.022)	0.011 (-0.004, 0.025)
$\omega_1 = \omega_2 = \log(2.0)$, $\delta_0 = 0.00$, $\delta_1 = 2.50$, $\delta_2 = 5.00$, $\delta_3 = 6.25$, $\nu = 0.0$	δ_2	0.003 (-0.005, 0.011)	0.005 (-0.004, 0.015)
$\omega_1 = \omega_2 = \log(2.0)$, $\delta_0 = 0.00$, $\delta_1 = 2.50$, $\delta_2 = 5.00$, $\delta_3 = 6.25$, $\nu = 0.0$	δ_3	0.006 (-0.002, 0.015)	0.006 (-0.005, 0.018)
$\omega_1 = \omega_2 = \log(2.0)$, $\delta_0 = 0.00$, $\delta_1 = 12.50$, $\delta_2 = 25.00$, $\delta_3 = 31.25$, $\nu = -0.2$	δ_0	—	—
$\omega_1 = \omega_2 = \log(2.0)$, $\delta_0 = 0.00$, $\delta_1 = 12.50$, $\delta_2 = 25.00$, $\delta_3 = 31.25$, $\nu = -0.2$	δ_1	**0.005 (0.002, 0.007)**	-0.002 (-0.005, 0.001)
$\omega_1 = \omega_2 = \log(2.0)$, $\delta_0 = 0.00$, $\delta_1 = 12.50$, $\delta_2 = 25.00$, $\delta_3 = 31.25$, $\nu = -0.2$	δ_2	**0.004 (0.002, 0.005)**	-0.001 (-0.002, 0.001)
$\omega_1 = \omega_2 = \log(2.0)$, $\delta_0 = 0.00$, $\delta_1 = 12.50$, $\delta_2 = 25.00$, $\delta_3 = 31.25$, $\nu = -0.2$	δ_3	**0.004 (0.002, 0.005)**	0.000 (-0.002, 0.002)
$\omega_1 = \omega_2 = \log(2.0)$, $\delta_0 = 0.00$, $\delta_1 = 12.50$, $\delta_2 = 25.00$, $\delta_3 = 31.25$, $\nu = 0.0$	δ_0	—	—
$\omega_1 = \omega_2 = \log(2.0)$, $\delta_0 = 0.00$, $\delta_1 = 12.50$, $\delta_2 = 25.00$, $\delta_3 = 31.25$, $\nu = 0.0$	δ_1	-0.001 (-0.004, 0.001)	-0.002 (-0.005, 0.001)
$\omega_1 = \omega_2 = \log(2.0)$, $\delta_0 = 0.00$, $\delta_1 = 12.50$, $\delta_2 = 25.00$, $\delta_3 = 31.25$, $\nu = 0.0$	δ_2	0.000 (-0.001, 0.002)	0.000 (-0.002, 0.002)
$\omega_1 = \omega_2 = \log(2.0)$, $\delta_0 = 0.00$, $\delta_1 = 12.50$, $\delta_2 = 25.00$, $\delta_3 = 31.25$, $\nu = 0.0$	δ_3	0.000 (-0.002, 0.002)	0.001 (-0.002, 0.003)
**Additional scenarios:**			
$i = 3 \times 4$	δ_0	—	—
$i = 3 \times 4$	δ_1	**-0.052 (-0.073, -0.031)**	-0.007 (-0.029, 0.015)

Table B14: Relative bias of treatment effect on the longitudinal outcome for the general time on treatment effect parametrisation, with 95% confidence intervals based on Monte Carlo errors. LMM denotes the linear mixed model, while JM denotes the joint model. Statistically significant biases are highlighted in bold. *(continued)*

Scenario	Parameter	LMM	JM
$i = 3 \times 4$	δ_2	**-0.037 (-0.050, -0.024)**	-0.007 (-0.021, 0.007)
$i = 3 \times 4$	δ_3	**-0.047 (-0.061, -0.032)**	-0.014 (-0.030, 0.001)
$i = 3 \times 4, N = 100$	δ_0	—	—
$i = 3 \times 4, N = 100$	δ_1	**-0.034 (-0.048, -0.019)**	0.004 (-0.012, 0.019)
$i = 3 \times 4, N = 100$	δ_2	**-0.025 (-0.034, -0.016)**	0.002 (-0.008, 0.012)
$i = 3 \times 4, N = 100$	δ_3	**-0.029 (-0.039, -0.018)**	0.000 (-0.011, 0.012)
$\sigma^2_\alpha = 1, \sigma^2_\phi = 27.5$	δ_0	—	—
$\sigma^2_\alpha = 1, \sigma^2_\phi = 27.5$	δ_1	**-0.035 (-0.047, -0.023)**	0.001 (-0.011, 0.013)
$\sigma^2_\alpha = 1, \sigma^2_\phi = 27.5$	δ_2	**-0.030 (-0.038, -0.022)**	-0.003 (-0.011, 0.005)
$\sigma^2_\alpha = 1, \sigma^2_\phi = 27.5$	δ_3	**-0.034 (-0.042, -0.025)**	-0.006 (-0.015, 0.003)
$\sigma^2_\alpha = 4, \sigma^2_\phi = 110$	δ_0	—	—
$\sigma^2_\alpha = 4, \sigma^2_\phi = 110$	δ_1	**-0.037 (-0.050, -0.024)**	0.001 (-0.013, 0.015)
$\sigma^2_\alpha = 4, \sigma^2_\phi = 110$	δ_2	**-0.027 (-0.036, -0.019)**	0.000 (-0.009, 0.009)
$\sigma^2_\alpha = 4, \sigma^2_\phi = 110$	δ_3	**-0.028 (-0.038, -0.019)**	-0.000 (-0.010, 0.010)

Table B15: Coverage probability of treatment effect on the longitudinal outcome for the general time on treatment effect parametrisation, with 95% confidence intervals based on Monte Carlo errors. LMM denotes the linear mixed model, while JM denotes the joint model.

Scenario	Parameter	LMM	JM
**Main scenarios:**			
$\omega_1 = \omega_2 = \log(0.5)$, $\delta_0 = \delta_1 = \delta_2 = \delta_3 = 0.00$, $\nu = -0.2$	δ_0	0.952 (0.939, 0.965)	0.958 (0.943, 0.973)
$\omega_1 = \omega_2 = \log(0.5)$, $\delta_0 = \delta_1 = \delta_2 = \delta_3 = 0.00$, $\nu = -0.2$	δ_1	0.949 (0.935, 0.962)	0.949 (0.932, 0.966)
$\omega_1 = \omega_2 = \log(0.5)$, $\delta_0 = \delta_1 = \delta_2 = \delta_3 = 0.00$, $\nu = -0.2$	δ_2	0.954 (0.941, 0.967)	0.961 (0.946, 0.976)
$\omega_1 = \omega_2 = \log(0.5)$, $\delta_0 = \delta_1 = \delta_2 = \delta_3 = 0.00$, $\nu = -0.2$	δ_3	0.962 (0.950, 0.973)	0.953 (0.937, 0.969)
$\omega_1 = \omega_2 = \log(0.5)$, $\delta_0 = \delta_1 = \delta_2 = \delta_3 = 0.00$, $\nu = 0.0$	δ_0	0.964 (0.953, 0.976)	0.969 (0.956, 0.982)
$\omega_1 = \omega_2 = \log(0.5)$, $\delta_0 = \delta_1 = \delta_2 = \delta_3 = 0.00$, $\nu = 0.0$	δ_1	0.960 (0.948, 0.972)	0.965 (0.951, 0.979)
$\omega_1 = \omega_2 = \log(0.5)$, $\delta_0 = \delta_1 = \delta_2 = \delta_3 = 0.00$, $\nu = 0.0$	δ_2	0.956 (0.944, 0.969)	0.951 (0.935, 0.968)
$\omega_1 = \omega_2 = \log(0.5)$, $\delta_0 = \delta_1 = \delta_2 = \delta_3 = 0.00$, $\nu = 0.0$	δ_3	0.956 (0.944, 0.969)	0.960 (0.946, 0.975)
$\omega_1 = \omega_2 = \log(0.5)$, $\delta_0 = 0.00, \delta_1 = 2.50, \delta_2 = 5.00, \delta_3 = 6.25$, $\nu = -0.2$	δ_0	0.959 (0.946, 0.971)	0.961 (0.946, 0.976)
$\omega_1 = \omega_2 = \log(0.5)$, $\delta_0 = 0.00, \delta_1 = 2.50, \delta_2 = 5.00, \delta_3 = 6.25$, $\nu = -0.2$	δ_1	0.952 (0.939, 0.965)	0.949 (0.933, 0.966)
$\omega_1 = \omega_2 = \log(0.5)$, $\delta_0 = 0.00, \delta_1 = 2.50, \delta_2 = 5.00, \delta_3 = 6.25$, $\nu = -0.2$	δ_2	0.949 (0.935, 0.962)	0.960 (0.945, 0.975)
$\omega_1 = \omega_2 = \log(0.5)$, $\delta_0 = 0.00, \delta_1 = 2.50, \delta_2 = 5.00, \delta_3 = 6.25$, $\nu = -0.2$	δ_3	0.955 (0.942, 0.968)	0.952 (0.936, 0.968)
$\omega_1 = \omega_2 = \log(0.5)$, $\delta_0 = 0.00, \delta_1 = 2.50, \delta_2 = 5.00, \delta_3 = 6.25$, $\nu = 0.0$	δ_0	0.929 (0.913, 0.945)	0.932 (0.913, 0.952)
$\omega_1 = \omega_2 = \log(0.5)$, $\delta_0 = 0.00, \delta_1 = 2.50, \delta_2 = 5.00, \delta_3 = 6.25$, $\nu = 0.0$	δ_1	0.946 (0.932, 0.960)	0.954 (0.938, 0.970)
$\omega_1 = \omega_2 = \log(0.5)$, $\delta_0 = 0.00, \delta_1 = 2.50, \delta_2 = 5.00, \delta_3 = 6.25$, $\nu = 0.0$	δ_2	0.945 (0.931, 0.959)	0.957 (0.941, 0.972)
$\omega_1 = \omega_2 = \log(0.5)$, $\delta_0 = 0.00, \delta_1 = 2.50, \delta_2 = 5.00, \delta_3 = 6.25$, $\nu = 0.0$	δ_3	0.947 (0.933, 0.960)	0.961 (0.947, 0.976)
$\omega_1 = \omega_2 = \log(0.5)$, $\delta_0 = 0.00, \delta_1 = 12.50, \delta_2 = 25.00, \delta_3 = 31.25$, $\nu = -0.2$	δ_0	0.939 (0.924, 0.954)	0.941 (0.923, 0.959)
$\omega_1 = \omega_2 = \log(0.5)$, $\delta_0 = 0.00, \delta_1 = 12.50, \delta_2 = 25.00, \delta_3 = 31.25$, $\nu = -0.2$	δ_1	0.945 (0.931, 0.959)	0.934 (0.915, 0.953)
$\omega_1 = \omega_2 = \log(0.5)$, $\delta_0 = 0.00, \delta_1 = 12.50, \delta_2 = 25.00, \delta_3 = 31.25$, $\nu = -0.2$	δ_2	0.951 (0.937, 0.964)	0.946 (0.928, 0.963)
$\omega_1 = \omega_2 = \log(0.5)$, $\delta_0 = 0.00, \delta_1 = 12.50, \delta_2 = 25.00, \delta_3 = 31.25$, $\nu = -0.2$	δ_3	0.955 (0.942, 0.968)	0.950 (0.934, 0.967)
$\omega_1 = \omega_2 = \log(0.5)$, $\delta_0 = 0.00, \delta_1 = 12.50, \delta_2 = 25.00, \delta_3 = 31.25$, $\nu = 0.0$	δ_0	0.942 (0.928, 0.957)	0.948 (0.931, 0.965)
$\omega_1 = \omega_2 = \log(0.5)$, $\delta_0 = 0.00, \delta_1 = 12.50, \delta_2 = 25.00, \delta_3 = 31.25$, $\nu = 0.0$	δ_1	0.945 (0.931, 0.959)	0.958 (0.942, 0.973)
$\omega_1 = \omega_2 = \log(0.5)$, $\delta_0 = 0.00, \delta_1 = 12.50, \delta_2 = 25.00, \delta_3 = 31.25$, $\nu = 0.0$	δ_2	0.946 (0.932, 0.960)	0.951 (0.935, 0.968)
$\omega_1 = \omega_2 = \log(0.5)$, $\delta_0 = 0.00, \delta_1 = 12.50, \delta_2 = 25.00, \delta_3 = 31.25$, $\nu = 0.0$	δ_3	0.959 (0.947, 0.971)	0.958 (0.942, 0.973)
$\omega_1 = \omega_2 = \log(0.9)$, $\delta_0 = \delta_1 = \delta_2 = \delta_3 = 0.00$, $\nu = -0.2$	δ_0	0.932 (0.917, 0.947)	0.938 (0.923, 0.953)

Table B15: Coverage probability of treatment effect on the longitudinal outcome for the general time on treatment effect parametrisation, with 95% confidence intervals based on Monte Carlo errors. LMM denotes the linear mixed model, while JM denotes the joint model. *(continued)*

Scenario	Parameter	LMM	JM
$\omega_1 = \omega_2 = \log(0.9)$, $\delta_0 = \delta_1 = \delta_2 = \delta_3 = 0.00$, $\nu = -0.2$	δ_1	0.934 (0.919, 0.949)	0.940 (0.925, 0.955)
$\omega_1 = \omega_2 = \log(0.9)$, $\delta_0 = \delta_1 = \delta_2 = \delta_3 = 0.00$, $\nu = -0.2$	δ_2	0.947 (0.934, 0.961)	0.952 (0.938, 0.965)
$\omega_1 = \omega_2 = \log(0.9)$, $\delta_0 = \delta_1 = \delta_2 = \delta_3 = 0.00$, $\nu = -0.2$	δ_3	0.938 (0.923, 0.953)	0.950 (0.936, 0.963)
$\omega_1 = \omega_2 = \log(0.9)$, $\delta_0 = \delta_1 = \delta_2 = \delta_3 = 0.00$, $\nu = 0.0$	δ_0	0.946 (0.932, 0.959)	0.949 (0.935, 0.962)
$\omega_1 = \omega_2 = \log(0.9)$, $\delta_0 = \delta_1 = \delta_2 = \delta_3 = 0.00$, $\nu = 0.0$	δ_1	0.942 (0.927, 0.956)	0.950 (0.936, 0.963)
$\omega_1 = \omega_2 = \log(0.9)$, $\delta_0 = \delta_1 = \delta_2 = \delta_3 = 0.00$, $\nu = 0.0$	δ_2	0.952 (0.939, 0.965)	0.944 (0.929, 0.958)
$\omega_1 = \omega_2 = \log(0.9)$, $\delta_0 = \delta_1 = \delta_2 = \delta_3 = 0.00$, $\nu = 0.0$	δ_3	0.943 (0.929, 0.957)	0.941 (0.926, 0.955)
$\omega_1 = \omega_2 = \log(0.9)$, $\delta_0 = 0.00$, $\delta_1 = 2.50$, $\delta_2 = 5.00$, $\delta_3 = 6.25$, $\nu = -0.2$	δ_0	0.948 (0.934, 0.961)	0.947 (0.933, 0.962)
$\omega_1 = \omega_2 = \log(0.9)$, $\delta_0 = 0.00$, $\delta_1 = 2.50$, $\delta_2 = 5.00$, $\delta_3 = 6.25$, $\nu = -0.2$	δ_1	0.939 (0.924, 0.953)	0.946 (0.932, 0.961)
$\omega_1 = \omega_2 = \log(0.9)$, $\delta_0 = 0.00$, $\delta_1 = 2.50$, $\delta_2 = 5.00$, $\delta_3 = 6.25$, $\nu = -0.2$	δ_2	0.947 (0.933, 0.960)	0.946 (0.932, 0.961)
$\omega_1 = \omega_2 = \log(0.9)$, $\delta_0 = 0.00$, $\delta_1 = 2.50$, $\delta_2 = 5.00$, $\delta_3 = 6.25$, $\nu = -0.2$	δ_3	0.945 (0.931, 0.958)	0.934 (0.918, 0.949)
$\omega_1 = \omega_2 = \log(0.9)$, $\delta_0 = 0.00$, $\delta_1 = 2.50$, $\delta_2 = 5.00$, $\delta_3 = 6.25$, $\nu = 0.0$	δ_0	0.959 (0.947, 0.971)	0.959 (0.946, 0.971)
$\omega_1 = \omega_2 = \log(0.9)$, $\delta_0 = 0.00$, $\delta_1 = 2.50$, $\delta_2 = 5.00$, $\delta_3 = 6.25$, $\nu = 0.0$	δ_1	0.960 (0.948, 0.972)	0.955 (0.942, 0.968)
$\omega_1 = \omega_2 = \log(0.9)$, $\delta_0 = 0.00$, $\delta_1 = 2.50$, $\delta_2 = 5.00$, $\delta_3 = 6.25$, $\nu = 0.0$	δ_2	0.950 (0.937, 0.963)	0.943 (0.928, 0.957)
$\omega_1 = \omega_2 = \log(0.9)$, $\delta_0 = 0.00$, $\delta_1 = 2.50$, $\delta_2 = 5.00$, $\delta_3 = 6.25$, $\nu = 0.0$	δ_3	0.950 (0.937, 0.963)	0.944 (0.929, 0.958)
$\omega_1 = \omega_2 = \log(0.9)$, $\delta_0 = 0.00$, $\delta_1 = 12.50$, $\delta_2 = 25.00$, $\delta_3 = 31.25$, $\nu = -0.2$	δ_0	0.950 (0.937, 0.963)	0.954 (0.941, 0.967)
$\omega_1 = \omega_2 = \log(0.9)$, $\delta_0 = 0.00$, $\delta_1 = 12.50$, $\delta_2 = 25.00$, $\delta_3 = 31.25$, $\nu = -0.2$	δ_1	0.936 (0.921, 0.951)	0.947 (0.932, 0.961)
$\omega_1 = \omega_2 = \log(0.9)$, $\delta_0 = 0.00$, $\delta_1 = 12.50$, $\delta_2 = 25.00$, $\delta_3 = 31.25$, $\nu = -0.2$	δ_2	0.940 (0.925, 0.954)	0.943 (0.929, 0.958)
$\omega_1 = \omega_2 = \log(0.9)$, $\delta_0 = 0.00$, $\delta_1 = 12.50$, $\delta_2 = 25.00$, $\delta_3 = 31.25$, $\nu = -0.2$	δ_3	0.937 (0.922, 0.952)	0.937 (0.922, 0.953)
$\omega_1 = \omega_2 = \log(0.9)$, $\delta_0 = 0.00$, $\delta_1 = 12.50$, $\delta_2 = 25.00$, $\delta_3 = 31.25$, $\nu = 0.0$	δ_0	0.969 (0.958, 0.979)	0.958 (0.945, 0.970)
$\omega_1 = \omega_2 = \log(0.9)$, $\delta_0 = 0.00$, $\delta_1 = 12.50$, $\delta_2 = 25.00$, $\delta_3 = 31.25$, $\nu = 0.0$	δ_1	0.954 (0.942, 0.967)	0.956 (0.943, 0.969)
$\omega_1 = \omega_2 = \log(0.9)$, $\delta_0 = 0.00$, $\delta_1 = 12.50$, $\delta_2 = 25.00$, $\delta_3 = 31.25$, $\nu = 0.0$	δ_2	0.959 (0.947, 0.971)	0.955 (0.942, 0.968)
$\omega_1 = \omega_2 = \log(0.9)$, $\delta_0 = 0.00$, $\delta_1 = 12.50$, $\delta_2 = 25.00$, $\delta_3 = 31.25$, $\nu = 0.0$	δ_3	0.954 (0.942, 0.967)	0.958 (0.945, 0.970)
$\omega_1 = \omega_2 = \log(1.0)$, $\delta_0 = \delta_1 = \delta_2 = \delta_3 = 0.00$, $\nu = -0.2$	δ_0	0.950 (0.937, 0.964)	0.949 (0.935, 0.962)
$\omega_1 = \omega_2 = \log(1.0)$, $\delta_0 = \delta_1 = \delta_2 = \delta_3 = 0.00$, $\nu = -0.2$	δ_1	0.937 (0.922, 0.952)	0.936 (0.921, 0.951)
$\omega_1 = \omega_2 = \log(1.0)$, $\delta_0 = \delta_1 = \delta_2 = \delta_3 = 0.00$, $\nu = -0.2$	δ_2	0.942 (0.928, 0.956)	0.943 (0.928, 0.957)

Table B15: Coverage probability of treatment effect on the longitudinal outcome for the general time on treatment effect parametrisation, with 95% confidence intervals based on Monte Carlo errors. LMM denotes the linear mixed model, while JM denotes the joint model. *(continued)*

Scenario	Parameter	LMM	JM
$\omega_1 = \omega_2 = \log(1.0)$, $\delta_0 = \delta_1 = \delta_2 = \delta_3 = 0.00$, $\nu = -0.2$	δ_3	0.935 (0.920, 0.950)	0.934 (0.918, 0.949)
$\omega_1 = \omega_2 = \log(1.0)$, $\delta_0 = \delta_1 = \delta_2 = \delta_3 = 0.00$, $\nu = 0.0$	δ_0	0.951 (0.938, 0.964)	0.950 (0.937, 0.964)
$\omega_1 = \omega_2 = \log(1.0)$, $\delta_0 = \delta_1 = \delta_2 = \delta_3 = 0.00$, $\nu = 0.0$	δ_1	0.945 (0.931, 0.959)	0.944 (0.929, 0.958)
$\omega_1 = \omega_2 = \log(1.0)$, $\delta_0 = \delta_1 = \delta_2 = \delta_3 = 0.00$, $\nu = 0.0$	δ_2	0.960 (0.948, 0.972)	0.957 (0.945, 0.970)
$\omega_1 = \omega_2 = \log(1.0)$, $\delta_0 = \delta_1 = \delta_2 = \delta_3 = 0.00$, $\nu = 0.0$	δ_3	0.951 (0.938, 0.964)	0.951 (0.938, 0.964)
$\omega_1 = \omega_2 = \log(1.0)$, $\delta_0 = 0.00, \delta_1 = 2.50, \delta_2 = 5.00, \delta_3 = 6.25$, $\nu = -0.2$	δ_0	0.955 (0.943, 0.968)	0.953 (0.940, 0.966)
$\omega_1 = \omega_2 = \log(1.0)$, $\delta_0 = 0.00, \delta_1 = 2.50, \delta_2 = 5.00, \delta_3 = 6.25$, $\nu = -0.2$	δ_1	0.949 (0.935, 0.962)	0.952 (0.939, 0.965)
$\omega_1 = \omega_2 = \log(1.0)$, $\delta_0 = 0.00, \delta_1 = 2.50, \delta_2 = 5.00, \delta_3 = 6.25$, $\nu = -0.2$	δ_2	0.957 (0.945, 0.969)	0.956 (0.944, 0.969)
$\omega_1 = \omega_2 = \log(1.0)$, $\delta_0 = 0.00, \delta_1 = 2.50, \delta_2 = 5.00, \delta_3 = 6.25$, $\nu = -0.2$	δ_3	0.955 (0.943, 0.968)	0.954 (0.941, 0.967)
$\omega_1 = \omega_2 = \log(1.0)$, $\delta_0 = 0.00, \delta_1 = 2.50, \delta_2 = 5.00, \delta_3 = 6.25$, $\nu = 0.0$	δ_0	0.952 (0.939, 0.965)	0.949 (0.936, 0.963)
$\omega_1 = \omega_2 = \log(1.0)$, $\delta_0 = 0.00, \delta_1 = 2.50, \delta_2 = 5.00, \delta_3 = 6.25$, $\nu = 0.0$	δ_1	0.949 (0.936, 0.963)	0.950 (0.937, 0.964)
$\omega_1 = \omega_2 = \log(1.0)$, $\delta_0 = 0.00, \delta_1 = 2.50, \delta_2 = 5.00, \delta_3 = 6.25$, $\nu = 0.0$	δ_2	0.954 (0.942, 0.967)	0.953 (0.940, 0.966)
$\omega_1 = \omega_2 = \log(1.0)$, $\delta_0 = 0.00, \delta_1 = 2.50, \delta_2 = 5.00, \delta_3 = 6.25$, $\nu = 0.0$	δ_3	0.955 (0.943, 0.968)	0.952 (0.939, 0.965)
$\omega_1 = \omega_2 = \log(1.0)$, $\delta_0 = 0.00, \delta_1 = 12.50, \delta_2 = 25.00, \delta_3 = 31.25$, $\nu = -0.2$	δ_0	0.940 (0.925, 0.954)	0.938 (0.923, 0.952)
$\omega_1 = \omega_2 = \log(1.0)$, $\delta_0 = 0.00, \delta_1 = 12.50, \delta_2 = 25.00, \delta_3 = 31.25$, $\nu = -0.2$	δ_1	0.943 (0.929, 0.957)	0.942 (0.928, 0.957)
$\omega_1 = \omega_2 = \log(1.0)$, $\delta_0 = 0.00, \delta_1 = 12.50, \delta_2 = 25.00, \delta_3 = 31.25$, $\nu = -0.2$	δ_2	0.950 (0.937, 0.963)	0.947 (0.934, 0.961)
$\omega_1 = \omega_2 = \log(1.0)$, $\delta_0 = 0.00, \delta_1 = 12.50, \delta_2 = 25.00, \delta_3 = 31.25$, $\nu = -0.2$	δ_3	0.951 (0.938, 0.964)	0.951 (0.938, 0.964)
$\omega_1 = \omega_2 = \log(1.0)$, $\delta_0 = 0.00, \delta_1 = 12.50, \delta_2 = 25.00, \delta_3 = 31.25$, $\nu = 0.0$	δ_0	0.949 (0.935, 0.962)	0.946 (0.932, 0.960)
$\omega_1 = \omega_2 = \log(1.0)$, $\delta_0 = 0.00, \delta_1 = 12.50, \delta_2 = 25.00, \delta_3 = 31.25$, $\nu = 0.0$	δ_1	0.950 (0.936, 0.963)	0.952 (0.939, 0.965)
$\omega_1 = \omega_2 = \log(1.0)$, $\delta_0 = 0.00, \delta_1 = 12.50, \delta_2 = 25.00, \delta_3 = 31.25$, $\nu = 0.0$	δ_2	0.943 (0.929, 0.957)	0.947 (0.933, 0.961)
$\omega_1 = \omega_2 = \log(1.0)$, $\delta_0 = 0.00, \delta_1 = 12.50, \delta_2 = 25.00, \delta_3 = 31.25$, $\nu = 0.0$	δ_3	0.950 (0.937, 0.964)	0.947 (0.933, 0.961)
$\omega_1 = \omega_2 = \log(2.0)$, $\delta_0 = \delta_1 = \delta_2 = \delta_3 = 0.00$, $\nu = -0.2$	δ_0	0.956 (0.943, 0.969)	0.951 (0.934, 0.968)
$\omega_1 = \omega_2 = \log(2.0)$, $\delta_0 = \delta_1 = \delta_2 = \delta_3 = 0.00$, $\nu = -0.2$	δ_1	0.955 (0.942, 0.968)	0.965 (0.951, 0.980)
$\omega_1 = \omega_2 = \log(2.0)$, $\delta_0 = \delta_1 = \delta_2 = \delta_3 = 0.00$, $\nu = -0.2$	δ_2	0.951 (0.938, 0.964)	0.946 (0.929, 0.964)
$\omega_1 = \omega_2 = \log(2.0)$, $\delta_0 = \delta_1 = \delta_2 = \delta_3 = 0.00$, $\nu = -0.2$	δ_3	0.956 (0.943, 0.969)	0.943 (0.925, 0.961)
$\omega_1 = \omega_2 = \log(2.0)$, $\delta_0 = \delta_1 = \delta_2 = \delta_3 = 0.00$, $\nu = 0.0$	δ_0	0.951 (0.938, 0.964)	0.959 (0.943, 0.975)

Table B15: Coverage probability of treatment effect on the longitudinal outcome for the general time on treatment effect parametrisation, with 95% confidence intervals based on Monte Carlo errors. LMM denotes the linear mixed model, while JM denotes the joint model. *(continued)*

Scenario	Parameter	LMM	JM
$\omega_1 = \omega_2 = \log(2.0)$, $\delta_0 = \delta_1 = \delta_2 = \delta_3 = 0.00$, $\nu = 0.0$	δ_1	0.961 (0.949, 0.973)	0.954 (0.937, 0.971)
$\omega_1 = \omega_2 = \log(2.0)$, $\delta_0 = \delta_1 = \delta_2 = \delta_3 = 0.00$, $\nu = 0.0$	δ_2	0.945 (0.931, 0.959)	0.956 (0.939, 0.972)
$\omega_1 = \omega_2 = \log(2.0)$, $\delta_0 = \delta_1 = \delta_2 = \delta_3 = 0.00$, $\nu = 0.0$	δ_3	0.956 (0.943, 0.969)	0.954 (0.937, 0.971)
$\omega_1 = \omega_2 = \log(2.0)$, $\delta_0 = 0.00, \delta_1 = 2.50, \delta_2 = 5.00, \delta_3 = 6.25, \nu = -0.2$	δ_0	0.953 (0.940, 0.966)	0.951 (0.933, 0.968)
$\omega_1 = \omega_2 = \log(2.0)$, $\delta_0 = 0.00, \delta_1 = 2.50, \delta_2 = 5.00, \delta_3 = 6.25, \nu = -0.2$	δ_1	0.932 (0.917, 0.948)	0.932 (0.912, 0.952)
$\omega_1 = \omega_2 = \log(2.0)$, $\delta_0 = 0.00, \delta_1 = 2.50, \delta_2 = 5.00, \delta_3 = 6.25, \nu = -0.2$	δ_2	0.939 (0.924, 0.954)	0.941 (0.922, 0.959)
$\omega_1 = \omega_2 = \log(2.0)$, $\delta_0 = 0.00, \delta_1 = 2.50, \delta_2 = 5.00, \delta_3 = 6.25, \nu = -0.2$	δ_3	0.952 (0.939, 0.965)	0.947 (0.930, 0.965)
$\omega_1 = \omega_2 = \log(2.0)$, $\delta_0 = 0.00, \delta_1 = 2.50, \delta_2 = 5.00, \delta_3 = 6.25, \nu = 0.0$	δ_0	0.956 (0.944, 0.969)	0.954 (0.937, 0.971)
$\omega_1 = \omega_2 = \log(2.0)$, $\delta_0 = 0.00, \delta_1 = 2.50, \delta_2 = 5.00, \delta_3 = 6.25, \nu = 0.0$	δ_1	0.931 (0.915, 0.946)	0.961 (0.945, 0.976)
$\omega_1 = \omega_2 = \log(2.0)$, $\delta_0 = 0.00, \delta_1 = 2.50, \delta_2 = 5.00, \delta_3 = 6.25, \nu = 0.0$	δ_2	0.951 (0.937, 0.964)	0.952 (0.935, 0.969)
$\omega_1 = \omega_2 = \log(2.0)$, $\delta_0 = 0.00, \delta_1 = 2.50, \delta_2 = 5.00, \delta_3 = 6.25, \nu = 0.0$	δ_3	0.942 (0.927, 0.956)	0.930 (0.909, 0.950)
$\omega_1 = \omega_2 = \log(2.0)$, $\delta_0 = 0.00, \delta_1 = 12.50, \delta_2 = 25.00, \delta_3 = 31.25, \nu = -0.2$	δ_0	0.952 (0.939, 0.965)	0.956 (0.939, 0.972)
$\omega_1 = \omega_2 = \log(2.0)$, $\delta_0 = 0.00, \delta_1 = 12.50, \delta_2 = 25.00, \delta_3 = 31.25, \nu = -0.2$	δ_1	0.951 (0.938, 0.964)	0.956 (0.939, 0.972)
$\omega_1 = \omega_2 = \log(2.0)$, $\delta_0 = 0.00, \delta_1 = 12.50, \delta_2 = 25.00, \delta_3 = 31.25, \nu = -0.2$	δ_2	0.951 (0.938, 0.964)	0.964 (0.949, 0.979)
$\omega_1 = \omega_2 = \log(2.0)$, $\delta_0 = 0.00, \delta_1 = 12.50, \delta_2 = 25.00, \delta_3 = 31.25, \nu = -0.2$	δ_3	0.943 (0.929, 0.957)	0.965 (0.951, 0.980)
$\omega_1 = \omega_2 = \log(2.0)$, $\delta_0 = 0.00, \delta_1 = 12.50, \delta_2 = 25.00, \delta_3 = 31.25, \nu = 0.0$	δ_0	0.944 (0.930, 0.958)	0.940 (0.920, 0.959)
$\omega_1 = \omega_2 = \log(2.0)$, $\delta_0 = 0.00, \delta_1 = 12.50, \delta_2 = 25.00, \delta_3 = 31.25, \nu = 0.0$	δ_1	0.947 (0.933, 0.961)	0.950 (0.932, 0.967)
$\omega_1 = \omega_2 = \log(2.0)$, $\delta_0 = 0.00, \delta_1 = 12.50, \delta_2 = 25.00, \delta_3 = 31.25, \nu = 0.0$	δ_2	0.947 (0.933, 0.961)	0.960 (0.944, 0.976)
$\omega_1 = \omega_2 = \log(2.0)$, $\delta_0 = 0.00, \delta_1 = 12.50, \delta_2 = 25.00, \delta_3 = 31.25, \nu = 0.0$	δ_3	0.952 (0.939, 0.965)	0.948 (0.930, 0.966)
**Additional scenarios:**			
$i = 3 \times 4$	δ_0	0.951 (0.938, 0.964)	0.955 (0.941, 0.968)
$i = 3 \times 4$	δ_1	0.943 (0.929, 0.958)	0.944 (0.929, 0.959)
$i = 3 \times 4$	δ_2	0.961 (0.949, 0.973)	0.961 (0.949, 0.974)
$i = 3 \times 4$	δ_3	0.963 (0.951, 0.974)	0.967 (0.955, 0.979)
$i = 3 \times 4, N = 100$	δ_0	0.961 (0.949, 0.972)	0.949 (0.934, 0.963)
$i = 3 \times 4, N = 100$	δ_1	0.951 (0.938, 0.964)	0.950 (0.935, 0.964)

Table B15: Coverage probability of treatment effect on the longitudinal outcome for the general time on treatment effect parametrisation, with 95% confidence intervals based on Monte Carlo errors. LMM denotes the linear mixed model, while JM denotes the joint model. *(continued)*

Scenario	Parameter	LMM	JM
$i = 3 \times 4, N = 100$	δ_2	0.955 (0.942, 0.968)	0.956 (0.943, 0.970)
$i = 3 \times 4, N = 100$	δ_3	0.958 (0.946, 0.970)	0.955 (0.942, 0.969)
$\sigma_\alpha^2 = 1, \sigma_\phi^2 = 27.5$	δ_0	0.945 (0.931, 0.958)	0.942 (0.927, 0.956)
$\sigma_\alpha^2 = 1, \sigma_\phi^2 = 27.5$	δ_1	0.947 (0.934, 0.961)	0.951 (0.937, 0.964)
$\sigma_\alpha^2 = 1, \sigma_\phi^2 = 27.5$	δ_2	0.937 (0.922, 0.952)	0.941 (0.926, 0.955)
$\sigma_\alpha^2 = 1, \sigma_\phi^2 = 27.5$	δ_3	0.935 (0.920, 0.950)	0.949 (0.935, 0.962)
$\sigma_\alpha^2 = 4, \sigma_\phi^2 = 110$	δ_0	0.948 (0.934, 0.961)	0.950 (0.936, 0.964)
$\sigma_\alpha^2 = 4, \sigma_\phi^2 = 110$	δ_1	0.934 (0.919, 0.949)	0.943 (0.928, 0.958)
$\sigma_\alpha^2 = 4, \sigma_\phi^2 = 110$	δ_2	0.950 (0.937, 0.964)	0.953 (0.939, 0.967)
$\sigma_\alpha^2 = 4, \sigma_\phi^2 = 110$	δ_3	0.947 (0.933, 0.960)	0.953 (0.939, 0.967)

Table B16: Bias of period effects on the longitudinal outcome for the general time on treatment effect parametrisation, with 95% confidence intervals based on Monte Carlo errors. LMM denotes the linear mixed model, while JM denotes the joint model. Statistically significant biases are highlighted in bold.

Scenario	Parameter	LMM	JM
**Main scenarios:**			
$\omega_1 = \omega_2 = \log(0.5), \delta = 0.0, \nu = -0.2$	β_1	**5.049 (5.031, 5.067)**	**0.947 (0.917, 0.978)**
$\omega_1 = \omega_2 = \log(0.5), \delta = 0.0, \nu = -0.2$	β_2	**5.413 (5.394, 5.433)**	**0.983 (0.950, 1.017)**
$\omega_1 = \omega_2 = \log(0.5), \delta = 0.0, \nu = -0.2$	β_3	**5.570 (5.550, 5.591)**	**0.978 (0.944, 1.012)**
$\omega_1 = \omega_2 = \log(0.5), \delta = 0.0, \nu = -0.2$	β_4	**5.697 (5.671, 5.722)**	**1.018 (0.980, 1.057)**
$\omega_1 = \omega_2 = \log(0.5), \delta = 0.0, \nu = -0.2$	β_5	**5.790 (5.756, 5.824)**	**1.034 (0.986, 1.082)**
$\omega_1 = \omega_2 = \log(0.5), \delta = 0.0, \nu = 0.0$	β_1	**5.047 (5.028, 5.065)**	**0.950 (0.921, 0.980)**
$\omega_1 = \omega_2 = \log(0.5), \delta = 0.0, \nu = 0.0$	β_2	**5.424 (5.404, 5.443)**	**1.003 (0.971, 1.035)**
$\omega_1 = \omega_2 = \log(0.5), \delta = 0.0, \nu = 0.0$	β_3	**5.586 (5.565, 5.607)**	**1.005 (0.971, 1.038)**
$\omega_1 = \omega_2 = \log(0.5), \delta = 0.0, \nu = 0.0$	β_4	**5.701 (5.674, 5.727)**	**1.044 (1.006, 1.083)**
$\omega_1 = \omega_2 = \log(0.5), \delta = 0.0, \nu = 0.0$	β_5	**5.743 (5.708, 5.777)**	**1.053 (1.007, 1.100)**
$\omega_1 = \omega_2 = \log(0.5), \delta = 5.0, \nu = -0.2$	β_1	**5.045 (5.028, 5.062)**	**0.952 (0.922, 0.981)**
$\omega_1 = \omega_2 = \log(0.5), \delta = 5.0, \nu = -0.2$	β_2	**5.408 (5.389, 5.426)**	**0.976 (0.945, 1.007)**
$\omega_1 = \omega_2 = \log(0.5), \delta = 5.0, \nu = -0.2$	β_3	**5.566 (5.546, 5.587)**	**0.991 (0.958, 1.025)**
$\omega_1 = \omega_2 = \log(0.5), \delta = 5.0, \nu = -0.2$	β_4	**5.681 (5.654, 5.708)**	**1.006 (0.967, 1.044)**
$\omega_1 = \omega_2 = \log(0.5), \delta = 5.0, \nu = -0.2$	β_5	**5.742 (5.706, 5.778)**	**0.993 (0.945, 1.040)**
$\omega_1 = \omega_2 = \log(0.5), \delta = 5.0, \nu = 0.0$	β_1	**5.044 (5.026, 5.061)**	**0.949 (0.918, 0.981)**
$\omega_1 = \omega_2 = \log(0.5), \delta = 5.0, \nu = 0.0$	β_2	**5.418 (5.399, 5.437)**	**0.980 (0.947, 1.013)**
$\omega_1 = \omega_2 = \log(0.5), \delta = 5.0, \nu = 0.0$	β_3	**5.582 (5.559, 5.604)**	**0.994 (0.958, 1.030)**
$\omega_1 = \omega_2 = \log(0.5), \delta = 5.0, \nu = 0.0$	β_4	**5.674 (5.646, 5.701)**	**1.003 (0.962, 1.044)**
$\omega_1 = \omega_2 = \log(0.5), \delta = 5.0, \nu = 0.0$	β_5	**5.751 (5.714, 5.789)**	**1.029 (0.980, 1.078)**
$\omega_1 = \omega_2 = \log(0.5), \delta = 25, \nu = -0.2$	β_1	**5.040 (5.022, 5.057)**	**0.948 (0.918, 0.978)**
$\omega_1 = \omega_2 = \log(0.5), \delta = 25, \nu = -0.2$	β_2	**5.418 (5.399, 5.438)**	**0.990 (0.958, 1.021)**
$\omega_1 = \omega_2 = \log(0.5), \delta = 25, \nu = -0.2$	β_3	**5.586 (5.564, 5.608)**	**1.005 (0.971, 1.040)**
$\omega_1 = \omega_2 = \log(0.5), \delta = 25, \nu = -0.2$	β_4	**5.685 (5.658, 5.712)**	**1.016 (0.976, 1.056)**

Table B16: Bias of period effects on the longitudinal outcome for the general time on treatment effect parametrisation, with 95% confidence intervals based on Monte Carlo errors. LMM denotes the linear mixed model, while JM denotes the joint model. Statistically significant biases are highlighted in bold. *(continued)*

Scenario	Parameter	LMM	JM
$\omega_1 = \omega_2 = \log(0.5)$, $\delta = 25$, $\nu = -0.2$	β_5	**5.769 (5.733, 5.805)**	**1.022 (0.973, 1.071)**
$\omega_1 = \omega_2 = \log(0.5)$, $\delta = 25$, $\nu = 0.0$	β_1	**5.044 (5.026, 5.062)**	**0.947 (0.917, 0.978)**
$\omega_1 = \omega_2 = \log(0.5)$, $\delta = 25$, $\nu = 0.0$	β_2	**5.411 (5.392, 5.431)**	**0.961 (0.929, 0.993)**
$\omega_1 = \omega_2 = \log(0.5)$, $\delta = 25$, $\nu = 0.0$	β_3	**5.570 (5.548, 5.592)**	**0.973 (0.939, 1.007)**
$\omega_1 = \omega_2 = \log(0.5)$, $\delta = 25$, $\nu = 0.0$	β_4	**5.672 (5.644, 5.699)**	**0.981 (0.943, 1.020)**
$\omega_1 = \omega_2 = \log(0.5)$, $\delta = 25$, $\nu = 0.0$	β_5	**5.731 (5.695, 5.768)**	**0.990 (0.943, 1.038)**
$\omega_1 = \omega_2 = \log(0.9)$, $\delta = 0.0$, $\nu = -0.2$	β_1	**1.489 (1.469, 1.510)**	0.016 (-0.008, 0.041)
$\omega_1 = \omega_2 = \log(0.9)$, $\delta = 0.0$, $\nu = -0.2$	β_2	**1.999 (1.977, 2.021)**	0.026 (-0.001, 0.053)
$\omega_1 = \omega_2 = \log(0.9)$, $\delta = 0.0$, $\nu = -0.2$	β_3	**2.283 (2.257, 2.309)**	**0.039 (0.008, 0.070)**
$\omega_1 = \omega_2 = \log(0.9)$, $\delta = 0.0$, $\nu = -0.2$	β_4	**2.466 (2.435, 2.498)**	**0.037 (0.000, 0.073)**
$\omega_1 = \omega_2 = \log(0.9)$, $\delta = 0.0$, $\nu = -0.2$	β_5	**2.615 (2.573, 2.656)**	0.036 (-0.010, 0.081)
$\omega_1 = \omega_2 = \log(0.9)$, $\delta = 0.0$, $\nu = 0.0$	β_1	**1.460 (1.440, 1.481)**	-0.022 (-0.046, 0.002)
$\omega_1 = \omega_2 = \log(0.9)$, $\delta = 0.0$, $\nu = 0.0$	β_2	**1.962 (1.939, 1.984)**	**-0.032 (-0.059, -0.004)**
$\omega_1 = \omega_2 = \log(0.9)$, $\delta = 0.0$, $\nu = 0.0$	β_3	**2.220 (2.194, 2.245)**	**-0.038 (-0.069, -0.008)**
$\omega_1 = \omega_2 = \log(0.9)$, $\delta = 0.0$, $\nu = 0.0$	β_4	**2.387 (2.355, 2.419)**	**-0.041 (-0.078, -0.004)**
$\omega_1 = \omega_2 = \log(0.9)$, $\delta = 0.0$, $\nu = 0.0$	β_5	**2.495 (2.452, 2.537)**	**-0.051 (-0.098, -0.005)**
$\omega_1 = \omega_2 = \log(0.9)$, $\delta = 5.0$, $\nu = -0.2$	β_1	**1.464 (1.443, 1.484)**	-0.018 (-0.043, 0.007)
$\omega_1 = \omega_2 = \log(0.9)$, $\delta = 5.0$, $\nu = -0.2$	β_2	**1.974 (1.952, 1.996)**	-0.010 (-0.038, 0.019)
$\omega_1 = \omega_2 = \log(0.9)$, $\delta = 5.0$, $\nu = -0.2$	β_3	**2.247 (2.223, 2.272)**	-0.008 (-0.039, 0.023)
$\omega_1 = \omega_2 = \log(0.9)$, $\delta = 5.0$, $\nu = -0.2$	β_4	**2.443 (2.413, 2.473)**	0.003 (-0.034, 0.039)
$\omega_1 = \omega_2 = \log(0.9)$, $\delta = 5.0$, $\nu = -0.2$	β_5	**2.598 (2.559, 2.637)**	0.011 (-0.034, 0.056)
$\omega_1 = \omega_2 = \log(0.9)$, $\delta = 5.0$, $\nu = 0.0$	β_1	**1.474 (1.453, 1.496)**	0.001 (-0.025, 0.026)
$\omega_1 = \omega_2 = \log(0.9)$, $\delta = 5.0$, $\nu = 0.0$	β_2	**1.981 (1.958, 2.005)**	-0.002 (-0.030, 0.026)
$\omega_1 = \omega_2 = \log(0.9)$, $\delta = 5.0$, $\nu = 0.0$	β_3	**2.253 (2.226, 2.280)**	0.000 (-0.032, 0.033)
$\omega_1 = \omega_2 = \log(0.9)$, $\delta = 5.0$, $\nu = 0.0$	β_4	**2.450 (2.419, 2.482)**	0.025 (-0.012, 0.061)

Table B16: Bias of period effects on the longitudinal outcome for the general time on treatment effect parametrisation, with 95% confidence intervals based on Monte Carlo errors. LMM denotes the linear mixed model, while JM denotes the joint model. Statistically significant biases are highlighted in bold. *(continued)*

Scenario	Parameter	LMM	JM
$\omega_1 = \omega_2 = \log(0.9)$, $\delta = 5.0$, $\nu = 0.0$	β_5	**2.587 (2.546, 2.629)**	0.042 (-0.004, 0.088)
$\omega_1 = \omega_2 = \log(0.9)$, $\delta = 25$, $\nu = -0.2$	β_1	**1.473 (1.453, 1.493)**	-0.002 (-0.026, 0.023)
$\omega_1 = \omega_2 = \log(0.9)$, $\delta = 25$, $\nu = -0.2$	β_2	**1.983 (1.961, 2.005)**	0.006 (-0.022, 0.033)
$\omega_1 = \omega_2 = \log(0.9)$, $\delta = 25$, $\nu = -0.2$	β_3	**2.261 (2.236, 2.286)**	0.013 (-0.018, 0.044)
$\omega_1 = \omega_2 = \log(0.9)$, $\delta = 25$, $\nu = -0.2$	β_4	**2.451 (2.420, 2.483)**	0.016 (-0.022, 0.053)
$\omega_1 = \omega_2 = \log(0.9)$, $\delta = 25$, $\nu = -0.2$	β_5	**2.612 (2.571, 2.654)**	0.031 (-0.015, 0.078)
$\omega_1 = \omega_2 = \log(0.9)$, $\delta = 25$, $\nu = 0.0$	β_1	**1.503 (1.482, 1.524)**	0.023 (-0.001, 0.048)
$\omega_1 = \omega_2 = \log(0.9)$, $\delta = 25$, $\nu = 0.0$	β_2	**1.997 (1.974, 2.019)**	0.007 (-0.020, 0.033)
$\omega_1 = \omega_2 = \log(0.9)$, $\delta = 25$, $\nu = 0.0$	β_3	**2.279 (2.253, 2.304)**	0.020 (-0.010, 0.050)
$\omega_1 = \omega_2 = \log(0.9)$, $\delta = 25$, $\nu = 0.0$	β_4	**2.461 (2.430, 2.492)**	**0.038 (0.003, 0.073)**
$\omega_1 = \omega_2 = \log(0.9)$, $\delta = 25$, $\nu = 0.0$	β_5	**2.577 (2.537, 2.617)**	0.031 (-0.012, 0.075)
$\omega_1 = \omega_2 = \log(1.0)$, $\delta = 0.0$, $\nu = -0.2$	β_1	-0.007 (-0.028, 0.015)	-0.009 (-0.031, 0.013)
$\omega_1 = \omega_2 = \log(1.0)$, $\delta = 0.0$, $\nu = -0.2$	β_2	-0.018 (-0.041, 0.004)	-0.021 (-0.045, 0.002)
$\omega_1 = \omega_2 = \log(1.0)$, $\delta = 0.0$, $\nu = -0.2$	β_3	-0.013 (-0.039, 0.014)	-0.014 (-0.042, 0.014)
$\omega_1 = \omega_2 = \log(1.0)$, $\delta = 0.0$, $\nu = -0.2$	β_4	0.000 (-0.032, 0.033)	0.002 (-0.032, 0.036)
$\omega_1 = \omega_2 = \log(1.0)$, $\delta = 0.0$, $\nu = -0.2$	β_5	-0.009 (-0.052, 0.034)	-0.010 (-0.055, 0.034)
$\omega_1 = \omega_2 = \log(1.0)$, $\delta = 0.0$, $\nu = 0.0$	β_1	-0.008 (-0.030, 0.013)	-0.006 (-0.029, 0.016)
$\omega_1 = \omega_2 = \log(1.0)$, $\delta = 0.0$, $\nu = 0.0$	β_2	-0.010 (-0.035, 0.014)	-0.009 (-0.034, 0.016)
$\omega_1 = \omega_2 = \log(1.0)$, $\delta = 0.0$, $\nu = 0.0$	β_3	-0.017 (-0.044, 0.010)	-0.015 (-0.043, 0.013)
$\omega_1 = \omega_2 = \log(1.0)$, $\delta = 0.0$, $\nu = 0.0$	β_4	-0.018 (-0.050, 0.014)	-0.017 (-0.050, 0.017)
$\omega_1 = \omega_2 = \log(1.0)$, $\delta = 0.0$, $\nu = 0.0$	β_5	-0.026 (-0.068, 0.015)	-0.023 (-0.066, 0.020)
$\omega_1 = \omega_2 = \log(1.0)$, $\delta = 5.0$, $\nu = -0.2$	β_1	-0.008 (-0.030, 0.014)	-0.006 (-0.029, 0.016)
$\omega_1 = \omega_2 = \log(1.0)$, $\delta = 5.0$, $\nu = -0.2$	β_2	-0.021 (-0.046, 0.004)	-0.021 (-0.046, 0.005)
$\omega_1 = \omega_2 = \log(1.0)$, $\delta = 5.0$, $\nu = -0.2$	β_3	-0.011 (-0.040, 0.017)	-0.011 (-0.040, 0.018)
$\omega_1 = \omega_2 = \log(1.0)$, $\delta = 5.0$, $\nu = -0.2$	β_4	-0.021 (-0.055, 0.012)	-0.022 (-0.056, 0.013)

Table B16: Bias of period effects on the longitudinal outcome for the general time on treatment effect parametrisation, with 95% confidence intervals based on Monte Carlo errors. LMM denotes the linear mixed model, while JM denotes the joint model. Statistically significant biases are highlighted in bold. *(continued)*

Scenario	Parameter	LMM	JM
$\omega_1 = \omega_2 = \log(1.0)$, $\delta = 5.0$, $\nu = -0.2$	β_5	-0.030 (-0.073, 0.013)	-0.030 (-0.075, 0.014)
$\omega_1 = \omega_2 = \log(1.0)$, $\delta = 5.0$, $\nu = 0.0$	β_1	0.004 (-0.019, 0.026)	0.005 (-0.019, 0.028)
$\omega_1 = \omega_2 = \log(1.0)$, $\delta = 5.0$, $\nu = 0.0$	β_2	0.024 (-0.001, 0.048)	0.024 (-0.001, 0.049)
$\omega_1 = \omega_2 = \log(1.0)$, $\delta = 5.0$, $\nu = 0.0$	β_3	-0.007 (-0.035, 0.020)	-0.008 (-0.037, 0.020)
$\omega_1 = \omega_2 = \log(1.0)$, $\delta = 5.0$, $\nu = 0.0$	β_4	0.004 (-0.029, 0.037)	0.003 (-0.031, 0.038)
$\omega_1 = \omega_2 = \log(1.0)$, $\delta = 5.0$, $\nu = 0.0$	β_5	-0.012 (-0.055, 0.030)	-0.010 (-0.054, 0.034)
$\omega_1 = \omega_2 = \log(1.0)$, $\delta = 25$, $\nu = -0.2$	β_1	-0.011 (-0.033, 0.011)	-0.011 (-0.034, 0.011)
$\omega_1 = \omega_2 = \log(1.0)$, $\delta = 25$, $\nu = -0.2$	β_2	**-0.026 (-0.049, -0.002)**	**-0.026 (-0.050, -0.002)**
$\omega_1 = \omega_2 = \log(1.0)$, $\delta = 25$, $\nu = -0.2$	β_3	**-0.028 (-0.054, -0.001)**	**-0.028 (-0.055, -0.001)**
$\omega_1 = \omega_2 = \log(1.0)$, $\delta = 25$, $\nu = -0.2$	β_4	-0.031 (-0.063, 0.001)	-0.030 (-0.064, 0.003)
$\omega_1 = \omega_2 = \log(1.0)$, $\delta = 25$, $\nu = -0.2$	β_5	**-0.051 (-0.093, -0.010)**	**-0.050 (-0.092, -0.007)**
$\omega_1 = \omega_2 = \log(1.0)$, $\delta = 25$, $\nu = 0.0$	β_1	-0.009 (-0.031, 0.014)	-0.010 (-0.033, 0.012)
$\omega_1 = \omega_2 = \log(1.0)$, $\delta = 25$, $\nu = 0.0$	β_2	0.006 (-0.017, 0.030)	0.004 (-0.021, 0.028)
$\omega_1 = \omega_2 = \log(1.0)$, $\delta = 25$, $\nu = 0.0$	β_3	0.002 (-0.026, 0.030)	-0.001 (-0.030, 0.028)
$\omega_1 = \omega_2 = \log(1.0)$, $\delta = 25$, $\nu = 0.0$	β_4	0.003 (-0.030, 0.037)	0.004 (-0.030, 0.038)
$\omega_1 = \omega_2 = \log(1.0)$, $\delta = 25$, $\nu = 0.0$	β_5	0.011 (-0.032, 0.055)	0.007 (-0.038, 0.051)
$\omega_1 = \omega_2 = \log(2.0)$, $\delta = 0.0$, $\nu = -0.2$	β_1	**-5.033 (-5.051, -5.015)**	**-0.930 (-0.960, -0.900)**
$\omega_1 = \omega_2 = \log(2.0)$, $\delta = 0.0$, $\nu = -0.2$	β_2	**-5.390 (-5.409, -5.371)**	**-0.949 (-0.981, -0.917)**
$\omega_1 = \omega_2 = \log(2.0)$, $\delta = 0.0$, $\nu = -0.2$	β_3	**-5.560 (-5.582, -5.539)**	**-0.958 (-0.992, -0.925)**
$\omega_1 = \omega_2 = \log(2.0)$, $\delta = 0.0$, $\nu = -0.2$	β_4	**-5.667 (-5.694, -5.641)**	**-0.989 (-1.028, -0.949)**
$\omega_1 = \omega_2 = \log(2.0)$, $\delta = 0.0$, $\nu = -0.2$	β_5	**-5.746 (-5.782, -5.711)**	**-0.990 (-1.037, -0.943)**
$\omega_1 = \omega_2 = \log(2.0)$, $\delta = 0.0$, $\nu = 0.0$	β_1	**-5.046 (-5.064, -5.028)**	**-0.959 (-0.991, -0.926)**
$\omega_1 = \omega_2 = \log(2.0)$, $\delta = 0.0$, $\nu = 0.0$	β_2	**-5.415 (-5.434, -5.396)**	**-0.981 (-1.015, -0.947)**
$\omega_1 = \omega_2 = \log(2.0)$, $\delta = 0.0$, $\nu = 0.0$	β_3	**-5.583 (-5.605, -5.560)**	**-1.005 (-1.042, -0.967)**
$\omega_1 = \omega_2 = \log(2.0)$, $\delta = 0.0$, $\nu = 0.0$	β_4	**-5.692 (-5.719, -5.666)**	**-1.042 (-1.084, -1.001)**

Table B16: Bias of period effects on the longitudinal outcome for the general time on treatment effect parametrisation, with 95% confidence intervals based on Monte Carlo errors. LMM denotes the linear mixed model, while JM denotes the joint model. Statistically significant biases are highlighted in bold. *(continued)*

Scenario	Parameter	LMM	JM
$\omega_1 = \omega_2 = \log(2.0)$, $\delta = 0.0$, $\nu = 0.0$	β_5	**-5.755 (-5.791, -5.719)**	**-1.029 (-1.080, -0.978)**
$\omega_1 = \omega_2 = \log(2.0)$, $\delta = 5.0$, $\nu = -0.2$	β_1	**-5.041 (-5.059, -5.024)**	**-0.940 (-0.973, -0.907)**
$\omega_1 = \omega_2 = \log(2.0)$, $\delta = 5.0$, $\nu = -0.2$	β_2	**-5.403 (-5.421, -5.384)**	**-0.966 (-0.999, -0.932)**
$\omega_1 = \omega_2 = \log(2.0)$, $\delta = 5.0$, $\nu = -0.2$	β_3	**-5.584 (-5.605, -5.563)**	**-0.992 (-1.028, -0.956)**
$\omega_1 = \omega_2 = \log(2.0)$, $\delta = 5.0$, $\nu = -0.2$	β_4	**-5.673 (-5.700, -5.646)**	**-0.985 (-1.028, -0.942)**
$\omega_1 = \omega_2 = \log(2.0)$, $\delta = 5.0$, $\nu = -0.2$	β_5	**-5.787 (-5.823, -5.752)**	**-1.027 (-1.079, -0.976)**
$\omega_1 = \omega_2 = \log(2.0)$, $\delta = 5.0$, $\nu = 0.0$	β_1	**-5.052 (-5.070, -5.035)**	**-0.960 (-0.993, -0.928)**
$\omega_1 = \omega_2 = \log(2.0)$, $\delta = 5.0$, $\nu = 0.0$	β_2	**-5.422 (-5.440, -5.403)**	**-0.986 (-1.019, -0.953)**
$\omega_1 = \omega_2 = \log(2.0)$, $\delta = 5.0$, $\nu = 0.0$	β_3	**-5.590 (-5.612, -5.568)**	**-1.007 (-1.044, -0.970)**
$\omega_1 = \omega_2 = \log(2.0)$, $\delta = 5.0$, $\nu = 0.0$	β_4	**-5.697 (-5.725, -5.670)**	**-1.033 (-1.074, -0.991)**
$\omega_1 = \omega_2 = \log(2.0)$, $\delta = 5.0$, $\nu = 0.0$	β_5	**-5.758 (-5.795, -5.722)**	**-1.047 (-1.098, -0.997)**
$\omega_1 = \omega_2 = \log(2.0)$, $\delta = 25$, $\nu = -0.2$	β_1	**-5.041 (-5.059, -5.024)**	**-0.937 (-0.969, -0.906)**
$\omega_1 = \omega_2 = \log(2.0)$, $\delta = 25$, $\nu = -0.2$	β_2	**-5.410 (-5.429, -5.392)**	**-0.956 (-0.989, -0.923)**
$\omega_1 = \omega_2 = \log(2.0)$, $\delta = 25$, $\nu = -0.2$	β_3	**-5.573 (-5.594, -5.551)**	**-0.971 (-1.007, -0.935)**
$\omega_1 = \omega_2 = \log(2.0)$, $\delta = 25$, $\nu = -0.2$	β_4	**-5.694 (-5.721, -5.667)**	**-0.973 (-1.014, -0.933)**
$\omega_1 = \omega_2 = \log(2.0)$, $\delta = 25$, $\nu = -0.2$	β_5	**-5.787 (-5.822, -5.753)**	**-0.985 (-1.033, -0.937)**
$\omega_1 = \omega_2 = \log(2.0)$, $\delta = 25$, $\nu = 0.0$	β_1	**-5.052 (-5.070, -5.035)**	**-0.931 (-0.962, -0.901)**
$\omega_1 = \omega_2 = \log(2.0)$, $\delta = 25$, $\nu = 0.0$	β_2	**-5.431 (-5.450, -5.411)**	**-0.977 (-1.011, -0.944)**
$\omega_1 = \omega_2 = \log(2.0)$, $\delta = 25$, $\nu = 0.0$	β_3	**-5.575 (-5.597, -5.553)**	**-0.956 (-0.992, -0.920)**
$\omega_1 = \omega_2 = \log(2.0)$, $\delta = 25$, $\nu = 0.0$	β_4	**-5.671 (-5.700, -5.643)**	**-0.972 (-1.014, -0.929)**
$\omega_1 = \omega_2 = \log(2.0)$, $\delta = 25$, $\nu = 0.0$	β_5	**-5.739 (-5.776, -5.702)**	**-0.975 (-1.028, -0.922)**
**Additional scenarios:**			
$i = 3 \times 4$	β_1	**1.484 (1.449, 1.519)**	-0.025 (-0.069, 0.019)
$i = 3 \times 4$	β_2	**1.993 (1.957, 2.029)**	-0.017 (-0.064, 0.030)
$i = 3 \times 4$	β_3	**2.260 (2.220, 2.300)**	-0.037 (-0.090, 0.015)

Table B16: Bias of period effects on the longitudinal outcome for the general time on treatment effect parametrisation, with 95% confidence intervals based on Monte Carlo errors. LMM denotes the linear mixed model, while JM denotes the joint model. Statistically significant biases are highlighted in bold. *(continued)*

Scenario	Parameter	LMM	JM
$i = 3 \times 4$	β_4	**2.439 (2.390, 2.488)**	-0.052 (-0.113, 0.008)
$i = 3 \times 4$	β_5	**2.629 (2.565, 2.693)**	0.006 (-0.070, 0.081)
$i = 3 \times 4, N = 100$	β_1	**1.492 (1.463, 1.520)**	0.024 (-0.012, 0.059)
$i = 3 \times 4, N = 100$	β_2	**1.996 (1.965, 2.026)**	0.028 (-0.010, 0.066)
$i = 3 \times 4, N = 100$	β_3	**2.249 (2.216, 2.281)**	0.014 (-0.027, 0.055)
$i = 3 \times 4, N = 100$	β_4	**2.445 (2.406, 2.485)**	0.026 (-0.021, 0.074)
$i = 3 \times 4, N = 100$	β_5	**2.572 (2.522, 2.622)**	0.014 (-0.045, 0.073)
$\sigma^2_\alpha = 1, \sigma^2_\phi = 27.5$	β_1	**0.697 (0.679, 0.714)**	-0.011 (-0.030, 0.008)
$\sigma^2_\alpha = 1, \sigma^2_\phi = 27.5$	β_2	**1.067 (1.048, 1.085)**	-0.014 (-0.035, 0.008)
$\sigma^2_\alpha = 1, \sigma^2_\phi = 27.5$	β_3	**1.322 (1.300, 1.344)**	-0.002 (-0.028, 0.023)
$\sigma^2_\alpha = 1, \sigma^2_\phi = 27.5$	β_4	**1.502 (1.474, 1.530)**	-0.004 (-0.035, 0.028)
$\sigma^2_\alpha = 1, \sigma^2_\phi = 27.5$	β_5	**1.669 (1.631, 1.708)**	0.013 (-0.028, 0.054)
$\sigma^2_\alpha = 4, \sigma^2_\phi = 110$	β_1	**3.024 (2.998, 3.050)**	-0.017 (-0.052, 0.018)
$\sigma^2_\alpha = 4, \sigma^2_\phi = 110$	β_2	**3.590 (3.563, 3.617)**	-0.014 (-0.052, 0.024)
$\sigma^2_\alpha = 4, \sigma^2_\phi = 110$	β_3	**3.829 (3.800, 3.859)**	-0.022 (-0.063, 0.018)
$\sigma^2_\alpha = 4, \sigma^2_\phi = 110$	β_4	**3.996 (3.962, 4.030)**	-0.024 (-0.070, 0.022)
$\sigma^2_\alpha = 4, \sigma^2_\phi = 110$	β_5	**4.133 (4.088, 4.178)**	-0.016 (-0.071, 0.038)

Table B17: Relative bias of period effects on the longitudinal outcome for the general time on treatment effect parametrisation, with 95% confidence intervals based on Monte Carlo errors. LMM denotes the linear mixed model, while JM denotes the joint model. Statistically significant biases are highlighted in bold.

Scenario	Parameter	LMM	JM
**Main scenarios:**			
$\omega_1 = \omega_2 = \log(0.5)$, $\delta = 0.0$, $\nu = -0.2$	β_1	**0.168 (0.168, 0.169)**	**0.032 (0.031, 0.033)**
$\omega_1 = \omega_2 = \log(0.5)$, $\delta = 0.0$, $\nu = -0.2$	β_2	**0.180 (0.180, 0.181)**	**0.033 (0.032, 0.034)**
$\omega_1 = \omega_2 = \log(0.5)$, $\delta = 0.0$, $\nu = -0.2$	β_3	**0.186 (0.185, 0.186)**	**0.033 (0.031, 0.034)**
$\omega_1 = \omega_2 = \log(0.5)$, $\delta = 0.0$, $\nu = -0.2$	β_4	**0.190 (0.189, 0.191)**	**0.034 (0.033, 0.035)**
$\omega_1 = \omega_2 = \log(0.5)$, $\delta = 0.0$, $\nu = -0.2$	β_5	**0.193 (0.192, 0.194)**	**0.034 (0.033, 0.036)**
$\omega_1 = \omega_2 = \log(0.5)$, $\delta = 0.0$, $\nu = 0.0$	β_1	**0.168 (0.168, 0.169)**	**0.032 (0.031, 0.033)**
$\omega_1 = \omega_2 = \log(0.5)$, $\delta = 0.0$, $\nu = 0.0$	β_2	**0.181 (0.180, 0.181)**	**0.033 (0.032, 0.034)**
$\omega_1 = \omega_2 = \log(0.5)$, $\delta = 0.0$, $\nu = 0.0$	β_3	**0.186 (0.185, 0.187)**	**0.033 (0.032, 0.035)**
$\omega_1 = \omega_2 = \log(0.5)$, $\delta = 0.0$, $\nu = 0.0$	β_4	**0.190 (0.189, 0.191)**	**0.035 (0.034, 0.036)**
$\omega_1 = \omega_2 = \log(0.5)$, $\delta = 0.0$, $\nu = 0.0$	β_5	**0.191 (0.190, 0.193)**	**0.035 (0.034, 0.037)**
$\omega_1 = \omega_2 = \log(0.5)$, $\delta = 5.0$, $\nu = -0.2$	β_1	**0.168 (0.168, 0.169)**	**0.032 (0.031, 0.033)**
$\omega_1 = \omega_2 = \log(0.5)$, $\delta = 5.0$, $\nu = -0.2$	β_2	**0.180 (0.180, 0.181)**	**0.033 (0.031, 0.034)**
$\omega_1 = \omega_2 = \log(0.5)$, $\delta = 5.0$, $\nu = -0.2$	β_3	**0.186 (0.185, 0.186)**	**0.033 (0.032, 0.034)**
$\omega_1 = \omega_2 = \log(0.5)$, $\delta = 5.0$, $\nu = -0.2$	β_4	**0.189 (0.188, 0.190)**	**0.034 (0.032, 0.035)**
$\omega_1 = \omega_2 = \log(0.5)$, $\delta = 5.0$, $\nu = -0.2$	β_5	**0.191 (0.190, 0.193)**	**0.033 (0.032, 0.035)**
$\omega_1 = \omega_2 = \log(0.5)$, $\delta = 5.0$, $\nu = 0.0$	β_1	**0.168 (0.168, 0.169)**	**0.032 (0.031, 0.033)**
$\omega_1 = \omega_2 = \log(0.5)$, $\delta = 5.0$, $\nu = 0.0$	β_2	**0.181 (0.180, 0.181)**	**0.033 (0.032, 0.034)**
$\omega_1 = \omega_2 = \log(0.5)$, $\delta = 5.0$, $\nu = 0.0$	β_3	**0.186 (0.185, 0.187)**	**0.033 (0.032, 0.034)**
$\omega_1 = \omega_2 = \log(0.5)$, $\delta = 5.0$, $\nu = 0.0$	β_4	**0.189 (0.188, 0.190)**	**0.033 (0.032, 0.035)**
$\omega_1 = \omega_2 = \log(0.5)$, $\delta = 5.0$, $\nu = 0.0$	β_5	**0.192 (0.190, 0.193)**	**0.034 (0.033, 0.036)**
$\omega_1 = \omega_2 = \log(0.5)$, $\delta = 25$, $\nu = -0.2$	β_1	**0.168 (0.167, 0.169)**	**0.032 (0.031, 0.033)**
$\omega_1 = \omega_2 = \log(0.5)$, $\delta = 25$, $\nu = -0.2$	β_2	**0.181 (0.180, 0.181)**	**0.033 (0.032, 0.034)**
$\omega_1 = \omega_2 = \log(0.5)$, $\delta = 25$, $\nu = -0.2$	β_3	**0.186 (0.185, 0.187)**	**0.034 (0.032, 0.035)**
$\omega_1 = \omega_2 = \log(0.5)$, $\delta = 25$, $\nu = -0.2$	β_4	**0.190 (0.189, 0.190)**	**0.034 (0.033, 0.035)**

Table B17: Relative bias of period effects on the longitudinal outcome for the general time on treatment effect parametrisation, with 95% confidence intervals based on Monte Carlo errors. LMM denotes the linear mixed model, while JM denotes the joint model. Statistically significant biases are highlighted in bold. *(continued)*

Scenario	Parameter	LMM	JM
$\omega_1 = \omega_2 = \log(0.5)$, $\delta = 25$, $\nu = -0.2$	β_5	**0.192 (0.191, 0.194)**	**0.034 (0.032, 0.036)**
$\omega_1 = \omega_2 = \log(0.5)$, $\delta = 25$, $\nu = 0.0$	β_1	**0.168 (0.168, 0.169)**	**0.032 (0.031, 0.033)**
$\omega_1 = \omega_2 = \log(0.5)$, $\delta = 25$, $\nu = 0.0$	β_2	**0.180 (0.180, 0.181)**	**0.032 (0.031, 0.033)**
$\omega_1 = \omega_2 = \log(0.5)$, $\delta = 25$, $\nu = 0.0$	β_3	**0.186 (0.185, 0.186)**	**0.032 (0.031, 0.034)**
$\omega_1 = \omega_2 = \log(0.5)$, $\delta = 25$, $\nu = 0.0$	β_4	**0.189 (0.188, 0.190)**	**0.033 (0.031, 0.034)**
$\omega_1 = \omega_2 = \log(0.5)$, $\delta = 25$, $\nu = 0.0$	β_5	**0.191 (0.190, 0.192)**	**0.033 (0.031, 0.035)**
$\omega_1 = \omega_2 = \log(0.9)$, $\delta = 0.0$, $\nu = -0.2$	β_1	**0.050 (0.049, 0.050)**	0.001 (-0.000, 0.001)
$\omega_1 = \omega_2 = \log(0.9)$, $\delta = 0.0$, $\nu = -0.2$	β_2	**0.067 (0.066, 0.067)**	0.001 (-0.000, 0.002)
$\omega_1 = \omega_2 = \log(0.9)$, $\delta = 0.0$, $\nu = -0.2$	β_3	**0.076 (0.075, 0.077)**	**0.001 (0.000, 0.002)**
$\omega_1 = \omega_2 = \log(0.9)$, $\delta = 0.0$, $\nu = -0.2$	β_4	**0.082 (0.081, 0.083)**	**0.001 (0.000, 0.002)**
$\omega_1 = \omega_2 = \log(0.9)$, $\delta = 0.0$, $\nu = -0.2$	β_5	**0.087 (0.086, 0.089)**	0.001 (-0.000, 0.003)
$\omega_1 = \omega_2 = \log(0.9)$, $\delta = 0.0$, $\nu = 0.0$	β_1	**0.049 (0.048, 0.049)**	-0.001 (-0.002, 0.000)
$\omega_1 = \omega_2 = \log(0.9)$, $\delta = 0.0$, $\nu = 0.0$	β_2	**0.065 (0.065, 0.066)**	**-0.001 (-0.002, -0.000)**
$\omega_1 = \omega_2 = \log(0.9)$, $\delta = 0.0$, $\nu = 0.0$	β_3	**0.074 (0.073, 0.075)**	**-0.001 (-0.002, -0.000)**
$\omega_1 = \omega_2 = \log(0.9)$, $\delta = 0.0$, $\nu = 0.0$	β_4	**0.080 (0.079, 0.081)**	**-0.001 (-0.003, -0.000)**
$\omega_1 = \omega_2 = \log(0.9)$, $\delta = 0.0$, $\nu = 0.0$	β_5	**0.083 (0.082, 0.085)**	**-0.002 (-0.003, -0.000)**
$\omega_1 = \omega_2 = \log(0.9)$, $\delta = 5.0$, $\nu = -0.2$	β_1	**0.049 (0.048, 0.049)**	-0.001 (-0.001, 0.000)
$\omega_1 = \omega_2 = \log(0.9)$, $\delta = 5.0$, $\nu = -0.2$	β_2	**0.066 (0.065, 0.067)**	-0.000 (-0.001, 0.001)
$\omega_1 = \omega_2 = \log(0.9)$, $\delta = 5.0$, $\nu = -0.2$	β_3	**0.075 (0.074, 0.076)**	-0.000 (-0.001, 0.001)
$\omega_1 = \omega_2 = \log(0.9)$, $\delta = 5.0$, $\nu = -0.2$	β_4	**0.081 (0.080, 0.082)**	0.000 (-0.001, 0.001)
$\omega_1 = \omega_2 = \log(0.9)$, $\delta = 5.0$, $\nu = -0.2$	β_5	**0.087 (0.085, 0.088)**	0.000 (-0.001, 0.002)
$\omega_1 = \omega_2 = \log(0.9)$, $\delta = 5.0$, $\nu = 0.0$	β_1	**0.049 (0.048, 0.050)**	0.000 (-0.001, 0.001)
$\omega_1 = \omega_2 = \log(0.9)$, $\delta = 5.0$, $\nu = 0.0$	β_2	**0.066 (0.065, 0.067)**	-0.000 (-0.001, 0.001)
$\omega_1 = \omega_2 = \log(0.9)$, $\delta = 5.0$, $\nu = 0.0$	β_3	**0.075 (0.074, 0.076)**	0.000 (-0.001, 0.001)
$\omega_1 = \omega_2 = \log(0.9)$, $\delta = 5.0$, $\nu = 0.0$	β_4	**0.082 (0.081, 0.083)**	0.001 (-0.000, 0.002)

Table B17: Relative bias of period effects on the longitudinal outcome for the general time on treatment effect parametrisation, with 95% confidence intervals based on Monte Carlo errors. LMM denotes the linear mixed model, while JM denotes the joint model. Statistically significant biases are highlighted in bold. *(continued)*

Scenario	Parameter	LMM	JM
$\omega_1 = \omega_2 = \log(0.9)$, $\delta = 5.0$, $\nu = 0.0$	β_5	**0.086 (0.085, 0.088)**	0.001 (-0.000, 0.003)
$\omega_1 = \omega_2 = \log(0.9)$, $\delta = 25$, $\nu = -0.2$	β_1	**0.049 (0.048, 0.050)**	-0.000 (-0.001, 0.001)
$\omega_1 = \omega_2 = \log(0.9)$, $\delta = 25$, $\nu = -0.2$	β_2	**0.066 (0.065, 0.067)**	0.000 (-0.001, 0.001)
$\omega_1 = \omega_2 = \log(0.9)$, $\delta = 25$, $\nu = -0.2$	β_3	**0.075 (0.075, 0.076)**	0.000 (-0.001, 0.001)
$\omega_1 = \omega_2 = \log(0.9)$, $\delta = 25$, $\nu = -0.2$	β_4	**0.082 (0.081, 0.083)**	0.001 (-0.001, 0.002)
$\omega_1 = \omega_2 = \log(0.9)$, $\delta = 25$, $\nu = -0.2$	β_5	**0.087 (0.086, 0.088)**	0.001 (-0.000, 0.003)
$\omega_1 = \omega_2 = \log(0.9)$, $\delta = 25$, $\nu = 0.0$	β_1	**0.050 (0.049, 0.051)**	0.001 (-0.000, 0.002)
$\omega_1 = \omega_2 = \log(0.9)$, $\delta = 25$, $\nu = 0.0$	β_2	**0.067 (0.066, 0.067)**	0.000 (-0.001, 0.001)
$\omega_1 = \omega_2 = \log(0.9)$, $\delta = 25$, $\nu = 0.0$	β_3	**0.076 (0.075, 0.077)**	0.001 (-0.000, 0.002)
$\omega_1 = \omega_2 = \log(0.9)$, $\delta = 25$, $\nu = 0.0$	β_4	**0.082 (0.081, 0.083)**	**0.001 (0.000, 0.002)**
$\omega_1 = \omega_2 = \log(0.9)$, $\delta = 25$, $\nu = 0.0$	β_5	**0.086 (0.085, 0.087)**	0.001 (-0.000, 0.002)
$\omega_1 = \omega_2 = \log(1.0)$, $\delta = 0.0$, $\nu = -0.2$	β_1	-0.000 (-0.001, 0.000)	-0.000 (-0.001, 0.000)
$\omega_1 = \omega_2 = \log(1.0)$, $\delta = 0.0$, $\nu = -0.2$	β_2	-0.001 (-0.001, 0.000)	-0.001 (-0.001, 0.000)
$\omega_1 = \omega_2 = \log(1.0)$, $\delta = 0.0$, $\nu = -0.2$	β_3	-0.000 (-0.001, 0.000)	-0.000 (-0.001, 0.000)
$\omega_1 = \omega_2 = \log(1.0)$, $\delta = 0.0$, $\nu = -0.2$	β_4	0.000 (-0.001, 0.001)	0.000 (-0.001, 0.001)
$\omega_1 = \omega_2 = \log(1.0)$, $\delta = 0.0$, $\nu = -0.2$	β_5	-0.000 (-0.002, 0.001)	-0.000 (-0.002, 0.001)
$\omega_1 = \omega_2 = \log(1.0)$, $\delta = 0.0$, $\nu = 0.0$	β_1	-0.000 (-0.001, 0.000)	-0.000 (-0.001, 0.001)
$\omega_1 = \omega_2 = \log(1.0)$, $\delta = 0.0$, $\nu = 0.0$	β_2	-0.000 (-0.001, 0.000)	-0.000 (-0.001, 0.001)
$\omega_1 = \omega_2 = \log(1.0)$, $\delta = 0.0$, $\nu = 0.0$	β_3	-0.001 (-0.001, 0.000)	-0.001 (-0.001, 0.000)
$\omega_1 = \omega_2 = \log(1.0)$, $\delta = 0.0$, $\nu = 0.0$	β_4	-0.001 (-0.002, 0.000)	-0.001 (-0.002, 0.001)
$\omega_1 = \omega_2 = \log(1.0)$, $\delta = 0.0$, $\nu = 0.0$	β_5	-0.001 (-0.002, 0.001)	-0.001 (-0.002, 0.001)
$\omega_1 = \omega_2 = \log(1.0)$, $\delta = 5.0$, $\nu = -0.2$	β_1	-0.000 (-0.001, 0.000)	-0.000 (-0.001, 0.001)
$\omega_1 = \omega_2 = \log(1.0)$, $\delta = 5.0$, $\nu = -0.2$	β_2	-0.001 (-0.002, 0.000)	-0.001 (-0.002, 0.000)
$\omega_1 = \omega_2 = \log(1.0)$, $\delta = 5.0$, $\nu = -0.2$	β_3	-0.000 (-0.001, 0.001)	-0.000 (-0.001, 0.001)
$\omega_1 = \omega_2 = \log(1.0)$, $\delta = 5.0$, $\nu = -0.2$	β_4	-0.001 (-0.002, 0.000)	-0.001 (-0.002, 0.000)

Table B17: Relative bias of period effects on the longitudinal outcome for the general time on treatment effect parametrisation, with 95% confidence intervals based on Monte Carlo errors. LMM denotes the linear mixed model, while JM denotes the joint model. Statistically significant biases are highlighted in bold. *(continued)*

Scenario	Parameter	LMM	JM
$\omega_1 = \omega_2 = \log(1.0)$, $\delta = 5.0$, $\nu = -0.2$	β_5	-0.001 (-0.002, 0.000)	-0.001 (-0.002, 0.000)
$\omega_1 = \omega_2 = \log(1.0)$, $\delta = 5.0$, $\nu = 0.0$	β_1	0.000 (-0.001, 0.001)	0.000 (-0.001, 0.001)
$\omega_1 = \omega_2 = \log(1.0)$, $\delta = 5.0$, $\nu = 0.0$	β_2	0.001 (-0.000, 0.002)	0.001 (-0.000, 0.002)
$\omega_1 = \omega_2 = \log(1.0)$, $\delta = 5.0$, $\nu = 0.0$	β_3	-0.000 (-0.001, 0.001)	-0.000 (-0.001, 0.001)
$\omega_1 = \omega_2 = \log(1.0)$, $\delta = 5.0$, $\nu = 0.0$	β_4	0.000 (-0.001, 0.001)	0.000 (-0.001, 0.001)
$\omega_1 = \omega_2 = \log(1.0)$, $\delta = 5.0$, $\nu = 0.0$	β_5	-0.000 (-0.002, 0.001)	-0.000 (-0.002, 0.001)
$\omega_1 = \omega_2 = \log(1.0)$, $\delta = 25$, $\nu = -0.2$	β_1	-0.000 (-0.001, 0.000)	-0.000 (-0.001, 0.000)
$\omega_1 = \omega_2 = \log(1.0)$, $\delta = 25$, $\nu = -0.2$	β_2	**-0.001 (-0.002, -0.000)**	**-0.001 (-0.002, -0.000)**
$\omega_1 = \omega_2 = \log(1.0)$, $\delta = 25$, $\nu = -0.2$	β_3	**-0.001 (-0.002, -0.000)**	**-0.001 (-0.002, -0.000)**
$\omega_1 = \omega_2 = \log(1.0)$, $\delta = 25$, $\nu = -0.2$	β_4	-0.001 (-0.002, 0.000)	-0.001 (-0.002, 0.000)
$\omega_1 = \omega_2 = \log(1.0)$, $\delta = 25$, $\nu = -0.2$	β_5	**-0.002 (-0.003, -0.000)**	**-0.002 (-0.003, -0.000)**
$\omega_1 = \omega_2 = \log(1.0)$, $\delta = 25$, $\nu = 0.0$	β_1	-0.000 (-0.001, 0.000)	-0.000 (-0.001, 0.000)
$\omega_1 = \omega_2 = \log(1.0)$, $\delta = 25$, $\nu = 0.0$	β_2	0.000 (-0.001, 0.001)	0.000 (-0.001, 0.001)
$\omega_1 = \omega_2 = \log(1.0)$, $\delta = 25$, $\nu = 0.0$	β_3	0.000 (-0.001, 0.001)	-0.000 (-0.001, 0.001)
$\omega_1 = \omega_2 = \log(1.0)$, $\delta = 25$, $\nu = 0.0$	β_4	0.000 (-0.001, 0.001)	0.000 (-0.001, 0.001)
$\omega_1 = \omega_2 = \log(1.0)$, $\delta = 25$, $\nu = 0.0$	β_5	0.000 (-0.001, 0.002)	0.000 (-0.001, 0.002)
$\omega_1 = \omega_2 = \log(2.0)$, $\delta = 0.0$, $\nu = -0.2$	β_1	**-0.168 (-0.168, -0.167)**	**-0.031 (-0.032, -0.030)**
$\omega_1 = \omega_2 = \log(2.0)$, $\delta = 0.0$, $\nu = -0.2$	β_2	**-0.180 (-0.180, -0.179)**	**-0.032 (-0.033, -0.031)**
$\omega_1 = \omega_2 = \log(2.0)$, $\delta = 0.0$, $\nu = -0.2$	β_3	**-0.185 (-0.186, -0.185)**	**-0.032 (-0.033, -0.031)**
$\omega_1 = \omega_2 = \log(2.0)$, $\delta = 0.0$, $\nu = -0.2$	β_4	**-0.189 (-0.190, -0.188)**	**-0.033 (-0.034, -0.032)**
$\omega_1 = \omega_2 = \log(2.0)$, $\delta = 0.0$, $\nu = -0.2$	β_5	**-0.192 (-0.193, -0.190)**	**-0.033 (-0.035, -0.031)**
$\omega_1 = \omega_2 = \log(2.0)$, $\delta = 0.0$, $\nu = 0.0$	β_1	**-0.168 (-0.169, -0.168)**	**-0.032 (-0.033, -0.031)**
$\omega_1 = \omega_2 = \log(2.0)$, $\delta = 0.0$, $\nu = 0.0$	β_2	**-0.180 (-0.181, -0.180)**	**-0.033 (-0.034, -0.032)**
$\omega_1 = \omega_2 = \log(2.0)$, $\delta = 0.0$, $\nu = 0.0$	β_3	**-0.186 (-0.187, -0.185)**	**-0.033 (-0.035, -0.032)**
$\omega_1 = \omega_2 = \log(2.0)$, $\delta = 0.0$, $\nu = 0.0$	β_4	**-0.190 (-0.191, -0.189)**	**-0.035 (-0.036, -0.033)**

Table B17: Relative bias of period effects on the longitudinal outcome for the general time on treatment effect parametrisation, with 95% confidence intervals based on Monte Carlo errors. LMM denotes the linear mixed model, while JM denotes the joint model. Statistically significant biases are highlighted in bold. *(continued)*

Scenario	Parameter	LMM	JM
$\omega_1 = \omega_2 = \log(2.0)$, $\delta = 0.0$, $\nu = 0.0$	β_5	**-0.192 (-0.193, -0.191)**	**-0.034 (-0.036, -0.033)**
$\omega_1 = \omega_2 = \log(2.0)$, $\delta = 5.0$, $\nu = -0.2$	β_1	**-0.168 (-0.169, -0.167)**	**-0.031 (-0.032, -0.030)**
$\omega_1 = \omega_2 = \log(2.0)$, $\delta = 5.0$, $\nu = -0.2$	β_2	**-0.180 (-0.181, -0.179)**	**-0.032 (-0.033, -0.031)**
$\omega_1 = \omega_2 = \log(2.0)$, $\delta = 5.0$, $\nu = -0.2$	β_3	**-0.186 (-0.187, -0.185)**	**-0.033 (-0.034, -0.032)**
$\omega_1 = \omega_2 = \log(2.0)$, $\delta = 5.0$, $\nu = -0.2$	β_4	**-0.189 (-0.190, -0.188)**	**-0.033 (-0.034, -0.031)**
$\omega_1 = \omega_2 = \log(2.0)$, $\delta = 5.0$, $\nu = -0.2$	β_5	**-0.193 (-0.194, -0.192)**	**-0.034 (-0.036, -0.033)**
$\omega_1 = \omega_2 = \log(2.0)$, $\delta = 5.0$, $\nu = 0.0$	β_1	**-0.168 (-0.169, -0.168)**	**-0.032 (-0.033, -0.031)**
$\omega_1 = \omega_2 = \log(2.0)$, $\delta = 5.0$, $\nu = 0.0$	β_2	**-0.181 (-0.181, -0.180)**	**-0.033 (-0.034, -0.032)**
$\omega_1 = \omega_2 = \log(2.0)$, $\delta = 5.0$, $\nu = 0.0$	β_3	**-0.186 (-0.187, -0.186)**	**-0.034 (-0.035, -0.032)**
$\omega_1 = \omega_2 = \log(2.0)$, $\delta = 5.0$, $\nu = 0.0$	β_4	**-0.190 (-0.191, -0.189)**	**-0.034 (-0.036, -0.033)**
$\omega_1 = \omega_2 = \log(2.0)$, $\delta = 5.0$, $\nu = 0.0$	β_5	**-0.192 (-0.193, -0.191)**	**-0.035 (-0.037, -0.033)**
$\omega_1 = \omega_2 = \log(2.0)$, $\delta = 25$, $\nu = -0.2$	β_1	**-0.168 (-0.169, -0.167)**	**-0.031 (-0.032, -0.030)**
$\omega_1 = \omega_2 = \log(2.0)$, $\delta = 25$, $\nu = -0.2$	β_2	**-0.180 (-0.181, -0.180)**	**-0.032 (-0.033, -0.031)**
$\omega_1 = \omega_2 = \log(2.0)$, $\delta = 25$, $\nu = -0.2$	β_3	**-0.186 (-0.186, -0.185)**	**-0.032 (-0.034, -0.031)**
$\omega_1 = \omega_2 = \log(2.0)$, $\delta = 25$, $\nu = -0.2$	β_4	**-0.190 (-0.191, -0.189)**	**-0.032 (-0.034, -0.031)**
$\omega_1 = \omega_2 = \log(2.0)$, $\delta = 25$, $\nu = -0.2$	β_5	**-0.193 (-0.194, -0.192)**	**-0.033 (-0.034, -0.031)**
$\omega_1 = \omega_2 = \log(2.0)$, $\delta = 25$, $\nu = 0.0$	β_1	**-0.168 (-0.169, -0.168)**	**-0.031 (-0.032, -0.030)**
$\omega_1 = \omega_2 = \log(2.0)$, $\delta = 25$, $\nu = 0.0$	β_2	**-0.181 (-0.182, -0.180)**	**-0.033 (-0.034, -0.031)**
$\omega_1 = \omega_2 = \log(2.0)$, $\delta = 25$, $\nu = 0.0$	β_3	**-0.186 (-0.187, -0.185)**	**-0.032 (-0.033, -0.031)**
$\omega_1 = \omega_2 = \log(2.0)$, $\delta = 25$, $\nu = 0.0$	β_4	**-0.189 (-0.190, -0.188)**	**-0.032 (-0.034, -0.031)**
$\omega_1 = \omega_2 = \log(2.0)$, $\delta = 25$, $\nu = 0.0$	β_5	**-0.191 (-0.193, -0.190)**	**-0.032 (-0.034, -0.031)**
**Additional scenarios:**			
$i = 3 \times 4$	β_1	**0.049 (0.048, 0.051)**	-0.001 (-0.002, 0.001)
$i = 3 \times 4$	β_2	**0.066 (0.065, 0.068)**	-0.001 (-0.002, 0.001)
$i = 3 \times 4$	β_3	**0.075 (0.074, 0.077)**	-0.001 (-0.003, 0.001)

Table B17: Relative bias of period effects on the longitudinal outcome for the general time on treatment effect parametrisation, with 95% confidence intervals based on Monte Carlo errors. LMM denotes the linear mixed model, while JM denotes the joint model. Statistically significant biases are highlighted in bold. *(continued)*

Scenario	Parameter	LMM	JM
$i = 3 \times 4$	β_4	**0.081 (0.080, 0.083)**	-0.002 (-0.004, 0.000)
$i = 3 \times 4$	β_5	**0.088 (0.085, 0.090)**	0.000 (-0.002, 0.003)
$i = 3 \times 4, N = 100$	β_1	**0.050 (0.049, 0.051)**	0.001 (-0.000, 0.002)
$i = 3 \times 4, N = 100$	β_2	**0.067 (0.066, 0.068)**	0.001 (-0.000, 0.002)
$i = 3 \times 4, N = 100$	β_3	**0.075 (0.074, 0.076)**	0.000 (-0.001, 0.002)
$i = 3 \times 4, N = 100$	β_4	**0.082 (0.080, 0.083)**	0.001 (-0.001, 0.002)
$i = 3 \times 4, N = 100$	β_5	**0.086 (0.084, 0.087)**	0.000 (-0.001, 0.002)
$\sigma^2_\alpha = 1, \sigma^2_\phi = 27.5$	β_1	**0.023 (0.023, 0.024)**	-0.000 (-0.001, 0.000)
$\sigma^2_\alpha = 1, \sigma^2_\phi = 27.5$	β_2	**0.036 (0.035, 0.036)**	-0.000 (-0.001, 0.000)
$\sigma^2_\alpha = 1, \sigma^2_\phi = 27.5$	β_3	**0.044 (0.043, 0.045)**	-0.000 (-0.001, 0.001)
$\sigma^2_\alpha = 1, \sigma^2_\phi = 27.5$	β_4	**0.050 (0.049, 0.051)**	-0.000 (-0.001, 0.001)
$\sigma^2_\alpha = 1, \sigma^2_\phi = 27.5$	β_5	**0.056 (0.054, 0.057)**	0.000 (-0.001, 0.002)
$\sigma^2_\alpha = 4, \sigma^2_\phi = 110$	β_1	**0.101 (0.100, 0.102)**	-0.001 (-0.002, 0.001)
$\sigma^2_\alpha = 4, \sigma^2_\phi = 110$	β_2	**0.120 (0.119, 0.121)**	-0.000 (-0.002, 0.001)
$\sigma^2_\alpha = 4, \sigma^2_\phi = 110$	β_3	**0.128 (0.127, 0.129)**	-0.001 (-0.002, 0.001)
$\sigma^2_\alpha = 4, \sigma^2_\phi = 110$	β_4	**0.133 (0.132, 0.134)**	-0.001 (-0.002, 0.001)
$\sigma^2_\alpha = 4, \sigma^2_\phi = 110$	β_5	**0.138 (0.136, 0.139)**	-0.001 (-0.002, 0.001)

Table B18: Coverage probability of period effects on the longitudinal outcome for the general time on treatment effect parametrisation, with 95% confidence intervals based on Monte Carlo errors. LMM denotes the linear mixed model, while JM denotes the joint model.

Scenario	Parameter	LMM	JM
**Main scenarios:**			
$\omega_1 = \omega_2 = \log(0.5)$, $\delta_0 = \delta_1 = \delta_2 = \delta_3 = 0.00$, $\nu = -0.2$	β_1	0.000 (0.000, 0.000)	0.575 (0.538, 0.613)
$\omega_1 = \omega_2 = \log(0.5)$, $\delta_0 = \delta_1 = \delta_2 = \delta_3 = 0.00$, $\nu = -0.2$	β_2	0.000 (0.000, 0.000)	0.536 (0.498, 0.574)
$\omega_1 = \omega_2 = \log(0.5)$, $\delta_0 = \delta_1 = \delta_2 = \delta_3 = 0.00$, $\nu = -0.2$	β_3	0.000 (0.000, 0.000)	0.608 (0.571, 0.645)
$\omega_1 = \omega_2 = \log(0.5)$, $\delta_0 = \delta_1 = \delta_2 = \delta_3 = 0.00$, $\nu = -0.2$	β_4	0.000 (0.000, 0.000)	0.634 (0.597, 0.670)
$\omega_1 = \omega_2 = \log(0.5)$, $\delta_0 = \delta_1 = \delta_2 = \delta_3 = 0.00$, $\nu = -0.2$	β_5	0.000 (0.000, 0.000)	0.740 (0.707, 0.774)
$\omega_1 = \omega_2 = \log(0.5)$, $\delta_0 = \delta_1 = \delta_2 = \delta_3 = 0.00$, $\nu = 0.0$	β_1	0.000 (0.000, 0.000)	0.553 (0.516, 0.590)
$\omega_1 = \omega_2 = \log(0.5)$, $\delta_0 = \delta_1 = \delta_2 = \delta_3 = 0.00$, $\nu = 0.0$	β_2	0.000 (0.000, 0.000)	0.528 (0.490, 0.565)
$\omega_1 = \omega_2 = \log(0.5)$, $\delta_0 = \delta_1 = \delta_2 = \delta_3 = 0.00$, $\nu = 0.0$	β_3	0.000 (0.000, 0.000)	0.571 (0.533, 0.608)
$\omega_1 = \omega_2 = \log(0.5)$, $\delta_0 = \delta_1 = \delta_2 = \delta_3 = 0.00$, $\nu = 0.0$	β_4	0.000 (0.000, 0.000)	0.615 (0.578, 0.651)
$\omega_1 = \omega_2 = \log(0.5)$, $\delta_0 = \delta_1 = \delta_2 = \delta_3 = 0.00$, $\nu = 0.0$	β_5	0.000 (0.000, 0.000)	0.706 (0.672, 0.740)
$\omega_1 = \omega_2 = \log(0.5)$, $\delta_0 = 0.00, \delta_1 = 2.50, \delta_2 = 5.00, \delta_3 = 6.25$, $\nu = -0.2$	β_1	0.000 (0.000, 0.000)	0.552 (0.514, 0.589)
$\omega_1 = \omega_2 = \log(0.5)$, $\delta_0 = 0.00, \delta_1 = 2.50, \delta_2 = 5.00, \delta_3 = 6.25$, $\nu = -0.2$	β_2	0.000 (0.000, 0.000)	0.562 (0.524, 0.600)
$\omega_1 = \omega_2 = \log(0.5)$, $\delta_0 = 0.00, \delta_1 = 2.50, \delta_2 = 5.00, \delta_3 = 6.25$, $\nu = -0.2$	β_3	0.000 (0.000, 0.000)	0.565 (0.527, 0.603)
$\omega_1 = \omega_2 = \log(0.5)$, $\delta_0 = 0.00, \delta_1 = 2.50, \delta_2 = 5.00, \delta_3 = 6.25$, $\nu = -0.2$	β_4	0.000 (0.000, 0.000)	0.638 (0.602, 0.675)
$\omega_1 = \omega_2 = \log(0.5)$, $\delta_0 = 0.00, \delta_1 = 2.50, \delta_2 = 5.00, \delta_3 = 6.25$, $\nu = -0.2$	β_5	0.000 (0.000, 0.000)	0.762 (0.730, 0.795)
$\omega_1 = \omega_2 = \log(0.5)$, $\delta_0 = 0.00, \delta_1 = 2.50, \delta_2 = 5.00, \delta_3 = 6.25$, $\nu = 0.0$	β_1	0.000 (0.000, 0.000)	0.547 (0.509, 0.585)
$\omega_1 = \omega_2 = \log(0.5)$, $\delta_0 = 0.00, \delta_1 = 2.50, \delta_2 = 5.00, \delta_3 = 6.25$, $\nu = 0.0$	β_2	0.000 (0.000, 0.000)	0.533 (0.495, 0.572)
$\omega_1 = \omega_2 = \log(0.5)$, $\delta_0 = 0.00, \delta_1 = 2.50, \delta_2 = 5.00, \delta_3 = 6.25$, $\nu = 0.0$	β_3	0.000 (0.000, 0.000)	0.569 (0.530, 0.607)
$\omega_1 = \omega_2 = \log(0.5)$, $\delta_0 = 0.00, \delta_1 = 2.50, \delta_2 = 5.00, \delta_3 = 6.25$, $\nu = 0.0$	β_4	0.000 (0.000, 0.000)	0.638 (0.601, 0.675)
$\omega_1 = \omega_2 = \log(0.5)$, $\delta_0 = 0.00, \delta_1 = 2.50, \delta_2 = 5.00, \delta_3 = 6.25$, $\nu = 0.0$	β_5	0.000 (0.000, 0.000)	0.723 (0.688, 0.757)
$\omega_1 = \omega_2 = \log(0.5)$, $\delta_0 = 0.00, \delta_1 = 12.50, \delta_2 = 25.00, \delta_3 = 31.25$, $\nu = -0.2$	β_1	0.000 (0.000, 0.000)	0.566 (0.529, 0.604)
$\omega_1 = \omega_2 = \log(0.5)$, $\delta_0 = 0.00, \delta_1 = 12.50, \delta_2 = 25.00, \delta_3 = 31.25$, $\nu = -0.2$	β_2	0.000 (0.000, 0.000)	0.535 (0.497, 0.573)
$\omega_1 = \omega_2 = \log(0.5)$, $\delta_0 = 0.00, \delta_1 = 12.50, \delta_2 = 25.00, \delta_3 = 31.25$, $\nu = -0.2$	β_3	0.000 (0.000, 0.000)	0.559 (0.521, 0.597)
$\omega_1 = \omega_2 = \log(0.5)$, $\delta_0 = 0.00, \delta_1 = 12.50, \delta_2 = 25.00, \delta_3 = 31.25$, $\nu = -0.2$	β_4	0.000 (0.000, 0.000)	0.633 (0.596, 0.670)
$\omega_1 = \omega_2 = \log(0.5)$, $\delta_0 = 0.00, \delta_1 = 12.50, \delta_2 = 25.00, \delta_3 = 31.25$, $\nu = -0.2$	β_5	0.000 (0.000, 0.000)	0.707 (0.672, 0.742)

Table B18: Coverage probability of period effects on the longitudinal outcome for the general time on treatment effect parametrisation, with 95% confidence intervals based on Monte Carlo errors. LMM denotes the linear mixed model, while JM denotes the joint model. *(continued)*

Scenario	Parameter	LMM	JM
$\omega_1 = \omega_2 = \log(0.5), \delta_0 = 0.00, \delta_1 = 12.50, \delta_2 = 25.00, \delta_3 = 31.25, \nu = 0.0$	β_1	0.000 (0.000, 0.000)	0.564 (0.526, 0.603)
$\omega_1 = \omega_2 = \log(0.5), \delta_0 = 0.00, \delta_1 = 12.50, \delta_2 = 25.00, \delta_3 = 31.25, \nu = 0.0$	β_2	0.000 (0.000, 0.000)	0.586 (0.548, 0.624)
$\omega_1 = \omega_2 = \log(0.5), \delta_0 = 0.00, \delta_1 = 12.50, \delta_2 = 25.00, \delta_3 = 31.25, \nu = 0.0$	β_3	0.000 (0.000, 0.000)	0.585 (0.546, 0.623)
$\omega_1 = \omega_2 = \log(0.5), \delta_0 = 0.00, \delta_1 = 12.50, \delta_2 = 25.00, \delta_3 = 31.25, \nu = 0.0$	β_4	0.000 (0.000, 0.000)	0.665 (0.628, 0.701)
$\omega_1 = \omega_2 = \log(0.5), \delta_0 = 0.00, \delta_1 = 12.50, \delta_2 = 25.00, \delta_3 = 31.25, \nu = 0.0$	β_5	0.000 (0.000, 0.000)	0.737 (0.703, 0.771)
$\omega_1 = \omega_2 = \log(0.9), \delta_0 = \delta_1 = \delta_2 = \delta_3 = 0.00, \nu = -0.2$	β_1	0.005 (0.001, 0.009)	0.946 (0.932, 0.961)
$\omega_1 = \omega_2 = \log(0.9), \delta_0 = \delta_1 = \delta_2 = \delta_3 = 0.00, \nu = -0.2$	β_2	0.001 (-0.001, 0.003)	0.958 (0.945, 0.971)
$\omega_1 = \omega_2 = \log(0.9), \delta_0 = \delta_1 = \delta_2 = \delta_3 = 0.00, \nu = -0.2$	β_3	0.000 (0.000, 0.000)	0.947 (0.933, 0.962)
$\omega_1 = \omega_2 = \log(0.9), \delta_0 = \delta_1 = \delta_2 = \delta_3 = 0.00, \nu = -0.2$	β_4	0.001 (-0.001, 0.003)	0.951 (0.937, 0.964)
$\omega_1 = \omega_2 = \log(0.9), \delta_0 = \delta_1 = \delta_2 = \delta_3 = 0.00, \nu = -0.2$	β_5	0.037 (0.026, 0.049)	0.941 (0.926, 0.956)
$\omega_1 = \omega_2 = \log(0.9), \delta_0 = \delta_1 = \delta_2 = \delta_3 = 0.00, \nu = 0.0$	β_1	0.011 (0.004, 0.017)	0.945 (0.931, 0.959)
$\omega_1 = \omega_2 = \log(0.9), \delta_0 = \delta_1 = \delta_2 = \delta_3 = 0.00, \nu = 0.0$	β_2	0.002 (-0.001, 0.005)	0.930 (0.914, 0.946)
$\omega_1 = \omega_2 = \log(0.9), \delta_0 = \delta_1 = \delta_2 = \delta_3 = 0.00, \nu = 0.0$	β_3	0.003 (-0.000, 0.006)	0.947 (0.933, 0.961)
$\omega_1 = \omega_2 = \log(0.9), \delta_0 = \delta_1 = \delta_2 = \delta_3 = 0.00, \nu = 0.0$	β_4	0.006 (0.001, 0.010)	0.937 (0.922, 0.952)
$\omega_1 = \omega_2 = \log(0.9), \delta_0 = \delta_1 = \delta_2 = \delta_3 = 0.00, \nu = 0.0$	β_5	0.051 (0.037, 0.064)	0.937 (0.922, 0.952)
$\omega_1 = \omega_2 = \log(0.9), \delta_0 = 0.00, \delta_1 = 2.50, \delta_2 = 5.00, \delta_3 = 6.25, \nu = -0.2$	β_1	0.010 (0.004, 0.015)	0.945 (0.931, 0.960)
$\omega_1 = \omega_2 = \log(0.9), \delta_0 = 0.00, \delta_1 = 2.50, \delta_2 = 5.00, \delta_3 = 6.25, \nu = -0.2$	β_2	0.000 (0.000, 0.000)	0.947 (0.933, 0.962)
$\omega_1 = \omega_2 = \log(0.9), \delta_0 = 0.00, \delta_1 = 2.50, \delta_2 = 5.00, \delta_3 = 6.25, \nu = -0.2$	β_3	0.001 (-0.001, 0.003)	0.948 (0.934, 0.962)
$\omega_1 = \omega_2 = \log(0.9), \delta_0 = 0.00, \delta_1 = 2.50, \delta_2 = 5.00, \delta_3 = 6.25, \nu = -0.2$	β_4	0.003 (-0.000, 0.006)	0.957 (0.944, 0.970)
$\omega_1 = \omega_2 = \log(0.9), \delta_0 = 0.00, \delta_1 = 2.50, \delta_2 = 5.00, \delta_3 = 6.25, \nu = -0.2$	β_5	0.031 (0.021, 0.042)	0.947 (0.933, 0.962)
$\omega_1 = \omega_2 = \log(0.9), \delta_0 = 0.00, \delta_1 = 2.50, \delta_2 = 5.00, \delta_3 = 6.25, \nu = 0.0$	β_1	0.009 (0.003, 0.014)	0.941 (0.926, 0.955)
$\omega_1 = \omega_2 = \log(0.9), \delta_0 = 0.00, \delta_1 = 2.50, \delta_2 = 5.00, \delta_3 = 6.25, \nu = 0.0$	β_2	0.000 (0.000, 0.000)	0.938 (0.923, 0.953)
$\omega_1 = \omega_2 = \log(0.9), \delta_0 = 0.00, \delta_1 = 2.50, \delta_2 = 5.00, \delta_3 = 6.25, \nu = 0.0$	β_3	0.000 (0.000, 0.000)	0.941 (0.926, 0.955)
$\omega_1 = \omega_2 = \log(0.9), \delta_0 = 0.00, \delta_1 = 2.50, \delta_2 = 5.00, \delta_3 = 6.25, \nu = 0.0$	β_4	0.001 (-0.001, 0.003)	0.947 (0.933, 0.961)
$\omega_1 = \omega_2 = \log(0.9), \delta_0 = 0.00, \delta_1 = 2.50, \delta_2 = 5.00, \delta_3 = 6.25, \nu = 0.0$	β_5	0.030 (0.019, 0.040)	0.947 (0.933, 0.961)
$\omega_1 = \omega_2 = \log(0.9), \delta_0 = 0.00, \delta_1 = 12.50, \delta_2 = 25.00, \delta_3 = 31.25, \nu = -0.2$	β_1	0.010 (0.004, 0.015)	0.948 (0.933, 0.962)

Table B18: Coverage probability of period effects on the longitudinal outcome for the general time on treatment effect parametrisation, with 95% confidence intervals based on Monte Carlo errors. LMM denotes the linear mixed model, while JM denotes the joint model. *(continued)*

Scenario	Parameter	LMM	JM
$\omega_1 = \omega_2 = \log(0.9)$, $\delta_0 = 0.00, \delta_1 = 12.50, \delta_2 = 25.00, \delta_3 = 31.25, \nu = -0.2$	β_2	0.000 (0.000, 0.000)	0.944 (0.930, 0.959)
$\omega_1 = \omega_2 = \log(0.9)$, $\delta_0 = 0.00, \delta_1 = 12.50, \delta_2 = 25.00, \delta_3 = 31.25, \nu = -0.2$	β_3	0.000 (0.000, 0.000)	0.947 (0.932, 0.961)
$\omega_1 = \omega_2 = \log(0.9)$, $\delta_0 = 0.00, \delta_1 = 12.50, \delta_2 = 25.00, \delta_3 = 31.25, \nu = -0.2$	β_4	0.003 (-0.000, 0.006)	0.939 (0.924, 0.954)
$\omega_1 = \omega_2 = \log(0.9)$, $\delta_0 = 0.00, \delta_1 = 12.50, \delta_2 = 25.00, \delta_3 = 31.25, \nu = -0.2$	β_5	0.028 (0.018, 0.038)	0.937 (0.922, 0.953)
$\omega_1 = \omega_2 = \log(0.9)$, $\delta_0 = 0.00, \delta_1 = 12.50, \delta_2 = 25.00, \delta_3 = 31.25, \nu = 0.0$	β_1	0.008 (0.002, 0.013)	0.954 (0.941, 0.967)
$\omega_1 = \omega_2 = \log(0.9)$, $\delta_0 = 0.00, \delta_1 = 12.50, \delta_2 = 25.00, \delta_3 = 31.25, \nu = 0.0$	β_2	0.000 (0.000, 0.000)	0.944 (0.930, 0.958)
$\omega_1 = \omega_2 = \log(0.9)$, $\delta_0 = 0.00, \delta_1 = 12.50, \delta_2 = 25.00, \delta_3 = 31.25, \nu = 0.0$	β_3	0.000 (0.000, 0.000)	0.946 (0.932, 0.960)
$\omega_1 = \omega_2 = \log(0.9)$, $\delta_0 = 0.00, \delta_1 = 12.50, \delta_2 = 25.00, \delta_3 = 31.25, \nu = 0.0$	β_4	0.005 (0.001, 0.009)	0.957 (0.944, 0.970)
$\omega_1 = \omega_2 = \log(0.9)$, $\delta_0 = 0.00, \delta_1 = 12.50, \delta_2 = 25.00, \delta_3 = 31.25, \nu = 0.0$	β_5	0.034 (0.023, 0.045)	0.951 (0.937, 0.964)
$\omega_1 = \omega_2 = \log(1.0)$, $\delta_0 = \delta_1 = \delta_2 = \delta_3 = 0.00, \nu = -0.2$	β_1	0.947 (0.933, 0.960)	0.952 (0.938, 0.965)
$\omega_1 = \omega_2 = \log(1.0)$, $\delta_0 = \delta_1 = \delta_2 = \delta_3 = 0.00, \nu = -0.2$	β_2	0.966 (0.955, 0.977)	0.967 (0.956, 0.978)
$\omega_1 = \omega_2 = \log(1.0)$, $\delta_0 = \delta_1 = \delta_2 = \delta_3 = 0.00, \nu = -0.2$	β_3	0.946 (0.932, 0.959)	0.953 (0.939, 0.966)
$\omega_1 = \omega_2 = \log(1.0)$, $\delta_0 = \delta_1 = \delta_2 = \delta_3 = 0.00, \nu = -0.2$	β_4	0.947 (0.933, 0.960)	0.950 (0.936, 0.963)
$\omega_1 = \omega_2 = \log(1.0)$, $\delta_0 = \delta_1 = \delta_2 = \delta_3 = 0.00, \nu = -0.2$	β_5	0.951 (0.938, 0.964)	0.952 (0.938, 0.965)
$\omega_1 = \omega_2 = \log(1.0)$, $\delta_0 = \delta_1 = \delta_2 = \delta_3 = 0.00, \nu = 0.0$	β_1	0.958 (0.946, 0.970)	0.961 (0.949, 0.973)
$\omega_1 = \omega_2 = \log(1.0)$, $\delta_0 = \delta_1 = \delta_2 = \delta_3 = 0.00, \nu = 0.0$	β_2	0.941 (0.927, 0.955)	0.948 (0.935, 0.962)
$\omega_1 = \omega_2 = \log(1.0)$, $\delta_0 = \delta_1 = \delta_2 = \delta_3 = 0.00, \nu = 0.0$	β_3	0.953 (0.941, 0.966)	0.956 (0.944, 0.969)
$\omega_1 = \omega_2 = \log(1.0)$, $\delta_0 = \delta_1 = \delta_2 = \delta_3 = 0.00, \nu = 0.0$	β_4	0.955 (0.943, 0.968)	0.959 (0.947, 0.971)
$\omega_1 = \omega_2 = \log(1.0)$, $\delta_0 = \delta_1 = \delta_2 = \delta_3 = 0.00, \nu = 0.0$	β_5	0.947 (0.933, 0.960)	0.948 (0.935, 0.962)
$\omega_1 = \omega_2 = \log(1.0)$, $\delta_0 = 0.00, \delta_1 = 2.50, \delta_2 = 5.00, \delta_3 = 6.25, \nu = -0.2$	β_1	0.939 (0.925, 0.953)	0.937 (0.922, 0.952)
$\omega_1 = \omega_2 = \log(1.0)$, $\delta_0 = 0.00, \delta_1 = 2.50, \delta_2 = 5.00, \delta_3 = 6.25, \nu = -0.2$	β_2	0.933 (0.918, 0.948)	0.938 (0.923, 0.952)
$\omega_1 = \omega_2 = \log(1.0)$, $\delta_0 = 0.00, \delta_1 = 2.50, \delta_2 = 5.00, \delta_3 = 6.25, \nu = -0.2$	β_3	0.931 (0.916, 0.947)	0.941 (0.926, 0.955)
$\omega_1 = \omega_2 = \log(1.0)$, $\delta_0 = 0.00, \delta_1 = 2.50, \delta_2 = 5.00, \delta_3 = 6.25, \nu = -0.2$	β_4	0.942 (0.928, 0.956)	0.946 (0.933, 0.960)
$\omega_1 = \omega_2 = \log(1.0)$, $\delta_0 = 0.00, \delta_1 = 2.50, \delta_2 = 5.00, \delta_3 = 6.25, \nu = -0.2$	β_5	0.944 (0.930, 0.958)	0.951 (0.938, 0.964)
$\omega_1 = \omega_2 = \log(1.0)$, $\delta_0 = 0.00, \delta_1 = 2.50, \delta_2 = 5.00, \delta_3 = 6.25, \nu = 0.0$	β_1	0.934 (0.919, 0.949)	0.943 (0.929, 0.958)
$\omega_1 = \omega_2 = \log(1.0)$, $\delta_0 = 0.00, \delta_1 = 2.50, \delta_2 = 5.00, \delta_3 = 6.25, \nu = 0.0$	β_2	0.940 (0.925, 0.954)	0.943 (0.929, 0.958)

Table B18: Coverage probability of period effects on the longitudinal outcome for the general time on treatment effect parametrisation, with 95% confidence intervals based on Monte Carlo errors. LMM denotes the linear mixed model, while JM denotes the joint model. *(continued)*

Scenario	Parameter	LMM	JM
$\omega_1 = \omega_2 = \log(1.0)$, $\delta_0 = 0.00, \delta_1 = 2.50, \delta_2 = 5.00, \delta_3 = 6.25, \nu = 0.0$	β_3	0.939 (0.924, 0.953)	0.942 (0.928, 0.957)
$\omega_1 = \omega_2 = \log(1.0)$, $\delta_0 = 0.00, \delta_1 = 2.50, \delta_2 = 5.00, \delta_3 = 6.25, \nu = 0.0$	β_4	0.938 (0.923, 0.953)	0.938 (0.923, 0.952)
$\omega_1 = \omega_2 = \log(1.0)$, $\delta_0 = 0.00, \delta_1 = 2.50, \delta_2 = 5.00, \delta_3 = 6.25, \nu = 0.0$	β_5	0.946 (0.932, 0.959)	0.953 (0.940, 0.966)
$\omega_1 = \omega_2 = \log(1.0)$, $\delta_0 = 0.00, \delta_1 = 12.50, \delta_2 = 25.00, \delta_3 = 31.25, \nu = -0.2$	β_1	0.947 (0.933, 0.960)	0.950 (0.937, 0.964)
$\omega_1 = \omega_2 = \log(1.0)$, $\delta_0 = 0.00, \delta_1 = 12.50, \delta_2 = 25.00, \delta_3 = 31.25, \nu = -0.2$	β_2	0.945 (0.931, 0.958)	0.951 (0.938, 0.964)
$\omega_1 = \omega_2 = \log(1.0)$, $\delta_0 = 0.00, \delta_1 = 12.50, \delta_2 = 25.00, \delta_3 = 31.25, \nu = -0.2$	β_3	0.955 (0.943, 0.968)	0.958 (0.946, 0.970)
$\omega_1 = \omega_2 = \log(1.0)$, $\delta_0 = 0.00, \delta_1 = 12.50, \delta_2 = 25.00, \delta_3 = 31.25, \nu = -0.2$	β_4	0.959 (0.947, 0.971)	0.960 (0.948, 0.972)
$\omega_1 = \omega_2 = \log(1.0)$, $\delta_0 = 0.00, \delta_1 = 12.50, \delta_2 = 25.00, \delta_3 = 31.25, \nu = -0.2$	β_5	0.961 (0.949, 0.973)	0.959 (0.947, 0.971)
$\omega_1 = \omega_2 = \log(1.0)$, $\delta_0 = 0.00, \delta_1 = 12.50, \delta_2 = 25.00, \delta_3 = 31.25, \nu = 0.0$	β_1	0.943 (0.929, 0.957)	0.944 (0.930, 0.958)
$\omega_1 = \omega_2 = \log(1.0)$, $\delta_0 = 0.00, \delta_1 = 12.50, \delta_2 = 25.00, \delta_3 = 31.25, \nu = 0.0$	β_2	0.948 (0.934, 0.961)	0.948 (0.935, 0.962)
$\omega_1 = \omega_2 = \log(1.0)$, $\delta_0 = 0.00, \delta_1 = 12.50, \delta_2 = 25.00, \delta_3 = 31.25, \nu = 0.0$	β_3	0.935 (0.920, 0.950)	0.945 (0.931, 0.959)
$\omega_1 = \omega_2 = \log(1.0)$, $\delta_0 = 0.00, \delta_1 = 12.50, \delta_2 = 25.00, \delta_3 = 31.25, \nu = 0.0$	β_4	0.954 (0.942, 0.967)	0.955 (0.942, 0.968)
$\omega_1 = \omega_2 = \log(1.0)$, $\delta_0 = 0.00, \delta_1 = 12.50, \delta_2 = 25.00, \delta_3 = 31.25, \nu = 0.0$	β_5	0.955 (0.943, 0.968)	0.959 (0.947, 0.971)
$\omega_1 = \omega_2 = \log(2.0)$, $\delta_0 = \delta_1 = \delta_2 = \delta_3 = 0.00, \nu = -0.2$	β_1	0.000 (0.000, 0.000)	0.523 (0.484, 0.562)
$\omega_1 = \omega_2 = \log(2.0)$, $\delta_0 = \delta_1 = \delta_2 = \delta_3 = 0.00, \nu = -0.2$	β_2	0.000 (0.000, 0.000)	0.551 (0.512, 0.590)
$\omega_1 = \omega_2 = \log(2.0)$, $\delta_0 = \delta_1 = \delta_2 = \delta_3 = 0.00, \nu = -0.2$	β_3	0.000 (0.000, 0.000)	0.572 (0.533, 0.610)
$\omega_1 = \omega_2 = \log(2.0)$, $\delta_0 = \delta_1 = \delta_2 = \delta_3 = 0.00, \nu = -0.2$	β_4	0.000 (0.000, 0.000)	0.638 (0.600, 0.675)
$\omega_1 = \omega_2 = \log(2.0)$, $\delta_0 = \delta_1 = \delta_2 = \delta_3 = 0.00, \nu = -0.2$	β_5	0.000 (0.000, 0.000)	0.734 (0.699, 0.768)
$\omega_1 = \omega_2 = \log(2.0)$, $\delta_0 = \delta_1 = \delta_2 = \delta_3 = 0.00, \nu = 0.0$	β_1	0.000 (0.000, 0.000)	0.520 (0.479, 0.560)
$\omega_1 = \omega_2 = \log(2.0)$, $\delta_0 = \delta_1 = \delta_2 = \delta_3 = 0.00, \nu = 0.0$	β_2	0.000 (0.000, 0.000)	0.525 (0.484, 0.565)
$\omega_1 = \omega_2 = \log(2.0)$, $\delta_0 = \delta_1 = \delta_2 = \delta_3 = 0.00, \nu = 0.0$	β_3	0.000 (0.000, 0.000)	0.544 (0.503, 0.584)
$\omega_1 = \omega_2 = \log(2.0)$, $\delta_0 = \delta_1 = \delta_2 = \delta_3 = 0.00, \nu = 0.0$	β_4	0.000 (0.000, 0.000)	0.593 (0.553, 0.633)
$\omega_1 = \omega_2 = \log(2.0)$, $\delta_0 = \delta_1 = \delta_2 = \delta_3 = 0.00, \nu = 0.0$	β_5	0.000 (0.000, 0.000)	0.747 (0.712, 0.782)
$\omega_1 = \omega_2 = \log(2.0)$, $\delta_0 = 0.00, \delta_1 = 2.50, \delta_2 = 5.00, \delta_3 = 6.25, \nu = -0.2$	β_1	0.000 (0.000, 0.000)	0.577 (0.537, 0.616)
$\omega_1 = \omega_2 = \log(2.0)$, $\delta_0 = 0.00, \delta_1 = 2.50, \delta_2 = 5.00, \delta_3 = 6.25, \nu = -0.2$	β_2	0.000 (0.000, 0.000)	0.563 (0.524, 0.603)
$\omega_1 = \omega_2 = \log(2.0)$, $\delta_0 = 0.00, \delta_1 = 2.50, \delta_2 = 5.00, \delta_3 = 6.25, \nu = -0.2$	β_3	0.000 (0.000, 0.000)	0.563 (0.524, 0.603)

Table B18: Coverage probability of period effects on the longitudinal outcome for the general time on treatment effect parametrisation, with 95% confidence intervals based on Monte Carlo errors. LMM denotes the linear mixed model, while JM denotes the joint model. *(continued)*

Scenario	Parameter	LMM	JM
$\omega_1 = \omega_2 = \log(2.0), \delta_0 = 0.00, \delta_1 = 2.50, \delta_2 = 5.00, \delta_3 = 6.25, \nu = -0.2$	β_4	0.000 (0.000, 0.000)	0.661 (0.623, 0.698)
$\omega_1 = \omega_2 = \log(2.0), \delta_0 = 0.00, \delta_1 = 2.50, \delta_2 = 5.00, \delta_3 = 6.25, \nu = -0.2$	β_5	0.000 (0.000, 0.000)	0.710 (0.674, 0.746)
$\omega_1 = \omega_2 = \log(2.0), \delta_0 = 0.00, \delta_1 = 2.50, \delta_2 = 5.00, \delta_3 = 6.25, \nu = 0.0$	β_1	0.000 (0.000, 0.000)	0.523 (0.483, 0.564)
$\omega_1 = \omega_2 = \log(2.0), \delta_0 = 0.00, \delta_1 = 2.50, \delta_2 = 5.00, \delta_3 = 6.25, \nu = 0.0$	β_2	0.000 (0.000, 0.000)	0.511 (0.471, 0.552)
$\omega_1 = \omega_2 = \log(2.0), \delta_0 = 0.00, \delta_1 = 2.50, \delta_2 = 5.00, \delta_3 = 6.25, \nu = 0.0$	β_3	0.000 (0.000, 0.000)	0.552 (0.512, 0.593)
$\omega_1 = \omega_2 = \log(2.0), \delta_0 = 0.00, \delta_1 = 2.50, \delta_2 = 5.00, \delta_3 = 6.25, \nu = 0.0$	β_4	0.000 (0.000, 0.000)	0.605 (0.566, 0.645)
$\omega_1 = \omega_2 = \log(2.0), \delta_0 = 0.00, \delta_1 = 2.50, \delta_2 = 5.00, \delta_3 = 6.25, \nu = 0.0$	β_5	0.000 (0.000, 0.000)	0.691 (0.654, 0.729)
$\omega_1 = \omega_2 = \log(2.0), \delta_0 = 0.00, \delta_1 = 12.50, \delta_2 = 25.00, \delta_3 = 31.25, \nu = -0.2$	β_1	0.000 (0.000, 0.000)	0.562 (0.523, 0.602)
$\omega_1 = \omega_2 = \log(2.0), \delta_0 = 0.00, \delta_1 = 12.50, \delta_2 = 25.00, \delta_3 = 31.25, \nu = -0.2$	β_2	0.000 (0.000, 0.000)	0.556 (0.516, 0.595)
$\omega_1 = \omega_2 = \log(2.0), \delta_0 = 0.00, \delta_1 = 12.50, \delta_2 = 25.00, \delta_3 = 31.25, \nu = -0.2$	β_3	0.000 (0.000, 0.000)	0.599 (0.560, 0.638)
$\omega_1 = \omega_2 = \log(2.0), \delta_0 = 0.00, \delta_1 = 12.50, \delta_2 = 25.00, \delta_3 = 31.25, \nu = -0.2$	β_4	0.000 (0.000, 0.000)	0.651 (0.613, 0.689)
$\omega_1 = \omega_2 = \log(2.0), \delta_0 = 0.00, \delta_1 = 12.50, \delta_2 = 25.00, \delta_3 = 31.25, \nu = -0.2$	β_5	0.000 (0.000, 0.000)	0.755 (0.721, 0.789)
$\omega_1 = \omega_2 = \log(2.0), \delta_0 = 0.00, \delta_1 = 12.50, \delta_2 = 25.00, \delta_3 = 31.25, \nu = 0.0$	β_1	0.000 (0.000, 0.000)	0.560 (0.521, 0.600)
$\omega_1 = \omega_2 = \log(2.0), \delta_0 = 0.00, \delta_1 = 12.50, \delta_2 = 25.00, \delta_3 = 31.25, \nu = 0.0$	β_2	0.000 (0.000, 0.000)	0.554 (0.514, 0.594)
$\omega_1 = \omega_2 = \log(2.0), \delta_0 = 0.00, \delta_1 = 12.50, \delta_2 = 25.00, \delta_3 = 31.25, \nu = 0.0$	β_3	0.000 (0.000, 0.000)	0.591 (0.551, 0.630)
$\omega_1 = \omega_2 = \log(2.0), \delta_0 = 0.00, \delta_1 = 12.50, \delta_2 = 25.00, \delta_3 = 31.25, \nu = 0.0$	β_4	0.000 (0.000, 0.000)	0.628 (0.589, 0.666)
**Additional scenarios:**			
$\omega_1 = \omega_2 = \log(2.0), \delta_0 = 0.00, \delta_1 = 12.50, \delta_2 = 25.00, \delta_3 = 31.25, \nu = 0.0$	β_5	0.000 (0.000, 0.000)	0.716 (0.680, 0.753)
$i = 3 \times 4$	β_1	0.223 (0.197, 0.248)	0.921 (0.903, 0.938)
$i = 3 \times 4$	β_2	0.089 (0.071, 0.106)	0.937 (0.922, 0.953)
$i = 3 \times 4$	β_3	0.097 (0.079, 0.115)	0.946 (0.931, 0.961)
$i = 3 \times 4$	β_4	0.165 (0.142, 0.188)	0.957 (0.944, 0.970)
$i = 3 \times 4$	β_5	0.321 (0.293, 0.350)	0.954 (0.940, 0.967)
$i = 3 \times 4, N = 100$	β_1	0.099 (0.081, 0.117)	0.931 (0.914, 0.947)
$i = 3 \times 4, N = 100$	β_2	0.016 (0.009, 0.024)	0.940 (0.924, 0.955)
$i = 3 \times 4, N = 100$	β_3	0.009 (0.003, 0.014)	0.944 (0.929, 0.959)

Table B18: Coverage probability of period effects on the longitudinal outcome for the general time on treatment effect parametrisation, with 95% confidence intervals based on Monte Carlo errors. LMM denotes the linear mixed model, while JM denotes the joint model. *(continued)*

Scenario	Parameter	LMM	JM
$i = 3 \times 4, N = 100$	β_4	0.031 (0.020, 0.041)	0.943 (0.928, 0.958)
$i = 3 \times 4, N = 100$	β_5	0.147 (0.125, 0.168)	0.953 (0.939, 0.967)
$\sigma^2_\alpha = 1, \sigma^2_\phi = 27.5$	β_1	0.296 (0.269, 0.324)	0.934 (0.918, 0.949)
$\sigma^2_\alpha = 1, \sigma^2_\phi = 27.5$	β_2	0.078 (0.062, 0.095)	0.953 (0.939, 0.966)
$\sigma^2_\alpha = 1, \sigma^2_\phi = 27.5$	β_3	0.059 (0.045, 0.074)	0.945 (0.931, 0.959)
$\sigma^2_\alpha = 1, \sigma^2_\phi = 27.5$	β_4	0.109 (0.090, 0.128)	0.950 (0.936, 0.963)
$\sigma^2_\alpha = 1, \sigma^2_\phi = 27.5$	β_5	0.245 (0.219, 0.271)	0.949 (0.935, 0.962)
$\sigma^2_\alpha = 4, \sigma^2_\phi = 110$	β_1	0.000 (0.000, 0.000)	0.950 (0.936, 0.964)
$\sigma^2_\alpha = 4, \sigma^2_\phi = 110$	β_2	0.000 (0.000, 0.000)	0.940 (0.925, 0.955)
$\sigma^2_\alpha = 4, \sigma^2_\phi = 110$	β_3	0.000 (0.000, 0.000)	0.949 (0.934, 0.963)
$\sigma^2_\alpha = 4, \sigma^2_\phi = 110$	β_4	0.000 (0.000, 0.000)	0.949 (0.934, 0.963)
$\sigma^2_\alpha = 4, \sigma^2_\phi = 110$	β_5	0.001 (-0.001, 0.003)	0.941 (0.926, 0.956)

Table B19: Bias of ICCs for the general time on treatment effect parametrisation, with 95% confidence intervals based on Monte Carlo errors. LMM denotes the linear mixed model, while JM denotes the joint model. Statistically significant biases are highlighted in bold.

Scenario	Parameter	LMM	JM
**Main scenarios:**			
$\omega_1 = \omega_2 = \log(0.5)$, $\delta = 0.0$, $\nu = -0.2$	ρ_a	**-0.224 (-0.225, -0.223)**	**-0.025 (-0.027, -0.024)**
$\omega_1 = \omega_2 = \log(0.5)$, $\delta = 0.0$, $\nu = -0.2$	ρ_d	**-0.015 (-0.016, -0.015)**	**-0.008 (-0.008, -0.007)**
$\omega_1 = \omega_2 = \log(0.5)$, $\delta = 0.0$, $\nu = 0.0$	ρ_a	**-0.226 (-0.227, -0.225)**	**-0.026 (-0.028, -0.025)**
$\omega_1 = \omega_2 = \log(0.5)$, $\delta = 0.0$, $\nu = 0.0$	ρ_d	**-0.015 (-0.016, -0.015)**	**-0.007 (-0.008, -0.007)**
$\omega_1 = \omega_2 = \log(0.5)$, $\delta = 5.0$, $\nu = -0.2$	ρ_a	**-0.225 (-0.226, -0.223)**	**-0.025 (-0.027, -0.024)**
$\omega_1 = \omega_2 = \log(0.5)$, $\delta = 5.0$, $\nu = -0.2$	ρ_d	**-0.015 (-0.016, -0.015)**	**-0.007 (-0.008, -0.007)**
$\omega_1 = \omega_2 = \log(0.5)$, $\delta = 5.0$, $\nu = 0.0$	ρ_a	**-0.226 (-0.227, -0.225)**	**-0.026 (-0.028, -0.025)**
$\omega_1 = \omega_2 = \log(0.5)$, $\delta = 5.0$, $\nu = 0.0$	ρ_d	**-0.015 (-0.016, -0.015)**	**-0.008 (-0.008, -0.007)**
$\omega_1 = \omega_2 = \log(0.5)$, $\delta = 25$, $\nu = -0.2$	ρ_a	**-0.224 (-0.225, -0.223)**	**-0.025 (-0.026, -0.023)**
$\omega_1 = \omega_2 = \log(0.5)$, $\delta = 25$, $\nu = -0.2$	ρ_d	**-0.015 (-0.016, -0.015)**	**-0.008 (-0.008, -0.007)**
$\omega_1 = \omega_2 = \log(0.5)$, $\delta = 25$, $\nu = 0.0$	ρ_a	**-0.226 (-0.227, -0.224)**	**-0.025 (-0.027, -0.024)**
$\omega_1 = \omega_2 = \log(0.5)$, $\delta = 25$, $\nu = 0.0$	ρ_d	**-0.015 (-0.015, -0.015)**	**-0.008 (-0.008, -0.007)**
$\omega_1 = \omega_2 = \log(0.9)$, $\delta = 0.0$, $\nu = -0.2$	ρ_a	**-0.053 (-0.054, -0.052)**	-0.000 (-0.001, 0.001)
$\omega_1 = \omega_2 = \log(0.9)$, $\delta = 0.0$, $\nu = -0.2$	ρ_d	**-0.006 (-0.006, -0.005)**	**-0.001 (-0.001, -0.000)**
$\omega_1 = \omega_2 = \log(0.9)$, $\delta = 0.0$, $\nu = 0.0$	ρ_a	**-0.054 (-0.055, -0.053)**	-0.000 (-0.001, 0.001)
$\omega_1 = \omega_2 = \log(0.9)$, $\delta = 0.0$, $\nu = 0.0$	ρ_d	**-0.006 (-0.006, -0.005)**	**-0.001 (-0.002, -0.000)**
$\omega_1 = \omega_2 = \log(0.9)$, $\delta = 5.0$, $\nu = -0.2$	ρ_a	**-0.053 (-0.054, -0.052)**	0.001 (-0.000, 0.002)
$\omega_1 = \omega_2 = \log(0.9)$, $\delta = 5.0$, $\nu = -0.2$	ρ_d	**-0.005 (-0.006, -0.005)**	-0.000 (-0.001, 0.000)
$\omega_1 = \omega_2 = \log(0.9)$, $\delta = 5.0$, $\nu = 0.0$	ρ_a	**-0.054 (-0.055, -0.053)**	0.000 (-0.001, 0.001)
$\omega_1 = \omega_2 = \log(0.9)$, $\delta = 5.0$, $\nu = 0.0$	ρ_d	**-0.005 (-0.006, -0.005)**	**-0.001 (-0.001, -0.000)**
$\omega_1 = \omega_2 = \log(0.9)$, $\delta = 25$, $\nu = -0.2$	ρ_a	**-0.054 (-0.055, -0.053)**	0.000 (-0.001, 0.001)
$\omega_1 = \omega_2 = \log(0.9)$, $\delta = 25$, $\nu = -0.2$	ρ_d	**-0.005 (-0.006, -0.005)**	-0.000 (-0.001, 0.000)
$\omega_1 = \omega_2 = \log(0.9)$, $\delta = 25$, $\nu = 0.0$	ρ_a	**-0.053 (-0.054, -0.052)**	**0.001 (0.000, 0.002)**
$\omega_1 = \omega_2 = \log(0.9)$, $\delta = 25$, $\nu = 0.0$	ρ_d	**-0.006 (-0.006, -0.005)**	**-0.001 (-0.002, -0.001)**
$\omega_1 = \omega_2 = \log(1.0)$, $\delta = 0.0$, $\nu = -0.2$	ρ_a	-0.001 (-0.002, 0.000)	-0.000 (-0.001, 0.000)
$\omega_1 = \omega_2 = \log(1.0)$, $\delta = 0.0$, $\nu = -0.2$	ρ_d	**-0.001 (-0.001, -0.000)**	-0.000 (-0.001, 0.000)
$\omega_1 = \omega_2 = \log(1.0)$, $\delta = 0.0$, $\nu = 0.0$	ρ_a	-0.001 (-0.002, 0.000)	-0.001 (-0.001, 0.000)
$\omega_1 = \omega_2 = \log(1.0)$, $\delta = 0.0$, $\nu = 0.0$	ρ_d	**-0.001 (-0.002, -0.001)**	**-0.001 (-0.001, -0.000)**
$\omega_1 = \omega_2 = \log(1.0)$, $\delta = 5.0$, $\nu = -0.2$	ρ_a	-0.000 (-0.001, 0.000)	-0.000 (-0.001, 0.001)
$\omega_1 = \omega_2 = \log(1.0)$, $\delta = 5.0$, $\nu = -0.2$	ρ_d	**-0.002 (-0.002, -0.001)**	**-0.001 (-0.002, -0.001)**
$\omega_1 = \omega_2 = \log(1.0)$, $\delta = 5.0$, $\nu = 0.0$	ρ_a	**-0.001 (-0.002, -0.000)**	-0.001 (-0.002, 0.000)
$\omega_1 = \omega_2 = \log(1.0)$, $\delta = 5.0$, $\nu = 0.0$	ρ_d	**-0.001 (-0.002, -0.001)**	**-0.001 (-0.001, -0.000)**
$\omega_1 = \omega_2 = \log(1.0)$, $\delta = 25$, $\nu = -0.2$	ρ_a	-0.000 (-0.001, 0.001)	-0.000 (-0.001, 0.001)
$\omega_1 = \omega_2 = \log(1.0)$, $\delta = 25$, $\nu = -0.2$	ρ_d	**-0.001 (-0.002, -0.001)**	**-0.001 (-0.002, -0.000)**
$\omega_1 = \omega_2 = \log(1.0)$, $\delta = 25$, $\nu = 0.0$	ρ_a	0.000 (-0.001, 0.001)	0.000 (-0.001, 0.001)
$\omega_1 = \omega_2 = \log(1.0)$, $\delta = 25$, $\nu = 0.0$	ρ_d	**-0.001 (-0.002, -0.001)**	**-0.001 (-0.001, -0.000)**

Table B19: Bias of ICCs for the general time on treatment effect parametrisation, with 95% confidence intervals based on Monte Carlo errors. LMM denotes the linear mixed model, while JM denotes the joint model. Statistically significant biases are highlighted in bold. *(continued)*

Scenario	Parameter	LMM	JM
$\omega_1 = \omega_2 = \log(2.0), \delta = 0.0, \nu = -0.2$	ρ_a	**-0.225 (-0.226, -0.224)**	**-0.025 (-0.027, -0.023)**
$\omega_1 = \omega_2 = \log(2.0), \delta = 0.0, \nu = -0.2$	ρ_d	**-0.015 (-0.016, -0.015)**	**-0.007 (-0.008, -0.007)**
$\omega_1 = \omega_2 = \log(2.0), \delta = 0.0, \nu = 0.0$	ρ_a	**-0.226 (-0.227, -0.224)**	**-0.026 (-0.028, -0.024)**
$\omega_1 = \omega_2 = \log(2.0), \delta = 0.0, \nu = 0.0$	ρ_d	**-0.015 (-0.016, -0.015)**	**-0.007 (-0.008, -0.007)**
$\omega_1 = \omega_2 = \log(2.0), \delta = 5.0, \nu = -0.2$	ρ_a	**-0.225 (-0.226, -0.224)**	**-0.026 (-0.028, -0.025)**
$\omega_1 = \omega_2 = \log(2.0), \delta = 5.0, \nu = -0.2$	ρ_d	**-0.015 (-0.015, -0.015)**	**-0.007 (-0.008, -0.007)**
$\omega_1 = \omega_2 = \log(2.0), \delta = 5.0, \nu = 0.0$	ρ_a	**-0.226 (-0.227, -0.224)**	**-0.027 (-0.028, -0.025)**
$\omega_1 = \omega_2 = \log(2.0), \delta = 5.0, \nu = 0.0$	ρ_d	**-0.015 (-0.016, -0.015)**	**-0.007 (-0.008, -0.007)**
$\omega_1 = \omega_2 = \log(2.0), \delta = 25, \nu = -0.2$	ρ_a	**-0.226 (-0.227, -0.224)**	**-0.027 (-0.028, -0.025)**
$\omega_1 = \omega_2 = \log(2.0), \delta = 25, \nu = -0.2$	ρ_d	**-0.015 (-0.016, -0.015)**	**-0.008 (-0.008, -0.007)**
$\omega_1 = \omega_2 = \log(2.0), \delta = 25, \nu = 0.0$	ρ_a	**-0.226 (-0.227, -0.224)**	**-0.026 (-0.028, -0.025)**
$\omega_1 = \omega_2 = \log(2.0), \delta = 25, \nu = 0.0$	ρ_d	**-0.015 (-0.016, -0.015)**	**-0.007 (-0.008, -0.007)**
**Additional scenarios:**			
$i = 3 \times 4$	ρ_a	**-0.052 (-0.054, -0.051)**	**0.002 (0.000, 0.004)**
$i = 3 \times 4$	ρ_d	**-0.007 (-0.008, -0.007)**	**-0.002 (-0.003, -0.001)**
$i = 3 \times 4, N = 100$	ρ_a	**-0.054 (-0.055, -0.053)**	-0.001 (-0.002, 0.000)
$i = 3 \times 4, N = 100$	ρ_d	**-0.007 (-0.007, -0.006)**	**-0.002 (-0.003, -0.001)**
$\sigma^2_\alpha = 1, \sigma^2_\phi = 27.5$	ρ_a	**-0.033 (-0.034, -0.032)**	0.000 (-0.001, 0.001)
$\sigma^2_\alpha = 1, \sigma^2_\phi = 27.5$	ρ_d	**-0.002 (-0.003, -0.002)**	0.000 (-0.000, 0.001)
$\sigma^2_\alpha = 4, \sigma^2_\phi = 110$	ρ_a	**-0.065 (-0.066, -0.064)**	0.001 (-0.000, 0.001)
$\sigma^2_\alpha = 4, \sigma^2_\phi = 110$	ρ_d	**-0.009 (-0.009, -0.008)**	**-0.001 (-0.002, -0.000)**

Table B20: Relative bias of ICCs for the general time on treatment effect parametrisation, with 95% confidence intervals based on Monte Carlo errors. LMM denotes the linear mixed model, while JM denotes the joint model. Statistically significant biases are highlighted in bold.

Scenario	Parameter	LMM	JM
**Main scenarios:**			
$\omega_1 = \omega_2 = \log(0.5), \delta = 0.0, \nu = -0.2$	ρ_a	**-0.381 (-0.383, -0.379)**	**-0.043 (-0.046, -0.040)**
$\omega_1 = \omega_2 = \log(0.5), \delta = 0.0, \nu = -0.2$	ρ_d	**-0.739 (-0.754, -0.723)**	**-0.371 (-0.393, -0.348)**
$\omega_1 = \omega_2 = \log(0.5), \delta = 0.0, \nu = 0.0$	ρ_a	**-0.385 (-0.387, -0.383)**	**-0.045 (-0.047, -0.042)**
$\omega_1 = \omega_2 = \log(0.5), \delta = 0.0, \nu = 0.0$	ρ_d	**-0.745 (-0.760, -0.730)**	**-0.362 (-0.384, -0.340)**
$\omega_1 = \omega_2 = \log(0.5), \delta = 5.0, \nu = -0.2$	ρ_a	**-0.382 (-0.384, -0.380)**	**-0.043 (-0.046, -0.041)**
$\omega_1 = \omega_2 = \log(0.5), \delta = 5.0, \nu = -0.2$	ρ_d	**-0.740 (-0.755, -0.725)**	**-0.355 (-0.377, -0.332)**
$\omega_1 = \omega_2 = \log(0.5), \delta = 5.0, \nu = 0.0$	ρ_a	**-0.385 (-0.387, -0.383)**	**-0.044 (-0.047, -0.042)**

Table B20: Relative bias of ICCs for the general time on treatment effect parametrisation, with 95% confidence intervals based on Monte Carlo errors. LMM denotes the linear mixed model, while JM denotes the joint model. Statistically significant biases are highlighted in bold. *(continued)*

Scenario	Parameter	LMM	JM
$\omega_1 = \omega_2 = \log(0.5)$, $\delta = 5.0$, $\nu = 0.0$	ρ_d	**-0.750 (-0.766, -0.735)**	**-0.371 (-0.394, -0.348)**
$\omega_1 = \omega_2 = \log(0.5)$, $\delta = 25$, $\nu = -0.2$	ρ_a	**-0.381 (-0.383, -0.379)**	**-0.043 (-0.045, -0.040)**
$\omega_1 = \omega_2 = \log(0.5)$, $\delta = 25$, $\nu = -0.2$	ρ_d	**-0.737 (-0.752, -0.723)**	**-0.368 (-0.390, -0.347)**
$\omega_1 = \omega_2 = \log(0.5)$, $\delta = 25$, $\nu = 0.0$	ρ_a	**-0.384 (-0.386, -0.382)**	**-0.043 (-0.046, -0.041)**
$\omega_1 = \omega_2 = \log(0.5)$, $\delta = 25$, $\nu = 0.0$	ρ_d	**-0.734 (-0.750, -0.718)**	**-0.374 (-0.397, -0.351)**
$\omega_1 = \omega_2 = \log(0.9)$, $\delta = 0.0$, $\nu = -0.2$	ρ_a	**-0.090 (-0.092, -0.089)**	-0.000 (-0.002, 0.002)
$\omega_1 = \omega_2 = \log(0.9)$, $\delta = 0.0$, $\nu = -0.2$	ρ_d	**-0.274 (-0.298, -0.249)**	**-0.041 (-0.069, -0.012)**
$\omega_1 = \omega_2 = \log(0.9)$, $\delta = 0.0$, $\nu = 0.0$	ρ_a	**-0.093 (-0.094, -0.091)**	-0.000 (-0.002, 0.002)
$\omega_1 = \omega_2 = \log(0.9)$, $\delta = 0.0$, $\nu = 0.0$	ρ_d	**-0.275 (-0.299, -0.251)**	**-0.046 (-0.073, -0.018)**
$\omega_1 = \omega_2 = \log(0.9)$, $\delta = 5.0$, $\nu = -0.2$	ρ_a	**-0.089 (-0.091, -0.088)**	0.002 (-0.000, 0.004)
$\omega_1 = \omega_2 = \log(0.9)$, $\delta = 5.0$, $\nu = -0.2$	ρ_d	**-0.255 (-0.280, -0.230)**	-0.015 (-0.044, 0.014)
$\omega_1 = \omega_2 = \log(0.9)$, $\delta = 5.0$, $\nu = 0.0$	ρ_a	**-0.091 (-0.093, -0.090)**	0.000 (-0.001, 0.002)
$\omega_1 = \omega_2 = \log(0.9)$, $\delta = 5.0$, $\nu = 0.0$	ρ_d	**-0.257 (-0.282, -0.233)**	**-0.030 (-0.058, -0.002)**
$\omega_1 = \omega_2 = \log(0.9)$, $\delta = 25$, $\nu = -0.2$	ρ_a	**-0.091 (-0.093, -0.089)**	0.000 (-0.002, 0.002)
$\omega_1 = \omega_2 = \log(0.9)$, $\delta = 25$, $\nu = -0.2$	ρ_d	**-0.265 (-0.291, -0.240)**	-0.020 (-0.050, 0.009)
$\omega_1 = \omega_2 = \log(0.9)$, $\delta = 25$, $\nu = 0.0$	ρ_a	**-0.091 (-0.092, -0.089)**	**0.002 (0.000, 0.004)**
$\omega_1 = \omega_2 = \log(0.9)$, $\delta = 25$, $\nu = 0.0$	ρ_d	**-0.286 (-0.310, -0.263)**	**-0.064 (-0.092, -0.036)**
$\omega_1 = \omega_2 = \log(1.0)$, $\delta = 0.0$, $\nu = -0.2$	ρ_a	-0.001 (-0.003, 0.000)	-0.001 (-0.002, 0.001)
$\omega_1 = \omega_2 = \log(1.0)$, $\delta = 0.0$, $\nu = -0.2$	ρ_d	**-0.039 (-0.068, -0.011)**	-0.019 (-0.047, 0.010)
$\omega_1 = \omega_2 = \log(1.0)$, $\delta = 0.0$, $\nu = 0.0$	ρ_a	-0.001 (-0.003, 0.000)	-0.001 (-0.003, 0.001)
$\omega_1 = \omega_2 = \log(1.0)$, $\delta = 0.0$, $\nu = 0.0$	ρ_d	**-0.058 (-0.085, -0.031)**	**-0.045 (-0.072, -0.018)**
$\omega_1 = \omega_2 = \log(1.0)$, $\delta = 5.0$, $\nu = -0.2$	ρ_a	-0.001 (-0.002, 0.001)	-0.000 (-0.002, 0.001)
$\omega_1 = \omega_2 = \log(1.0)$, $\delta = 5.0$, $\nu = -0.2$	ρ_d	**-0.078 (-0.106, -0.051)**	**-0.069 (-0.096, -0.041)**
$\omega_1 = \omega_2 = \log(1.0)$, $\delta = 5.0$, $\nu = 0.0$	ρ_a	**-0.002 (-0.003, -0.000)**	-0.001 (-0.003, 0.000)
$\omega_1 = \omega_2 = \log(1.0)$, $\delta = 5.0$, $\nu = 0.0$	ρ_d	**-0.061 (-0.088, -0.034)**	**-0.045 (-0.071, -0.018)**
$\omega_1 = \omega_2 = \log(1.0)$, $\delta = 25$, $\nu = -0.2$	ρ_a	-0.001 (-0.002, 0.001)	-0.000 (-0.002, 0.001)
$\omega_1 = \omega_2 = \log(1.0)$, $\delta = 25$, $\nu = -0.2$	ρ_d	**-0.059 (-0.086, -0.031)**	**-0.047 (-0.074, -0.019)**
$\omega_1 = \omega_2 = \log(1.0)$, $\delta = 25$, $\nu = 0.0$	ρ_a	0.000 (-0.001, 0.002)	0.000 (-0.001, 0.002)
$\omega_1 = \omega_2 = \log(1.0)$, $\delta = 25$, $\nu = 0.0$	ρ_d	**-0.059 (-0.087, -0.031)**	**-0.041 (-0.069, -0.013)**
$\omega_1 = \omega_2 = \log(2.0)$, $\delta = 0.0$, $\nu = -0.2$	ρ_a	**-0.383 (-0.385, -0.381)**	**-0.043 (-0.045, -0.040)**
$\omega_1 = \omega_2 = \log(2.0)$, $\delta = 0.0$, $\nu = -0.2$	ρ_d	**-0.746 (-0.761, -0.731)**	**-0.363 (-0.385, -0.340)**
$\omega_1 = \omega_2 = \log(2.0)$, $\delta = 0.0$, $\nu = 0.0$	ρ_a	**-0.384 (-0.386, -0.382)**	**-0.044 (-0.047, -0.042)**
$\omega_1 = \omega_2 = \log(2.0)$, $\delta = 0.0$, $\nu = 0.0$	ρ_d	**-0.746 (-0.761, -0.731)**	**-0.360 (-0.384, -0.337)**
$\omega_1 = \omega_2 = \log(2.0)$, $\delta = 5.0$, $\nu = -0.2$	ρ_a	**-0.383 (-0.385, -0.380)**	**-0.044 (-0.047, -0.042)**
$\omega_1 = \omega_2 = \log(2.0)$, $\delta = 5.0$, $\nu = -0.2$	ρ_d	**-0.731 (-0.747, -0.716)**	**-0.354 (-0.378, -0.330)**
$\omega_1 = \omega_2 = \log(2.0)$, $\delta = 5.0$, $\nu = 0.0$	ρ_a	**-0.384 (-0.386, -0.382)**	**-0.045 (-0.048, -0.043)**
$\omega_1 = \omega_2 = \log(2.0)$, $\delta = 5.0$, $\nu = 0.0$	ρ_d	**-0.741 (-0.756, -0.726)**	**-0.359 (-0.382, -0.336)**
$\omega_1 = \omega_2 = \log(2.0)$, $\delta = 25$, $\nu = -0.2$	ρ_a	**-0.384 (-0.386, -0.382)**	**-0.045 (-0.048, -0.043)**

Table B20: Relative bias of ICCs for the general time on treatment effect parametrisation, with 95% confidence intervals based on Monte Carlo errors. LMM denotes the linear mixed model, while JM denotes the joint model. Statistically significant biases are highlighted in bold. *(continued)*

Scenario	Parameter	LMM	JM
$\omega_1 = \omega_2 = \log(2.0), \delta = 25, \nu = -0.2$	ρ_d	**-0.740 (-0.755, -0.724)**	**-0.379 (-0.403, -0.355)**
$\omega_1 = \omega_2 = \log(2.0), \delta = 25, \nu = 0.0$	ρ_a	**-0.384 (-0.386, -0.382)**	**-0.045 (-0.047, -0.042)**
$\omega_1 = \omega_2 = \log(2.0), \delta = 25, \nu = 0.0$	ρ_d	**-0.742 (-0.757, -0.727)**	**-0.346 (-0.369, -0.322)**
**Additional scenarios:**			
$i = 3 \times 4$	ρ_a	**-0.089 (-0.092, -0.086)**	**0.003 (0.000, 0.006)**
$i = 3 \times 4$	ρ_d	**-0.354 (-0.390, -0.318)**	**-0.079 (-0.123, -0.036)**
$i = 3 \times 4, N = 100$	ρ_a	**-0.092 (-0.093, -0.090)**	-0.002 (-0.004, 0.000)
$i = 3 \times 4, N = 100$	ρ_d	**-0.323 (-0.351, -0.295)**	**-0.095 (-0.129, -0.061)**
$\sigma^2_\alpha = 1, \sigma^2_\phi = 27.5$	ρ_a	**-0.080 (-0.083, -0.078)**	0.001 (-0.002, 0.003)
$\sigma^2_\alpha = 1, \sigma^2_\phi = 27.5$	ρ_d	**-0.166 (-0.194, -0.137)**	0.009 (-0.022, 0.039)
$\sigma^2_\alpha = 4, \sigma^2_\phi = 110$	ρ_a	**-0.088 (-0.089, -0.087)**	0.001 (-0.000, 0.002)
$\sigma^2_\alpha = 4, \sigma^2_\phi = 110$	ρ_d	**-0.335 (-0.358, -0.313)**	**-0.036 (-0.064, -0.009)**

Table B21: Coverage probability of ICCs for the general time on treatment effect parametrisation, with 95% confidence intervals based on Monte Carlo errors. LMM denotes the linear mixed model, while JM denotes the joint model.

Scenario	Parameter	LMM	JM
**Main scenarios:**			
$\omega_1 = \omega_2 = \log(0.5), \delta = 0.0, \nu = -0.2$	ρ_a	0.000 (0.000, 0.000)	0.761 (0.729, 0.794)
$\omega_1 = \omega_2 = \log(0.5), \delta = 0.0, \nu = -0.2$	ρ_d	0.286 (0.258, 0.314)	0.677 (0.642, 0.713)
$\omega_1 = \omega_2 = \log(0.5), \delta = 0.0, \nu = 0.0$	ρ_a	0.000 (0.000, 0.000)	0.765 (0.733, 0.797)
$\omega_1 = \omega_2 = \log(0.5), \delta = 0.0, \nu = 0.0$	ρ_d	0.274 (0.246, 0.301)	0.685 (0.650, 0.720)
$\omega_1 = \omega_2 = \log(0.5), \delta = 5.0, \nu = -0.2$	ρ_a	0.000 (0.000, 0.000)	0.764 (0.732, 0.796)
$\omega_1 = \omega_2 = \log(0.5), \delta = 5.0, \nu = -0.2$	ρ_d	0.266 (0.239, 0.293)	0.713 (0.679, 0.747)
$\omega_1 = \omega_2 = \log(0.5), \delta = 5.0, \nu = 0.0$	ρ_a	0.000 (0.000, 0.000)	0.740 (0.706, 0.773)
$\omega_1 = \omega_2 = \log(0.5), \delta = 5.0, \nu = 0.0$	ρ_d	0.257 (0.230, 0.284)	0.658 (0.621, 0.694)
$\omega_1 = \omega_2 = \log(0.5), \delta = 25, \nu = -0.2$	ρ_a	0.000 (0.000, 0.000)	0.781 (0.749, 0.812)
$\omega_1 = \omega_2 = \log(0.5), \delta = 25, \nu = -0.2$	ρ_d	0.268 (0.240, 0.295)	0.689 (0.654, 0.724)
$\omega_1 = \omega_2 = \log(0.5), \delta = 25, \nu = 0.0$	ρ_a	0.000 (0.000, 0.000)	0.757 (0.724, 0.790)
$\omega_1 = \omega_2 = \log(0.5), \delta = 25, \nu = 0.0$	ρ_d	0.295 (0.267, 0.323)	0.666 (0.630, 0.703)
$\omega_1 = \omega_2 = \log(0.9), \delta = 0.0, \nu = -0.2$	ρ_a	0.076 (0.060, 0.092)	0.961 (0.949, 0.973)
$\omega_1 = \omega_2 = \log(0.9), \delta = 0.0, \nu = -0.2$	ρ_d	0.790 (0.765, 0.815)	0.908 (0.889, 0.926)
$\omega_1 = \omega_2 = \log(0.9), \delta = 0.0, \nu = 0.0$	ρ_a	0.075 (0.059, 0.090)	0.945 (0.931, 0.959)
$\omega_1 = \omega_2 = \log(0.9), \delta = 0.0, \nu = 0.0$	ρ_d	0.810 (0.786, 0.834)	0.917 (0.899, 0.934)

Table B21: Coverage probability of ICCs for the general time on treatment effect parametrisation, with 95% confidence intervals based on Monte Carlo errors. LMM denotes the linear mixed model, while JM denotes the joint model. *(continued)*

Scenario	Parameter	LMM	JM
$\omega_1 = \omega_2 = \log(0.9)$, $\delta = 5.0$, $\nu = -0.2$	ρ_a	0.088 (0.071, 0.105)	0.948 (0.934, 0.962)
$\omega_1 = \omega_2 = \log(0.9)$, $\delta = 5.0$, $\nu = -0.2$	ρ_d	0.806 (0.782, 0.830)	0.929 (0.913, 0.946)
$\omega_1 = \omega_2 = \log(0.9)$, $\delta = 5.0$, $\nu = 0.0$	ρ_a	0.082 (0.066, 0.099)	0.957 (0.944, 0.969)
$\omega_1 = \omega_2 = \log(0.9)$, $\delta = 5.0$, $\nu = 0.0$	ρ_d	0.808 (0.784, 0.832)	0.915 (0.898, 0.933)
$\omega_1 = \omega_2 = \log(0.9)$, $\delta = 25$, $\nu = -0.2$	ρ_a	0.072 (0.056, 0.087)	0.951 (0.937, 0.964)
$\omega_1 = \omega_2 = \log(0.9)$, $\delta = 25$, $\nu = -0.2$	ρ_d	0.781 (0.756, 0.806)	0.913 (0.895, 0.931)
$\omega_1 = \omega_2 = \log(0.9)$, $\delta = 25$, $\nu = 0.0$	ρ_a	0.075 (0.059, 0.091)	0.951 (0.937, 0.964)
$\omega_1 = \omega_2 = \log(0.9)$, $\delta = 25$, $\nu = 0.0$	ρ_d	0.808 (0.784, 0.832)	0.908 (0.890, 0.926)
$\omega_1 = \omega_2 = \log(1.0)$, $\delta = 0.0$, $\nu = -0.2$	ρ_a	0.951 (0.938, 0.964)	0.953 (0.939, 0.966)
$\omega_1 = \omega_2 = \log(1.0)$, $\delta = 0.0$, $\nu = -0.2$	ρ_d	0.906 (0.888, 0.923)	0.916 (0.899, 0.933)
$\omega_1 = \omega_2 = \log(1.0)$, $\delta = 0.0$, $\nu = 0.0$	ρ_a	0.935 (0.920, 0.950)	0.931 (0.915, 0.946)
$\omega_1 = \omega_2 = \log(1.0)$, $\delta = 0.0$, $\nu = 0.0$	ρ_d	0.905 (0.887, 0.922)	0.915 (0.898, 0.932)
$\omega_1 = \omega_2 = \log(1.0)$, $\delta = 5.0$, $\nu = -0.2$	ρ_a	0.944 (0.930, 0.958)	0.946 (0.933, 0.960)
$\omega_1 = \omega_2 = \log(1.0)$, $\delta = 5.0$, $\nu = -0.2$	ρ_d	0.898 (0.880, 0.916)	0.905 (0.887, 0.923)
$\omega_1 = \omega_2 = \log(1.0)$, $\delta = 5.0$, $\nu = 0.0$	ρ_a	0.944 (0.930, 0.958)	0.942 (0.928, 0.957)
$\omega_1 = \omega_2 = \log(1.0)$, $\delta = 5.0$, $\nu = 0.0$	ρ_d	0.904 (0.886, 0.921)	0.914 (0.897, 0.931)
$\omega_1 = \omega_2 = \log(1.0)$, $\delta = 25$, $\nu = -0.2$	ρ_a	0.954 (0.941, 0.967)	0.953 (0.940, 0.966)
$\omega_1 = \omega_2 = \log(1.0)$, $\delta = 25$, $\nu = -0.2$	ρ_d	0.906 (0.889, 0.924)	0.912 (0.895, 0.930)
$\omega_1 = \omega_2 = \log(1.0)$, $\delta = 25$, $\nu = 0.0$	ρ_a	0.950 (0.936, 0.963)	0.945 (0.931, 0.959)
$\omega_1 = \omega_2 = \log(1.0)$, $\delta = 25$, $\nu = 0.0$	ρ_d	0.890 (0.871, 0.908)	0.905 (0.887, 0.923)
$\omega_1 = \omega_2 = \log(2.0)$, $\delta = 0.0$, $\nu = -0.2$	ρ_a	0.000 (0.000, 0.000)	0.756 (0.722, 0.789)
$\omega_1 = \omega_2 = \log(2.0)$, $\delta = 0.0$, $\nu = -0.2$	ρ_d	0.268 (0.241, 0.295)	0.680 (0.644, 0.717)
$\omega_1 = \omega_2 = \log(2.0)$, $\delta = 0.0$, $\nu = 0.0$	ρ_a	0.000 (0.000, 0.000)	0.735 (0.699, 0.771)
$\omega_1 = \omega_2 = \log(2.0)$, $\delta = 0.0$, $\nu = 0.0$	ρ_d	0.270 (0.242, 0.297)	0.684 (0.646, 0.721)
$\omega_1 = \omega_2 = \log(2.0)$, $\delta = 5.0$, $\nu = -0.2$	ρ_a	0.000 (0.000, 0.000)	0.738 (0.703, 0.773)
$\omega_1 = \omega_2 = \log(2.0)$, $\delta = 5.0$, $\nu = -0.2$	ρ_d	0.305 (0.276, 0.333)	0.687 (0.650, 0.724)
$\omega_1 = \omega_2 = \log(2.0)$, $\delta = 5.0$, $\nu = 0.0$	ρ_a	0.000 (0.000, 0.000)	0.768 (0.734, 0.803)
$\omega_1 = \omega_2 = \log(2.0)$, $\delta = 5.0$, $\nu = 0.0$	ρ_d	0.285 (0.257, 0.313)	0.686 (0.648, 0.724)
$\omega_1 = \omega_2 = \log(2.0)$, $\delta = 25$, $\nu = -0.2$	ρ_a	0.000 (0.000, 0.000)	0.722 (0.686, 0.758)
$\omega_1 = \omega_2 = \log(2.0)$, $\delta = 25$, $\nu = -0.2$	ρ_d	0.264 (0.236, 0.291)	0.664 (0.627, 0.702)
$\omega_1 = \omega_2 = \log(2.0)$, $\delta = 25$, $\nu = 0.0$	ρ_a	0.000 (0.000, 0.000)	0.760 (0.726, 0.794)
$\omega_1 = \omega_2 = \log(2.0)$, $\delta = 25$, $\nu = 0.0$	ρ_d	0.274 (0.246, 0.301)	0.711 (0.675, 0.748)
**Additional scenarios:**			
$i = 3 \times 4$	ρ_a	0.503 (0.472, 0.534)	0.952 (0.938, 0.966)
$i = 3 \times 4$	ρ_d	0.731 (0.704, 0.759)	0.864 (0.841, 0.886)
$i = 3 \times 4, N = 100$	ρ_a	0.174 (0.151, 0.198)	0.956 (0.943, 0.970)
$i = 3 \times 4, N = 100$	ρ_d	0.725 (0.698, 0.752)	0.853 (0.830, 0.877)
$\sigma^2_\alpha = 1, \sigma^2_\phi = 27.5$	ρ_a	0.519 (0.489, 0.549)	0.954 (0.941, 0.967)

Table B21: Coverage probability of ICCs for the general time on treatment effect parametrisation, with 95% confidence intervals based on Monte Carlo errors. LMM denotes the linear mixed model, while JM denotes the joint model. *(continued)*

Scenario	Parameter	LMM	JM
$\sigma_\alpha^2 = 1, \sigma_\phi^2 = 27.5$	ρ_d	0.867 (0.847, 0.888)	0.943 (0.928, 0.957)
$\sigma_\alpha^2 = 4, \sigma_\phi^2 = 110$	ρ_a	0.000 (0.000, 0.000)	0.947 (0.932, 0.961)
$\sigma_\alpha^2 = 4, \sigma_\phi^2 = 110$	ρ_d	0.744 (0.718, 0.771)	0.925 (0.908, 0.942)

Table B22: Bias of variance components for the general time on treatment effect parametrisation, with 95% confidence intervals based on Monte Carlo errors. LMM denotes the linear mixed model, while JM denotes the joint model. Statistically significant biases are highlighted in bold.

Scenario	Parameter	LMM	JM
**Main scenarios:**			
$\omega_1 = \omega_2 = \log(0.5), \delta = 0.0, \nu = -0.2$	σ_α^2	**-1.659 (-1.680, -1.639)**	**-0.822 (-0.864, -0.780)**
$\omega_1 = \omega_2 = \log(0.5), \delta = 0.0, \nu = -0.2$	σ_ϕ^2	**-32.372 (-32.488, -32.257)**	**-5.155 (-5.444, -4.865)**
$\omega_1 = \omega_2 = \log(0.5), \delta = 0.0, \nu = -0.2$	σ_ϵ^2	**0.128 (0.066, 0.191)**	**-0.415 (-0.489, -0.340)**
$\omega_1 = \omega_2 = \log(0.5), \delta = 0.0, \nu = 0.0$	σ_α^2	**-1.668 (-1.688, -1.649)**	**-0.809 (-0.851, -0.767)**
$\omega_1 = \omega_2 = \log(0.5), \delta = 0.0, \nu = 0.0$	σ_ϕ^2	**-32.549 (-32.657, -32.441)**	**-5.371 (-5.640, -5.102)**
$\omega_1 = \omega_2 = \log(0.5), \delta = 0.0, \nu = 0.0$	σ_ϵ^2	**0.165 (0.103, 0.228)**	**-0.396 (-0.470, -0.323)**
$\omega_1 = \omega_2 = \log(0.5), \delta = 5.0, \nu = -0.2$	σ_α^2	**-1.661 (-1.681, -1.642)**	**-0.790 (-0.833, -0.746)**
$\omega_1 = \omega_2 = \log(0.5), \delta = 5.0, \nu = -0.2$	σ_ϕ^2	**-32.403 (-32.512, -32.293)**	**-5.209 (-5.478, -4.940)**
$\omega_1 = \omega_2 = \log(0.5), \delta = 5.0, \nu = -0.2$	σ_ϵ^2	**0.170 (0.107, 0.232)**	**-0.380 (-0.452, -0.309)**
$\omega_1 = \omega_2 = \log(0.5), \delta = 5.0, \nu = 0.0$	σ_α^2	**-1.675 (-1.695, -1.655)**	**-0.827 (-0.870, -0.783)**
$\omega_1 = \omega_2 = \log(0.5), \delta = 5.0, \nu = 0.0$	σ_ϕ^2	**-32.565 (-32.672, -32.458)**	**-5.420 (-5.688, -5.152)**
$\omega_1 = \omega_2 = \log(0.5), \delta = 5.0, \nu = 0.0$	σ_ϵ^2	**0.108 (0.046, 0.169)**	**-0.464 (-0.537, -0.391)**
$\omega_1 = \omega_2 = \log(0.5), \delta = 25, \nu = -0.2$	σ_α^2	**-1.657 (-1.677, -1.638)**	**-0.814 (-0.856, -0.773)**
$\omega_1 = \omega_2 = \log(0.5), \delta = 25, \nu = -0.2$	σ_ϕ^2	**-32.366 (-32.480, -32.253)**	**-5.087 (-5.366, -4.807)**
$\omega_1 = \omega_2 = \log(0.5), \delta = 25, \nu = -0.2$	σ_ϵ^2	**0.138 (0.076, 0.201)**	**-0.394 (-0.467, -0.320)**
$\omega_1 = \omega_2 = \log(0.5), \delta = 25, \nu = 0.0$	σ_α^2	**-1.654 (-1.674, -1.633)**	**-0.827 (-0.870, -0.783)**
$\omega_1 = \omega_2 = \log(0.5), \delta = 25, \nu = 0.0$	σ_ϕ^2	**-32.518 (-32.630, -32.407)**	**-5.209 (-5.490, -4.928)**
$\omega_1 = \omega_2 = \log(0.5), \delta = 25, \nu = 0.0$	σ_ϵ^2	**0.159 (0.096, 0.221)**	**-0.425 (-0.500, -0.350)**
$\omega_1 = \omega_2 = \log(0.9), \delta = 0.0, \nu = -0.2$	σ_α^2	**-0.702 (-0.746, -0.657)**	**-0.081 (-0.139, -0.023)**
$\omega_1 = \omega_2 = \log(0.9), \delta = 0.0, \nu = -0.2$	σ_ϕ^2	**-10.041 (-10.186, -9.895)**	0.016 (-0.192, 0.225)
$\omega_1 = \omega_2 = \log(0.9), \delta = 0.0, \nu = -0.2$	σ_ϵ^2	**0.238 (0.174, 0.301)**	**-0.110 (-0.176, -0.045)**
$\omega_1 = \omega_2 = \log(0.9), \delta = 0.0, \nu = 0.0$	σ_α^2	**-0.704 (-0.747, -0.660)**	**-0.086 (-0.143, -0.030)**
$\omega_1 = \omega_2 = \log(0.9), \delta = 0.0, \nu = 0.0$	σ_ϕ^2	**-10.178 (-10.330, -10.025)**	0.066 (-0.147, 0.279)
$\omega_1 = \omega_2 = \log(0.9), \delta = 0.0, \nu = 0.0$	σ_ϵ^2	**0.319 (0.254, 0.384)**	**-0.073 (-0.139, -0.007)**
$\omega_1 = \omega_2 = \log(0.9), \delta = 5.0, \nu = -0.2$	σ_α^2	**-0.668 (-0.713, -0.623)**	-0.024 (-0.083, 0.035)
$\omega_1 = \omega_2 = \log(0.9), \delta = 5.0, \nu = -0.2$	σ_ϕ^2	**-10.006 (-10.161, -9.850)**	0.197 (-0.033, 0.426)

Table B22: Bias of variance components for the general time on treatment effect parametrisation, with 95% confidence intervals based on Monte Carlo errors. LMM denotes the linear mixed model, while JM denotes the joint model. Statistically significant biases are highlighted in bold. *(continued)*

Scenario	Parameter	LMM	JM
$\omega_1 = \omega_2 = \log(0.9)$, $\delta = 5.0$, $\nu = -0.2$	σ^2_ϵ	**0.205 (0.141, 0.269)**	**-0.137 (-0.204, -0.070)**
$\omega_1 = \omega_2 = \log(0.9)$, $\delta = 5.0$, $\nu = 0.0$	σ^2_α	**-0.673 (-0.718, -0.628)**	-0.057 (-0.115, 0.001)
$\omega_1 = \omega_2 = \log(0.9)$, $\delta = 5.0$, $\nu = 0.0$	σ^2_ϕ	**-10.149 (-10.301, -9.997)**	0.037 (-0.175, 0.249)
$\omega_1 = \omega_2 = \log(0.9)$, $\delta = 5.0$, $\nu = 0.0$	σ^2_ϵ	**0.263 (0.200, 0.326)**	**-0.126 (-0.190, -0.062)**
$\omega_1 = \omega_2 = \log(0.9)$, $\delta = 25$, $\nu = -0.2$	σ^2_α	**-0.687 (-0.732, -0.641)**	-0.035 (-0.096, 0.025)
$\omega_1 = \omega_2 = \log(0.9)$, $\delta = 25$, $\nu = -0.2$	σ^2_ϕ	**-10.086 (-10.241, -9.931)**	0.052 (-0.170, 0.275)
$\omega_1 = \omega_2 = \log(0.9)$, $\delta = 25$, $\nu = -0.2$	σ^2_ϵ	**0.271 (0.209, 0.334)**	**-0.080 (-0.145, -0.015)**
$\omega_1 = \omega_2 = \log(0.9)$, $\delta = 25$, $\nu = 0.0$	σ^2_α	**-0.725 (-0.767, -0.683)**	**-0.123 (-0.179, -0.067)**
$\omega_1 = \omega_2 = \log(0.9)$, $\delta = 25$, $\nu = 0.0$	σ^2_ϕ	**-10.055 (-10.209, -9.902)**	**0.250 (0.037, 0.464)**
$\omega_1 = \omega_2 = \log(0.9)$, $\delta = 25$, $\nu = 0.0$	σ^2_ϵ	**0.219 (0.158, 0.280)**	**-0.182 (-0.243, -0.121)**
$\omega_1 = \omega_2 = \log(1.0)$, $\delta = 0.0$, $\nu = -0.2$	σ^2_α	**-0.076 (-0.134, -0.018)**	-0.034 (-0.092, 0.024)
$\omega_1 = \omega_2 = \log(1.0)$, $\delta = 0.0$, $\nu = -0.2$	σ^2_ϕ	-0.087 (-0.253, 0.080)	-0.046 (-0.216, 0.125)
$\omega_1 = \omega_2 = \log(1.0)$, $\delta = 0.0$, $\nu = -0.2$	σ^2_ϵ	-0.044 (-0.102, 0.015)	-0.047 (-0.106, 0.013)
$\omega_1 = \omega_2 = \log(1.0)$, $\delta = 0.0$, $\nu = 0.0$	σ^2_α	**-0.117 (-0.173, -0.062)**	**-0.090 (-0.145, -0.036)**
$\omega_1 = \omega_2 = \log(1.0)$, $\delta = 0.0$, $\nu = 0.0$	σ^2_ϕ	-0.113 (-0.289, 0.064)	-0.090 (-0.269, 0.089)
$\omega_1 = \omega_2 = \log(1.0)$, $\delta = 0.0$, $\nu = 0.0$	σ^2_ϵ	**-0.097 (-0.163, -0.032)**	**-0.093 (-0.159, -0.027)**
$\omega_1 = \omega_2 = \log(1.0)$, $\delta = 5.0$, $\nu = -0.2$	σ^2_α	**-0.158 (-0.214, -0.103)**	**-0.138 (-0.194, -0.083)**
$\omega_1 = \omega_2 = \log(1.0)$, $\delta = 5.0$, $\nu = -0.2$	σ^2_ϕ	-0.027 (-0.198, 0.145)	-0.007 (-0.180, 0.166)
$\omega_1 = \omega_2 = \log(1.0)$, $\delta = 5.0$, $\nu = -0.2$	σ^2_ϵ	**-0.112 (-0.174, -0.050)**	**-0.108 (-0.171, -0.045)**
$\omega_1 = \omega_2 = \log(1.0)$, $\delta = 5.0$, $\nu = 0.0$	σ^2_α	**-0.123 (-0.177, -0.068)**	**-0.089 (-0.143, -0.035)**
$\omega_1 = \omega_2 = \log(1.0)$, $\delta = 5.0$, $\nu = 0.0$	σ^2_ϕ	-0.107 (-0.285, 0.072)	-0.102 (-0.282, 0.077)
$\omega_1 = \omega_2 = \log(1.0)$, $\delta = 5.0$, $\nu = 0.0$	σ^2_ϵ	**-0.068 (-0.130, -0.007)**	**-0.070 (-0.131, -0.008)**
$\omega_1 = \omega_2 = \log(1.0)$, $\delta = 25$, $\nu = -0.2$	σ^2_α	**-0.117 (-0.173, -0.061)**	**-0.092 (-0.149, -0.036)**
$\omega_1 = \omega_2 = \log(1.0)$, $\delta = 25$, $\nu = -0.2$	σ^2_ϕ	-0.035 (-0.208, 0.138)	-0.012 (-0.187, 0.163)
$\omega_1 = \omega_2 = \log(1.0)$, $\delta = 25$, $\nu = -0.2$	σ^2_ϵ	**-0.099 (-0.161, -0.038)**	**-0.094 (-0.156, -0.032)**
$\omega_1 = \omega_2 = \log(1.0)$, $\delta = 25$, $\nu = 0.0$	σ^2_α	**-0.120 (-0.176, -0.063)**	**-0.084 (-0.141, -0.028)**
$\omega_1 = \omega_2 = \log(1.0)$, $\delta = 25$, $\nu = 0.0$	σ^2_ϕ	0.006 (-0.166, 0.179)	0.005 (-0.169, 0.180)
$\omega_1 = \omega_2 = \log(1.0)$, $\delta = 25$, $\nu = 0.0$	σ^2_ϵ	**-0.153 (-0.215, -0.091)**	**-0.150 (-0.212, -0.087)**
$\omega_1 = \omega_2 = \log(2.0)$, $\delta = 0.0$, $\nu = -0.2$	σ^2_α	**-1.669 (-1.689, -1.649)**	**-0.806 (-0.849, -0.762)**
$\omega_1 = \omega_2 = \log(2.0)$, $\delta = 0.0$, $\nu = -0.2$	σ^2_ϕ	**-32.458 (-32.571, -32.345)**	**-5.143 (-5.437, -4.848)**
$\omega_1 = \omega_2 = \log(2.0)$, $\delta = 0.0$, $\nu = -0.2$	σ^2_ϵ	**0.106 (0.043, 0.168)**	**-0.435 (-0.511, -0.359)**
$\omega_1 = \omega_2 = \log(2.0)$, $\delta = 0.0$, $\nu = 0.0$	σ^2_α	**-1.670 (-1.690, -1.650)**	**-0.808 (-0.853, -0.763)**
$\omega_1 = \omega_2 = \log(2.0)$, $\delta = 0.0$, $\nu = 0.0$	σ^2_ϕ	**-32.529 (-32.642, -32.416)**	**-5.472 (-5.782, -5.162)**
$\omega_1 = \omega_2 = \log(2.0)$, $\delta = 0.0$, $\nu = 0.0$	σ^2_ϵ	**0.101 (0.038, 0.164)**	**-0.513 (-0.592, -0.433)**
$\omega_1 = \omega_2 = \log(2.0)$, $\delta = 5.0$, $\nu = -0.2$	σ^2_α	**-1.650 (-1.670, -1.630)**	**-0.791 (-0.837, -0.745)**
$\omega_1 = \omega_2 = \log(2.0)$, $\delta = 5.0$, $\nu = -0.2$	σ^2_ϕ	**-32.462 (-32.570, -32.354)**	**-5.352 (-5.647, -5.058)**
$\omega_1 = \omega_2 = \log(2.0)$, $\delta = 5.0$, $\nu = -0.2$	σ^2_ϵ	**0.125 (0.062, 0.188)**	**-0.396 (-0.476, -0.316)**

Table B22: Bias of variance components for the general time on treatment effect parametrisation, with 95% confidence intervals based on Monte Carlo errors. LMM denotes the linear mixed model, while JM denotes the joint model. Statistically significant biases are highlighted in bold. *(continued)*

Scenario	Parameter	LMM	JM
$\omega_1 = \omega_2 = \log(2.0)$, $\delta = 5.0$, $\nu = 0.0$	σ^2_α	**-1.663 (-1.683, -1.643)**	**-0.802 (-0.847, -0.757)**
$\omega_1 = \omega_2 = \log(2.0)$, $\delta = 5.0$, $\nu = 0.0$	σ^2_ϕ	**-32.498 (-32.611, -32.385)**	**-5.399 (-5.693, -5.105)**
$\omega_1 = \omega_2 = \log(2.0)$, $\delta = 5.0$, $\nu = 0.0$	σ^2_ϵ	**0.172 (0.110, 0.234)**	**-0.346 (-0.425, -0.268)**
$\omega_1 = \omega_2 = \log(2.0)$, $\delta = 25$, $\nu = -0.2$	σ^2_α	**-1.661 (-1.681, -1.640)**	**-0.837 (-0.883, -0.791)**
$\omega_1 = \omega_2 = \log(2.0)$, $\delta = 25$, $\nu = -0.2$	σ^2_ϕ	**-32.493 (-32.604, -32.381)**	**-5.362 (-5.648, -5.077)**
$\omega_1 = \omega_2 = \log(2.0)$, $\delta = 25$, $\nu = -0.2$	σ^2_ϵ	**0.181 (0.118, 0.244)**	**-0.352 (-0.430, -0.274)**
$\omega_1 = \omega_2 = \log(2.0)$, $\delta = 25$, $\nu = 0.0$	σ^2_α	**-1.665 (-1.684, -1.645)**	**-0.778 (-0.822, -0.733)**
$\omega_1 = \omega_2 = \log(2.0)$, $\delta = 25$, $\nu = 0.0$	σ^2_ϕ	**-32.540 (-32.651, -32.430)**	**-5.400 (-5.689, -5.112)**
$\omega_1 = \omega_2 = \log(2.0)$, $\delta = 25$, $\nu = 0.0$	σ^2_ϵ	**0.118 (0.056, 0.180)**	**-0.400 (-0.477, -0.324)**
**Additional scenarios:**			
$i = 3 \times 4$	σ^2_α	**-0.840 (-0.906, -0.774)**	**-0.140 (-0.230, -0.049)**
$i = 3 \times 4$	σ^2_ϕ	**-9.788 (-10.041, -9.535)**	**0.618 (0.247, 0.989)**
$i = 3 \times 4$	σ^2_ϵ	**0.124 (0.020, 0.228)**	**-0.208 (-0.317, -0.098)**
$i = 3 \times 4, N = 100$	σ^2_α	**-0.787 (-0.838, -0.736)**	**-0.185 (-0.255, -0.114)**
$i = 3 \times 4, N = 100$	σ^2_ϕ	**-10.019 (-10.189, -9.849)**	0.012 (-0.233, 0.258)
$i = 3 \times 4, N = 100$	σ^2_ϵ	**0.277 (0.205, 0.349)**	-0.059 (-0.135, 0.017)
$\sigma^2_\alpha = 1, \sigma^2_\phi = 27.5$	σ^2_α	**-0.206 (-0.233, -0.179)**	0.009 (-0.022, 0.040)
$\sigma^2_\alpha = 1, \sigma^2_\phi = 27.5$	σ^2_ϕ	**-3.375 (-3.467, -3.283)**	0.002 (-0.112, 0.116)
$\sigma^2_\alpha = 1, \sigma^2_\phi = 27.5$	σ^2_ϵ	**0.171 (0.109, 0.233)**	**-0.097 (-0.159, -0.035)**
$\sigma^2_\alpha = 4, \sigma^2_\phi = 110$	σ^2_α	**-1.854 (-1.928, -1.780)**	**-0.126 (-0.239, -0.013)**
$\sigma^2_\alpha = 4, \sigma^2_\phi = 110$	σ^2_ϕ	**-28.489 (-28.753, -28.226)**	0.435 (-0.012, 0.882)
$\sigma^2_\alpha = 4, \sigma^2_\phi = 110$	σ^2_ϵ	**0.197 (0.136, 0.258)**	**-0.120 (-0.184, -0.055)**

Table B23: Relative bias of variance components for the general time on treatment effect parametrisation, with 95% confidence intervals based on Monte Carlo errors. LMM denotes the linear mixed model, while JM denotes the joint model. Statistically significant biases are highlighted in bold.

Scenario	Parameter	LMM	JM
**Main scenarios:**			
$\omega_1 = \omega_2 = \log(0.5)$, $\delta = 0.0$, $\nu = -0.2$	σ^2_α	**-0.830 (-0.840, -0.819)**	**-0.411 (-0.432, -0.390)**
$\omega_1 = \omega_2 = \log(0.5)$, $\delta = 0.0$, $\nu = -0.2$	σ^2_ϕ	**-0.589 (-0.591, -0.586)**	**-0.094 (-0.099, -0.088)**
$\omega_1 = \omega_2 = \log(0.5)$, $\delta = 0.0$, $\nu = -0.2$	σ^2_ϵ	**0.003 (0.002, 0.005)**	**-0.010 (-0.012, -0.008)**
$\omega_1 = \omega_2 = \log(0.5)$, $\delta = 0.0$, $\nu = 0.0$	σ^2_α	**-0.834 (-0.844, -0.824)**	**-0.405 (-0.426, -0.384)**
$\omega_1 = \omega_2 = \log(0.5)$, $\delta = 0.0$, $\nu = 0.0$	σ^2_ϕ	**-0.592 (-0.594, -0.590)**	**-0.098 (-0.103, -0.093)**

Table B23: Relative bias of variance components for the general time on treatment effect parametrisation, with 95% confidence intervals based on Monte Carlo errors. LMM denotes the linear mixed model, while JM denotes the joint model. Statistically significant biases are highlighted in bold. *(continued)*

Scenario	Parameter	LMM	JM
$\omega_1 = \omega_2 = \log(0.5)$, $\delta = 0.0$, $\nu = 0.0$	σ_ϵ^2	**0.004 (0.003, 0.006)**	**-0.010 (-0.012, -0.008)**
$\omega_1 = \omega_2 = \log(0.5)$, $\delta = 5.0$, $\nu = -0.2$	σ_α^2	**-0.831 (-0.841, -0.821)**	**-0.395 (-0.417, -0.373)**
$\omega_1 = \omega_2 = \log(0.5)$, $\delta = 5.0$, $\nu = -0.2$	σ_ϕ^2	**-0.589 (-0.591, -0.587)**	**-0.095 (-0.100, -0.090)**
$\omega_1 = \omega_2 = \log(0.5)$, $\delta = 5.0$, $\nu = -0.2$	σ_ϵ^2	**0.004 (0.003, 0.006)**	**-0.010 (-0.011, -0.008)**
$\omega_1 = \omega_2 = \log(0.5)$, $\delta = 5.0$, $\nu = 0.0$	σ_α^2	**-0.838 (-0.848, -0.828)**	**-0.413 (-0.435, -0.392)**
$\omega_1 = \omega_2 = \log(0.5)$, $\delta = 5.0$, $\nu = 0.0$	σ_ϕ^2	**-0.592 (-0.594, -0.590)**	**-0.099 (-0.103, -0.094)**
$\omega_1 = \omega_2 = \log(0.5)$, $\delta = 5.0$, $\nu = 0.0$	σ_ϵ^2	**0.003 (0.001, 0.004)**	**-0.012 (-0.013, -0.010)**
$\omega_1 = \omega_2 = \log(0.5)$, $\delta = 25$, $\nu = -0.2$	σ_α^2	**-0.829 (-0.838, -0.819)**	**-0.407 (-0.428, -0.386)**
$\omega_1 = \omega_2 = \log(0.5)$, $\delta = 25$, $\nu = -0.2$	σ_ϕ^2	**-0.588 (-0.591, -0.586)**	**-0.092 (-0.098, -0.087)**
$\omega_1 = \omega_2 = \log(0.5)$, $\delta = 25$, $\nu = -0.2$	σ_ϵ^2	**0.003 (0.002, 0.005)**	**-0.010 (-0.012, -0.008)**
$\omega_1 = \omega_2 = \log(0.5)$, $\delta = 25$, $\nu = 0.0$	σ_α^2	**-0.827 (-0.837, -0.817)**	**-0.413 (-0.435, -0.391)**
$\omega_1 = \omega_2 = \log(0.5)$, $\delta = 25$, $\nu = 0.0$	σ_ϕ^2	**-0.591 (-0.593, -0.589)**	**-0.095 (-0.100, -0.090)**
$\omega_1 = \omega_2 = \log(0.5)$, $\delta = 25$, $\nu = 0.0$	σ_ϵ^2	**0.004 (0.002, 0.006)**	**-0.011 (-0.012, -0.009)**
$\omega_1 = \omega_2 = \log(0.9)$, $\delta = 0.0$, $\nu = -0.2$	σ_α^2	**-0.351 (-0.373, -0.328)**	**-0.040 (-0.069, -0.011)**
$\omega_1 = \omega_2 = \log(0.9)$, $\delta = 0.0$, $\nu = -0.2$	σ_ϕ^2	**-0.183 (-0.185, -0.180)**	0.000 (-0.003, 0.004)
$\omega_1 = \omega_2 = \log(0.9)$, $\delta = 0.0$, $\nu = -0.2$	σ_ϵ^2	**0.006 (0.004, 0.008)**	**-0.003 (-0.004, -0.001)**
$\omega_1 = \omega_2 = \log(0.9)$, $\delta = 0.0$, $\nu = 0.0$	σ_α^2	**-0.352 (-0.373, -0.330)**	**-0.043 (-0.071, -0.015)**
$\omega_1 = \omega_2 = \log(0.9)$, $\delta = 0.0$, $\nu = 0.0$	σ_ϕ^2	**-0.185 (-0.188, -0.182)**	0.001 (-0.003, 0.005)
$\omega_1 = \omega_2 = \log(0.9)$, $\delta = 0.0$, $\nu = 0.0$	σ_ϵ^2	**0.008 (0.006, 0.010)**	**-0.002 (-0.003, -0.000)**
$\omega_1 = \omega_2 = \log(0.9)$, $\delta = 5.0$, $\nu = -0.2$	σ_α^2	**-0.334 (-0.357, -0.311)**	-0.012 (-0.042, 0.017)
$\omega_1 = \omega_2 = \log(0.9)$, $\delta = 5.0$, $\nu = -0.2$	σ_ϕ^2	**-0.182 (-0.185, -0.179)**	0.004 (-0.001, 0.008)
$\omega_1 = \omega_2 = \log(0.9)$, $\delta = 5.0$, $\nu = -0.2$	σ_ϵ^2	**0.005 (0.004, 0.007)**	**-0.003 (-0.005, -0.002)**
$\omega_1 = \omega_2 = \log(0.9)$, $\delta = 5.0$, $\nu = 0.0$	σ_α^2	**-0.336 (-0.359, -0.314)**	-0.028 (-0.057, 0.000)
$\omega_1 = \omega_2 = \log(0.9)$, $\delta = 5.0$, $\nu = 0.0$	σ_ϕ^2	**-0.185 (-0.187, -0.182)**	0.001 (-0.003, 0.005)
$\omega_1 = \omega_2 = \log(0.9)$, $\delta = 5.0$, $\nu = 0.0$	σ_ϵ^2	**0.007 (0.005, 0.008)**	**-0.003 (-0.005, -0.002)**
$\omega_1 = \omega_2 = \log(0.9)$, $\delta = 25$, $\nu = -0.2$	σ_α^2	**-0.343 (-0.366, -0.321)**	-0.018 (-0.048, 0.012)
$\omega_1 = \omega_2 = \log(0.9)$, $\delta = 25$, $\nu = -0.2$	σ_ϕ^2	**-0.183 (-0.186, -0.181)**	0.001 (-0.003, 0.005)
$\omega_1 = \omega_2 = \log(0.9)$, $\delta = 25$, $\nu = -0.2$	σ_ϵ^2	**0.007 (0.005, 0.008)**	**-0.002 (-0.004, -0.000)**
$\omega_1 = \omega_2 = \log(0.9)$, $\delta = 25$, $\nu = 0.0$	σ_α^2	**-0.363 (-0.384, -0.341)**	**-0.061 (-0.090, -0.033)**
$\omega_1 = \omega_2 = \log(0.9)$, $\delta = 25$, $\nu = 0.0$	σ_ϕ^2	**-0.183 (-0.186, -0.180)**	**0.005 (0.001, 0.008)**
$\omega_1 = \omega_2 = \log(0.9)$, $\delta = 25$, $\nu = 0.0$	σ_ϵ^2	**0.005 (0.004, 0.007)**	**-0.005 (-0.006, -0.003)**
$\omega_1 = \omega_2 = \log(1.0)$, $\delta = 0.0$, $\nu = -0.2$	σ_α^2	**-0.038 (-0.067, -0.009)**	-0.017 (-0.046, 0.012)
$\omega_1 = \omega_2 = \log(1.0)$, $\delta = 0.0$, $\nu = -0.2$	σ_ϕ^2	-0.002 (-0.005, 0.001)	-0.001 (-0.004, 0.002)
$\omega_1 = \omega_2 = \log(1.0)$, $\delta = 0.0$, $\nu = -0.2$	σ_ϵ^2	-0.001 (-0.003, 0.000)	-0.001 (-0.003, 0.000)
$\omega_1 = \omega_2 = \log(1.0)$, $\delta = 0.0$, $\nu = 0.0$	σ_α^2	**-0.059 (-0.086, -0.031)**	**-0.045 (-0.073, -0.018)**
$\omega_1 = \omega_2 = \log(1.0)$, $\delta = 0.0$, $\nu = 0.0$	σ_ϕ^2	-0.002 (-0.005, 0.001)	-0.002 (-0.005, 0.002)
$\omega_1 = \omega_2 = \log(1.0)$, $\delta = 0.0$, $\nu = 0.0$	σ_ϵ^2	**-0.002 (-0.004, -0.001)**	**-0.002 (-0.004, -0.001)**

Table B23: Relative bias of variance components for the general time on treatment effect parametrisation, with 95% confidence intervals based on Monte Carlo errors. LMM denotes the linear mixed model, while JM denotes the joint model. Statistically significant biases are highlighted in bold. *(continued)*

Scenario	Parameter	LMM	JM
$\omega_1 = \omega_2 = \log(1.0), \delta = 5.0, \nu = -0.2$	σ^2_α	**-0.079 (-0.107, -0.051)**	**-0.069 (-0.097, -0.041)**
$\omega_1 = \omega_2 = \log(1.0), \delta = 5.0, \nu = -0.2$	σ^2_ϕ	-0.000 (-0.004, 0.003)	-0.000 (-0.003, 0.003)
$\omega_1 = \omega_2 = \log(1.0), \delta = 5.0, \nu = -0.2$	σ^2_ϵ	**-0.003 (-0.004, -0.001)**	**-0.003 (-0.004, -0.001)**
$\omega_1 = \omega_2 = \log(1.0), \delta = 5.0, \nu = 0.0$	σ^2_α	**-0.061 (-0.089, -0.034)**	**-0.045 (-0.072, -0.017)**
$\omega_1 = \omega_2 = \log(1.0), \delta = 5.0, \nu = 0.0$	σ^2_ϕ	-0.002 (-0.005, 0.001)	-0.002 (-0.005, 0.001)
$\omega_1 = \omega_2 = \log(1.0), \delta = 5.0, \nu = 0.0$	σ^2_ϵ	**-0.002 (-0.003, -0.000)**	**-0.002 (-0.003, -0.000)**
$\omega_1 = \omega_2 = \log(1.0), \delta = 25, \nu = -0.2$	σ^2_α	**-0.058 (-0.087, -0.030)**	**-0.046 (-0.074, -0.018)**
$\omega_1 = \omega_2 = \log(1.0), \delta = 25, \nu = -0.2$	σ^2_ϕ	-0.001 (-0.004, 0.003)	-0.000 (-0.003, 0.003)
$\omega_1 = \omega_2 = \log(1.0), \delta = 25, \nu = -0.2$	σ^2_ϵ	**-0.002 (-0.004, -0.001)**	**-0.002 (-0.004, -0.001)**
$\omega_1 = \omega_2 = \log(1.0), \delta = 25, \nu = 0.0$	σ^2_α	**-0.060 (-0.088, -0.032)**	**-0.042 (-0.070, -0.014)**
$\omega_1 = \omega_2 = \log(1.0), \delta = 25, \nu = 0.0$	σ^2_ϕ	0.000 (-0.003, 0.003)	0.000 (-0.003, 0.003)
$\omega_1 = \omega_2 = \log(1.0), \delta = 25, \nu = 0.0$	σ^2_ϵ	**-0.004 (-0.005, -0.002)**	**-0.004 (-0.005, -0.002)**
$\omega_1 = \omega_2 = \log(2.0), \delta = 0.0, \nu = -0.2$	σ^2_α	**-0.835 (-0.844, -0.825)**	**-0.403 (-0.424, -0.381)**
$\omega_1 = \omega_2 = \log(2.0), \delta = 0.0, \nu = -0.2$	σ^2_ϕ	**-0.590 (-0.592, -0.588)**	**-0.094 (-0.099, -0.088)**
$\omega_1 = \omega_2 = \log(2.0), \delta = 0.0, \nu = -0.2$	σ^2_ϵ	**0.003 (0.001, 0.004)**	**-0.011 (-0.013, -0.009)**
$\omega_1 = \omega_2 = \log(2.0), \delta = 0.0, \nu = 0.0$	σ^2_α	**-0.835 (-0.845, -0.825)**	**-0.404 (-0.427, -0.381)**
$\omega_1 = \omega_2 = \log(2.0), \delta = 0.0, \nu = 0.0$	σ^2_ϕ	**-0.591 (-0.593, -0.589)**	**-0.099 (-0.105, -0.094)**
$\omega_1 = \omega_2 = \log(2.0), \delta = 0.0, \nu = 0.0$	σ^2_ϵ	**0.003 (0.001, 0.004)**	**-0.013 (-0.015, -0.011)**
$\omega_1 = \omega_2 = \log(2.0), \delta = 5.0, \nu = -0.2$	σ^2_α	**-0.825 (-0.835, -0.815)**	**-0.396 (-0.419, -0.373)**
$\omega_1 = \omega_2 = \log(2.0), \delta = 5.0, \nu = -0.2$	σ^2_ϕ	**-0.590 (-0.592, -0.588)**	**-0.097 (-0.103, -0.092)**
$\omega_1 = \omega_2 = \log(2.0), \delta = 5.0, \nu = -0.2$	σ^2_ϵ	**0.003 (0.002, 0.005)**	**-0.010 (-0.012, -0.008)**
$\omega_1 = \omega_2 = \log(2.0), \delta = 5.0, \nu = 0.0$	σ^2_α	**-0.831 (-0.841, -0.822)**	**-0.401 (-0.423, -0.379)**
$\omega_1 = \omega_2 = \log(2.0), \delta = 5.0, \nu = 0.0$	σ^2_ϕ	**-0.591 (-0.593, -0.589)**	**-0.098 (-0.104, -0.093)**
$\omega_1 = \omega_2 = \log(2.0), \delta = 5.0, \nu = 0.0$	σ^2_ϵ	**0.004 (0.003, 0.006)**	**-0.009 (-0.011, -0.007)**
$\omega_1 = \omega_2 = \log(2.0), \delta = 25, \nu = -0.2$	σ^2_α	**-0.830 (-0.841, -0.820)**	**-0.418 (-0.441, -0.396)**
$\omega_1 = \omega_2 = \log(2.0), \delta = 25, \nu = -0.2$	σ^2_ϕ	**-0.591 (-0.593, -0.589)**	**-0.097 (-0.103, -0.092)**
$\omega_1 = \omega_2 = \log(2.0), \delta = 25, \nu = -0.2$	σ^2_ϵ	**0.005 (0.003, 0.006)**	**-0.009 (-0.011, -0.007)**
$\omega_1 = \omega_2 = \log(2.0), \delta = 25, \nu = 0.0$	σ^2_α	**-0.832 (-0.842, -0.822)**	**-0.389 (-0.411, -0.367)**
$\omega_1 = \omega_2 = \log(2.0), \delta = 25, \nu = 0.0$	σ^2_ϕ	**-0.592 (-0.594, -0.590)**	**-0.098 (-0.103, -0.093)**
$\omega_1 = \omega_2 = \log(2.0), \delta = 25, \nu = 0.0$	σ^2_ϵ	**0.003 (0.001, 0.005)**	**-0.010 (-0.012, -0.008)**
**Additional scenarios:**			
$i = 3 \times 4$	σ^2_α	**-0.420 (-0.453, -0.387)**	**-0.070 (-0.115, -0.025)**
$i = 3 \times 4$	σ^2_ϕ	**-0.178 (-0.183, -0.173)**	**0.011 (0.004, 0.018)**
$i = 3 \times 4$	σ^2_ϵ	**0.003 (0.001, 0.006)**	**-0.005 (-0.008, -0.002)**
$i = 3 \times 4, N = 100$	σ^2_α	**-0.393 (-0.419, -0.368)**	**-0.092 (-0.128, -0.057)**
$i = 3 \times 4, N = 100$	σ^2_ϕ	**-0.182 (-0.185, -0.179)**	0.000 (-0.004, 0.005)
$i = 3 \times 4, N = 100$	σ^2_ϵ	**0.007 (0.005, 0.009)**	-0.001 (-0.003, 0.000)

Table B23: Relative bias of variance components for the general time on treatment effect parametrisation, with 95% confidence intervals based on Monte Carlo errors. LMM denotes the linear mixed model, while JM denotes the joint model. Statistically significant biases are highlighted in bold. *(continued)*

Scenario	Parameter	LMM	JM
$\sigma_\alpha^2 = 1, \sigma_\phi^2 = 27.5$	σ_α^2	**-0.206 (-0.233, -0.179)**	0.009 (-0.022, 0.040)
$\sigma_\alpha^2 = 1, \sigma_\phi^2 = 27.5$	σ_ϕ^2	**-0.123 (-0.126, -0.119)**	0.000 (-0.004, 0.004)
$\sigma_\alpha^2 = 1, \sigma_\phi^2 = 27.5$	σ_ϵ^2	**0.004 (0.003, 0.006)**	**-0.002 (-0.004, -0.001)**
$\sigma_\alpha^2 = 4, \sigma_\phi^2 = 110$	σ_α^2	**-0.464 (-0.482, -0.445)**	**-0.031 (-0.060, -0.003)**
$\sigma_\alpha^2 = 4, \sigma_\phi^2 = 110$	σ_ϕ^2	**-0.259 (-0.261, -0.257)**	0.004 (-0.000, 0.008)
$\sigma_\alpha^2 = 4, \sigma_\phi^2 = 110$	σ_ϵ^2	**0.005 (0.003, 0.006)**	**-0.003 (-0.005, -0.001)**

Table B24: Coverage probability of variance components for the general time on treatment effect parametrisation, with 95% confidence intervals based on Monte Carlo errors. LMM denotes the linear mixed model, while JM denotes the joint model.

Scenario	Parameter	LMM	JM
**Main scenarios:**			
$\omega_1 = \omega_2 = \log(0.5), \delta = 0.0, \nu = -0.2$	σ_α^2	0.055 (0.041, 0.069)	0.613 (0.576, 0.650)
$\omega_1 = \omega_2 = \log(0.5), \delta = 0.0, \nu = -0.2$	σ_ϕ^2	0.000 (0.000, 0.000)	0.677 (0.642, 0.713)
$\omega_1 = \omega_2 = \log(0.5), \delta = 0.0, \nu = -0.2$	σ_ϵ^2	0.954 (0.941, 0.967)	0.910 (0.888, 0.932)
$\omega_1 = \omega_2 = \log(0.5), \delta = 0.0, \nu = 0.0$	σ_α^2	0.048 (0.034, 0.061)	0.622 (0.586, 0.659)
$\omega_1 = \omega_2 = \log(0.5), \delta = 0.0, \nu = 0.0$	σ_ϕ^2	0.000 (0.000, 0.000)	0.682 (0.647, 0.717)
$\omega_1 = \omega_2 = \log(0.5), \delta = 0.0, \nu = 0.0$	σ_ϵ^2	0.958 (0.946, 0.971)	0.932 (0.913, 0.951)
$\omega_1 = \omega_2 = \log(0.5), \delta = 5.0, \nu = -0.2$	σ_α^2	0.041 (0.029, 0.054)	0.650 (0.614, 0.686)
$\omega_1 = \omega_2 = \log(0.5), \delta = 5.0, \nu = -0.2$	σ_ϕ^2	0.000 (0.000, 0.000)	0.704 (0.669, 0.739)
$\omega_1 = \omega_2 = \log(0.5), \delta = 5.0, \nu = -0.2$	σ_ϵ^2	0.946 (0.932, 0.960)	0.946 (0.929, 0.963)
$\omega_1 = \omega_2 = \log(0.5), \delta = 5.0, \nu = 0.0$	σ_α^2	0.046 (0.033, 0.059)	0.582 (0.544, 0.620)
$\omega_1 = \omega_2 = \log(0.5), \delta = 5.0, \nu = 0.0$	σ_ϕ^2	0.000 (0.000, 0.000)	0.655 (0.618, 0.691)
$\omega_1 = \omega_2 = \log(0.5), \delta = 5.0, \nu = 0.0$	σ_ϵ^2	0.958 (0.945, 0.970)	0.938 (0.920, 0.957)
$\omega_1 = \omega_2 = \log(0.5), \delta = 25, \nu = -0.2$	σ_α^2	0.055 (0.041, 0.069)	0.613 (0.576, 0.650)
$\omega_1 = \omega_2 = \log(0.5), \delta = 25, \nu = -0.2$	σ_ϕ^2	0.000 (0.000, 0.000)	0.698 (0.663, 0.733)
$\omega_1 = \omega_2 = \log(0.5), \delta = 25, \nu = -0.2$	σ_ϵ^2	0.946 (0.932, 0.960)	0.926 (0.906, 0.946)
$\omega_1 = \omega_2 = \log(0.5), \delta = 25, \nu = 0.0$	σ_α^2	0.051 (0.037, 0.064)	0.607 (0.569, 0.644)
$\omega_1 = \omega_2 = \log(0.5), \delta = 25, \nu = 0.0$	σ_ϕ^2	0.000 (0.000, 0.000)	0.704 (0.668, 0.739)
$\omega_1 = \omega_2 = \log(0.5), \delta = 25, \nu = 0.0$	σ_ϵ^2	0.953 (0.940, 0.966)	0.933 (0.913, 0.952)
$\omega_1 = \omega_2 = \log(0.9), \delta = 0.0, \nu = -0.2$	σ_α^2	0.717 (0.690, 0.745)	0.907 (0.888, 0.925)
$\omega_1 = \omega_2 = \log(0.9), \delta = 0.0, \nu = -0.2$	σ_ϕ^2	0.026 (0.016, 0.035)	0.961 (0.949, 0.973)
$\omega_1 = \omega_2 = \log(0.9), \delta = 0.0, \nu = -0.2$	σ_ϵ^2	0.933 (0.918, 0.948)	0.938 (0.923, 0.953)
$\omega_1 = \omega_2 = \log(0.9), \delta = 0.0, \nu = 0.0$	σ_α^2	0.724 (0.697, 0.751)	0.917 (0.899, 0.934)

Table B24: Coverage probability of variance components for the general time on treatment effect parametrisation, with 95% confidence intervals based on Monte Carlo errors. LMM denotes the linear mixed model, while JM denotes the joint model. *(continued)*

Scenario	Parameter	LMM	JM
$\omega_1 = \omega_2 = \log(0.9)$, $\delta = 0.0$, $\nu = 0.0$	σ^2_ϕ	0.025 (0.015, 0.034)	0.952 (0.938, 0.965)
$\omega_1 = \omega_2 = \log(0.9)$, $\delta = 0.0$, $\nu = 0.0$	σ^2_ϵ	0.947 (0.934, 0.961)	0.938 (0.923, 0.953)
$\omega_1 = \omega_2 = \log(0.9)$, $\delta = 5.0$, $\nu = -0.2$	σ^2_α	0.738 (0.711, 0.764)	0.926 (0.910, 0.943)
$\omega_1 = \omega_2 = \log(0.9)$, $\delta = 5.0$, $\nu = -0.2$	σ^2_ϕ	0.033 (0.023, 0.044)	0.939 (0.924, 0.954)
$\omega_1 = \omega_2 = \log(0.9)$, $\delta = 5.0$, $\nu = -0.2$	σ^2_ϵ	0.941 (0.927, 0.955)	0.934 (0.918, 0.949)
$\omega_1 = \omega_2 = \log(0.9)$, $\delta = 5.0$, $\nu = 0.0$	σ^2_α	0.742 (0.715, 0.768)	0.911 (0.894, 0.929)
$\omega_1 = \omega_2 = \log(0.9)$, $\delta = 5.0$, $\nu = 0.0$	σ^2_ϕ	0.027 (0.017, 0.037)	0.954 (0.941, 0.967)
$\omega_1 = \omega_2 = \log(0.9)$, $\delta = 5.0$, $\nu = 0.0$	σ^2_ϵ	0.949 (0.936, 0.963)	0.946 (0.932, 0.960)
$\omega_1 = \omega_2 = \log(0.9)$, $\delta = 25$, $\nu = -0.2$	σ^2_α	0.735 (0.708, 0.762)	0.909 (0.891, 0.927)
$\omega_1 = \omega_2 = \log(0.9)$, $\delta = 25$, $\nu = -0.2$	σ^2_ϕ	0.031 (0.020, 0.041)	0.945 (0.931, 0.960)
$\omega_1 = \omega_2 = \log(0.9)$, $\delta = 25$, $\nu = -0.2$	σ^2_ϵ	0.938 (0.923, 0.952)	0.951 (0.937, 0.964)
$\omega_1 = \omega_2 = \log(0.9)$, $\delta = 25$, $\nu = 0.0$	σ^2_α	0.740 (0.714, 0.767)	0.903 (0.884, 0.921)
$\omega_1 = \omega_2 = \log(0.9)$, $\delta = 25$, $\nu = 0.0$	σ^2_ϕ	0.036 (0.025, 0.048)	0.953 (0.940, 0.966)
$\omega_1 = \omega_2 = \log(0.9)$, $\delta = 25$, $\nu = 0.0$	σ^2_ϵ	0.956 (0.944, 0.969)	0.956 (0.943, 0.969)
$\omega_1 = \omega_2 = \log(1.0)$, $\delta = 0.0$, $\nu = -0.2$	σ^2_α	0.894 (0.875, 0.913)	0.914 (0.897, 0.931)
$\omega_1 = \omega_2 = \log(1.0)$, $\delta = 0.0$, $\nu = -0.2$	σ^2_ϕ	0.958 (0.946, 0.970)	0.957 (0.945, 0.970)
$\omega_1 = \omega_2 = \log(1.0)$, $\delta = 0.0$, $\nu = -0.2$	σ^2_ϵ	0.952 (0.939, 0.965)	0.952 (0.938, 0.965)
$\omega_1 = \omega_2 = \log(1.0)$, $\delta = 0.0$, $\nu = 0.0$	σ^2_α	0.902 (0.884, 0.920)	0.914 (0.897, 0.931)
$\omega_1 = \omega_2 = \log(1.0)$, $\delta = 0.0$, $\nu = 0.0$	σ^2_ϕ	0.937 (0.922, 0.952)	0.936 (0.921, 0.951)
$\omega_1 = \omega_2 = \log(1.0)$, $\delta = 0.0$, $\nu = 0.0$	σ^2_ϵ	0.932 (0.917, 0.948)	0.933 (0.918, 0.948)
$\omega_1 = \omega_2 = \log(1.0)$, $\delta = 5.0$, $\nu = -0.2$	σ^2_α	0.896 (0.878, 0.915)	0.895 (0.876, 0.914)
$\omega_1 = \omega_2 = \log(1.0)$, $\delta = 5.0$, $\nu = -0.2$	σ^2_ϕ	0.950 (0.937, 0.964)	0.950 (0.937, 0.964)
$\omega_1 = \omega_2 = \log(1.0)$, $\delta = 5.0$, $\nu = -0.2$	σ^2_ϵ	0.940 (0.926, 0.954)	0.940 (0.925, 0.954)
$\omega_1 = \omega_2 = \log(1.0)$, $\delta = 5.0$, $\nu = 0.0$	σ^2_α	0.892 (0.873, 0.911)	0.903 (0.885, 0.921)
$\omega_1 = \omega_2 = \log(1.0)$, $\delta = 5.0$, $\nu = 0.0$	σ^2_ϕ	0.942 (0.928, 0.956)	0.940 (0.926, 0.955)
$\omega_1 = \omega_2 = \log(1.0)$, $\delta = 5.0$, $\nu = 0.0$	σ^2_ϵ	0.947 (0.933, 0.960)	0.948 (0.935, 0.962)
$\omega_1 = \omega_2 = \log(1.0)$, $\delta = 25$, $\nu = -0.2$	σ^2_α	0.909 (0.892, 0.927)	0.911 (0.894, 0.929)
$\omega_1 = \omega_2 = \log(1.0)$, $\delta = 25$, $\nu = -0.2$	σ^2_ϕ	0.950 (0.937, 0.963)	0.948 (0.935, 0.962)
$\omega_1 = \omega_2 = \log(1.0)$, $\delta = 25$, $\nu = -0.2$	σ^2_ϵ	0.949 (0.936, 0.963)	0.948 (0.935, 0.962)
$\omega_1 = \omega_2 = \log(1.0)$, $\delta = 25$, $\nu = 0.0$	σ^2_α	0.894 (0.876, 0.913)	0.908 (0.890, 0.926)
$\omega_1 = \omega_2 = \log(1.0)$, $\delta = 25$, $\nu = 0.0$	σ^2_ϕ	0.944 (0.930, 0.958)	0.944 (0.930, 0.958)
$\omega_1 = \omega_2 = \log(1.0)$, $\delta = 25$, $\nu = 0.0$	σ^2_ϵ	0.944 (0.930, 0.958)	0.944 (0.930, 0.958)
$\omega_1 = \omega_2 = \log(2.0)$, $\delta = 0.0$, $\nu = -0.2$	σ^2_α	0.055 (0.041, 0.069)	0.600 (0.562, 0.638)
$\omega_1 = \omega_2 = \log(2.0)$, $\delta = 0.0$, $\nu = -0.2$	σ^2_ϕ	0.000 (0.000, 0.000)	0.685 (0.649, 0.721)
$\omega_1 = \omega_2 = \log(2.0)$, $\delta = 0.0$, $\nu = -0.2$	σ^2_ϵ	0.958 (0.946, 0.970)	0.920 (0.899, 0.941)
$\omega_1 = \omega_2 = \log(2.0)$, $\delta = 0.0$, $\nu = 0.0$	σ^2_α	0.057 (0.043, 0.071)	0.614 (0.574, 0.653)
$\omega_1 = \omega_2 = \log(2.0)$, $\delta = 0.0$, $\nu = 0.0$	σ^2_ϕ	0.000 (0.000, 0.000)	0.668 (0.630, 0.707)
$\omega_1 = \omega_2 = \log(2.0)$, $\delta = 0.0$, $\nu = 0.0$	σ^2_ϵ	0.943 (0.929, 0.957)	0.909 (0.886, 0.933)

Table B24: Coverage probability of variance components for the general time on treatment effect parametrisation, with 95% confidence intervals based on Monte Carlo errors. LMM denotes the linear mixed model, while JM denotes the joint model. *(continued)*

Scenario	Parameter	LMM	JM
$\omega_1 = \omega_2 = \log(2.0)$, $\delta = 5.0$, $\nu = -0.2$	σ^2_α	0.048 (0.035, 0.061)	0.616 (0.577, 0.655)
$\omega_1 = \omega_2 = \log(2.0)$, $\delta = 5.0$, $\nu = -0.2$	σ^2_ϕ	0.000 (0.000, 0.000)	0.667 (0.630, 0.705)
$\omega_1 = \omega_2 = \log(2.0)$, $\delta = 5.0$, $\nu = -0.2$	σ^2_ϵ	0.949 (0.935, 0.962)	0.924 (0.903, 0.945)
$\omega_1 = \omega_2 = \log(2.0)$, $\delta = 5.0$, $\nu = 0.0$	σ^2_α	0.054 (0.040, 0.068)	0.633 (0.594, 0.672)
$\omega_1 = \omega_2 = \log(2.0)$, $\delta = 5.0$, $\nu = 0.0$	σ^2_ϕ	0.000 (0.000, 0.000)	0.695 (0.657, 0.732)
$\omega_1 = \omega_2 = \log(2.0)$, $\delta = 5.0$, $\nu = 0.0$	σ^2_ϵ	0.951 (0.937, 0.964)	0.940 (0.921, 0.959)
$\omega_1 = \omega_2 = \log(2.0)$, $\delta = 25$, $\nu = -0.2$	σ^2_α	0.058 (0.043, 0.072)	0.595 (0.556, 0.634)
$\omega_1 = \omega_2 = \log(2.0)$, $\delta = 25$, $\nu = -0.2$	σ^2_ϕ	0.000 (0.000, 0.000)	0.681 (0.644, 0.718)
$\omega_1 = \omega_2 = \log(2.0)$, $\delta = 25$, $\nu = -0.2$	σ^2_ϵ	0.939 (0.924, 0.954)	0.933 (0.913, 0.952)
$\omega_1 = \omega_2 = \log(2.0)$, $\delta = 25$, $\nu = 0.0$	σ^2_α	0.050 (0.036, 0.063)	0.646 (0.608, 0.684)
$\omega_1 = \omega_2 = \log(2.0)$, $\delta = 25$, $\nu = 0.0$	σ^2_ϕ	0.000 (0.000, 0.000)	0.668 (0.630, 0.706)
$\omega_1 = \omega_2 = \log(2.0)$, $\delta = 25$, $\nu = 0.0$	σ^2_ϵ	0.957 (0.945, 0.970)	0.924 (0.903, 0.946)
**Additional scenarios:**			
$i = 3 \times 4$	σ^2_α	0.696 (0.668, 0.724)	0.861 (0.839, 0.884)
$i = 3 \times 4$	σ^2_ϕ	0.347 (0.318, 0.376)	0.950 (0.936, 0.965)
$i = 3 \times 4$	σ^2_ϵ	0.943 (0.929, 0.958)	0.934 (0.918, 0.950)
$i = 3 \times 4, N = 100$	σ^2_α	0.661 (0.632, 0.689)	0.858 (0.835, 0.881)
$i = 3 \times 4, N = 100$	σ^2_ϕ	0.072 (0.056, 0.088)	0.971 (0.960, 0.982)
$i = 3 \times 4, N = 100$	σ^2_ϵ	0.961 (0.949, 0.972)	0.950 (0.935, 0.964)
$\sigma^2_\alpha = 1, \sigma^2_\phi = 27.5$	σ^2_α	0.846 (0.824, 0.868)	0.943 (0.928, 0.957)
$\sigma^2_\alpha = 1, \sigma^2_\phi = 27.5$	σ^2_ϕ	0.434 (0.404, 0.464)	0.952 (0.938, 0.965)
$\sigma^2_\alpha = 1, \sigma^2_\phi = 27.5$	σ^2_ϵ	0.942 (0.927, 0.956)	0.940 (0.925, 0.954)
$\sigma^2_\alpha = 4, \sigma^2_\phi = 110$	σ^2_α	0.583 (0.553, 0.613)	0.914 (0.896, 0.932)
$\sigma^2_\alpha = 4, \sigma^2_\phi = 110$	σ^2_ϕ	0.000 (0.000, 0.000)	0.951 (0.937, 0.965)
$\sigma^2_\alpha = 4, \sigma^2_\phi = 110$	σ^2_ϵ	0.961 (0.949, 0.973)	0.953 (0.939, 0.967)

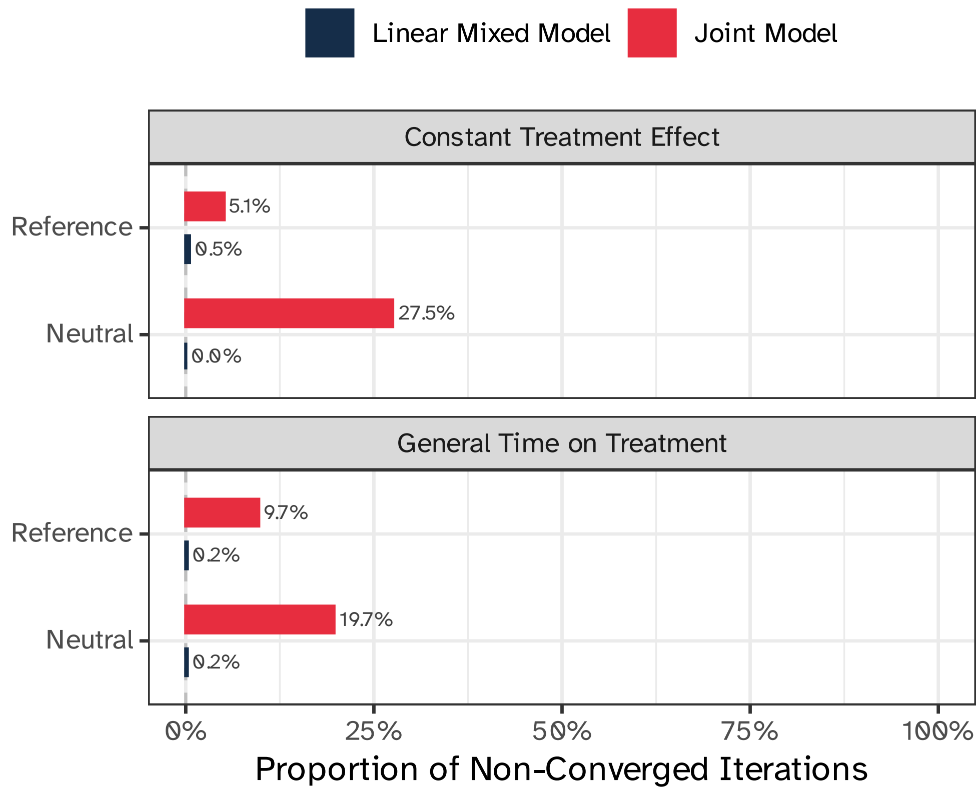

Figure B3: Proportion of non-converged repetitions for the joint and linear mixed models, for both constant intervention and general time on treatment parametrizations, neutral simulation scenario.

B.4 Neutral simulation scenario

Convergence rates for the joint and linear mixed models in the neutral simulation scenario are depicted in Figure B3.

In the next two subsection, we summarise results for the neutral simulation scenarios for both model parametrizations.

B.4.1 Constant intervention model

Bias (with 95% C.I. based on Monte Carlo standard errors) for the treatment effect on the longitudinal outcome are tabulated in Table B25, with relative bias in Table B26. Coverage probabilities are tabulated in Table B27.

Bias, relative bias, and coverage probabilities for the period effects are tabulated in Tables B28, B29 and B30, respectively.

Bias, relative bias, and coverage probabilities for the ICCs are tabulated in Tables B31, B32 and B33.

Finally, bias, relative bias, and coverage probabilities for the variance components are tabulated in Tables B34, B35 and B36.

B.4.2 General time on treatment model

Bias (with 95% C.I. based on Monte Carlo standard errors) for the treatment effect on the longitudinal outcome are tabulated in Table B37, with relative bias in Table B38. Coverage probabilities are tabulated in Table B39.

Bias, relative bias, and coverage probabilities for the period effects are tabulated in Tables B40, B41 and B42, respectively.

Bias, relative bias, and coverage probabilities for the ICCs are tabulated in Tables B43, B44 and B45.

Table B25: Bias of treatment effect on the longitudinal outcome for the constant treatment effect parametrisation, with 95% confidence intervals based on Monte Carlo errors. LMM denotes the linear mixed model, while JM denotes the joint model. Statistically significant biases are highlighted in bold. Comparison of scenarios based on a joint model and on a neutral data-generating mechanism.

Scenario	LMM	JM
Reference	**-0.053 (-0.074, -0.031)**	-0.010 (-0.032, 0.012)
Neutral	**-0.082 (-0.100, -0.063)**	**0.066 (0.041, 0.090)**

Table B26: Relative bias of treatment effect on the longitudinal outcome for the constant treatment effect parametrisation, with 95% confidence intervals based on Monte Carlo errors. LMM denotes the linear mixed model, while JM denotes the joint model. Statistically significant biases are highlighted in bold. Comparison of scenarios based on a joint model and on a neutral data-generating mechanism.

Scenario	LMM	JM
Reference	**-0.011 (-0.015, -0.006)**	-0.002 (-0.006, 0.002)
Neutral	**-0.016 (-0.020, -0.013)**	**0.013 (0.008, 0.018)**

Table B27: Coverage probability of treatment effect on the longitudinal outcome for the constant treatment effect parametrisation, with 95% confidence intervals based on Monte Carlo errors. LMM denotes the linear mixed model, while JM denotes the joint model. Comparison of scenarios based on a joint model and on a neutral data-generating mechanism.

Scenario	LMM	JM
Reference	0.952 (0.938, 0.965)	0.959 (0.946, 0.972)
Neutral	0.939 (0.924, 0.954)	0.917 (0.897, 0.937)

Table B28: Bias of period effects on the longitudinal outcome for the constant treatment effect parametrisation, with 95% confidence intervals based on Monte Carlo errors. LMM denotes the linear mixed model, while JM denotes the joint model. Statistically significant biases are highlighted in bold. Comparison of scenarios based on a joint model and on a neutral data-generating mechanism.

Scenario	Parameter	LMM	JM
Reference	β_1	**1.468 (1.446, 1.489)**	-0.006 (-0.031, 0.019)
Reference	β_2	**1.957 (1.933, 1.981)**	-0.014 (-0.043, 0.014)
Reference	β_3	**2.223 (2.197, 2.249)**	-0.004 (-0.035, 0.027)
Reference	β_4	**2.382 (2.352, 2.411)**	-0.007 (-0.041, 0.027)
Reference	β_5	**2.490 (2.456, 2.524)**	-0.009 (-0.047, 0.029)
Neutral	β_1	**-0.493 (-0.515, -0.471)**	**-0.204 (-0.230, -0.179)**
Neutral	β_2	**-0.765 (-0.787, -0.743)**	**-0.317 (-0.343, -0.291)**
Neutral	β_3	**-0.967 (-0.991, -0.943)**	**-0.425 (-0.454, -0.395)**
Neutral	β_4	**-1.103 (-1.129, -1.077)**	**-0.519 (-0.551, -0.487)**
Neutral	β_5	**-1.435 (-1.468, -1.403)**	**-0.578 (-0.623, -0.534)**

Table B29: Relative bias of period effects on the longitudinal outcome for the constant treatment effect parametrisation, with 95% confidence intervals based on Monte Carlo errors. LMM denotes the linear mixed model, while JM denotes the joint model. Statistically significant biases are highlighted in bold. Comparison of scenarios based on a joint model and on a neutral data-generating mechanism.

Scenario	Parameter	LMM	JM
Reference	β_1	**0.049 (0.048, 0.050)**	-0.000 (-0.001, 0.001)
Reference	β_2	**0.065 (0.064, 0.066)**	-0.000 (-0.001, 0.000)
Reference	β_3	**0.074 (0.073, 0.075)**	-0.000 (-0.001, 0.001)
Reference	β_4	**0.079 (0.078, 0.080)**	-0.000 (-0.001, 0.001)
Reference	β_5	**0.083 (0.082, 0.084)**	-0.000 (-0.002, 0.001)
Neutral	β_1	**-0.016 (-0.017, -0.016)**	**-0.007 (-0.008, -0.006)**
Neutral	β_2	**-0.026 (-0.026, -0.025)**	**-0.011 (-0.011, -0.010)**
Neutral	β_3	**-0.032 (-0.033, -0.031)**	**-0.014 (-0.015, -0.013)**
Neutral	β_4	**-0.037 (-0.038, -0.036)**	**-0.017 (-0.018, -0.016)**
Neutral	β_5	**-0.048 (-0.049, -0.047)**	**-0.019 (-0.021, -0.018)**

Table B30: Coverage probability of period effects on the longitudinal outcome for the constant treatment effect parametrisation, with 95% confidence intervals based on Monte Carlo errors. LMM denotes the linear mixed model, while JM denotes the joint model. Comparison of scenarios based on a joint model and on a neutral data-generating mechanism.

Scenario	Parameter	LMM	JM
Reference	β_1	0.016 (0.008, 0.024)	0.940 (0.925, 0.955)
Reference	β_2	0.001 (-0.001, 0.003)	0.945 (0.931, 0.960)
Reference	β_3	0.000 (0.000, 0.000)	0.944 (0.930, 0.959)
Reference	β_4	0.000 (0.000, 0.000)	0.954 (0.940, 0.967)
Reference	β_5	0.003 (-0.000, 0.006)	0.954 (0.940, 0.967)
Neutral	β_1	0.664 (0.635, 0.693)	0.886 (0.862, 0.909)
Neutral	β_2	0.373 (0.343, 0.403)	0.855 (0.830, 0.881)
Neutral	β_3	0.266 (0.239, 0.293)	0.779 (0.749, 0.809)
Neutral	β_4	0.242 (0.215, 0.269)	0.766 (0.735, 0.796)
Neutral	β_5	0.197 (0.172, 0.222)	0.806 (0.777, 0.834)

Table B31: Bias of ICCs for the constant treatment effect parametrisation, with 95% confidence intervals based on Monte Carlo errors. LMM denotes the linear mixed model, while JM denotes the joint model. Statistically significant biases are highlighted in bold. Comparison of scenarios based on a joint model and on a neutral data-generating mechanism.

Scenario	Parameter	LMM	JM
Reference	ρ_a	**-0.053 (-0.054, -0.052)**	0.000 (-0.001, 0.001)
Reference	ρ_d	**-0.005 (-0.006, -0.004)**	-0.001 (-0.001, 0.000)
Neutral	ρ_a	**-0.037 (-0.038, -0.036)**	**-0.015 (-0.017, -0.012)**
Neutral	ρ_d	**-0.004 (-0.004, -0.003)**	-0.001 (-0.001, 0.000)

Table B32: Relative bias of ICCs for the constant treatment effect parametrisation, with 95% confidence intervals based on Monte Carlo errors. LMM denotes the linear mixed model, while JM denotes the joint model. Statistically significant biases are highlighted in bold. Comparison of scenarios based on a joint model and on a neutral data-generating mechanism.

Scenario	Parameter	LMM	JM
Reference	ρ_a	**-0.091 (-0.092, -0.089)**	0.000 (-0.002, 0.002)
Reference	ρ_d	**-0.242 (-0.267, -0.217)**	-0.026 (-0.054, 0.003)
Neutral	ρ_a	**-0.064 (-0.065, -0.062)**	**-0.025 (-0.029, -0.021)**
Neutral	ρ_d	**-0.193 (-0.216, -0.170)**	-0.033 (-0.069, 0.004)

Table B33: Coverage probability of ICCs for the constant treatment effect parametrisation, with 95% confidence intervals based on Monte Carlo errors. LMM denotes the linear mixed model, while JM denotes the joint model. Comparison of scenarios based on a joint model and on a neutral data-generating mechanism.

Scenario	Parameter	LMM	JM
Reference	ρ_a	0.070 (0.054, 0.086)	0.949 (0.935, 0.963)
Reference	ρ_d	0.832 (0.809, 0.855)	0.925 (0.908, 0.942)
Neutral	ρ_a	0.217 (0.191, 0.243)	0.865 (0.840, 0.890)
Neutral	ρ_d	0.840 (0.817, 0.863)	0.901 (0.879, 0.922)

Table B34: Bias of variance components for the constant treatment effect parametrisation, with 95% confidence intervals based on Monte Carlo errors. LMM denotes the linear mixed model, while JM denotes the joint model. Statistically significant biases are highlighted in bold. Comparison of scenarios based on a joint model and on a neutral data-generating mechanism.

Scenario	Parameter	LMM	JM
Reference	σ^2_α	**-0.644 (-0.690, -0.599)**	-0.049 (-0.107, 0.009)
Reference	σ^2_ϕ	**-10.092 (-10.247, -9.936)**	0.022 (-0.194, 0.237)
Reference	σ^2_ϵ	**0.265 (0.202, 0.329)**	**-0.100 (-0.163, -0.036)**
Neutral	σ^2_α	**-0.505 (-0.549, -0.462)**	**-0.093 (-0.178, -0.007)**
Neutral	σ^2_ϕ	**-7.126 (-7.275, -6.977)**	**-2.919 (-3.164, -2.674)**
Neutral	σ^2_ϵ	**0.301 (0.247, 0.356)**	**0.396 (0.011, 0.781)**

Table B35: Relative bias of variance components for the constant treatment effect parametrisation, with 95% confidence intervals based on Monte Carlo errors. LMM denotes the linear mixed model, while JM denotes the joint model. Statistically significant biases are highlighted in bold. Comparison of scenarios based on a joint model and on a neutral data-generating mechanism.

Scenario	Parameter	LMM	JM
Reference	σ^2_α	**-0.322 (-0.345, -0.299)**	-0.025 (-0.054, 0.004)
Reference	σ^2_ϕ	**-0.183 (-0.186, -0.181)**	0.000 (-0.004, 0.004)
Reference	σ^2_ϵ	**0.007 (0.005, 0.008)**	**-0.002 (-0.004, -0.001)**
Neutral	σ^2_α	**-0.253 (-0.274, -0.231)**	**-0.046 (-0.089, -0.004)**
Neutral	σ^2_ϕ	**-0.130 (-0.132, -0.127)**	**-0.053 (-0.058, -0.049)**
Neutral	σ^2_ϵ	**0.008 (0.006, 0.009)**	**0.010 (0.000, 0.020)**

Table B36: Coverage probability of variance components for the constant treatment effect parametrisation, with 95% confidence intervals based on Monte Carlo errors. LMM denotes the linear mixed model, while JM denotes the joint model. Comparison of scenarios based on a joint model and on a neutral data-generating mechanism.

Scenario	Parameter	LMM	JM
Reference	σ^2_α	0.755 (0.728, 0.782)	0.921 (0.904, 0.938)
Reference	σ^2_ϕ	0.029 (0.019, 0.040)	0.947 (0.933, 0.962)
Reference	σ^2_ϵ	0.947 (0.933, 0.961)	0.953 (0.939, 0.966)
Neutral	σ^2_α	0.791 (0.766, 0.816)	0.888 (0.865, 0.911)
Neutral	σ^2_ϕ	0.147 (0.125, 0.169)	0.782 (0.752, 0.812)
Neutral	σ^2_ϵ	0.941 (0.926, 0.956)	0.917 (0.897, 0.937)

Table B37: Bias of treatment effect on the longitudinal outcome for the general time on treatment effect parametrisation, with 95% confidence intervals based on Monte Carlo errors. LMM denotes the linear mixed model, while JM denotes the joint model. Statistically significant biases are highlighted in bold. Comparison of scenarios based on a joint model and on a neutral data-generating mechanism.

Scenario	Parameter	LMM	JM
Reference	δ_0	**-0.059 (-0.082, -0.036)**	-0.000 (-0.025, 0.024)
Reference	δ_1	**-0.119 (-0.150, -0.087)**	-0.023 (-0.057, 0.010)
Reference	δ_2	**-0.159 (-0.201, -0.117)**	-0.025 (-0.069, 0.019)
Reference	δ_3	**-0.210 (-0.271, -0.150)**	-0.030 (-0.094, 0.035)
Neutral	δ_0	**-0.038 (-0.057, -0.018)**	**0.119 (0.097, 0.141)**
Neutral	δ_1	**-0.099 (-0.124, -0.073)**	**0.165 (0.136, 0.194)**
Neutral	δ_2	**-0.156 (-0.191, -0.121)**	**0.223 (0.184, 0.263)**
Neutral	δ_3	**-0.313 (-0.368, -0.259)**	**0.179 (0.116, 0.242)**

Finally, bias, relative bias, and coverage probabilities for the variance components are tabulated in Tables B46, B47 and B48.

Table B38: Relative bias of treatment effect on the longitudinal outcome for the general time on treatment effect parametrisation, with 95% confidence intervals based on Monte Carlo errors. LMM denotes the linear mixed model, while JM denotes the joint model. Statistically significant biases are highlighted in bold. Comparison of scenarios based on a joint model and on a neutral data-generating mechanism.

Scenario	Parameter	LMM	JM
Reference	δ_0	—	—
Reference	δ_1	**-0.047 (-0.060, -0.035)**	-0.009 (-0.023, 0.004)
Reference	δ_2	**-0.032 (-0.040, -0.023)**	-0.005 (-0.014, 0.004)
Reference	δ_3	**-0.034 (-0.043, -0.024)**	-0.005 (-0.015, 0.006)
Neutral	δ_0	—	—
Neutral	δ_1	**-0.040 (-0.050, -0.029)**	**0.066 (0.055, 0.078)**
Neutral	δ_2	**-0.031 (-0.038, -0.024)**	**0.045 (0.037, 0.053)**
Neutral	δ_3	**-0.050 (-0.059, -0.041)**	**0.029 (0.019, 0.039)**

Table B39: Coverage probability of treatment effect on the longitudinal outcome for the general time on treatment effect parametrisation, with 95% confidence intervals based on Monte Carlo errors. LMM denotes the linear mixed model, while JM denotes the joint model. Comparison of scenarios based on a joint model and on a neutral data-generating mechanism.

Scenario	Parameter	LMM	JM
Reference	δ_0	0.948 (0.934, 0.962)	0.950 (0.936, 0.964)
Reference	δ_1	0.939 (0.924, 0.954)	0.945 (0.930, 0.960)
Reference	δ_2	0.948 (0.934, 0.962)	0.946 (0.931, 0.961)
Reference	δ_3	0.942 (0.927, 0.956)	0.931 (0.915, 0.948)
Neutral	δ_0	0.945 (0.931, 0.959)	0.918 (0.899, 0.937)
Neutral	δ_1	0.941 (0.926, 0.956)	0.930 (0.913, 0.948)
Neutral	δ_2	0.941 (0.926, 0.956)	0.930 (0.913, 0.948)
Neutral	δ_3	0.938 (0.923, 0.953)	0.940 (0.924, 0.957)

Table B40: Bias of period effects on the longitudinal outcome for the general time on treatment effect parametrisation, with 95% confidence intervals based on Monte Carlo errors. LMM denotes the linear mixed model, while JM denotes the joint model. Statistically significant biases are highlighted in bold. Comparison of scenarios based on a joint model and on a neutral data-generating mechanism.

Scenario	Parameter	LMM	JM
Reference	β_1	**1.460 (1.438, 1.481)**	-0.021 (-0.047, 0.004)
Reference	β_2	**1.972 (1.949, 1.994)**	-0.011 (-0.040, 0.017)
Reference	β_3	**2.244 (2.219, 2.269)**	-0.009 (-0.040, 0.022)
Reference	β_4	**2.446 (2.415, 2.478)**	0.009 (-0.028, 0.046)
Reference	β_5	**2.599 (2.559, 2.639)**	0.015 (-0.031, 0.061)
Neutral	β_1	**-0.544 (-0.566, -0.522)**	**-0.224 (-0.248, -0.201)**
Neutral	β_2	**-0.832 (-0.854, -0.809)**	**-0.355 (-0.380, -0.331)**
Neutral	β_3	**-1.025 (-1.050, -1.000)**	**-0.482 (-0.510, -0.454)**
Neutral	β_4	**-1.149 (-1.178, -1.120)**	**-0.613 (-0.646, -0.579)**
Neutral	β_5	**-1.410 (-1.446, -1.373)**	**-0.651 (-0.695, -0.608)**

Table B41: Relative bias of period effects on the longitudinal outcome for the general time on treatment effect parametrisation, with 95% confidence intervals based on Monte Carlo errors. LMM denotes the linear mixed model, while JM denotes the joint model. Statistically significant biases are highlighted in bold. Comparison of scenarios based on a joint model and on a neutral data-generating mechanism.

Scenario	Parameter	LMM	JM
Reference	β_1	**0.049 (0.048, 0.049)**	-0.001 (-0.002, 0.000)
Reference	β_2	**0.066 (0.065, 0.066)**	-0.000 (-0.001, 0.001)
Reference	β_3	**0.075 (0.074, 0.076)**	-0.000 (-0.001, 0.001)
Reference	β_4	**0.082 (0.081, 0.083)**	0.000 (-0.001, 0.002)
Reference	β_5	**0.087 (0.085, 0.088)**	0.001 (-0.001, 0.002)
Neutral	β_1	**-0.018 (-0.019, -0.017)**	**-0.007 (-0.008, -0.007)**
Neutral	β_2	**-0.028 (-0.028, -0.027)**	**-0.012 (-0.013, -0.011)**
Neutral	β_3	**-0.034 (-0.035, -0.033)**	**-0.016 (-0.017, -0.015)**
Neutral	β_4	**-0.038 (-0.039, -0.037)**	**-0.020 (-0.022, -0.019)**
Neutral	β_5	**-0.047 (-0.048, -0.046)**	**-0.022 (-0.023, -0.020)**

Table B42: Coverage probability of period effects on the longitudinal outcome for the general time on treatment effect parametrisation, with 95% confidence intervals based on Monte Carlo errors. LMM denotes the linear mixed model, while JM denotes the joint model. Comparison of scenarios based on a joint model and on a neutral data-generating mechanism.

Scenario	Parameter	LMM	JM
Reference	β_1	0.009 (0.003, 0.015)	0.948 (0.933, 0.962)
Reference	β_2	0.000 (0.000, 0.000)	0.951 (0.937, 0.965)
Reference	β_3	0.001 (-0.001, 0.003)	0.950 (0.936, 0.964)
Reference	β_4	0.003 (-0.000, 0.006)	0.958 (0.945, 0.971)
Reference	β_5	0.030 (0.019, 0.041)	0.948 (0.933, 0.962)
Neutral	β_1	0.609 (0.579, 0.639)	0.887 (0.865, 0.909)
Neutral	β_2	0.308 (0.279, 0.336)	0.828 (0.802, 0.854)
Neutral	β_3	0.237 (0.211, 0.264)	0.757 (0.728, 0.787)
Neutral	β_4	0.275 (0.247, 0.302)	0.716 (0.685, 0.747)
Neutral	β_5	0.342 (0.312, 0.371)	0.797 (0.769, 0.825)

Table B43: Bias of ICCs for the general time on treatment effect parametrisation, with 95% confidence intervals based on Monte Carlo errors. LMM denotes the linear mixed model, while JM denotes the joint model. Statistically significant biases are highlighted in bold. Comparison of scenarios based on a joint model and on a neutral data-generating mechanism.

Scenario	Parameter	LMM	JM
Reference	ρ_a	**-0.053 (-0.054, -0.052)**	0.001 (-0.000, 0.002)
Reference	ρ_d	**-0.005 (-0.006, -0.005)**	-0.000 (-0.001, 0.000)
Neutral	ρ_a	**-0.038 (-0.039, -0.037)**	**-0.012 (-0.013, -0.011)**
Neutral	ρ_d	**-0.004 (-0.004, -0.003)**	**-0.001 (-0.001, -0.000)**

Table B44: Relative bias of ICCs for the general time on treatment effect parametrisation, with 95% confidence intervals based on Monte Carlo errors. LMM denotes the linear mixed model, while JM denotes the joint model. Statistically significant biases are highlighted in bold. Comparison of scenarios based on a joint model and on a neutral data-generating mechanism.

Scenario	Parameter	LMM	JM
Reference	ρ_a	**-0.090 (-0.092, -0.088)**	0.001 (-0.001, 0.003)
Reference	ρ_d	**-0.255 (-0.280, -0.229)**	-0.014 (-0.044, 0.016)
Neutral	ρ_a	**-0.065 (-0.066, -0.063)**	**-0.021 (-0.022, -0.019)**
Neutral	ρ_d	**-0.178 (-0.201, -0.154)**	**-0.040 (-0.068, -0.012)**

Table B45: Coverage probability of ICCs for the general time on treatment effect parametrisation, with 95% confidence intervals based on Monte Carlo errors. LMM denotes the linear mixed model, while JM denotes the joint model. Comparison of scenarios based on a joint model and on a neutral data-generating mechanism.

Scenario	Parameter	LMM	JM
Reference	ρ_a	0.087 (0.070, 0.105)	0.949 (0.935, 0.963)
Reference	ρ_d	0.807 (0.782, 0.831)	0.928 (0.911, 0.945)
Neutral	ρ_a	0.190 (0.166, 0.215)	0.875 (0.853, 0.898)
Neutral	ρ_d	0.850 (0.828, 0.872)	0.914 (0.895, 0.933)

Table B46: Bias of variance components for the general time on treatment effect parametrisation, with 95% confidence intervals based on Monte Carlo errors. LMM denotes the linear mixed model, while JM denotes the joint model. Statistically significant biases are highlighted in bold. Comparison of scenarios based on a joint model and on a neutral data-generating mechanism.

Scenario	Parameter	LMM	JM
Reference	σ^2_α	**-0.668 (-0.714, -0.622)**	-0.024 (-0.085, 0.036)
Reference	σ^2_ϕ	**-10.051 (-10.210, -9.891)**	0.141 (-0.094, 0.375)
Reference	σ^2_ϵ	**0.208 (0.142, 0.273)**	**-0.132 (-0.201, -0.063)**
Neutral	σ^2_α	**-0.478 (-0.522, -0.435)**	**-0.137 (-0.192, -0.082)**
Neutral	σ^2_ϕ	**-7.290 (-7.436, -7.144)**	**-2.784 (-2.966, -2.601)**
Neutral	σ^2_ϵ	**0.302 (0.249, 0.354)**	**-0.153 (-0.264, -0.042)**

Table B47: Relative bias of variance components for the general time on treatment effect parametrisation, with 95% confidence intervals based on Monte Carlo errors. LMM denotes the linear mixed model, while JM denotes the joint model. Statistically significant biases are highlighted in bold. Comparison of scenarios based on a joint model and on a neutral data-generating mechanism.

Scenario	Parameter	LMM	JM
Reference	σ^2_α	**-0.334 (-0.357, -0.311)**	-0.012 (-0.042, 0.018)
Reference	σ^2_ϕ	**-0.183 (-0.186, -0.180)**	0.003 (-0.002, 0.007)
Reference	σ^2_ϵ	**0.005 (0.004, 0.007)**	**-0.003 (-0.005, -0.002)**
Neutral	σ^2_α	**-0.239 (-0.261, -0.217)**	**-0.068 (-0.096, -0.041)**
Neutral	σ^2_ϕ	**-0.133 (-0.135, -0.130)**	**-0.051 (-0.054, -0.047)**
Neutral	σ^2_ϵ	**0.008 (0.006, 0.009)**	**-0.004 (-0.007, -0.001)**

Table B48: Coverage probability of variance components for the general time on treatment effect parametrisation, with 95% confidence intervals based on Monte Carlo errors. LMM denotes the linear mixed model, while JM denotes the joint model. Comparison of scenarios based on a joint model and on a neutral data-generating mechanism.

Scenario	Parameter	LMM	JM
Reference	σ_α^2	0.738 (0.711, 0.766)	0.926 (0.909, 0.943)
Reference	σ_ϕ^2	0.031 (0.020, 0.042)	0.939 (0.923, 0.955)
Reference	σ_ϵ^2	0.939 (0.924, 0.954)	0.934 (0.917, 0.950)
Neutral	σ_α^2	0.792 (0.766, 0.817)	0.902 (0.881, 0.922)
Neutral	σ_ϕ^2	0.131 (0.110, 0.152)	0.775 (0.746, 0.803)
Neutral	σ_ϵ^2	0.950 (0.936, 0.963)	0.946 (0.931, 0.962)

B.5 Monte Carlo standard errors

The distribution of Monte Carlo standard errors for bias and every model parameter is depicted, for both model parametrizations, in Figure B4 and B5. Monte Carlo standard errors are consistently within the acceptable range (as defined in Section A) for the key parameters of interest, and were larger — as expected — in scenarios with a lower proportion of converged repetitions (Figure B5) and for parameters of larger magnitude.

Moreover, focusing on relative bias of the treatment effect parameters, we obtain Figure B6 and B7: Monte Carlo standard errors for relative bias were consistently below 1%, which we also deemed acceptable.

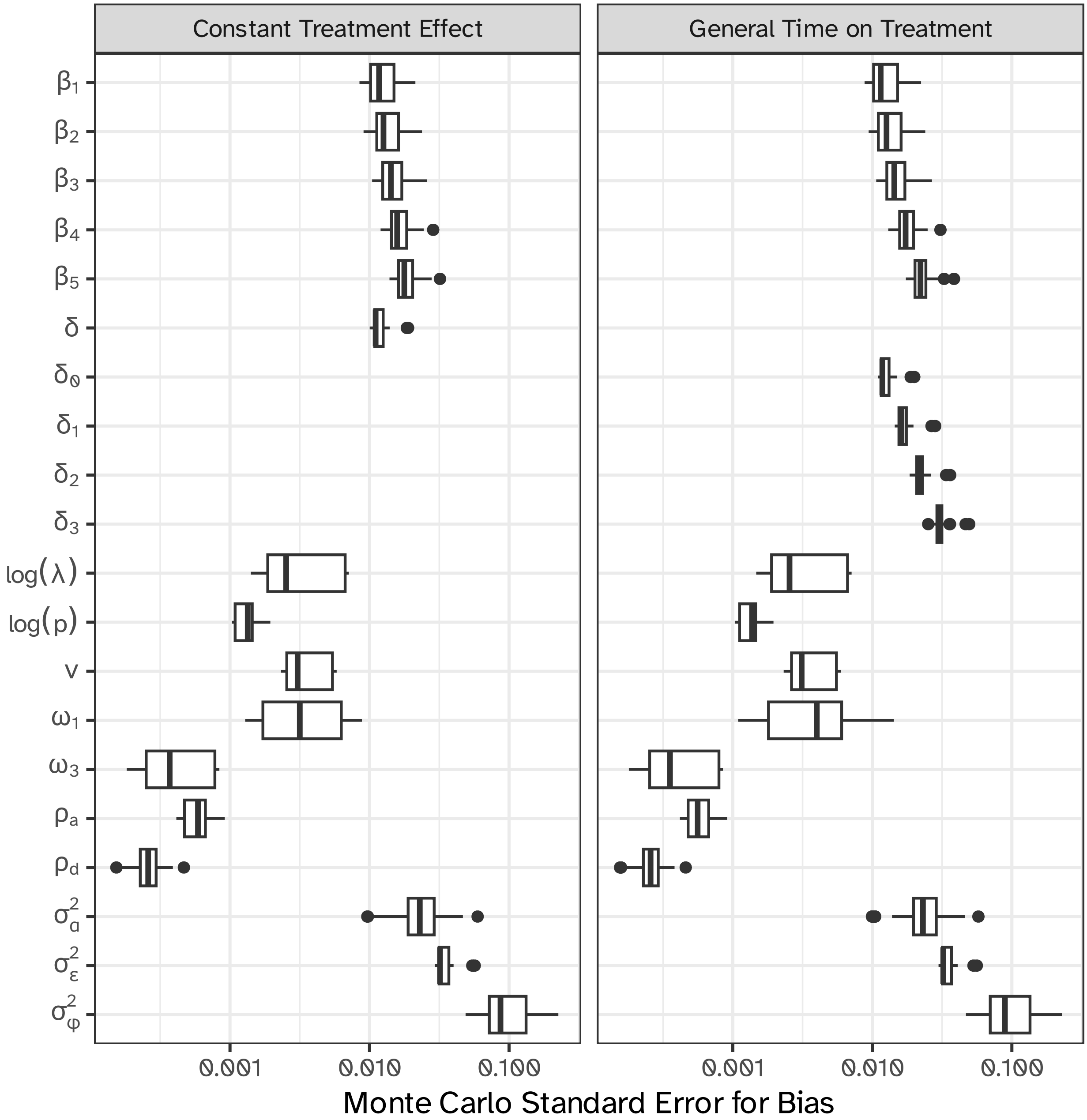

Figure B4: Distribution of Monte Carlo standard errors for bias across all model parameters and for both model parametrizations.

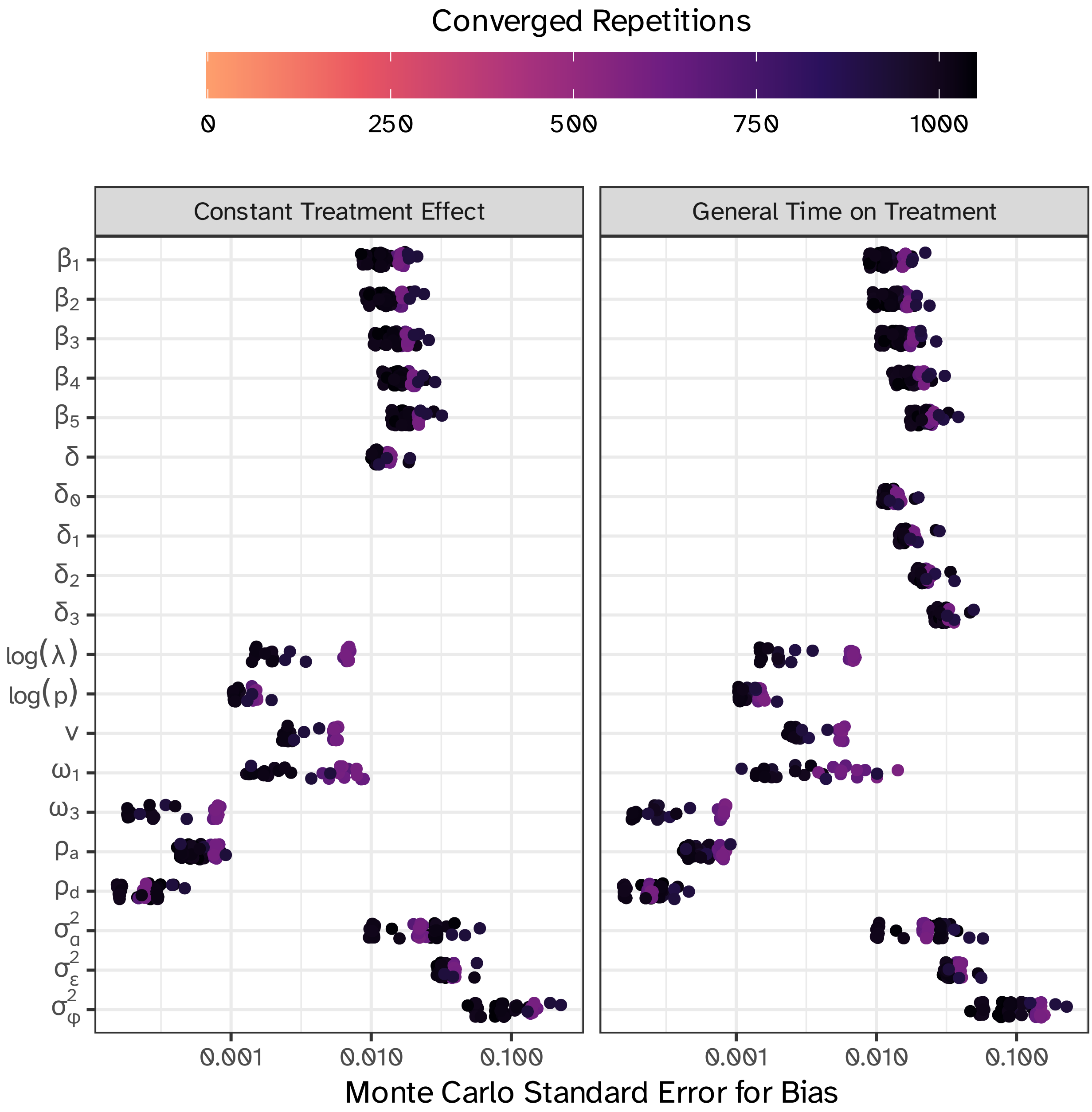

Figure B5: Distribution of Monte Carlo standard errors for bias by number of converged repetitions for a certain model and data-generating mechanism, across all model parameters and for both model parametrizations.

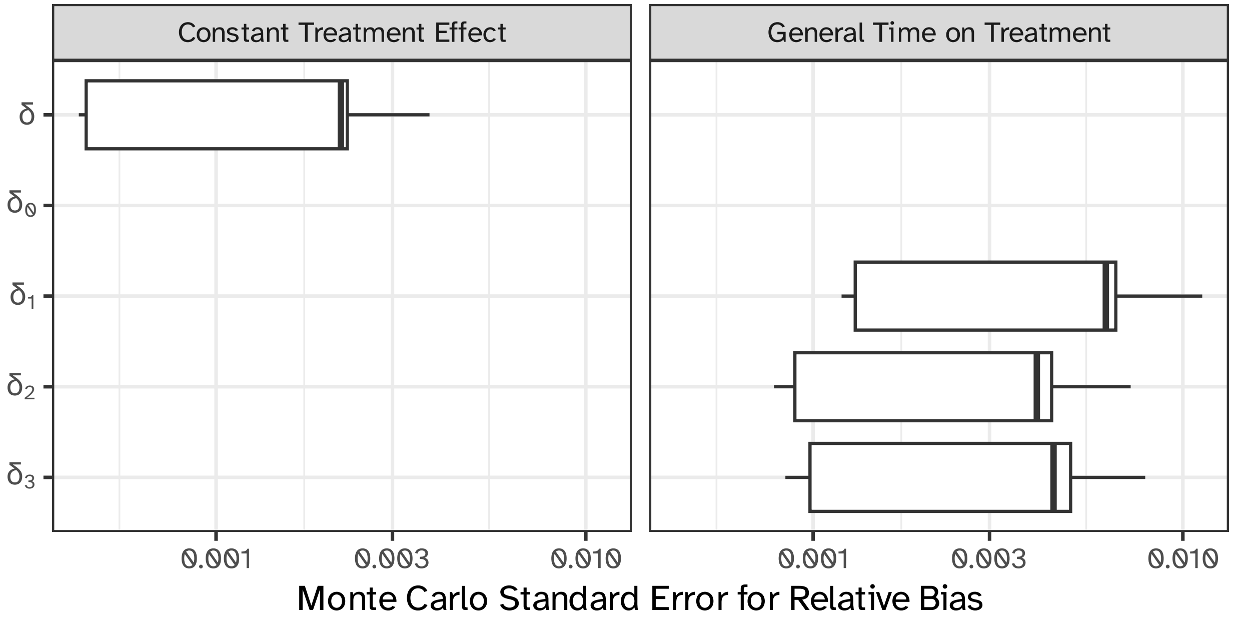

Figure B6: Distribution of Monte Carlo standard errors for relative bias across all treatment effect parameters and for both model parametrizations.

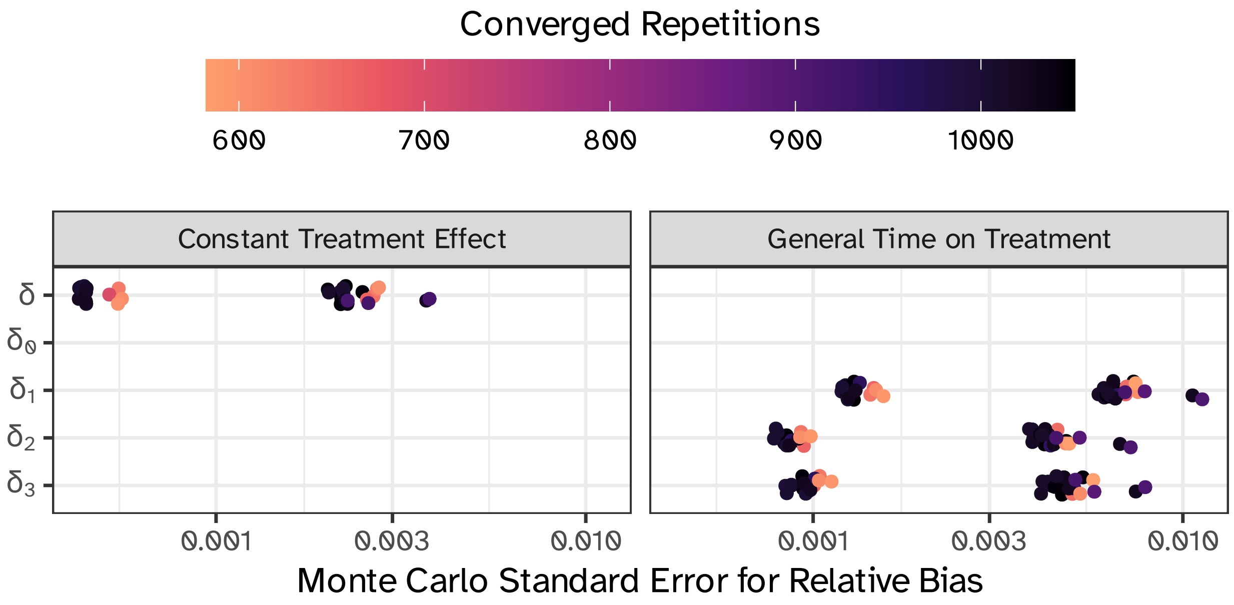

Figure B7: Distribution of Monte Carlo standard errors for relative bias by number of converged repetitions for a certain model and data-generating mechanism, across all treatment effect parameters and for both model parametrizations.

Appendix C Improving convergence of the joint model estimation procedure

As reported in Appendix B.1, the joint model had lower convergence rates than the linear mixed-effects model. In this Section, we discuss possible steps to improve the convergence of the joint model estimation procedure.

The first possible step consists of using a different estimation algorithm. According to our experience, the Broyden-Fletcher-Goldfarb-Shanno (BFGS) algorithm is our first choice (argument `technique(bfgs)` when calling `gsem` in Stata) and it is therefore what we have been using for the simulation study reported in this manuscript. Another option is the default in Stata, its modified Newton-Raphson algorithm[1] (argument `technique(nr)`, or omitting the argument altogether).

We also recommend using the `startgrid` option when calling `gsem`: this uses a grid of possible starting values for the variances of the random effects, leading to better starting values and thus improved convergence rates.

The next possible step to improve the convergence of the estimation algorithm consists of increasing the number of quadrature points used by the adaptive Gaussian quadrature procedure in `gsem`. The default is to use 7 integration points, and increasing the number increases accuracy at the cost of increased computational time, with computational time roughly proportional to the number specified. This is controlled by the `intpoints` option of `gsem`.

Sometimes the likelihood function may be difficult to maximize with default settings because of non-concave regions. We can instruct Stata to use a different stepping algorithm for non-concave regions via the `difficult` option, which may improve convergence rates in some settings. Nonetheless, there is no guarantee that with `difficult` it will work better, sometimes it is better and sometimes worse.

Then, a useful option to improve convergence rates is that of providing custom starting values to `gsem` via the `from` option. For instance, one may first fit a linear mixed-effects model, completely ignoring the dropout process, and then pass these values to `gsem`. The likelihood maximization algorithm will thus start from a much better point, compared to default settings, once again improving convergence rates.

Finally, note that it is not guaranteed that following any of the steps above (or a combination thereof) will lead to convergence of a joint model fit using `gsem`. Sometimes certain parameters of the joint model may be on the boundary of the parameter space (e.g., random effect variances of zero) or not identifiable (e.g., because of not having enough data). In those settings, one may consider simplifying the model instead or using a different estimation package altogether (such as `merlin` in Stata[2]).

The steps outlined above are illustrated in practice with an applied example that we include in the public project repository on GitHub: https://github.com/ellessenne/swjm. These data and code are archived on Zenodo as well.[3]

Appendix D Reanalysis of the 'Frail Older Adults: Care in Transition' trial, additional results

In the main body of the manuscript, we re-analyzed the 'Frail Older Adults: Care in Transition' (ACT) trial applying the joint modeling approach[4]. In this appendix we include additional results that are referred to but not included in the manuscript.

Specifically, estimated treatment effects according to each model are tabulated in Table D49, with estimated period effects in Table D50. Period effects are also plotted in Figure D8. Then, estimated ICC values are tabulated in Table D51, with variance components in Table D52 and Figure D9.

Table D49: Estimated treatment effect parameters for the constant intervention and general time on treatment joint and linear mixed model, with 95% confidence intervals.

	Constant Treatment Effect		General Time on Treatment	
Period Effect	Joint Model	LMM	Joint Model	LMM
Outcome: SF-12 MCS				
δ	0.301 (-0.487, 1.090)	0.202 (-0.551, 0.955)	—	—
δ_0	—	—	0.450 (-0.385, 1.284)	0.269 (-0.526, 1.063)
δ_1	—	—	-0.013 (-1.059, 1.034)	-0.236 (-1.276, 0.803)
δ_2	—	—	0.407 (-0.891, 1.705)	0.063 (-1.278, 1.404)
δ_3	—	—	0.627 (-1.077, 2.332)	-0.233 (-2.017, 1.551)
Outcome: SF-12 PCS				
δ	-0.121 (-0.844, 0.602)	-0.024 (-0.687, 0.639)	—	—
δ_0	—	—	-0.374 (-1.135, 0.387)	-0.323 (-1.032, 0.387)
δ_1	—	—	0.198 (-0.766, 1.163)	0.301 (-0.636, 1.237)
δ_2	—	—	-0.275 (-1.483, 0.932)	-0.070 (-1.290, 1.150)
δ_3	—	—	-1.226 (-2.823, 0.371)	-1.014 (-2.642, 0.613)

Table D50: Estimated period effects for the constant intervention and general time on treatment joint and linear mixed model, with 95% confidence intervals.

	Constant Treatment Effect		General Time on Treatment	
Period Effect	Joint Model	LMM	Joint Model	LMM
Outcome: SF-12 MCS				
β_1	47.957 (44.374, 51.541)	48.327 (43.426, 53.228)	47.989 (44.402, 51.575)	48.303 (43.404, 53.202)
β_2	47.917 (44.309, 51.525)	48.370 (43.438, 53.302)	47.891 (44.279, 51.503)	48.320 (43.391, 53.248)
β_3	48.950 (45.309, 52.592)	49.509 (44.541, 54.476)	49.070 (45.423, 52.718)	49.638 (44.671, 54.605)
β_4	49.206 (45.524, 52.888)	49.792 (44.782, 54.801)	49.231 (45.536, 52.925)	49.896 (44.873, 54.919)
β_5	50.046 (46.315, 53.777)	50.538 (45.481, 55.595)	49.970 (46.193, 53.747)	50.779 (45.670, 55.888)
Outcome: SF-12 PCS				
β_1	41.517 (38.226, 44.807)	39.535 (34.965, 44.105)	41.407 (38.114, 44.701)	39.480 (34.908, 44.051)
β_2	42.612 (39.304, 45.921)	40.574 (35.975, 45.172)	42.601 (39.289, 45.913)	40.633 (36.033, 45.233)
β_3	42.954 (39.617, 46.291)	40.837 (36.207, 45.466)	42.776 (39.436, 46.116)	40.719 (36.085, 45.354)
β_4	41.621 (38.251, 44.992)	39.508 (34.840, 44.175)	41.558 (38.179, 44.937)	39.465 (34.778, 44.153)
β_5	40.730 (37.316, 44.144)	38.534 (33.825, 43.243)	41.042 (37.589, 44.496)	38.843 (34.075, 43.611)

Table D51: Estimated ICC values for the constant intervention and general time on treatment joint and linear mixed model, with 95% confidence intervals.

	Constant Treatment Effect		General Time on Treatment	
ICC	Joint Model	LMM	Joint Model	LMM
Outcome: SF-12 MCS				
ρ_a	0.328 (0.287, 0.370)	0.416 (0.384, 0.448)	0.329 (0.287, 0.370)	0.416 (0.384, 0.448)
ρ_d	0.003 (0.000, 0.013)	0.004 (0.000, 0.013)	0.003 (0.000, 0.013)	0.004 (0.000, 0.013)
Outcome: SF-12 PCS				
ρ_a	0.344 (0.303, 0.386)	0.465 (0.433, 0.497)	0.344 (0.303, 0.386)	0.466 (0.434, 0.498)
ρ_d	0.030 (0.000, 0.060)	0.015 (0.000, 0.034)	0.030 (0.000, 0.061)	0.016 (0.000, 0.035)

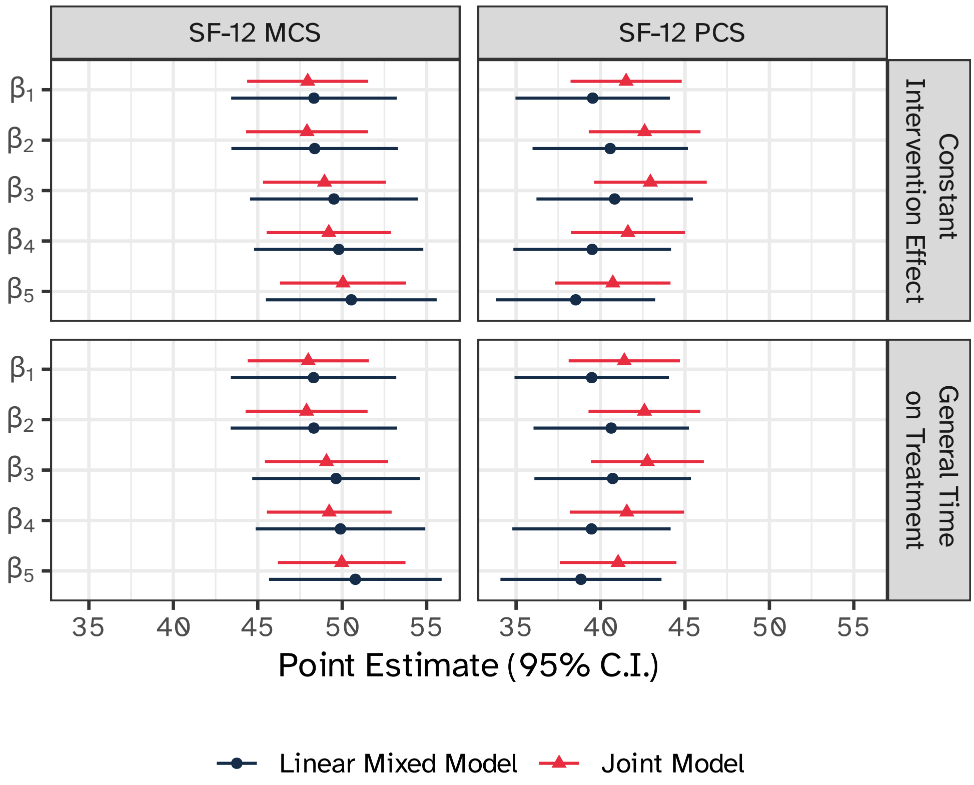

Figure D8: Estimated period effect parameters for the SF-12 MCS and PCS outcomes, according to both the constant intervention and general time on treatment joint and linear mixed models, with 95% confidence intervals.

Table D52: Estimated variance components for the constant intervention and general time on treatment joint and linear mixed model, with 95% confidence intervals.

	Constant Treatment Effect		General Time on Treatment	
Variance	Joint Model	LMM	Joint Model	LMM
Outcome: SF-12 MCS				
σ^2_α	0.363 (0.025, 5.239)	0.366 (0.036, 3.690)	0.369 (0.026, 5.272)	0.326 (0.026, 4.143)
σ^2_ϕ	34.436 (28.833, 41.129)	37.098 (32.971, 41.741)	34.440 (28.836, 41.133)	37.107 (32.980, 41.752)
σ^2_ε	71.138 (68.045, 74.371)	52.542 (50.095, 55.108)	71.114 (68.022, 74.346)	52.526 (50.080, 55.092)
Outcome: SF-12 PCS				
σ^2_α	2.536 (0.892, 7.212)	1.106 (0.319, 3.830)	2.601 (0.925, 7.313)	1.140 (0.332, 3.915)
σ^2_ϕ	26.865 (22.579, 31.965)	32.940 (29.396, 36.911)	26.810 (22.532, 31.901)	32.935 (29.392, 36.904)
σ^2_ε	56.039 (53.589, 58.600)	39.139 (37.314, 41.053)	55.975 (53.528, 58.533)	39.062 (37.241, 40.972)

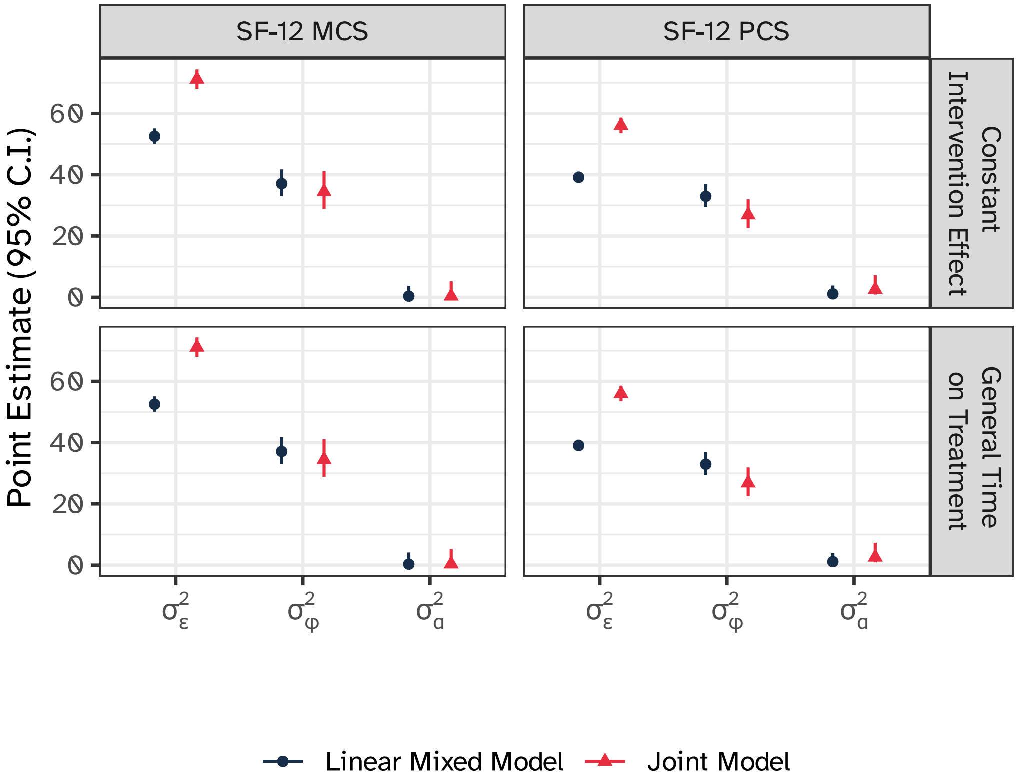

Figure D9: Estimated variance components for the SF-12 MCS and PCS outcomes, according to both the constant intervention and general time on treatment joint and linear mixed models, with 95% confidence intervals.

References

[1] W. W. Gould, J. Pitblado, and B. Poi. *Maximum Likelihood Estimation with Stata*. Stata Press, 2010.

[2] M. J. Crowther. `merlin` — A unified modeling framework for data analysis and methods development in Stata. *The Stata Journal: Promoting communications on statistics and Stata*, 20(4):763–784, 2020. doi: 10.1177/1536867X20976311.

[3] Alessandro Gasparini. `ellessenne/swjm`: Code for revision, October 2024. URL `https://doi.org/10.5281/zenodo.14013790`.

[4] E. O. Hoogendijk, H. E. van der Horst, P. M. van de Ven, J. W. R. Twisk, D. J. H. Deeg, D. H. M. Frijters, K. M. van Leeuwen, J. P. C. M. van Campen, G. Nijpels, A. P. D. Jansen, and H. P. J. van Hout. Effectiveness of a geriatric care model for frail older adults in primary care: Results from a stepped wedge cluster randomized trial. *European Journal of Internal Medicine*, 28:43–51, 2016. doi: 10.1016/j.ejim.2015.10.023.
